# Supplementary material for: Filopodia powered by class x myosin promote fusion of mammalian myoblasts
Source: eLife. 2021 Sep 14;10:e72419. doi: 10.7554/eLife.72419 (PMC8500716; doi:10.7554/eLife.72419)
Supplement: Figure 1—figure supplement 1—source data 1. [file elife-72419-fig1-figsupp1-data1.pdf]

Fig S1B&C- Extendi			
	Anterior		
Exp	Elongating filopodia (mean)	Retraction fibers (mean)	
1	17	0	
2	19.5	0	
3	10	0	
4	12	0	

ng Projections and Retraction Fibers on Undifferentiated Myoblasts

Dorsal/Side			Poster
Elongating filopodia (mean)	Retraction fibers (mean)		Elongating filopodia (mean)
5	6.5		0
1.5	4.5		0
0.5	1		0
1.5	3		0.5

ior
**Retraction fibers (mean)**
10.5
9
5
8
