# Supplementary material for: Filopodia powered by class x myosin promote fusion of mammalian myoblasts
Source: eLife. 2021 Sep 14;10:e72419. doi: 10.7554/eLife.72419 (PMC8500716; doi:10.7554/eLife.72419)
Supplement: Figure 1—figure supplement 1—source data 2. [file elife-72419-fig1-figsupp1-data2.pdf]

Fig S1D- Myocyte projection Length											
Culture Repicate #1				Culture Repicate #2				Culture Repicate #3			
Cell#	Projection#	Myoblast	Myotube	Cell#	Projection#	Myoblast	Myotube	Cell#	Projection#	Myoblast	Myotube
1	1	1.4	3.51	1	1	0.46	0.75	1	1	1	9.95
	2	1.1	2.99		2	0.59	2.14		2	3.5	2.49
	3	0.81	3.15		3	1.39	1.7		3	4.29	4.05
	4	0.81	1.58		4	2.52	1		4	1.2	1.73
	5	1.71	1.37		5	2.33	2.36		5	1.15	4.85
	6	2.47	2.07		6	1.13	2.06		6	0.84	1.46
	7	1.51	2.58		7	0.46	1.36		7	0.63	2.84
	8	0.97	3.18		8	0.98	1.2		8	1.34	3.44
	9	1.19	3.69		9	0.56	2.49		9	0.69	5.69
	10	1.96	6.86		10	0.66	1.3		10	0.97	1.38
	11	0.23	4.57		11		1.68		11	0.34	5.26
	12		4.04		12		1.67		12	0.77	3.35
	13		1.36		13		3.02		13	2.42	4.69
	14		12.66		14		2.41		14	0.86	0.86
	15		2.52		15		6.85		15	1.22	4.14
	16		3.92		16		1.32		16	0.62	0.79
	17		2.87		17		0.79		17		7.2
	18		1.48		18		2.94		18		4.83
	19		1.49		19		2.98		19		6.59
	20		1.63		20		2.39		20		1.72
	21		4.03		21		2.51		21		4.34
	22		1.4		22		1.55		22		3.56
	23		0.5		23		3.18		23		5.19
2	1	0.93	2.58		24		3.82		24		2.69
	2	1.24	1.02		25		0.88		25		2.45
	3	0.78	2.75	2	1	1.97	2.59		26		0.29
	4	2.02	1.14		2	1.55	1.71		27		4.34
	5	1.22	1.33		3	2.56	2.02		28		2.46
	6	2.72	2.18		4	2.77	1.08		29		6.56
	7	3.59	1.19		5	2.44	2.01		30		2.77
	8	0.83	1.24		6	1.99	1.49		31		3.24
	9	1.78	2.22		7	3.61	4.79		32		5.49
	10	0.75	2.28		8	5.18	3.08	2	1	1.02	2.59
	11	2.16	2.24		9	0.74	1.96		2	0.82	3.23
	12	0.5	1.37		10	0.72	1.01		3	1.8	3.58
	13		2.59		11	2.33	1.33		4	2.24	2.84
	14		2.62		12	5.52	1.82		5	1.64	1.18
	15		2.93		13	1.19	2.21		6	2.23	2.29
	16		2.81		14	0.6	3.29		7	1.29	2.48
	17		2.86		15		1.37		8	1.14	2.99
	18		3.16		16		5.84		9	1.18	4.63
	19		3.99		17		1.03		10	3.96	4.73
	20		2.03		18		2.71		11	2.81	0.77
	21		4.06		19		5.29		12		2.77
	22		3.18		20		3.18		13		2.4
	23		0.78		21		2.87		14		2.71
	24		2.6		22		3.66		15		2.77
	25		2.91		23		2.24		16		2.15
	26		4.29		24		2.05		17		6.46
3	1	1.6	2.32		25		1.12		18		5.4
	2	0.61	4.14		26		1.27		19		2.55
	3	0.58	3.79		27		2.72		20		3.31
	4	0.83	3.04		28		3.09		21		3.38
	5	1.92	3.19	3	1	1.65	0.99		22		4.06
	6	0.98	3.57		2	2.07	1.13		23		4.03
	7	1.86	3.22		3	4.93	1.38		24		2.29
	8	1.64	1.24		4	2.49	1.74		25		1.98
	9	0.42	6.62		5	0.62	2.76		26		2.19
	10	1.64	4.68		6	1.33	1.31		27		2.4
	11	2.4	0.98		7	1.78	0.44		28		2.53
	12	2.29	1.94		8	1.32	3.54		29		5.51

	13	2.8	0.79		9	0.55	2.02		30		3.4
	14	1.18	1.52		10	0.96	2.66		31		4.36
	15		2.99		11	0.56	3.46		32		2.45
	16		1.68		12		2.44		33		2.81
	17		9.02		13		4.74		34		1.73
	18		4.64	3	14		11.38		1	1.08	1.89
					15		2.01		2	1.11	1.68
					16		1.3		3	1.75	5.84
					17		1.66		4	1.65	3.77
					18		3.61		5	1.35	1.25
					19		3.14		6	1.44	1.56
					20		3.83		7	0.96	2.51
					21		2.3		8	1.18	4.29
					22		3.49		9	0.73	4.38
					23		3.34		10	0.74	2.1
					24		5.16		11	0.46	3.36
					25		2.01		12	0.73	1.39
					26		2.55		13	1.45	2.07
									14	2.31	4.09
									15	3.35	2.72
								3	16	3.39	4.72
									17		4.17
									18		3.89
									19		2.19
									20		3.06
									21		3.1
									22		5.8
									23		1.72
									24		2.18
									25		1.1
									26		2.28
									27		2.51
									28		2.11
									29		4.68
									30		3.86
									31		1.91
									32		4.46
