# Supplementary material for: Filopodia powered by class x myosin promote fusion of mammalian myoblasts
Source: eLife. 2021 Sep 14;10:e72419. doi: 10.7554/eLife.72419 (PMC8500716; doi:10.7554/eLife.72419)
Supplement: Figure 2—source data 1. [file elife-72419-fig2-data1.pdf]

Fig 2A- *Myo10* Expression in C2C12 Cells			
**Day**	**1**	**3**	**5**
Rep 1	1	2.18	9.42
Rep 2	1.21	4.53	8.91
Rep 3	1.5	6.46	6.13
