# Supplementary material for: Filopodia powered by class x myosin promote fusion of mammalian myoblasts
Source: eLife. 2021 Sep 14;10:e72419. doi: 10.7554/eLife.72419 (PMC8500716; doi:10.7554/eLife.72419)
Supplement: Figure 2—figure supplement 1—source data 1. [file elife-72419-fig2-figsupp1-data1.pdf]

Fig S2A- Myo10 protein in C2C12 cells			
**Day**	**1**	**3**	**5**
Rep 1	1.832494	2.493703	7.575567
Rep 2	0	2.90932	8.236776
Rep 3	1.167506	3.608312	6.479849
