# Supplementary figures and images for: Filopodia powered by class x myosin promote fusion of mammalian myoblasts
Source: eLife. 2021 Sep 14;10:e72419. doi: 10.7554/eLife.72419 (PMC8500716; doi:10.7554/eLife.72419)

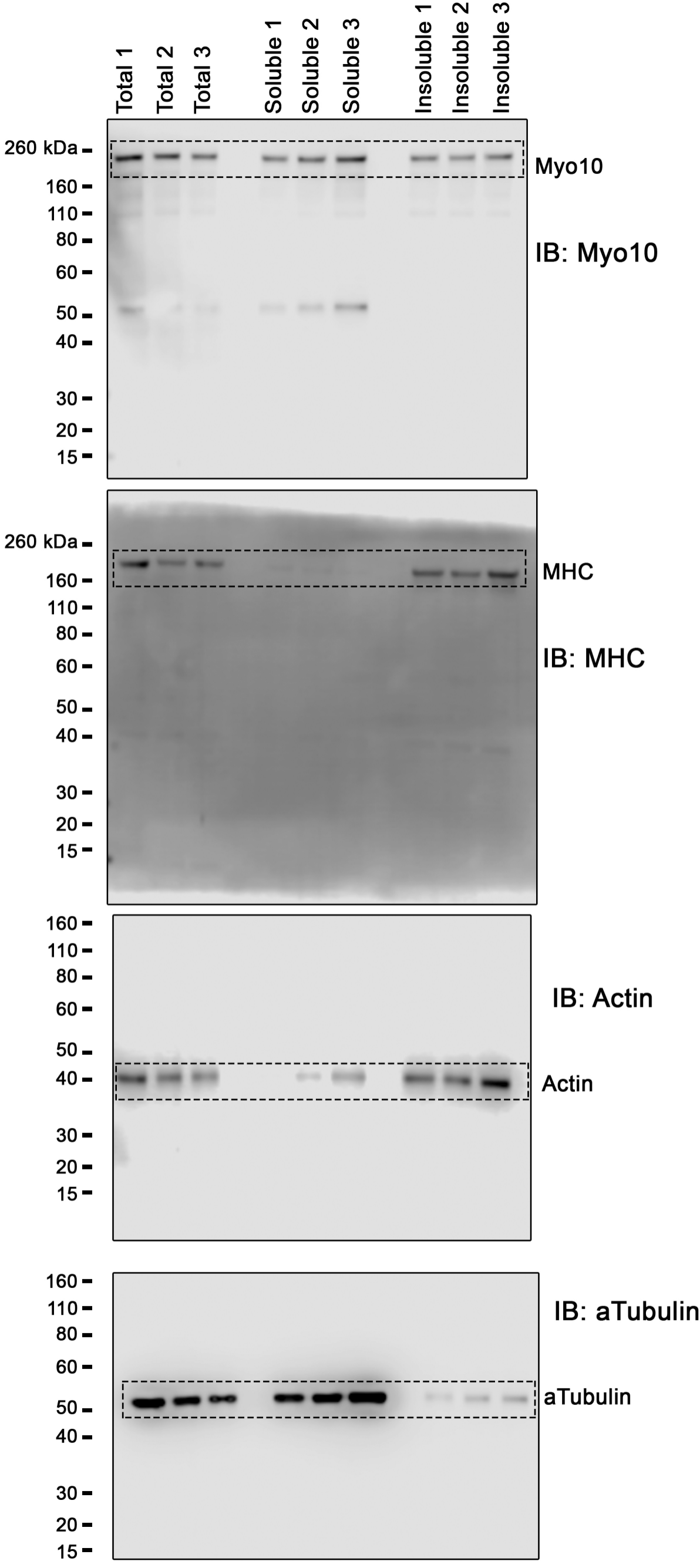

Supplement: Figure 2—figure supplement 1—source data 2. [file elife-72419-fig2-figsupp1-data2.pdf]

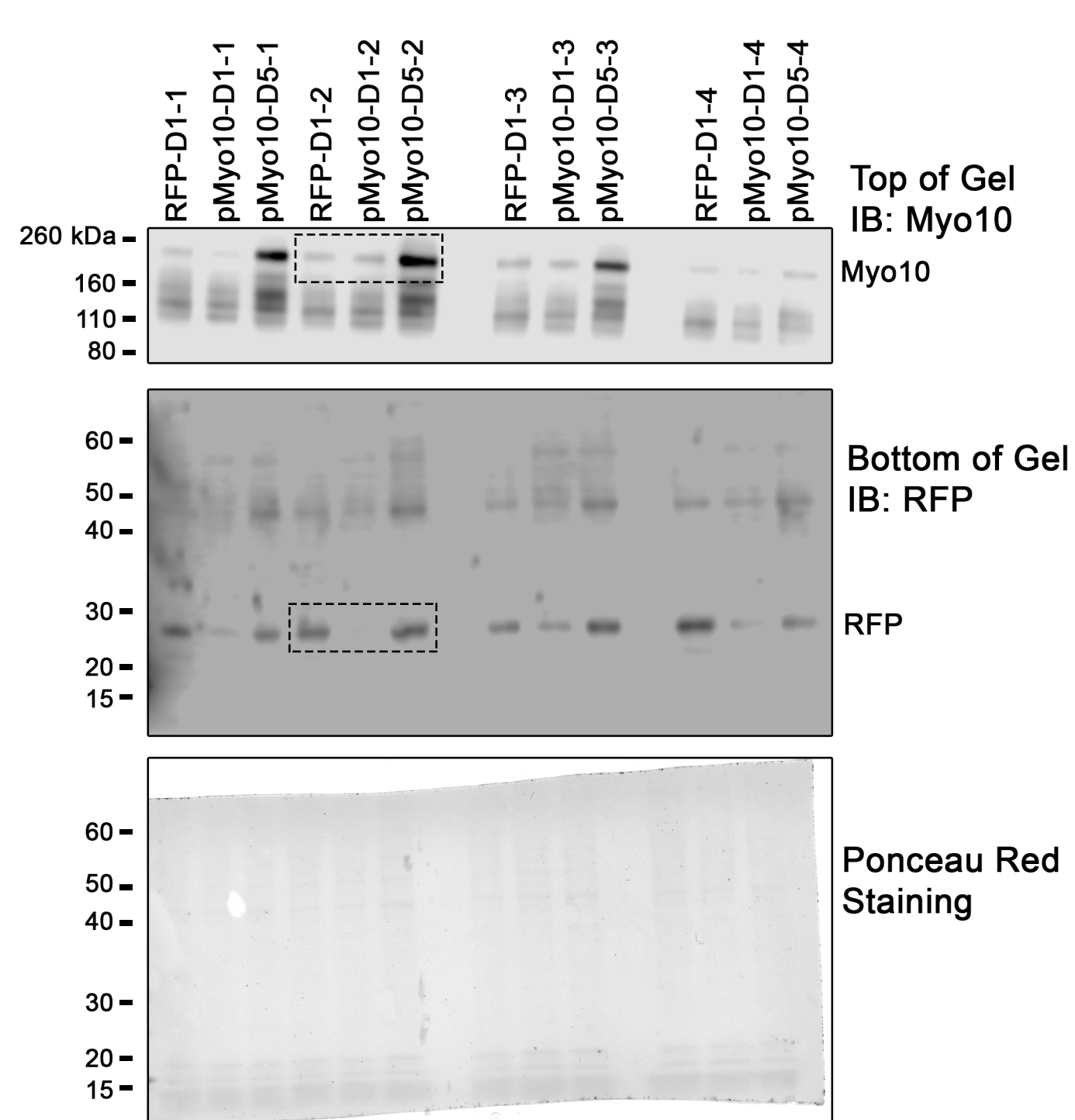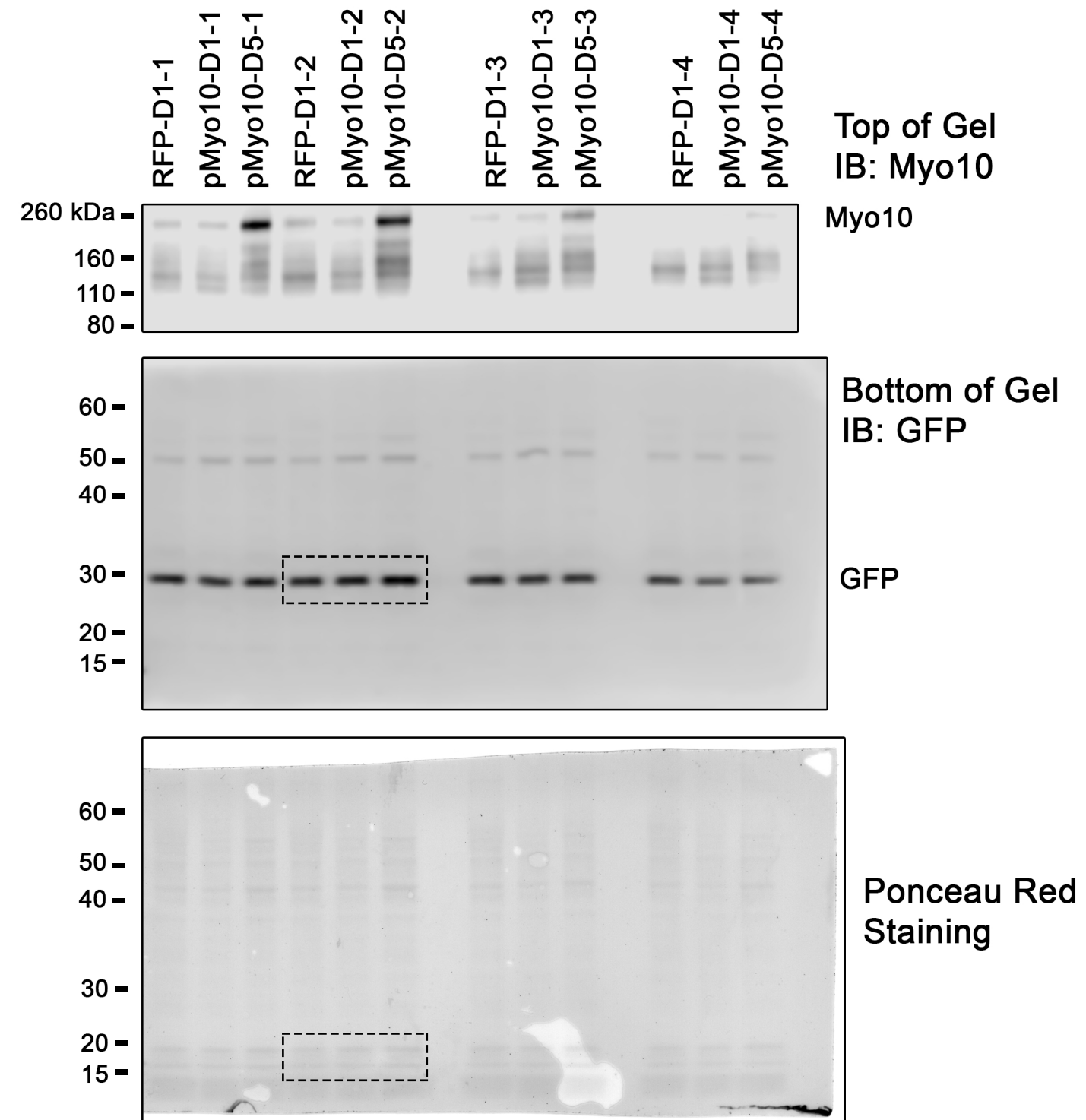

Supplement: Figure 2—figure supplement 1—source data 4. [file elife-72419-fig2-figsupp1-data4.pdf]

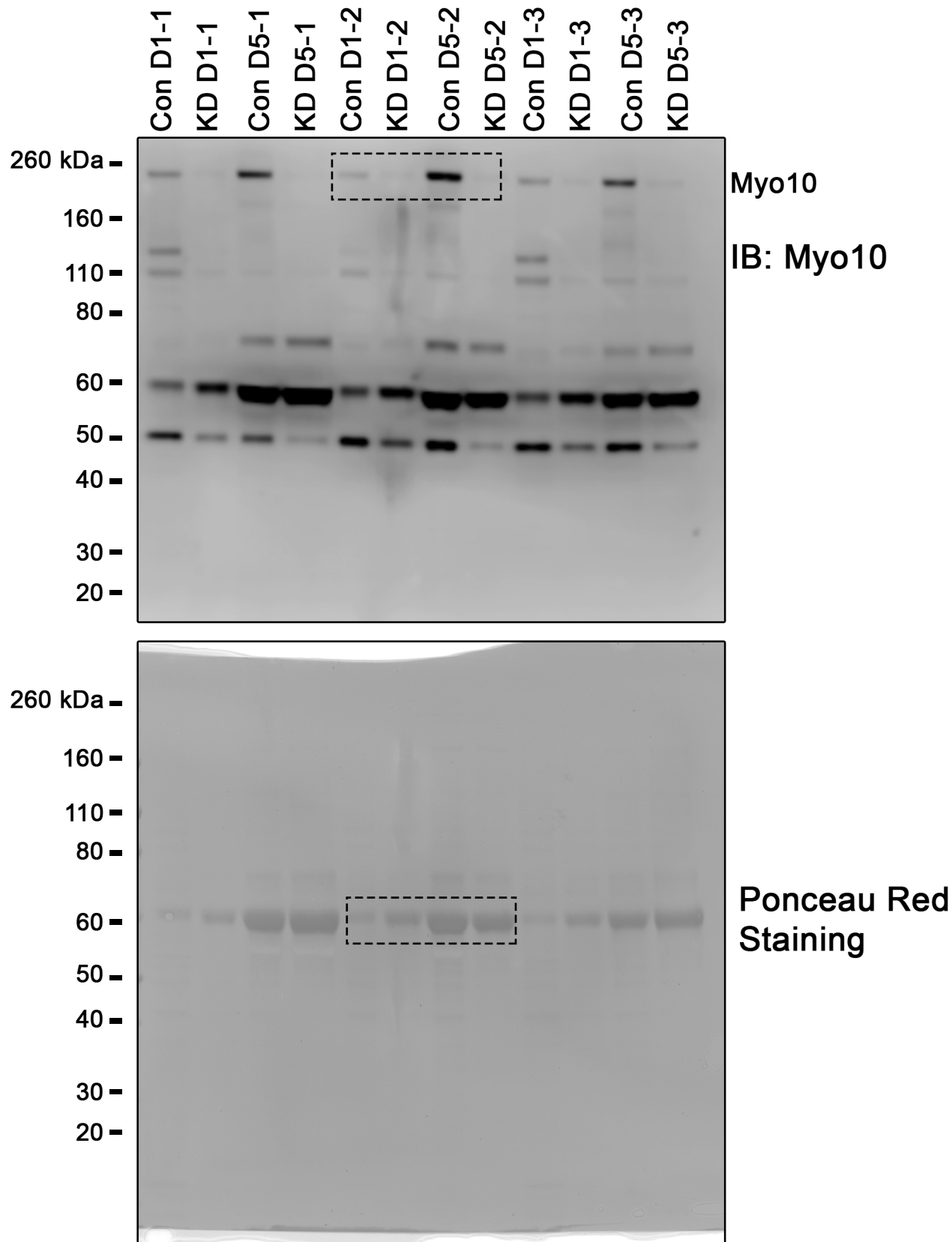

Supplement: Figure 3—source data 4. [file elife-72419-fig3-data4.pdf]

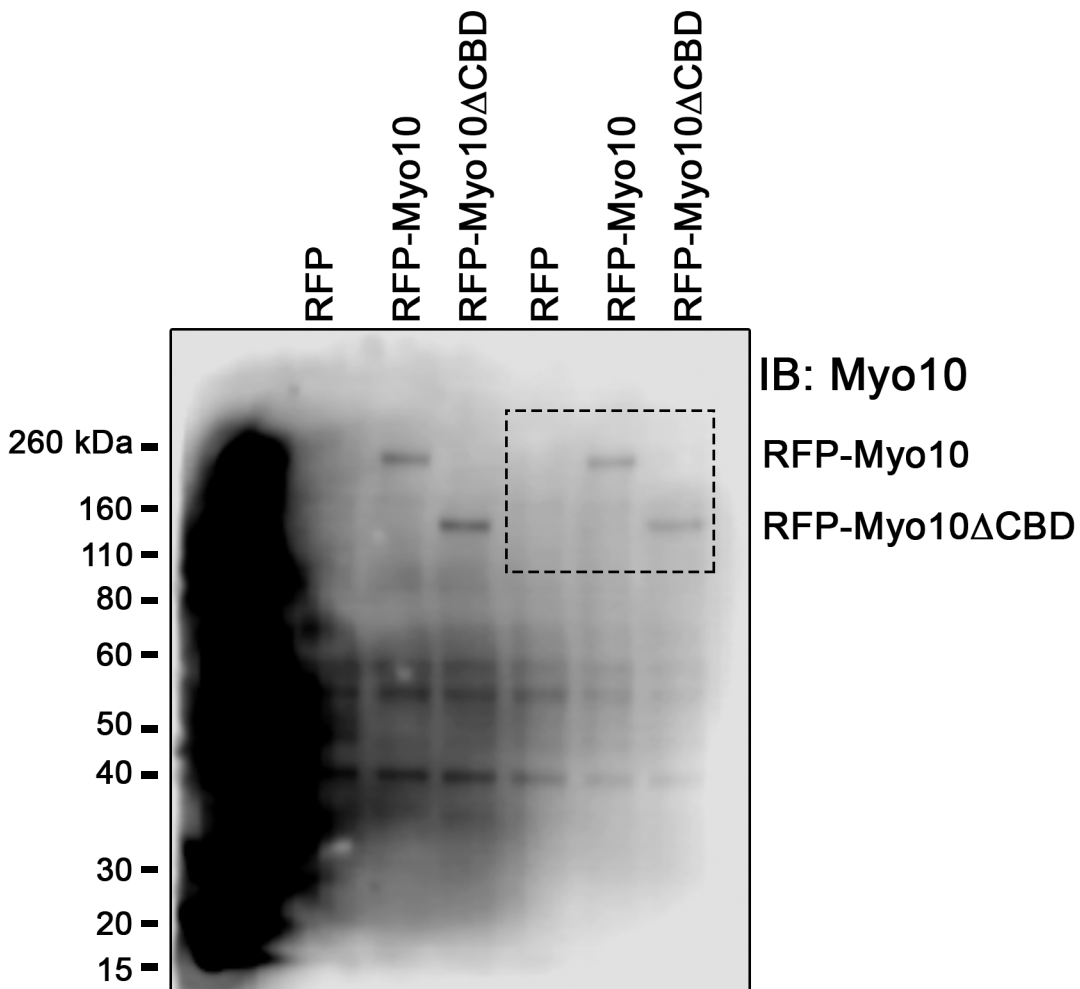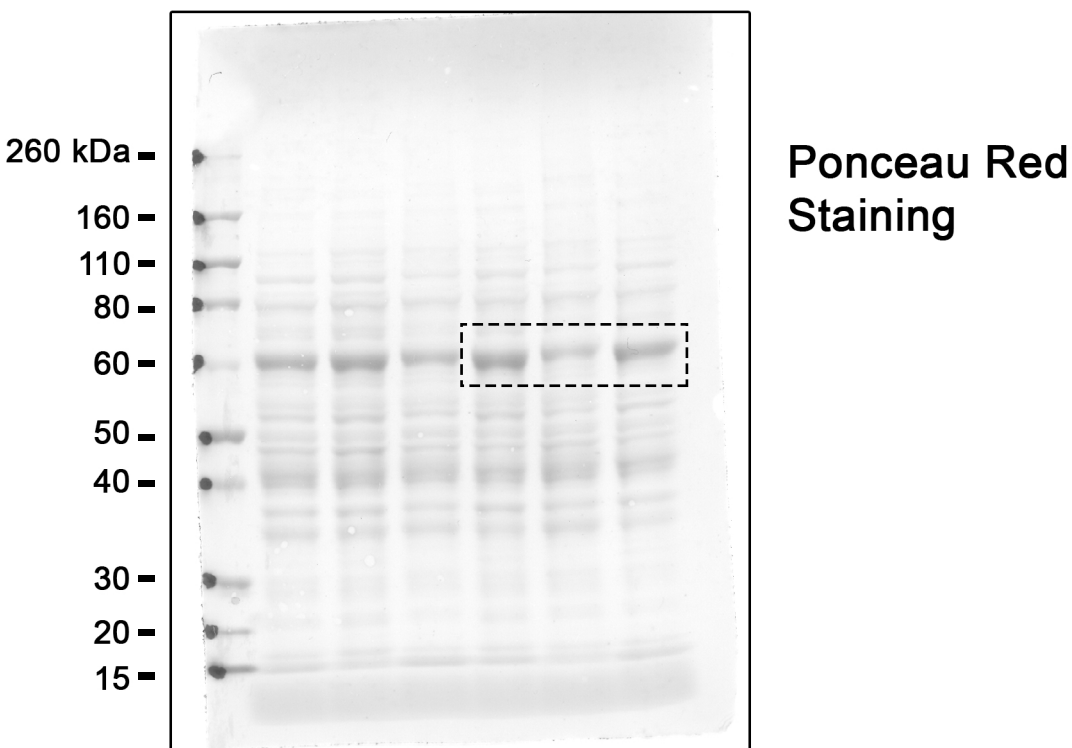

Supplement: Figure 3—figure supplement 1—source data 1. [file elife-72419-fig3-figsupp1-data1.pdf]

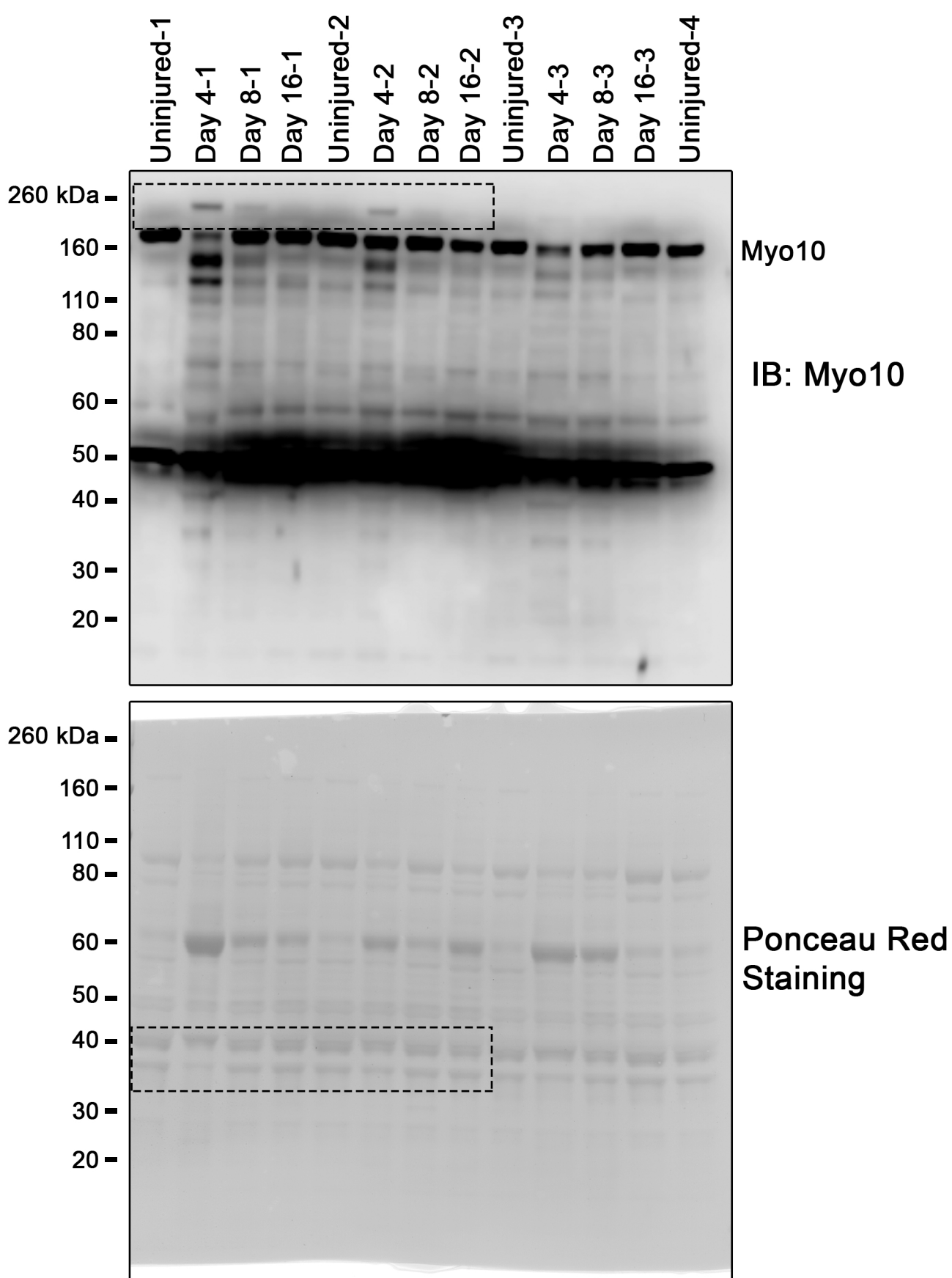

Supplement: Figure 4—figure supplement 1—source data 2. [file elife-72419-fig4-figsupp1-data2.pdf]
