# Supplementary material for: Filopodia powered by class x myosin promote fusion of mammalian myoblasts
Source: eLife. 2021 Sep 14;10:e72419. doi: 10.7554/eLife.72419 (PMC8500716; doi:10.7554/eLife.72419)
Supplement: Figure 2—figure supplement 1—source data 3. [file elife-72419-fig2-figsupp1-data3.pdf]

Fig S2E- Activation of Myo10 promoter in differentiating myoblasts			
Group	Exp	Myo10 Content (AU)	RFP/GFP Ratio (AU)
Day 1	1	0.308105207	0.731026873
	2	0.632313473	0.024183398
	3	1.621041331	2.158233826
	4	1.438539989	1.086555903
Day 4	1	10.05904455	5.412274917
	2	15.02952228	9.515767858
	3	8.760064412	13.12439948
	4	3.252818035	6.635219526
