# Supplementary material for: Filopodia powered by class x myosin promote fusion of mammalian myoblasts
Source: eLife. 2021 Sep 14;10:e72419. doi: 10.7554/eLife.72419 (PMC8500716; doi:10.7554/eLife.72419)
Supplement: Figure 2—figure supplement 1—source data 5. [file elife-72419-fig2-figsupp1-data5.pdf]

Fig 2H- *Myo10* expression at Day 0		
**Group**	**Control**	**Myo10 KD**
Rep 1	1	0.39
Rep 2	0.9	0.24
Rep 3	1.15	0.27
