# Supplementary material for: Filopodia powered by class x myosin promote fusion of mammalian myoblasts
Source: eLife. 2021 Sep 14;10:e72419. doi: 10.7554/eLife.72419 (PMC8500716; doi:10.7554/eLife.72419)
Supplement: Figure 2—figure supplement 1—source data 6. [file elife-72419-fig2-figsupp1-data6.pdf]

Fig S2I- Gene expression at Day 5				
	Myo10		***Myh2***	
	**Control**	**Myo10 KD**	**Control**	**Myo10 KD**
Rep 1	1	0.14	1	0.76
Rep 2	0.63	0.12	0.86	0.79
Rep 3	0.86	0.06	0.94	0.94
