# Supplementary material for: Filopodia powered by class x myosin promote fusion of mammalian myoblasts
Source: eLife. 2021 Sep 14;10:e72419. doi: 10.7554/eLife.72419 (PMC8500716; doi:10.7554/eLife.72419)
Supplement: Figure 3—source data 1. [file elife-72419-fig3-data1.pdf]

| Fig 3E- Cellular Extension Length | | | | | | | | |
|---|---|---|---|---|---|---|---|---|
| Cell #1 | | | Cell #2 | | | Cell #3 | | |
| Extension # | Control shRNA | Myo10 KD | Extension # | Control shRNA | Myo10 KD | Extension # | Control shRNA | Myo10 KD |
| Ext1 | 2.07 | 0.52 | Ext1 | 3.56 | 0.79 | Ext1 | 2.11 | 0.71 |
| Ext2 | 4.14 | 0.48 | Ext2 | 2.69 | 0.55 | Ext2 | 3.86 | 0.88 |
| Ext3 | 4.06 | 0.44 | Ext3 | 0.29 | 0.25 | Ext3 | 4.46 | 0.95 |
| Ext4 | 1.94 | 0.45 | Ext4 | 2.46 | 0.29 | Ext4 | 3.57 | 1.14 |
| Ext5 | 0.98 | 0.29 | Ext5 | 3.18 | 0.42 | Ext5 | 3.22 | 0.8 |
| Ext6 | 0.79 | 0.73 | Ext6 | 2.77 | 0.6 | Ext6 | 3.23 | 0.66 |
| Ext7 | 3.19 | 0.49 | Ext7 | 1.18 | 0.57 | Ext7 | 4.63 | 0.25 |
| Ext8 | 1.2 | 0.44 | Ext8 | 0.77 | 0.91 | Ext8 | 2.77 | 0.27 |
| Ext9 | 2.49 | | Ext9 | 5.4 | 0.45 | Ext9 | 4.06 | 0.11 |
| Ext10 | 1.36 | | Ext10 | 1.98 | 1.06 | Ext10 | 5.51 | 0.26 |
| Ext11 | 2.06 | | Ext11 | 2.45 | 0.46 | Ext11 | 1.68 | 0.25 |
| Ext12 | 1.3 | | Ext12 | 1.25 | | Ext12 | 4.38 | 0.5 |
| Ext13 | 1.68 | | Ext13 | 3.36 | | Ext13 | 4.29 | |
| Ext14 | 1.37 | | Ext14 | 3.15 | | Ext14 | 4.72 | |
| Ext15 | 2.72 | | Ext15 | 3.24 | | Ext15 | 3.06 | |
| Ext16 | 1.27 | | Ext16 | 2.29 | | Ext16 | 5.8 | |
| Ext17 | 2.24 | | Ext17 | 2.77 | | Ext17 | 2.18 | |
| Ext18 | 2.58 | | Ext18 | 2.55 | | Ext18 | 3.04 | |
| Ext19 | 0.44 | | Ext19 | 2.19 | | Ext19 | 5.29 | |
| Ext20 | 3.46 | | Ext20 | 2.81 | | Ext20 | 3.18 | |
| Ext21 | 1.74 | | Ext21 | 1.56 | | Ext21 | 2.87 | |
| Ext22 | 2.76 | | Ext22 | 1.39 | | Ext22 | 3.66 | |
| Ext23 | 1.12 | | Ext23 | 2.07 | | Ext23 | 3.54 | |
| Ext24 | 1.31 | | Ext24 | 4.17 | | Ext24 | 3.58 | |
| Ext25 | 2.02 | | Ext25 | 4.09 | | Ext25 | 4.73 | |
| Ext26 | 2.66 | | Ext26 | 3.89 | | Ext26 | 2.15 | |
| Ext27 | 2.05 | | Ext27 | 1.1 | | Ext27 | 4.03 | |
| Ext28 | 2.71 | | Ext28 | 2.51 | | Ext28 | 3.4 | |
| Ext29 | 1.58 | | Ext29 | 4.68 | | Ext29 | 5.84 | |
| Ext30 | 2.01 | | Ext30 | 1.91 | | Ext30 | 4.29 | |
| Ext31 | 2.3 | | Ext31 | 2.99 | | Ext31 | 5.26 | |
| Ext32 | 1.3 | | Ext32 | 5.49 | | Ext32 | 4.69 | |
| Ext33 | 1.66 | | Ext33 | 2.48 | | Ext33 | 4.14 | |
| Ext34 | 2.44 | | Ext34 | 2.4 | | Ext34 | 7.2 | |
| Ext35 | 2.55 | | Ext35 | 3.31 | | Ext35 | 6.59 | |
| Ext36 | 3.61 | | Ext36 | 2.4 | | Ext36 | 4.34 | |
| Ext37 | 4.74 | | Ext37 | 3.79 | | Ext37 | 5.19 | |
| Ext38 | 3.49 | | Ext38 | 2.59 | | Ext38 | 2.45 | |
| Ext39 | 11.38 | | Ext39 | 2.99 | | Ext39 | 4.34 | |
| Ext40 | 9.95 | | Ext40 | 2.71 | | Ext40 | 5.16 | |
| Ext41 | 3.34 | | Ext41 | 3.38 | | Ext41 | 1.24 | |
| Ext42 | 2.32 | | Ext42 | 2.53 | | Ext42 | 6.62 | |
| Ext43 | 1.38 | | Ext43 | 1.89 | | Ext43 | 4.68 | |
| Ext44 | 3.35 | | Ext44 | 1.73 | | Ext44 | 3.14 | |
| Ext45 | 0.86 | | Ext45 | 2.51 | | Ext45 | 4.05 | |
| Ext46 | 0.79 | | Ext46 | 2.72 | | Ext46 | 3.51 | |
| Ext47 | 4.83 | | Ext47 | 2.19 | | Ext47 | 3.83 | |
| Ext48 | 1.72 | | Ext48 | 3.1 | | Ext48 | 6.56 | |
| | | | Ext49 | 1.72 | | Ext49 | 2.84 | |
| | | | Ext50 | 2.28 | | Ext50 | 2.84 | |
| | | | | | | Ext51 | 6.46 | |
| | | | | | | Ext52 | 2.29 | |
| | | | | | | Ext53 | 4.36 | |
| | | | | | | Ext54 | 3.77 | |
