# Supplementary material for: Filopodia powered by class x myosin promote fusion of mammalian myoblasts
Source: eLife. 2021 Sep 14;10:e72419. doi: 10.7554/eLife.72419 (PMC8500716; doi:10.7554/eLife.72419)
Supplement: Figure 3—source data 2. [file elife-72419-fig3-data2.pdf]

Fig 3F- Myonuclei per differentiated myocyte

		Nuclei per cell											
		1		2		3		4		5		6+	
Culture#	Field of View #	Control shRNA	Myo10 KD	Control shRNA	Myo10 KD	Control shRNA	Myo10 KD	Control shRNA	Myo10 KD	Control shRNA	Myo10 KD	Control shRNA	Myo10 KD
1	1	2	4	4	1	5	0	4	0	1	0	7	0
	2	3	4	5	0	2	0	4	0	1	0	4	0
	3	2	6	7	1	5	0	2	0	1	0	7	0
	4	3	7	1	2	4	0	2	0	1	0	8	0
	5	2	7	3	1	2	0	3	0	1	0	8	0
2	1	2	5	3	1	2	0	3	0	1	0	4	0
	2	2	4	2	1	1	1	1	0	0	0	4	0
	3	2	7	1	2	3	0	1	0	1	0	8	0
	4	2	7	6	0	4	0	4	0	2	0	6	0
	5	4	6	5	1	3	0	3	0	1	0	9	0
3	1	4	6	2	2	0	0	2	0	2	0	5	0
	2	2	7	2	2	2	0	0	0	0	0	10	0
	3	3	7	3	2	3	0	0	0	1	0	10	0
	4	4	8	4	0	3	0	3	0	1	0	6	0
	5	3	6	4	1	5	3	1	2	1	0	6	0
