# Supplementary material for: Filopodia powered by class x myosin promote fusion of mammalian myoblasts
Source: eLife. 2021 Sep 14;10:e72419. doi: 10.7554/eLife.72419 (PMC8500716; doi:10.7554/eLife.72419)
Supplement: Figure 3—source data 3. [file elife-72419-fig3-data3.pdf]

Fig 3H- Differentiated Myocytes with 3+ nuclei (%)			
Culture#	Control shRNA	Myo10 KD+RFP	Myo10 KD+RFP-Myo10
1	0	24.21	51.28
2	0	24.78	46.4
3	0	25.93	52.34
4	0	21.25	
5	0		
6	2.7		
