# Supplementary material for: Filopodia powered by class x myosin promote fusion of mammalian myoblasts
Source: eLife. 2021 Sep 14;10:e72419. doi: 10.7554/eLife.72419 (PMC8500716; doi:10.7554/eLife.72419)
Supplement: Figure 3—figure supplement 1—source data 2. [file elife-72419-fig3-figsupp1-data2.pdf]

RFP_1_1	RFP_1_2	RFP_1_3	RFP_1_4	RFP_1_5	RFP_2_1
Length (um)	Length (um)	Length (um)	Length (um)	Length (um)	Length (um)
0.39	0.8	0.26	0.33	0.19	0.49
0.24	0.55	0.34	1.54	0.14	0.37
0.87	0.29	0.53	1.07	1.1	0.39
0.29	0.34		0.44	0.13	0.39
0.14			0.61	0.5	0.36
0.43				0.19	1.26
0.29				0.32	
0.28					
0.62					

FL_1_1	FL_1_2	FL_1_3	FL_1_4	FL_1_5	FL_1_6
Length (um)	Length (um)	Length (um)	Length (um)	Length (um)	Length (um)
2.47	2.29	2.58	4.2	4.76	2.94
1.92	0.69	2.88	0.96	0.58	6.43
2.33	0.34	3.53	2.15	1.07	2.8
1.46	0.88	3.19	1.96	1.15	10.62
3	1.15	4.04	1.83	1.11	1.94
4.99	1.41	3.6	2.08	2.11	12.1
6.45	1.15	4.54	1.33	1.79	4.63
3.45	3.92	2.53	2.18	3.9	4.42
7.49	1.46	2.79	3.48	1.88	2.63
3.38	4.22	1.93	2.91	3.24	1.48
2.74	7.88	3.37	1.8	3.89	4.73
3.49	3.22	5.17	1.87	3.41	3.47
1.52	3.22	3.2	5.78	4.87	3.98
1.1	1.57	3.78	6.04	1.32	7.07
1.61	1.89	2.7	7.53	0.69	6.41
0.9	6.94	2.56	1.72	5.2	7.2
2.03	1.52	2.87	3.47	1.54	5.84
1.32	2.99	3.24	5.89	3.78	5.74
3.77	1.84	2.43	2.01	3.15	3.7
4.86	2.86	2.19	1.57	1.4	7.44
0.65	1.63	3.94	1.3	2.17	3.7
3.24	0.8	5.61	3.47	5.22	4.12
3.41	7.6	4.16	3.54	2.37	6.35
3.79	10.45	1.27	2.24	5.13	8.56
	1.72	1.97	1.61		8.68

	0.88	18.24			15.87
	2.727	7.78			19.21
	1.99	6.7			9.61
	2.79	11.74			12.67
	3.33	2.12			14.57
	1.32	1.26			10.82
	4.23	1.23			11.49
	3.46	2.76			4.57
	2.47	2.52			13.69
	1.49	2.09			13.87
	2.12	0.91			4.47
	1.61	2.78			1.12
	2.77	1.89			5.14
	2.07	1.98			6.75
	1.64	2.17			10.75
	3.44	1.23			
	1.97	0.81			
	4.21	0.92			
	2.93	2.07			
	4.33	1.54			
	0.81	1.88			
	2.32	0.48			
	0.52	0.59			
	0.93	0.26			
	3.03	2.09			
	2.27	1.52			
	5.49	1.73			
	4.06	1.94			
	4.31	0.153			
	2.49	0.72			
	0.56	2.38			
	3.22	2.77			
	4.49	3.14			
	7.55	1.8			
	10.8	1.93			
	11.71	1.7			
	8.91	2.63			
	8.06				
	6.76				
	5.24				
	7.77				
	11.14				
	2.91				
	3.28				

dCBD_1_1	dCBD_1_2	dCBD_1_3	dCBD_1_4	dCBD_1_5	dCBD_1_6
Length (um)	Length (um)	Length (um)	Length (um)	Length (um)	Length (um)
6.87	3.17	1.92	1.25	3.87	4.2
1.58	3.99	1.84	6.48	4.38	5.89
2.04	4.46	3.05	4.41	1.83	6.21
2.96	3.83	4.65	5.58	4.64	2.57
1.83	3.77	4.19	4.21	3.58	2.09
2.53	5.53	3.27	6.95	2.98	4.5
1.64	2.19	5.59	10.15	0.83	1.8
2.96	9.12	4.79	1.97	1.72	4.11
4.82	5.46	3.9	7.43	2.64	7.65
3.4	1.14	4.09	2.37	4.7	8.01
5.65	3.4	7.8	3.24	6.72	5.99
1.79	3.99	8.51	2.73	1.76	8.07
6.3	7.17	6.71	1.7	2.47	0.88
5.43	7.92	2.06	6.62	2.32	1.19
2.87	1.64	4.56	3.39	5.04	4.26
5.63	1.7	12.53	8.6	3.87	3
8.03	1.43	7.02	5.66	1.98	12.97
	1.81	4.54	2.33	2.99	3.37
	2.09	3.45	4.53	2.39	6.85
		4.74	2.15	1.45	6.46
		12.05	10.14	2.15	5.79
		8.4	10.07	2.69	9.04
		7.09	12.08	4.47	4.59
		9.99	7.04	2.58	9.3
		7.52	8.89	2.75	7.75
		4.94	11.17		2.1
		5.43	6.55		
		4.8	2.43		
		3.99	7.73		
		1.05	6.67		
		1.92	5.78		
		1.84	16.81		
		3.05	15.63		
		4.65	15.59		

		4.19	7.08		
		3.27	6.13		
		5.59	6.08		
		4.79	8.2		
		3.9	8.03		
		4.09	4.58		
		7.8	4.01		
		8.51	5.05		
		6.71	6.36		
		2.06	7.36		
		4.56	3.52		
		12.53	5.98		
		7.02	6.03		
		4.54			
		3.45			
		4.74			
		12.05			
		8.4			
		7.09			
		9.99			
		7.52			
		4.94			
		5.43			
		4.8			
		3.99			
		1.05			

Fig S3C-D Projection nu

RFP_2_2	RFP_2_3	RFP_2_4	RFP_2_5	RFP_3_1	RFP_3_2	RFP_3_3
Length (um)	Length (um)	Length (um)	Length (um)	Length (um)	Length (um)	Length (um)
0.5	0.65	0.24	0.08	0.25	0.21	0.22
0.45	0.25	0.2	0.23	0.3	0.11	0.33
0.61	0.27	0.4	0.3	0.2	0.59	0.35
0.52	0.38	0.27	0.08	0.22	0.17	0.15
0.38	0.4	0.45	0.19	0.07	0.21	0.31
0.3			0.13	0.41	0.25	0.39
			0.25	0.37	0.28	0.6
			0.17		0.54	0.26
			0.34		0.34	0.34
						0.11
						0.2
						0.37

FL_1_7	FL_1_8	FL_1_9	FL_1_10	FL_2_1	FL_2_2	FL_2_3
Length (um)	Length (um)	Length (um)	Length (um)	Length (um)	Length (um)	Length (um)
5.73	1.98	1.56	3.84	2.5	5.15	2.99
2.05	1.05	3.4	3.25	1.85	1.65	4.15
1.98	1.22	2.27	4.04	3.38	3.63	2.15
5.66	0.54	5.51	1.18	3.45	5.48	2.26
3.41	1.65	10.36	4.16	1.75	3.56	5.58
6.17	3.83	4.45	1.9	4.15	2.19	6.84
8.25	3.12	10.81	0.86	4.96	7.03	3.74
1.42	5.51	5.36	1.41	1.61	2.99	2.83
1.72	1.5	4.83	2.47	3.22	3.6	5.98
5.01	5.34	2.27	3.9	5.41	3.86	2.34
2.81	6.35	3.03	4.17	3.38	2.05	3.23
3.14	4.12	7.5	2.04	4.17	5.56	4.25
6.85	5.57	7.45	2.23	2.37	10.99	4.12
6.01	3.58	8.98	1.87	4.19	3.89	1.64
4.28	3.1	9.77	1.52	4.05	4.17	1.79
3.75	4.5	6.91	1.65	2	9.05	4.62
4.58	3.33	2.03	5.51	1.45	7.06	1.25
5.66	3.69	4.83	4.12	2.62	4.17	1.88
6.65	5.23	10.36	4.19	1.44	1.71	3.49
8.73	5.33	4.45	3.31	1.71	1.89	2.12
8.78	4.51	10.81	7.23	2.45	6.43	2.46
5.32	6.37	5.36	4.46	3.24	4.36	1.17
10.69	5.89	3.03	2.34	6.71	3.9	7.65
9.36	9.44	12.29	5.64	5.32	4.6	4.34
6.08	4.45	5.83	2.28	2.54	2	5.42

7.1	5.43	11.52	2.99	3.58	4.46	2.54
9.2	4.99		2.13	4.96	6.19	2.94
10.67	7.25		7.29	5.47	3.51	4.16
6.22	6.25		4.25	6.57	9.38	5.69
5.09	7.67		0.77	6.8	9.94	4.61
5.05	9.8		5.59	6.13	6.91	5.04
4.93	11.03		5.25	6.75	6.23	7.97
3.76	12.76		9.46		4.09	4.75
10.08	6.03		12.21		3.95	4.07
6.19	3.68		5.68		8.15	2.61
4.91	6.34		4.64		4.86	5.77
	7.36		4.26		12.17	1
	1.02		5.57			7.97
	3.4		4.35			7.05
	4.21		2.61			3.82
			4.27			3.05
			3.8			1.23
						4.87
						3.73
						11.06
						7.92
						10.25
						2.4
						5.51
						9.39
						7.59
						2.92
						6.01
						12.44
						6.66
						1.08
						1.63
						2.92
						3.75
						1.83
						2.86
						4.35
						1.69
						5.17
						4.17
						1.21
						2.98
						4.24
						3.87
						3.29
						3.65
						3.75

						1.78
						1.49
						3.1
						2.22
						2.28
						1.83
						2.68
						3.65
						1.46
						2.53
dCBD_1_7	dCBD_1_8	dCBD_1_9	dCBD_1_10	dCBD_2_1	dCBD_2_2	dCBD_2_3
Length (um)	Length (um)	Length (um)	Length (um)	Length (um)	Length (um)	Length (um)
2.07	7	10.06	4.16	10.23	3.68	2.51
5.91	6.11	7.24	2.65	8.83	3.54	2.07
3.52	8.7	12.62	7.87	4.76	2.46	1.88
7.44	11.12	14.27	12.39	3.23	2.21	1.81
7.62	3.78	13.7	10.86	4.82	3.92	1.71
0.73	1.33	5.6	8.16	1.39	2.29	3.77
1.58	7.46	11.59	6.09	2.81	5.72	1.41
5.6	9.45	11.36	5.49	2.48	6.61	1.88
3.7	3.57	5.39	0.33	2.34	7.11	2.14
10	5.31	10.5	2.47	4.27	8.67	1.94
8	4.57	5.7	4.49	3.14	7.4	3.97
7.6	1.26	6.03	2.54	6.34	8.83	5.42
2.75	0.89	6.85	3.82	1.86	4.84	8.02
4	1.44	3.26	6.33	6.96	5.56	5.16
3.2	1.97	3.12	4.76	4.18	4.03	3.95
2.51	1.92	3.48	4.81	1.82	5.06	1.7
3.99	4.4	4.95	3.82	3.65	7.65	2.33
1.01	4.53	10.85	1.47	4.39	3.25	7.04
3.17	2.48	10.06	0.99	1.81	10.23	8.32
1.19	1.34	3.7	2.54	1.85	8.83	3.66
1.35	1.36	1.69	2.06	1.97	4.76	4.34
0.98	2.07	2.82	3.83	2.27	3.23	4.95
2.35	0.88	5.08	5.12		4.82	8.6
5.62	1.13	3.44	3.47		1.39	11
3.33	1.61		1.83		2.81	10.67
1.1	1.99		2.66		2.48	10.19
0.86	1.77		2.39		2.34	10.06
0.65	2.11		3.77		4.27	8.68
1.09			4.64		3.14	8.84
1.57			3.29		6.34	4.61
0.54			10.55		1.86	3.81
2.07			4.89		6.96	3.56
1.6			3.55		4.18	2.48
2.25			1.79		1.82	3.66

1.03					3.65	3.26
1.15					4.39	3.88
1.19					1.81	
2.51					1.85	
2.75					1.97	
3.2					2.27	
4						
3.37						

ımbers and lengths following expression of RFP, RFP-Myo10, or RFP-Myo10dCBD

RFP_3_4	RFP_3_5	RFP_4_1	RFP_4_2	RFP_4_3	RFP_4_4	RFP_4_5
Length (um)	Length (um)	Length (um)	Length (um)	Length (um)	Length (um)	Length (um)
0.24	0.11	0.17	0.47	0.18	0.45	0.27
0.22	0.12	0.09	0.26	0.41	0.24	0.18
0.39	0.4	0.14	0.23	0.64	0.21	0.21
0.2	0.64	0.26	0.39	0.15	0.18	0.38
0.17	0.11	0.12	0.54	0.15	0.24	0.33
0.24	0.41	0.49	0.14	0.25	0.03	0.12
0.11	0.08	0.45	0.19	0.47	0.17	0.17
0.22	0.17	0.45	0.26	0.26	0.2	0.17
0.4	0.29	0.16	0.26		0.46	0.32
	0.14	0.06	0.23		0.28	0.3
	0.15	0.3	0.27		0.14	0.38
	0.03	0.27	0.37		0.11	
		0.17	0.37			
		0.12	0.32			
		0.04				
FL_2_4	FL_2_5	FL_2_6	FL_2_7	FL_2_8	FL_2_9	FL_2_10
Length (um)	Length (um)	Length (um)	Length (um)	Length (um)	Length (um)	Length (um)
5.14	4.29	2.45	2.17	6.9	1.45	3.26
5.33	5.12	2.76	2.49	7.49	6.79	1.69
3.75	3.51	4.18	4.19	1.67	6.91	2.99
3.56	4.37	4.42	4.87	4.28	4.9	3.59
4.03	6.12	3.17	2.72	2.54	12.52	4.15
2.75	3.66	4.26	5.56	4.5	8.19	2.35
2.18	4.45	1.88	1.07	2.98	6.74	3.97
1.45	4.2	2.01	3.21	1.65	5.18	4.76
1.23	1.86	2.27	2.67	4.25	2.92	5.11
1.8	0.9	1.85	3.25	5.03	2.98	6.34
0.88	2.93	1.42	4.87	4.01	4.7	5.79
3.05	3.53	5.26	5.21	4.82	3.32	4.79
2.3	3.57	4.38	4.85	3.55	4.25	9.27
1.1	5.24	2.13	1.36	3.09	6.08	19.19
2.46	4.17	5.08	4.25	2.6	2.97	7.2
1.41	9.66	4.9	4.9	4.37	2.08	7.59
2.83	2.68	4.27	6.29	5.58	4.46	0.99
1.55	3.62	10.55	4.16	3.35	2.08	2.36
2.87	3.08	3.4	2.41	4.31	5.28	2.43
5.1	2.95	3.98	3.98	3.18	5.82	2.64
2.05	3.25	2.8	3.54	3.61	5.18	6.68
2.32	5.32	4.11	4.95	2.04	3.44	6.45
3.98	7.53	3.6	4	2.17	1.81	9
1.62	5.76	6.88	5.72	2.41	2.55	6.79
2.32	6.87	7.6	1.75	1.28	2.7	5.79

1.68	3.4	3.11	6.28	2.82	4.59	11.53
2.19	1.75	7.19	3.73	3.19	1.63	8.45
2.03	2.38	1.89	5.05	3.26	6.15	4.74
1.83	5.85	1.45	1.85	2.19	4.46	9.22
1.47	5.95	1.25	2.76	2.5	5.45	5.78
1.51	1.97	2.09	3.08	3.14	2.79	5.42
1.28	3.47	2.48	4.09	1.17	5.08	8.01
2.5	2.18	3.13	3.55	3.28	3.01	3.8
1.72	4.9	4.69	5.31	2.91	3.76	4.71
	2.98	1.53	1.64	3.86	8.07	9.52
	3.2	1.11	2.14	2.19	2.85	6.38
	2.94	1.65	3.81	2.45	7.24	6.62
	5.11	3.18	0.91	3.24	6.34	2.45
	5.91	1.49	5.59	1.5	6.02	8.55
	4.12	1.77	4.13	1.75	9.92	5.45
	4.45	3.97	10.02	4.07	4.27	5.49
	4.8	1.88	3.69	1.2	5.55	4.82
	2.63	2.8	3.79	3.21	3.01	7.57
	3.88	1.81	1.71	2.89	3.9	7.34
	2.38	3.8	6.86	3.8	5	4.36
	7.28	1.36	5.38	1.21	3.56	8.25
	0.91	1.65	3.4	1.44	4.46	3.48
	1.1	2.15	3.19	2.07	5.26	3.63
	1.03	2.16	2.26	2.81	3.91	2.52
	1.29	3.33	3.49	0.85	1.47	4.12
	2.63	3.32	2.67	2.08	3.72	1.77
	2.27	2.53	2.17	2.61	6.49	0.66
	2.55	2.29	2.55	0.54	2.73	6.89
	2.75	3.72	1.84	5.54	4.62	3.91
	3.59	3.12	2.56	2	8.92	2.74
		2.43	2.15	2.5	2.7	2.17
		3.5	4.38	3.59	6.19	
		11.87	3.31	3.3	3.7	
		7.81	1.02	3.44	4.74	
		2.9	5.71	5.78	5.24	
			4.77	4.42	4.09	
			3.39	3.87	7.14	
			2.85	4.8	6.6	
			3.54	4.21	1.69	
			2.47	4.74	5.86	
			3.28	1.48	3.91	
			2.51	3.06		
			4.48			
			4.84			
			6.03			

dCBD_2_4	dCBD_2_5	dCBD_2_6	dCBD_2_7	dCBD_2_8	dCBD_2_9	dCBD_2_10
Length (um)	Length (um)	Length (um)	Length (um)	Length (um)	Length (um)	Length (um)
1.09	4.28	1.6	9.89	2.28	1.41	1.61
3.51	4.21	4.49	7.35	3.98	1.67	1.93
3.95	2.94	3.78	4.95	3.68	1.13	12
1.23	5.03	6.88	5.11	2.62	3.49	3.09
1.7	7.45	2.03	8.53	2.83	3.5	2.18
2.93	1.62	2.39	5.52	4.23	3.32	2.5
6.76	6.77	6.81	6.34	3.21	2.99	1.4
2.56	10.89	5.43	10.44	3.5	2.92	1.52
5.89	5.03	5.68	7.72	3.48	5.34	1.52
6.53	6.29	7.38	3.53	1.63	12.64	1.92
6	2.45	9.04	3.7	3.38	1.88	2.77
8.11	3.95	6.05	1.45	4.08	12.47	7.82
6.38	4.76	11.64	1.29	3.89	2.46	1.55
1.65	2.1	9.87	2.51	4.42	3.29	1.56
3.05	2.81	4.9	1.61	3.65	2.68	1.99
4.03	5	4.13	2.84	1.77	0.88	1.96
4.88	2.64	8.44	0.89	3.1	1.18	3.06
3.87	0.8	9.41	4.31	2	1.4	3.14
5.53	2.06	2.4	1.39	3.18	2.58	3.91
5.06	4.14	3.82	3.97	3.73	2.27	4.03
5.45	2.71	3.5	1.76	9.47	2.12	4.67
4.15	2.34	2.7	1.6	2.4	3.28	4.9
1.55	1.77	6.5	1.52	2.77	2.42	3.25
2.23	2.89	4.02	1.68	6.5	3.14	2.99
1.29	2.48	3.07	3.04	7.5	2.46	2.86
1.12	1.27	1.5	1.71	6.77	2.4	1.85
0.51	3.2	3.38	1.91	2.5	2.83	6.73
2.2	6.4	1.69	1.49	2.65	2.27	4.81
3	4.53	1.3	1.05	5.41	1.99	6.5
2.34	3.5	2.27	1.19	5.25	2.58	2.77
	3.3	2.52	1.54	5.97	1.89	5.06
		2.63	1.66	2.03	2.6	5.35
		1.48	0.37	1.73	1.94	4.8
		3.22	1.11	0.92	1.71	5.84

		1.05		3.04	1.51	1.86
		0.71		2.62	2.23	4.45
		6.82		1.92	3.67	3.34
					5.15	3.4
					3.52	1.58
					4.39	2.04
					2.02	2.84
					1.53	2.35
					2.62	
					2.57	
					1.41	
					1.51	
					2.32	
					1.91	

FL_3_1	FL_3_2	FL_3_3	FL_3_4	FL_3_5	FL_3_6	FL_3_7
Length (um)	Length (um)	Length (um)	Length (um)	Length (um)	Length (um)	Length (um)
3.59	1.9	1.67	2.55	3.3	9.78	5.82
3.74	1.73	1.52	2.42	3.03	1.77	3.42
2.49	0.78	0.69	5.38	3.57	4.06	5
3.63	1.57	1.08	6.58	2.77	3.16	2.03
3.83	1.11	2.66	5.89	6.47	1.82	5.14
3.26	2.14	2.66	2.85	5.66	2.74	1.7
3.46	0.65	1.26	4.1	2.32	2.93	8.29
8.88	2.13	0.77	1.05	4.68	1.94	3.95
5.68	0.64	2.9	5.51	5.73	2.23	5.95
7.17	2.02	2.11	0.88	6.8	1.61	3.15
7.82	2.97	2.06	1.26	2.42	4.1	3.49
11	4.65	2.58	2.42	5.59	5.22	3.78
2.34	2.04	2.21	3.26	5.96	1.77	2.45
2.78	2.31	2.5	2.61	7.62	2.34	2.74
9.26	2.07	2.23	5.21	7.08	2.83	2.9
2.96	2.89	0.89	3.32	8.09	1.42	2.98
5.61	0.82	1.52	6.54	14.44	2.07	3.7
3.02	1.82	0.8	2.81	4.27	3.3	4.3
3.28	1.87	1.7	5.15	7.74	2.41	5.51
6.6	4.14	1.64	4.11	1.79	2.55	3.41
2.63	1.82	1.12	3.72	4.95	2.4	4.01
7.16	1.64	1.78	10.42	2.02	3.82	2.69
7.31	1.97	0.72	3.32	4.04	3.45	5.41
8.97	2.56	2.39	7.46	2.44	3.17	4.88
3.14	2.56		9.27	3.48	1.61	6.36

7.19	5.08		2.16	4.66	10.06	
7.18	1.64		5.21	3.1		
8.81	2.11		2.85	2.44		
10.72	1.13		2.87	0.8		
5.58	3.38		13.03	2.81		
3.68	2.66		4.78	3.5		
4.12	5.28		4.18	1.9		
3.8	1.26		0.86	2.29		
4.84	3.08			1.46		
5.73	1.21			1.87		
10.89	2.87			2.13		
3.86	3.99			7.23		
6.44	5.31			6.9		
7.55	2.86			1.64		
5.14	2.87			4.22		
4.58				3.26		
6.4				1.79		
6.85				7.74		
6.91						
4.3						
3.85						
4.62						
2.09						
1.48						
1.48						
1.45						
3.67						
2.09						
1.6						
1.35						
2.78						
1.89						
2.31						
1.81						
3.65						
0.55						
2.31						
1.6						
1.21						
3.01						
1.31						
2.05						
2.89						
2.09						
1.83						

dCBD_3_1	dCBD_3_2	dCBD_3_3	dCBD_3_4	dCBD_3_5	dCBD_3_6	dCBD_3_7
Length (um)	Length (um)	Length (um)	Length (um)	Length (um)	Length (um)	Length (um)
0.98	1.52	1.5	0.78	2.6	1.08	2.43
2.03	1.07	1.59	1.52	4.2	0.36	1.58
1.07	1.3	0.98	1.95	2.79	0.85	2.5
1.68	0.88	0.16	2.7	2.65	0.97	3.58
1.4	0.74	0.63	2.31	5.09	1.16	3.37
1.12	1.38	1.4	1.24	4.34	1.06	1.15
0.16	1.1	1.48	1.85	1.77	0.87	8
2.79	1.22	1.54	1.99	0.97	1.73	9.13
2.89	2.26	1.5	1.68	1.2	1.24	2.2
4.6	1.66	0.92	1.69	0.82	2.14	1.06
2.75	1.97	1.56	2.7	0.43	0.69	2.22
1.53	2.54	1.34	1.66	2.22	1.06	2.35
1.68	1.13	1.13	1.64	1.24	0.62	5.76
3.86	1.8	0.64	1.53	3.75	2.65	4.39
3.2	1.22	1.06	1.47	0.51	1.22	1.12
4.2	7.26	1.44	0.75	0.58	1.02	2.12
2.79	3.44	0.98	0.65	5.63	2.48	1.04
5.18	0.85	0.94	0.51	0.77	1.16	1.31
3.81	3.93	1.51	2.28	1.15	2.28	2.77
5.43	5.41	1.25	1.27	4.84	2.18	1.33
3	3.5	3.26	3.68	4.04	1.48	4.63
3.93	7	2.2	2.43	2.14	0.71	3.55
1.53	2.48	2.61	0.98	1.62	0.51	0.88
1.68	2.11	2.32	1.87	2.27	1.73	1.04
	1.61	3.53	0.72	0.99	2.37	0.78
	1.02	3.14	1.71	0.84	2.32	3.49
	0.69	3.76	0.96	1.52	2.99	1.31
	0.77	1.03	0.72	0.79	2.96	2.77
	1.2	3.32	1.41	0.66	5.05	1.33
	2.24	1.28	0.84	0.68	12.76	3.49
	1.02	1.8	10.5	0.73	3.38	2.72
		1.1	1.24	0.58	5.06	4.64
		1.99	1.15	0.67	5.85	1.15
		1.43	0.51	0.67	5.93	2.65

		0.73	1.48	1.93	3.65	1.62
		1.54	2.06	3.35	2.19	0.8
		9.02	1.12	4.99	2.19	0.71
		6.7	1.23	2.27	2.28	0.62
		8.29	0.53	1.62	1.16	1.24
		7.98	1.43	2.14	2.48	1.63
		9.71	3.02	1.94	1.02	1.8
		10.07	3.05	0.95	0.9	1.91
			3.38	0.72	1.7	0.22
			2.38	0.28	3.72	
			2.26	1.4	2.84	
			1.7	0.45	4.82	
			2.52	0.86	5.92	
			1.47	0.67	5.04	
			0.35		2.33	
			0.58		2.28	
			0.65			
			2.2			
			2.17			

FL_3_8	FL_3_9	FL_3_10
Length (um)	Length (um)	Length (um)
1.97	3.65	2.14
3.81	4.28	1.62
5.85	4.07	2.35
3.98	2.46	1.11
6.64	7.1	1.58
4.38	2.98	4.22
3.05	9.48	0.69
1.62	4.49	13.17
4.06	2.1	7.39
6.63	4.32	11.53
4.56	10.16	9.33
4.83	17.57	11.81
5.5	11.94	9.38
8.27	7.45	9.99
6.91	3.79	4.48
8.06	5.72	3.6
8.81	8.79	3.63
11.57	18.51	2.63
10.72	6.15	0.91
8.88	4.81	1.17
5.65	2.5	0.92
3.72	2.1	3.38
9.28	2.58	1.61
6.63	6.59	1.95
5.68	3.88	2.55

1.66	5.83	3.19
5.38	1.61	1.96
3.04	3.69	3.59
3.77	3.53	2.57
3.34	4.29	1.74
3.85	2.81	3.41
3.62	1.8	1.91
4.11	4.19	3.66
4.22	2.49	1.48
3.5	4.49	
3.3	4.47	
5.5	2.73	
6.03	4.16	
5.05	4.67	
4	2.16	
1.29	6.7	
3.11	3.11	
0.98	3.17	
1.07	3.82	
3.45	3.93	
	4.25	
	5.73	
	5.55	
	2.21	
	1.76	
	2.7	
	1.28	

dCBD_3_8	dCBD_3_9	dCBD_3_10
Length (um)	Length (um)	Length (um)
2.23	3.57	1.05
1.63	1.37	0.78
1.05	0.81	1.89
0.66	2.32	0.92
3.66	3.6	0.76
4.39	4.61	0.88
2.04	5.9	0.64
1.89	2	0.78
2.8	4.48	0.67
3.09	3.44	0.88
3.46	8.85	1.72
1.67	11.92	1.22
2.06	5.38	1.24
1.27	5.9	0.77
1.31	6.01	1.72
3.03	2.77	0.83
1.2	5.26	4.94
0.7	3.33	6.17
0.49	13.46	1.99
2.18	4	1.43
1.65	5.77	1.47
1.54	5.55	4.66
1.83	4.56	4.72
1.6	5.61	1.34
1.69	5.86	0.59
1.51	5.5	1.33
4.29	5.29	4.66
3.75	6.96	0.68
3.78	5.51	2.4
12.3	9.76	2
5.92	2.41	1.79
3.02	3.69	1.65
1.31	6	2.4
1.69	6.52	3.75

1.52	6.61	3.74
1.51	1.66	4.29
2.54	0.68	1.63
2.6	0.96	2.82
1.05	2.15	3.19
2.33	0.58	2.23
1.13	1.03	3.24
2.2	0.71	1.82
0.74	2.38	2.87
1.81	5.94	2.05
10.61	2.18	5.54
2.23	1.14	0.44
2.04	2.74	6.51
5.34	1.58	5.92
1.73	2.97	3.03
0.36	2.69	1.84
1.87	1.81	2.65
0.15	1.21	0.75
2.56	2.84	1.52
2.33	2.61	1.1
	1.47	1.68
	3.83	1.05
	0.42	0.78
	1.43	0.92
	2.13	
	2.02	
	1.45	
	2.77	
