# Supplementary material for: Filopodia powered by class x myosin promote fusion of mammalian myoblasts
Source: eLife. 2021 Sep 14;10:e72419. doi: 10.7554/eLife.72419 (PMC8500716; doi:10.7554/eLife.72419)
Supplement: Figure 3—figure supplement 1—source data 3. [file elife-72419-fig3-figsupp1-data3.pdf]

Fig S3B- Myoblast fusion requires Myo10 cargo binding							
Control shRNA				Myo10 KD + RFP			
Exp	Total	3+	%	Exp	Total	3+	%
1	187	58	31.01604	1	100	1	1
2	233	51	21.88841	2	135	0	0
3	79	19	24.05063	3	73	0	0
4	173	42	24.27746	4	149	0	0
5	271	84	30.99631	5	41	0	0
6	267	51	19.10112	6	75	0	0

; domains			
Myo10 KD + RFP-Myo10ΔCBD			
Exp	Total	3+	%
1	35	0	0
2	69	0	0
3	44	0	0
4	109	0	0
5	60	0	0
6	79	0	0
