# Supplementary material for: Filopodia powered by class x myosin promote fusion of mammalian myoblasts
Source: eLife. 2021 Sep 14;10:e72419. doi: 10.7554/eLife.72419 (PMC8500716; doi:10.7554/eLife.72419)
Supplement: Figure 3—figure supplement 2—source data 1. [file elife-72419-fig3-figsupp2-data1.pdf]

Fig S4C- Mixed Fusion Assay			
	% of Labeled Myotubes		
**Experiment**	**Green Only**	**Red Only**	**G+R**
Exp 1	80	20	0
	100	0	0
	100	0	0
	100	0	0
Exp 2	85.71428571	0	14.28571
	100	0	0
	100	0	0
	100	0	0
Exp 3	100	0	0
	100	0	0
	100	0	0
	100	0	0
Exp 4	85.71428571	14.28571	0
	100	0	0
	100	0	0
	100	0	0
Exp 5	100	0	0
	90	10	0
	100	0	0
	100	0	0
