# Supplementary material for: Filopodia powered by class x myosin promote fusion of mammalian myoblasts
Source: eLife. 2021 Sep 14;10:e72419. doi: 10.7554/eLife.72419 (PMC8500716; doi:10.7554/eLife.72419)
Supplement: Figure 4—source data 1. [file elife-72419-fig4-data1.pdf]

Fig 4G-H- Muscle Fiber Diameters at Day 8 following CTX											
Muscle Sample #1		Muscle Sample #2		Muscle Sample #3		Muscle Sample #4		Muscle Sample #5		Muscle Sample #6	
Pax7-WT	Pax7-M10cKO	Pax7-WT	Pax7-M10cKO	Pax7-WT	Pax7-M10cKO	Pax7-WT	Pax7-M10cKO	Pax7-WT	Pax7-M10cKO	Pax7-WT	Pax7-M10cKO
22.647	15.796	22.187	20.522	14.88	14.435	16.57	20.879	21.854	24.466	39.237	26.256
17.098	28.981	24.282	14.198	18.049	14.128	17.241	24.728	23.062	11.267	20.225	32.711
24.615	16.484	7.764	17.241	29.881	11.131	14.51	20.105	20.251	22.929	13.426	12.161
33.465	22.026	21.59	9.255	19.194	17.241	19.029	15.693	25.896	10.423	16.343	25.862
20.869	24.095	18.53	19.538	35.878	26.66	19.403	22.759	16.417	11.338	29.038	21.445
22.414	14.531	11.8	15.871	16.17	14.368	10.92	18.818	25.803	19.549	22.508	9.744
29.064	17.816	22.32	7.093	15.998	20.69	19.088	10.972	21.264	27.485	32.471	20.497
18.88	10.312	24.259	11.247	15.246	13.286	19.889	13.793	16.092	22.057	12.045	15.249
16.132	20.111	27.9	8.689	14.913	25.287	12.596	10.547	23.597	16.149	16.38	25.201
19.433	19.137	18.844	13.969	12.93	12.357	20.056	24.127	35.877	35	10.703	53.97
30.501	13.444	32.704	16.939	9.647	17.397	17.241	15.175	15.916	23.115	35.35	39.796
31.256	5.747	23.436	11.494	18.966	32.107	19.214	15.508	22.91	18.921	24.886	12.579
20.408	16.052	30.779	14.762	14.08	7.974	7.047	7.275	21.241	29.182	14.863	13.137
18.621	5.564	11.254	12.617	15.954	23.647	14.123	17.693	23.107	37.086	22.886	16.253
33.809	14.281	25.494	10.964	24.789	14.571	24.245	7.93	31.631	14.043	26.653	15.618
28.326	7.278	9.218	15.359	24.713	24.077	17.699	6.919	21.591	17.974	28.559	29.849
20.232	8.302	21.796	16.306	15.517	14.223	9.539	10.8	12.606	22.641	40.625	24.641
24.713	13.813	18.722	19.217	10.66	12.143	15.801	7.454	15.131	29.064	13.368	18.004
24.491	8.046	26.112	12.345	22.814	17.02	16.992	25.035	27.4	11.712	21.639	13.073
26.195	17.481	23.769	10.54	20.946	13.522	13.112	36.527	19.798	28.16	18.342	10.345
25.731	30.038	13.815	14.022	26.726	12.818	17.002	34.483	22.593	20.873	13.636	39.08
17.33	17.635	41.17	11.494	14.796	26.635	18.868	11.729	49.463	16.637	26.239	7.592
14.936	11.555	15.362	18.391	12.533	12.357	10.612	20.58	32.563	22.989	16.381	10.118
38.442	24.169	30.382	17.252	14.121	13.793	14.72	20.184	17.766	13.793	18.802	15.714
28.288	15.695	25.515	13.311	22.763	25.212	10.566	41.193	20.87	22.063	24.222	15.884
27.042	6.897	20.69	10.388	8.361	12.069	23.706	8.174	16.258	21.39	18.966	8.046
24.83	10.345	26.124	14.04	29.637	6.579	22.004	27.725	18.391	37.945	18.353	11.139
24.408	7.159	24.424	22.247	10.228	21.118	17.938	43.724	21.174	25.141	20.131	10.345
26.558	10.92	21.465	24.819	12.943	13.681	17.241	17.757	16.956	13.703	20.034	12.553
19.937	23.071	17.816	18.2	9.412	12.36	10.345	26.93	16.996	26.961	25.865	24.938
32.253	12.199	25.285	10.92	15.855	18.096	23.579	16.665	21.244	34.461	14.486	21.291
34.924	15.443	19.279	9.615	21.245	17.459	18.501	23.609	29.562	23.634	20.47	9.145
27.877	22.929	20.383	21.633	14.486	12.508	23.659	17.613	31.382	17.389	24.888	11.899
22.187	17.391	29.311	8.407	15.607	15.476	19.971	17.559	34.088	27.561	26.961	18.428
20.037	24.397	15.995	8.331	25.213	15.273	26.221	22.018	30.885	21.649	22.7	20.119
38.839	14.318	35.863	11.588	16.779	27.924	16.037	5.43	29.984	21.251	27.315	17.052
30.919	20.647	40.399	10.926	26.643	16.967	12.069	9.77	19.297	13.587	24.749	27.109
31.618	20.843	42.068	5.805	28.156	5.747	18.424	10.566	24.109	12.286	19.684	32.579
23.192	21.061	45.454	8.882	17.012	18.825	13.825	18.809	18.391	19.596	25.284	11.398
26.171	15.869	35.632	7.113	19.865	20.472	11.572	22.566	21.142	20.94	30.308	12.077
31.527	24.115	20.549	21.682	21.106	12.271	16.398	29.46	20.566	20.156	35.044	17.241
32.759	17.153	22.001	27.314	30.574	8.061	23.532	21.046	18.392	13.549	27.701	20.415
34.373	12.179	17.272	19.037	24.423	27.83	24.686	12.062	33.924	15.001	62.745	16.018
19.962	12.757	17.459	31.473	11.689	25.745	14.402	17.189	23.551	23.308	32.385	12.151
27.558	11.956	24.358	11.942	14.716	7.045	13.502	22.426	33.591	14.363	17.596	12.598
28.029	24.585	21.489	14.626	24.171	25.287	8.486	14.368	26.503	14.754	37.964	32.773
34.087	17.93	18.779	17.188	14.021	22.069	15.655	12.899	21.313	12.952	25.439	14.251
35.713	37.996	30.212	12.399	27.894	18.465	17.266	22.34	27.731	12.644	24.887	19.445
17.181	21.057	24.61	19.807	30.466	22.365	15.36	18.124	26.277	10.694	20.929	23.326
17.773	26.192	25.252	9.861	30.184	13.107	21.442	18.549	29.467	14.794	14.895	22.88
28.736	15.643	15.941	20.849	33.696	23.703	12.412	14.795	25.832	15.63	14.91	22.601
23.882	16.015	14.862	15.764	15.461	9.261	23.624	14.84	17.816		16.046	26.225
24.843	24.972	28.125	9.607	13.974	13.162	22.932	8.888	17.268		13.102	16.289
19.54	15.938	21.706	24.635	26.78	31.107	12.882	29.321	21.072		14.446	12.717
13.998	17.816	23.624	18.082	34.088	12.021	16.011	12.162	17.207		16.997	28.072
16.299	17.805	33.733	17.479	29.462	17.009	10.382	20.097	39.314		23.437	19.514
19.807	26.369	27.76	9.738	22.332	7.457	16.076	14.754	22.426		18.649	16.066
21.122	9.484	17.427	10.345	24.597	15.176	24.23	14.712	37.991		14.685	13.437
35.697	8.913	29.169	9.102	17.504	14.453	20.999	14.891	35.131		22.438	26.359
16.168	15.841	15.977	9.483	29.251	12.55	20.122	7.464	40.929		28.136	32.12
16.557	14.432	18.362	7.315	25.892	14.917	6.017	9.639	26.432		32.511	20.336
27.74	22.807	14.008	13.813	12.175	11.614	14.869	24.183	33.165		15.602	19.652
29.634	32.558	19.506	12.829	16.453	10.26	22.594	20.903	29.207		24.414	13.494
36.646	12.431	26.176	5.849	15.927	20.469	10.851	12.574	43.996		10.391	27.533
27.781	30.002	18.694	13.55	15.975	16.109	23.265	18.821	29.114		17.147	17.16
36.767	32.359	21.244	11.961	21.839	16.541	6.322	18.545	29.58		12.726	16.204
23.1	36.84	23.45	16.815	26.04	20.981	20.9	15.428	26.989		15.596	12.144
12.445	10.712	31.428	14.222	18.689	13.229	13.487	7.288	23.488		14.464	25.896
16.757	19.105	21.344	9.737	28.781	11.053	12.506	10.345	23.532		14.057	17.877
37.476	33.04	21.502	6.322	35.194	9.721	24.253	13.793	18.576		24.636	14.988

24.185	23.226	17.188	8.94	15.399	11.655	13.575	12.379	32.941		22.626	12.193
18.573	20.115	20.286	29.361	24.903	9.955	22.414	21.538	12.644		11.067	18.175
20.456	15.517	18.73	18.029	12.606	18.922	29.773	20.274	33.077		17.375	27.011
17.33	20.856	25.107	10.657	32.269	12.539	19.903	14.123	23.215		13.793	21.052
19.477	7.964	22.593	13.793	23.819	23.29	15.447	24.66	32.642		19.776	11.836
35.4	16.092	10.675	10.738	22.55	6.873	24.677	18.546	28.177		17.799	14.398
20.997	12.644	13.015	12.665	30.264	10.215	18.687	16.19	26.837		24.261	17.241
25.65	17.745	16.401	26.688	34.059	18.051	25.855	9.579	31.496		15.803	25.287
32.857	10.061	22.643	20.69	19.158	15.491	31.235	19.234	30.46		36.672	16.065
17.947	13.757	29.81	13.387	29.646	18.169	26.687	9.756	20.018		24.739	14.469
23.927	15.982	19.773	16.638	17.535	14.377	19.752	14.363	21.429		16.272	7.162
11.222	12.774	24.872	24.713	20.388	9.407	24.954	26.313	26.784		9.47	22.066
24.153	13.222	47.091	13.274	30.407	16.796	21.907	24.985	27.783		31.121	14.273
13.958	13.259	30.479	25.372	27.739	9.956	22.847	9.608	18.299		28.579	16.199
20.21	14.106	33.908	20.699	25.849	20.69	25.456	14.736	28.178		10.338	28.287
27.216	16.364	29.252	11.661	33.498	12.286	21.495	18.415	29.52		12.644	19.555
20.367	26.754	35.702	15.41	18.805	17.295	20.084	18.863	25.862		17.205	10.566
19.772	9.424	48.104	3.959	24.522	21.119	13.793	11.772	29.665		13.793	18.966
22.249	10.416	25.701	13.354	14.178	13.869	15.754	11.251	30.628		18.805	34.949
22.692	11.201	18.299	10.217	31.558	16.907	22.078	10.685	26.437		17.621	10.11
25.095	13.551	25.53	25.268	28.501	16.81	11.794	8.786	24.624		16.647	16.897
9.775	39.416	30.4	15.035	33.656	16.575	18.117	12.335	24.797		26.343	47.323
31.864	11.016	18.958	15.389	32.521	14.061	23.3	19.222	28.817		19.789	20.929
20.502	8.621	18.391	26.821	41.007	15.236	16.766	14.324	31.129		20.719	18.958
19.01	9.006	28.702	10.507	37.433	8.449	24.981	42.015	31.686		23.702	18.976
20.362	22.989	22.115	12.649	25.604	11.555	16.113	20.627	27.011		22.692	18.966
11.867	23.453	31.913	13.112	35.36	14.622	23.195	10.091	19.363		22.582	13.377
15.607	7.59	37.298	24.518	21.408	8.875	32.774	6.572	30.922		39.436	10.855
15.717	15.041	35.309	14.305	29.765	12.598	28.06	16.763	27.727		24.138	14.424
17.727	22.895	40.192	18.416	26.787	20.479	25.324	9.831	27.464		12.193	28.058
24.303	24.938	25.537	20.845	13.248	22.051	24.801	13.342	19.419		23.017	13.484
10.067	16.11	26.112	11.396	28.173	29.428	26.678	33.757	23.721		31.237	17.972
18.184	11.198	17.284	9.961	25.564	23.526	25.438	23.563	25.503		34.689	14.977
8.654	18.772	24.068	23.475	35.632	10.674	24.613	10.069	32.655		13.767	15.329
15.999	20.777	29.121	16.451	16.331	31.546	19.511	3.113	24.887		15.347	31.767
11.502	19.187	21.138	13.609	20.315	9.848	33.082	18.425	26.4		23.434	19.22
21.735	13.94	25.52	12.597	33.73	13.669	16.422	21.645	23.954		19.599	24.867
31.043	19.613	43.553	13.955	30.372	11.016	13.863	9.414	26.922		21.353	13.817
16.429	6.692	21.865	19.895	31.321	6.143	29.634	14.949	19.094		21.062	19.492
25.168	4.989	28.992	26.775	23.864	33.504	21.751	25.88	22.989		23.988	18.371
12.585	14.053	24.283	13.994	40.066	20.793	24.383	19.763	16.859		13.588	23.917
22.199	14.221	29.658	25.298	29.849	34.866	29.64	9.347	33.094		23.563	13.505
20.771	25.291	23.985	8.641	26.917	19.601	26.484	14.368	22.222		28.377	19.064
18.264	16.322	28.076	14.67	33.164	12.965	25.192	24.715	30.177		24.245	23.88
30.835	9.43	25.477	14.886	28.327	28.738	23.561	16.263	27.828		19.581	31.604
25.295	20.041	41.04	17.131	32.648	23.256	25.196	13.208			34.426	17.826
25.502	17.692	11.907	13.439	19.458	11.712	18.615	15.603			25.182	12.001
15.818	10.615	14.219	14.03	23.914	11.923	16.834	24.038			31.216	29.008
15.944	7.994	18.895	13.344	36.186	28.284	32.029	10.429			29.439	15.527
26.435	15.928	12.717	17.645	24.138	18.249	28.735	12.276			29.431	17.326
12.931	19.976	25.993	19.184	30.4	19.741	35.057	10.92			18.627	16.361
26.894	9.537	29.729	22.119	23.371	19.321	25.114	15.607			23.641	14.518
13.3	16.882	30.534	26.98	29.666	14.097	26.544	19.936			33.726	23.576
28.161	11.632	15.2	14.368	26.927	15.598	46.731	22.187			23.58	25.316
13.225	14.731	26.828	23.083	28.975	14.111	18.164	13.753			30.242	18.391
32.445	18.007	17.625	19.962	26.814	28.999	37.928	31.124			25.489	27.722
22.196	22.37	34.076	13.759	25.111	33.344	29.271	26.416			19.003	12.549
14.78	9.753	28.257	6.176	25.576		30.351	20.166			15.056	17.91
24.414	16.796	23.541	16.031	17.842		16.168	10.008			27.013	30.625
20.623	15.277	20.095	8.356	39.078		29.452	9.625			27.546	12.316
29.639	11.152	25.984	32.618	28.183		32.33	14.911			23.93	24.424
32.978	19.882	14.096	13.484	23.274		24.977	17.489			21.569	17.241
18.914	16.008	34.67	26.745	31.095		28.242	22.989			28.198	14.201
25.093	18.966	19.368	26.76	39.74		18.208	8.232			28.909	17.661
20.171	13.658	32.575	10.751	41.438		36.329	14.059			17.918	17.792
19.574	26.834	14.188	22.798	21.741		30.066	12.857			11.058	17.137
18.052	10.287	32.734	14.029	27.428		41.454	17.35			10.662	
22.481	8.046	55.86	24.235	23.153		31.201	18.874			29.658	
15.851	30.152	16.77	15.23	24.138		27.433	14.643			19.1	
21.689	7.74	49.056	16.903	31.902		10.163	23.167			20.692	
13.927	21.819	21.577	20.096	22.096		30.791	13.776			26.335	
23.52	17.229	22.913	21.514	21.698		32.436	22.815			28.778	
15.186	22.062	14.047	13.851	38.012		28.887	10.965			16.518	
23.12	21.445	17.107	11.942	31.348		20.842	28.947			26.897	

27.007	16.667	26.308	12.803	27.493		24.494				10.429	
18.183	11.459	39.33	17.082	14.184		23.888				21.144	
21.511	14.654	20.111	23.006	34.445		24.617				20.835	
15.281	10.92	19.411	18.907	33.128		29.434				35.057	
14.958	13.339	23.356	10.319	32.092		31.714				29.124	
15.062	14.716	22.818	16.523	37.642		19.399				39.571	
33.039	10.314	13.336	13.094			34.159				23.025	
22.598	7.96	32.595	9.241			20.175				24.023	
15.962	13.543	22.41	9.319			12.709				30.164	
15.878	6.451	24.501	13.467			22.328				33.457	
18.275	12.506	14.487	17.705			22.263				25.983	
19.914	10.786	21.147	13.853			20.69				22.414	
22.365	16.459	22.283	18.397			29.553				28.642	
21.264	12.032	21.094	9.221			23.703				31.157	
24.924	18.888	32.184	16.591			13.151				19.149	
31.26	8.916	32.424	24.612			18.732				22.723	
9.988	20.69	53.453	15.889			43.964					
10.369	16.468	29.624	25.633			21.12					
12.983	19.403	22.448	15.517			18.952					
21.484	10.202	40.675	15.798			26.625					
18.337	20.692	26.073	9.729			19.628					
19.274	16.811	31.554	24.245			35.665					
9.956	10.826	18.711	28.05			32.786					
16.971	12.421	23.563	20.986			46.174					
8.903	7.381	28.069	21.474			29.67					
13.579	12.598	47.218	6.897			43.179					
30.98	17.732	27.611	9.036			32.669					
17.467	22.989	48.799	17.875			26.775					
31.565	12.463	41.528	3.998			18.372					
11.494	18.777	26.311	24.718			28.317					
8.7	16.25	24.803	14.312								
16.576	9.508	28.187	14.367								
34.755	12.03	37.334	18.086								
30.811	6.184	30.141	13.503								
29.278	14.05	24.923	17.362								
28.54	8.913	31.68	26.645								
27.005	14.022	24.106	12.306								
41.161	8.087	27.834	8.407								
28.791	11.436	27.202	15.046								
16.667	24.123	22.835	13.343								
25.543	14.281	32.698	28.54								
17.497	12.085	24.333	9.441								
19.635	9.238	42.669	15.87								
15.947	14.255	32.585	11.751								
10.911	8.964	33.034	13.003								
28.633	9.494	31.145	10.123								
37.676	14.31	20.685	11.875								
23.359	17.09	34.692	19.519								
30.616	16.589	35.781	12.512								
30.177	31.559	27.375	14.238								
24.356	21.38	36.952	18.51								
22.629	16.574	20.679	11.542								
32.444	10.92	27.461	22.208								
18.131	11.494	31.88	16.843								
22.71	13.966	22.465	13.698								
25.159	16.664	32.631	22.123								
20.431	28.024	39.093	10.92								
10.28	9.722	27.814	19.353								
25.287	12.389	22.325	14.725								
20.214	16.97	31.899	16.075								
44.036	11.644	33.707	8.046								
35.229	18.908	22.989	18.379								
38.285	14.936	26.484	18.437								
27.512	11.973	21.924	18.41								
16.094	15.612	28.326	24.211								
20.62	33.132	42.367	20.69								
26.661	13.001	26.866	17.07								
15.873	9.962	31.676	12.55								
23.335	14.13	21.284	11.238								
16.908	9.337	16.81	20.981								
12.24	24.927	24.524	19.241								
25.287	19.588		23.563								
14.796	13.44		16.217								
22.762	9.195		25.849								

10.86	13.704		21.538								
16.971	13.398		21.076								
14.687	12.273		13.793								
17.792	16.271		13.995								
12.069	13.412		12.77								
17.478	8.153		23.165								
27.856	21.055		14.924								
17.043	13.647		11.286								
13.217	17.894		12.652								
25.287	7.265		12.48								
24.639	27.228		13.352								
13.113	15.214		14.666								
21.733	13.092		13.69								
40.308	10.351		18.646								
34.967	13.464		17.239								
22.035	14.045		9.77								
47.785	11.28		24.447								
12.955	19.727		13.974								
28.637	14.131		13.789								
38.867	19.929		11.057								
14.81	10.345		16.049								
13.005	11.751		12.851								
46.928	31.375		12.609								
31.636	8.226		21.828								
10.844	14.485		18.081								
14.341	9.195		22.875								
6.973	7.297		14.731								
21.674	23.965		10.303								
15.31	18.593		15.534								
37.853	13.896		16.419								
22.739	10.265		14.671								
29.678	13.521		29.147								
18.092	20.69		14.589								
26.726	25.287		18.391								
7.351	13.026		10.734								
18.781	16.626		9.77								
11.124	13.558		19.18								
25.275	20.53		11.343								
28.993	24.99		14.348								
13.453	22.116		12.005								
20.122	16.667		11.57								
23.923	15.787		7.812								
22.628	12.619		14.681								
12.578	19.894		9.667								
12.42	19.184		15.322								
25.287	11.122		25.16								
17.443			16.882								
21.079			7.562								
21.264			16.662								
13.928			20.585								
15.534			12.062								
8.756											
15.995											
13.845											
9.207											
13.344											
13.34											
11.853											
14.947											
15.426											
10.491											
11.545											
9.091											
12.372											
12.241											
10.285											
21.482											
9.753											
11.494											
6.696											
9.571											
9.345											
21.25											
10.045											

7.471											
20.951											
26.518											
12.695											
8.572											
10.615											
15.9											
13.711											
10.774											
10.768											
9.77											
10.714											
28.735											
19.607											
7.009											
38.029											
25.372											
22.806											
21.163											
10.345											
17.289											
13.894											
14.185											
27.011											
7.679											
14.475											
28.058											
11.337											
25.39											
16.731											
15.517											
20.252											
20.115											
20.676											
9.987											
14.662											
6.851											
10.034											
21.932											
11.062											
10.166											
5.719											
12.719											
14.211											
19.631											
28.084											
17.293											
18.499											
23.74											
7.999											
16.027											
11.864											
11.831											
13.7											
13.662											
16.548											
23.363											
6.515											
8.368											
13.652											
15.359											
20.822											
21.777											
19.812											
15.781											
28.188											
9.432											
22.556											
9.258											
23.332											
26.764											
24.946											
18.949											
20.934											

13.997											
20.343											
13.975											
12.644											
20.978											
23.437											
31.676											
22.357											
19.724											
14.886											
22.112											
8.847											
22.593											
11.96											
27.988											
24.913											
15.366											
15.927											
12.069											
22.342											
12.651											
16.868											
1024											
21.618											
18.391											
29.01											
26.687											
20.094											
12.644											
26.48											
10.033											
28.622											
26.569											
24.651											
25.231											
14.04											
12.644											
25.139											
26.386											
37.387											
22.151											
14.047											
30.517											
21.629											
15.517											
22.088											
17.565											
28.853											
20.141											
18.107											
19.656											
10.345											
20.296											
19.768											
14.775											
18.519											
15.915											
15.31											
12.007											
13.594											
14.387											
8.935											
15.135											
24.377											
8.046											
11.828											
11.968											
38.754											
24.072											
26.099											
28.197											
30.765											
28.507											
20.333											

23.266											
39.331											
26.227											
21.723											
35.789											
37.931											
26.799											
31.011											
22.46											
20.817											
25.762											
21.839											
31.233											
25.62											
25.219											
32.459											
12.644											
25.076											
20.294											
35.155											
22.243											
16.662											
24.702											
19.526											
17.993											
24.061											
27.483											
25.62											
27.715											
29.529											
20.53											
18.166											
38.759											
29.931											
29.81											
24.838											
16.732											
19.574											
19.38											
36.69											
33.096											
22.414											
27.714											
17.196											
28.08											
16.815											
37.799											
34.793											
24.97											
13.589											
20.487											
24.604											
24.801											
