# Supplementary material for: Filopodia powered by class x myosin promote fusion of mammalian myoblasts
Source: eLife. 2021 Sep 14;10:e72419. doi: 10.7554/eLife.72419 (PMC8500716; doi:10.7554/eLife.72419)
Supplement: Figure 4—figure supplement 1—source data 1. [file elife-72419-fig4-figsupp1-data1.pdf]

Fig S5D- Muscle fiber diameters without tamoxifen administration																	
Uninjured TA									Sham-treated Pax7-WT TA at Day 8					Sham-treated Pax7M10cKO TA at Day 8			
Muscle #1	Muscle #2	Muscle #3	Muscle #4	Muscle #5	Muscle #6	Muscle #7	Muscle #8	Muscle #9	Muscle #1	Muscle #2	Muscle #3	Muscle #4	Muscle #5	Muscle #1	Muscle #2	Muscle #3	Muscle #4
39.007	56.413	37.527	69.134	52.267	36.594	28.195	41.926	55.172	23.61	33.907	45.591	22.684	61.313	20.003	28.66	76.761	9.794
29.83	56.625	46.32	47.607	66.203	31.569	67.053	53.668	62.779	17.954	25.52	35.952	16.09	35.09	47.694	20.592	39.075	49.762
57.019	12.195	52.397	56.887	27.726	57.319	54.597	51.006	51.411	12.892	67.674	19.159	36.124	9.512	14.063	39.595	46.849	29.083
44.783	53.73	15.325	73.743	20.513	43.747	54.469	32.818	51.254	18.342	32.184	33.465	25.242	42.571	10.156	16.28	45.508	45.26
30.936	55.125	28.316	62.934	66.978	50.808	67.835	46.063	64.114	18.454	34.754	30.599	50.913	52.89	29.185	33.458	40.2	50.798
38.25	56.655	35.923	80.013	39.592	58.867	46.567	51.057	40.835	14.079	32.809	29.766	19.224	45.044	30.269	17.624	15.72	53.448
39.08	49.553	28.025	76.773	52.299	34.646	60.49	34.722	47.885	30.885	39.427	23.2	16.904	38.646	16.506	34.842	44.801	40.805
45.515	47.024	39.536	49.679	24.871	45.834	49.807	39.86	66.162	20.676	30.227	27.16	14.702	45.335	13.237	47.996	43.819	46.519
49.314	56.23	36.887	65.11	42.381	53.229	78.02	32.758	56.139	24.343	41.308	28.211	35.847	39.692	16.908	27.458	70.433	50.388
32.93	62.953	10.448	71.714	39.691	51.345	38.601	45.14	54.462	14.021	49.118	32.883	23.828	47.776	27.128	40.478	26.117	18.638
61.149	54.147	41.389	62.612	57.116	54.347	51.257	47.673	57.63	12.069	25.54	35.548	38.751	15.846	15.024	20.883	39.411	33.153
40.565	57.018	55.973	77.79	65.174	69.983	39.142	48.499	65.479	15.809	24.372	31.604	30.349	13.235	36.174	21.891	29.014	23.938
30.026	45.885	30.744	54.015	62.712	66.352	42.67	44.729	44.788	14.499	28.445	19.282	34.067	39.387	19.54	29.885	43.28	62.847
48.713	44.332	31.756	50.2	61.712	71.344	49.569	29.081	51.637	25.493	20.875	33.813	51.726	28.493	32.128	19.724	17.441	19.638
45.66	59.65	26.13	14.152	45.164	17.761	66.805	20.697	56.742	49.336	19.726	36.533	44.115	38.03	25.236	27.586	41.954	38.626
30.796	51.045	44.259	66.535	53.143	34.431	46.355	34.858	41.833	10.87	34.708	32.759	19.34	24.988	9.796	42.498	10.343	34.104
38.046	66.063	56.592	66.687	33.559	54.985	38.564	63.218	50.426	24.383	35.418	28.479	13.118	46.124	9.275	13.41	28.642	30.002
32.061	37.742	38.251	40.264	40.319	32.427	56.301	34.375	55.545	66.913	31.014	27.718	57.618	38.903	15.426	15.956	17.379	37.429
30.849	59.536	27.256	63.571	80.288	63.131	75.295	39.557	43.032	18.11	39.692	42.523	50.104	59.111	16.308	16.821	36.302	43.058
35.021	48.362	34.442	57.269	42.763	56.56	67.822	33.357	29.958	13.218	40.23	25.095	55.231	17.688	35.575	34.308	39.655	17.547
36.197	57.136	21.375	61.85	54.548	57.864	41.15	27.438	71.901	18.688	49.095	26.121	14.68	47.5	16.582	43.653	64.079	39.586
33.295	61.619	51.665	68.358	54.441	44.44	74.434	48.131	63.463	52.94	18.518	27.347	41.901	57.408	34.756	18.966	22.414	36.706
34.963	56.771	31.304	73.18	31.672	62.8	46.325	42.086	64.82	20.204	22.648	17.816	52.179	10.131	32.44	41.772	36.142	59.77
56.311	34.786	51.997	53.643	33.865	41.845	54.41	43.548	60.603	28.057	34.735	24.027	28.04	59.195	19.08	12.522	56.417	39.976
32.846	51.403	34.885	44.716	57.474	48.967	35.306	45.933	72.838	22.757	41.151	20.69	39.552	19.637	36.612	33.575	31.795	28.678
34.303	59.938	43.269	32.58	43.41	63.034	48.363	61.394	47.326	24.833	28.256	30.388	26.319	43.491	37.344	19.504	35.866	30.041
45.212	53.405	36.825	62.278	58.448	59.856	42.949	40.003	64.357	14.291	32.533	39.795	49.447	43.667	13.491	34.924	46.961	28.153
35.452	57.898	35.496	13.921	60.47	42.594	47.763	52.768	45.665	13.106	31.182	33.831	37.186	36.421	34.68	26.727	49.841	44.182
63.726	37.163	42.94	68.286	37.356	32.789	51.878	29.971	62.454	29.029	39.645	27.701	53.513	34.571	15.819	34.853	33.996	29.426
48.969	52.357	38.574	63.005	34.091	63.028	54.51	48.768	54.624	20.825	41.265	36.012	39.678	29.885	13.878	15.981	19.032	35.273
37.143	57.933	51.669	63.488	52.597	60.814	40.633	58.992	43.103	18.394	12.79	38.645	41.837	24.633	17.576	13.95	16.357	27.878
31.446	45.756	29.479	40.827	37.499	33.685	51.014	40.571	52.081	18.622	33.93	40.785	34.924	42.806	32.704	33.438	45.187	39.013
41.042	33.352	60.256	48.601	34.096	53.04	71.389	46.182	46.793	13.793	28.989	20.008	22.622	31.861	12.125	27.881	74.283	40.727
55.793	53.228	60.553	49.696	68.23	52.139	44.338	58.274	42.088	40.614	26.848	25.492	21.083	21.272	25.097	11.739	28.651	47.426
36.081	43.696	15.784	42.811	50.936	31.218	43.578	31.141	56.814	16.378	13.796	30.384	18.738	19.778	28.173	44.239	23.343	44.003
25.602	48.979	25.129	10.308	39.366	58.046	71.199	34.826	67.043	12.509	26.437	17.875	40.67	39.639	40.609	33.16	37.807	31.494
35.632	47.625	46.567	70.091	20.115	43.193	34.171	42.474	40.647	16.899	37.295	36.907	50.003	15.746	28.181	33.363	15.512	46.723
23.856	29.586	31.698	63.952	50.79	62.246	43.018	38.785	53.089	15.491	18.797	22.491	49.015	35.632	15.961	24.533	38.889	39.29
14.029	52.599	29.373	50.937	53.678	53.586	36.133	40.23	51.719	15.903	36.167	14.429	28.736	53.996	15.763	21.887	70.951	49.666
33.973	64.824	33.599	54.519	30.762	36.75	34.316	45.422	66.22	44.02	18.966	26.111	25.862	37.77	33.024	14.511	42.896	34.03
31.313	32.759	40.687	53.959	44.641	34.632	45.228	30.141	68.768	13.452	42.313	21.236	23.268	26.372	17.083	38.552	41.945	54.679
31.636	53.307	44.824	52.521	45.401	60.668	28.12	40.858	35.015	21.142	28.422	23.563	44.906	25.862	40.499	14.652	82.122	11.149
39.276	63.652	40.152	41.958	31.24	50.17	50.335	54.268	47.979	32.245	38.506	24.084	37.319	32.643	26.81	18.488	37.888	29.492
56.35	56.429	52.97	71.902	31.363	55.653	58.45	49.704	56.136	22.4	30.18	35.685	26.447	14.942	22.525	33.383	31.001	42.33
49.368	54.26	34.839	35.493	40.418	46.357	38.265	28.552	51.854	20.51	24.126	19.825	36.251	28.586	18.336	19.54	26.484	43.014
47.028	40.519	28.909	64.343	26.368	47.951	44.533	34.768	65.884	28.585	33.759	31.698	15.391	26.773	15.697	15.028	21.839	52.18
28.288	46.473	23.27	50.238	53.806	33.843	47.618	46.552	57.147	22.296	23.339	12.378	43.446	35.515	37.1	17.853	34.733	32.027
43.027	66.92	37.413	54.889	34.638	40.973	48.678	53.003	52.465	14.763	22.154	16.042	46.243	18.391	27.011	39.047	34.106	36.14
26.176	51.767	42.827	56.641	47.077	58.317	35.532	44.413	42.436	18.694	50.374	35.989	14.385	37.498	37.002	37.711	39.003	35.856

31.357	54.24	64.324	52.589	56.897	47.624	51.755	49.253	46.307	27.606	16.053	20.997	43.699	46.087	16.255	15.084	68.237	68.941
52.626	58.914	51.57	54.801	21.723	53.11	58.289	58.391	34.368	25.686	18.982	17.519	34.98	37.411	29.262	50.945	37.58	33.71
27.147	55.859	34.345	36.021	26.437	39.523	54.848	29.177	47.018	13.522	25.283	25.91	37.931	18.676	22.001	24.582	55.815	44.484
42.089	40.898	13.826	47.599	36.782	57.176	44.821	79.711	68.819	23.301	24.991	27.662	35.974	53.519	14.404	25.967	66.74	56.174
39.386	41.021	50.579	70.772	51.382	57.625	37.3	35.475	58.394	23.394	24.246	25.08	31.317	34.227	12.19	13.793	27.353	26.853
41.804	53.054	58.355	56.905	28.657	37.477	42.534	37.209	61.516	22.185	36.782	29.609	40.732	38.551	8.531	57.387	17.38	28.798
38.831	56.253	34.947	69.465	34.745	47.419	50.789	55.39	36.919	23.377	25.38	36.472	18.936	28.806	27.563	11.823	29.459	42.545
37.062	68.105	29.174	42.529	60.36	60.918	43.325	29.807	71.693	20.506	36.614	20.582	42.224	40.281	25.298	66.894	36.651	27.502
39.839	58.229	39.337	64.487	29.31	54.075	44.514	28.963	54.608	27.361	19.958	36.899	34.634	22.616	31.509	25.848	34.442	43.542
43.891	52.489	36.864	29.672	60.224	54.309	46.875	44.243	53.184	23.682	37.419	19.62	22.797	29.443	29.28	17.92	30.354	35.057
28.205	52.41	43.125	48.905	52.407	75.371	66.738	39.72	47.126	18.93	32.178	24.619	33.954	39.617	17.749	18.686	32.176	25.561
46.784	60.448	12.259	39.766	28.719	37.122	56.029	36.041	65.664	33.249	18.715	45.439	20.571	24.6	23.683	11.942	22.775	29.829
50.422	52.82	33.123	36.991	62.203	36.128	40.548	49.882	41.022	13.729	47.814	22.194	42.656	21.631	36.955	20.648	23.329	40.443
38.447	56.472	56.511	59.039	42.565	47.612	38.262	48.74	60.204	21.906	42.315	37.874	39.08	42.16	27.085	29.087	46.474	
37.381	38.422	15.969	25.071	38.396	50.371	59.822	39.298	60.963	17.174	13.087	34.781	22.423	36.348	36.675	31.422	29.885	
39.071	23.053	41.374	47.88	53.697	65.434	39.743	48.002	41.299	11.288	38.525	39.816	21.077	20.604	32.943	27.737	45.752	
37.96	59.742	38.035	36.294	28.538	70.828	57.139	40.963	41.172	12.349	43.926	26.456	20.467	20.534	24.384	14.497	31.507	
45.507	37.367	47.261	36.09	33.423	53.464	42.576	37.596	38.102	19.945	33.552	39.877	24.785	39.421	23.563	11.295	22.351	
24.713	56.248	36.532	42.174	48.993	58.348	32.383	50.388	59.028	44.844	60.782	11.738	13.828	31.546	21.426	34.839	13.92	
38.901	56.828	21.544	46.176	27.032	53.399	49.172	41.006	70.184	41.651	27.586	24.898	24.739	30.954	33.607	13.273	39.034	
44.724	43.985	49.869	53.541	26.285	45.776	49.671	45.737	53.04	28.357	25.069	10.778	18.715	30.479	20.871	35.781		
36.888	38.956	16.941	14.602	25.622	46.845	48.767	39.714	60.756	11.313	25.244	31.955	16.667	21.335	44.574	13.7		
28.016	48.002	46.572	53.148	48.483	50.236	40.182	28.283	51.665	18.435	28.733	32.677	16.753	34.345	36.82	43.593		
49.98	54.893	47.943	63.128	38.569	42.354	58.758	42.727	55.169	12.934	21.884	29.727	40.189	25.078	42.45	14.934		
37.888	54.699	42.481	46.164	32.194	55.602	34.832	29.666	58.825	20.676	21.825	25.093	16.495	34.208	33.945	55.937		
49.291	50.411	45.087	58.547	32.895	40.313	37.145	35.643	54.971	32.254	42.461	33.603	18.346	14.369	19.303	14.378		
47.969	56.812	28.07	40.716	33.24	33.069	47.994	42.955	64.832	43.283	30.694	22.328	22.444	43.406	30.026	18.054		
30.556	58.224	31.787	37.978	65.474	47.155	40.673	31.581	56.108	17.514	37.671	25.897	14.394	30.72	25.078	13.793		
35.574	52.836	47.891	56.998	33.667	44.006	44.465	44.305	44.828	32.983	41.388	25.574	25.608	27.011	36.178	13.595		
11.839	46.61	40.177	49.131	32.934	33.404	53.27	31.345	44.102	29.275	25.134	32.207	25.069	39.903	30.221	41.741		
30.02	51.197	41.909	52.778	37.786	33.032	54.023	45.666	50.851	32.434	36.138	30.608	18.908	35.354	21.21	17.461		
43.068	49.931	28.792	36.172	57.777	36.46	65.504	35.949	41.736	26.178	36.445	23.419	21.938	36.19	26.979	49.232		
27.27	54.787	16.968	12.854	37.512	44.862	65.453	53.081	45.665	19.469	25.29	32.184	26.713	41.014	27.311	29.481		
33.131	42.003	41.952	53.131	30.172	28.884	9.534	34	60.41	21.649	22.328	40.665	18.908	36.732	47.496	15.114		
29.226	14.985	54.167	50.746	51.602	41.825	32.301	41.892	55.222	27.961	16.327	26.837		29.666	39.316	21.361		
38.021	54.308	41.363	41.166	28.659	49.899	56.164	31.774	47.004	29.295	45.937	25.597		17.479	13.207	41.761		
6.679	59.098	61.175	62.177	31.469	49.513	37.793	54.966	44.281	35.785	39.583	38.783		14.016	16.543	33.055		
61.982	50.125	38.123	47.825	38.018	23.155	29.838	52.181	35.232	40.101	21.756	33.757		12.285	16.646	33.932		
69.172	46.084	32.776	61.187	30.968	40.15	52.742	35.729	58.541	19.963	28.443	21.706		30.593	23.305	21.49		
45.78	39.342	38.87	14.771	29.566	34.617	33.823	48.359	46.552	49.657	28.529	19.559		30.076	25.899	33.244		
42.36	31.204	41.454	43.889	27.349	27.724	45.208	43.889	43.715	25.299	21.599	40.23		41.736	16.595	29.33		
56.019	19.616	38.131	42.843	25.944	38.757	59.749	40.181	40.311	24.679	27.198	31.608		32.217	20.209	14.997		
51.029	54.554	6.919	38.785	58.639	43.47	64.565	28.681	63.348	31.142	43.06	30.605		42.56	42.989	18.247		
64.421	48.135	13.076	33.033	22.438	35.04	52.795	48.603	61.975	36.02	43.247	26.623		33.778	17.211	18.242		
49.002	45.053	42.208	35.212	27.524	31.803	66.177	36.866	48.067	28.248	34.225	35.437		34.751	42.437	14.918		
51.149	52.538	53.639	37.931	15.645	27.1	60.899	27.704	42.025	35.598	26.654	36.752		36.207	22.887	19.788		
33.295	58.305	29.901	62.766	35.107	42.983	54.865	44.009	58.225	20.115	31.265	27.351		34.115	17.518	14.44		
34.904	49.994	58.796	53.713	58.762	39.016	55.286	40.951	40.301	27.011	34.994	19.613		27.669	32.054	28.234		
34.762	33.603	68.628	49.452	33.033	19.929	23.214	40.435	41.789	43.281	24.477	30.809		29.781	24.713	18.972		
17.292	54.982	49.327	12.77	53.329	30.828	64.099	37.652	46.863	18.654	18.199	34.136		29.247	16.548	15.229		
25.958	49.079	44.522	69.408	34.205	33.038	55.498	48.637	42.642	33.027	28.327	31.609		18.254	45.197	14.799		
38.885	52.01	12.372	45.517	51.346	46.096	29.074	42.019	56.508	23.206	23.963	35.632		32.23	20.226	12.84		
43.516	36.846	39.539	47.849	45.047	62.421	52.035	47.294	48.122	34.354	33.778	17.389		32.824	21.352	18.604		

52.837	35.561	49.664	44.041	50.681	31.802	35.345	35.388	44.081	32.337	30.369	22.414		22.724	25.063	34.745		
37.928	33.14	21.844	61.09	36.328	47.701	58.023	41.727	42.241	50.572	18.767	32.533		44.102	13.413	14.368		
39.275	37.134	35.145	61.867	26.585	27.816	24.65	33.14	56.194	31.034	29.048	9.969		39.978	22.362	47.285		
33.025	35.61	29.912	26.122	43.346	25.499	41.566	44.549	38.865	16.827	16.753	27.516		33.741	29.092	29.205		
	52.428	40.208	38.043	26.548	44.246	64.919	35.542	57.967	54.022		25.667		25.744	19.506	17.521		
	60.258	29.523	41.992	44.317	64.065	23.991	37.659	57.259	31.723		31.489		37.011	25.906	20.012		
	39.971	18.434	45.265	51.472	37.778	62.571	31.489	48.447	38.416		27.805		31.783	29.898	33.322		
	48.07	31.205	54.811	55.606	35.241	49.588	28.9	60.222	37.752		33.703		29.295	17.442	27.327		
	41.486	21.261	41.219	49.754	38.246	59.056	44.793	44.747	27.558		22.989		37.363	16.283	32.2		
	53.782	50.665	53.592	55.047	53.199	46.249	50.63	38.865	16.825		30.415		34.949	16.89	38.506		
	53.652	51.683	29.281	29.162	48.365	55.191	50.757	36.186	30.471		23.887		48.851	27.842	22.806		
	34.446	58.401	73.668	30.084	45.008	46.46	48.85	49.966	38.56		33.732		42.581	21.518	52.441		
	44.119	47.701	45.294	43.326	38.49	46.237	42.415	61.073	45.671		33.333		34.897	12.504	12.366		
	54.163	36.323	32.104	36.023	25.489	49.434	49.611	53.223	25.862		54.598		32.697	12.926	40.446		
	42.651	65.278	21.584	30.74	31.967	62.58	39.573	46.518	32.123		16.117		31.213	20.606	30.097		
	14.981	26.279	44.631	25.526	20.831	46.942	48.579	49.177	33.564		33.526		25.518	27.598	31.861		
	39.831	29.895	54.798	46.643	49.985	26.278	37.807	67.44	32.413		23.563		29.659	15.959	22.528		
	32.616	52.421	57.589	41.862	36.68	57.051	46.285	61.427	38.797		29.71		40.211	16.975	18.318		
	31.311	89.471	19.34	45.742	26.777	24.897	65.503	45.621	38.111		21.45		31.469	25.493	33.515		
	44.579	51.308	34.96	43.388	29.002	65.139	51.096	38.279	30.46		24.77		28.762	25.208	15.195		
	35.108	31.351	45.39	52.645	44.066	47.492	45.433	54.036	19.13		24.329		24.427	47.109	9.01		
	54.253	37.507	34.483	58.718	26.119	31.488	54.267	42.79	18.66		24.024		26.997	14.686	14.535		
	51.309	85.825	41.264	58.142	32.134	26.134	32.003	60.725	28.617		22.126		25.923	32.158	17.9		
	30.208	54.16	55.958	53.786	56.248	54.159	54.441	56.05	27.411		13.529		28.355	17.945	31.886		
	57.296	30.636	59.506	53.493	35.153	41.278	66.028	34.837	48.614		26.029		27.789	19.277	27.994		
	52.659	34.724	37.423	36.325	29.685	47.393	38.901	53.161	20.239		20.456		37.087	18.443	29.885		
	26.992	45.741	37.848	54.713	37.077	30.686	26.487	65.795	27.254		19.146		30.81	26.222	30.702		
	49.755	39.545	43.17	30.895	48.257	51.402	37.954	56.672	20.966		19.371		49.135	13.846	31.609		
	55.685	33.957	56.416	59.396	33.99	50.733	39.429	52.609	52.859		14.63		22.845	25.857	20.754		
	31.785	50.27	46.989	55.236	44.647	54.822	35.752	55.559	34.262				38.373	28.306	15.81		
	42.79	28.902	51.191	35.18	40.088	49.273	50.237	47.613	52.537				30.23	26.026	33.44		
	47.122	61.013	59.582	36.514	46.631	43.286	60.893	38.029	38.822				38.444	26.351	21.221		
	39.17	42.326	58.219	29.276	38.721	17.152	28.313	34.739	25.019				35.149	22.948	29.759		
	32.007	21.59	9.166	50.14	31.775	40.237	41.683	56.821	25.196				39.125	22.67	17.508		
	25.648	34.472	39.327	41.493	33.333	36.275	57.263	50.902	32.334				36.242	17.35	17.18		
	35.095	40.536	46.977	20.104	19.383		55.724	57.424	36.648				42.982	27.721	10.946		
	42.003	37.443	52.47	53.923	34.798		57.388	37.638					26.368	29.89	44.704		
	29.277	23.755	60.322	34.158	29.024		54.338	59.195					42.905	19.039	16.522		
	58.483	34.867	64.633	28.09	40.107		57.536	41.51					36.292	13.947	21.066		
	56.835	28.996	35.184	47.133	36.782		29.395	36.469					39.617	26.752	30.637		
	49.131	21.813	39.902	33.555	44.685		59.53	52.999					39.786	21.977	11.471		
	47.648	36.343	10.134	19.407	38.923		56.841	39.693					31.024	32.728	17.503		
	70.346	39.665	41.379	32.791	31.016		53.874	60.178					13.473	30.468	31.609		
	31.384	23.229	58.577	22.245	37.002		44.509	64.715					14.163	47.733	14.659		
	46.869	40.043	64.916	25.287	41.1		56.829	41.839					30.327	18.805	36.47		
	48.61	29.731	57.466	49.362	40.26		54.857	35.036					32.54	25.676	30.581		
	40.42	42.67	42.024	49.741	46.623		37.726	56.741					35.655	14.63	24.635		
	19.351	33.195	41.33	52.749	62.099		51.034	38.109					28.606	34.901	21.171		
	57.993	28.798	51.584	27.83	26.516		53.077	41.961					31.108	34.396	17.856		
	40.028	42.904	59.681	25.046	44.284		52.599	49.638					32.426	22	17.285		
	28.926	27.867	63.991	50.075	43.103		37.627	48.637					25.862	22.846	23.796		
	30.801	33.434	54.696	51.008	35.347		28.586	47.542					22.256	13.243	16.963		
	44.364	42.347	42.268	54.2	32.393		59.453	53.907					38.328	20.225	14.811		

	40.007	17.114	36.768	37.876	33.623		58.06	44.399					10.626	16.406	42.901		
	52.998	42.002	62.433	26.314	30.563		51.322	49.547					10.328	31.965	25.862		
	53.03	30.596	54.362	31.822	43.649		49.705	61.752					29.034	27.448	13.932		
	60.258	43.273	33.394	43.525	31.34		36.328	41.868						34.127			
	41.293	32.702	36.056	44.284	22.924		45.303	58.238						30.331			
	31.324	43.928	41.63	37.241	53.175		59.073	51.713						23.645			
	64.067			38.927	30.473		61.469	52.686						28.625			
	41.01			45.696	39.335		38.506	36.453						36.746			
	29.385			55.568	40.522		30.46	45.336						39.08			
	43.848			42.726	38.575		34.326	43.448						38.021			
	11.145			39.7	30.005		43.783	65.17						25.979			
	47.397			31.419	37.27		48.371	59.541						17.735			
	27.534			50.883	34.609		31.885	43.997						30.867			
	46.158			53.799	12.343		27.396	61.994						52.736			
	51.724			31.77	35.106		38.32	55.552						17.671			
	40.805			28.676	39.617		53.799	39.289						32.02			
	63.14			30.867	51.51		57.625	54.362						28.667			
	34.621			32.745	30.592		35.017	30.084						33.317			
	38.424			30.846	35.702		53.202	37.187						44.754			
	55.391			27.848	35.941		38.983	35.732						26.487			
	39.12			52.364	26.083		59.686	35.93						20.519			
	38.223			23.175	33.335		53.48							25.672			
	61.975			21.348	42.319		53.371							19.54			
	32.917			27.43	46.324		40.633							15.682			
	31.034			42.676	30.851		52.017							39.387			
	44.22			61.319	45.628		30.694							23.261			
	58.332			37.931	37.9		50.19							34.804			
	28.545			56.547	31.689		42.848							31.37			
	36.266			25.006	41.379		47.12							29.709			
	34.989			51.327	12.673		26.938							23.977			
	60.302			29.269	28.161		33.407							24.814			
	53.575			45.775	45.977		48.576							48.037			
	32.963			40.229	35.475		42.126							30.885			
	46.171			45.785			42.891							41.236			
	41.199			60.112			41.435							28.736			
	33.656			43.15			40.893							14.503			
	52.814			35.761			48.026							27.098			
	64.589			27.539			47.165							13.42			
	21.565			35.141			58.807							36.246			
	34.208			42.513			30.583							28.983			
	59.478			50.594			50.379							17.216			
	32.212			50.801			33.813							24.542			
	32.212			24.427			37.373							41.935			
	32.212			25.647			40.307							27.205			
	32.212			25.899			45.694							21.711			
	32.212			27.747			33.686							25.1			
	32.212			36.525			34.255							32.999			
				41.883			44.233							27.034			
				31.033			33.85							64.642			
				39			27.896							26.667			
				20.101			46.101							12.344			
				44.272			64.829							28.971			
							49.386							19.727			

							45.839							19.763			
							48.6							36.108			
							38.678							15.615			
							33.926							27.586			
														17.863			
														27.852			
														25.474			
														19.279			
														37.45			
														27.876			
														19.086			
														55.233			
														18.129			
														21.813			
														16.36			
														16.049			
