# Supplementary material for: Filopodia powered by class x myosin promote fusion of mammalian myoblasts
Source: eLife. 2021 Sep 14;10:e72419. doi: 10.7554/eLife.72419 (PMC8500716; doi:10.7554/eLife.72419)
Supplement: Figure 4—figure supplement 1—source data 3. [file elife-72419-fig4-figsupp1-data3.pdf]

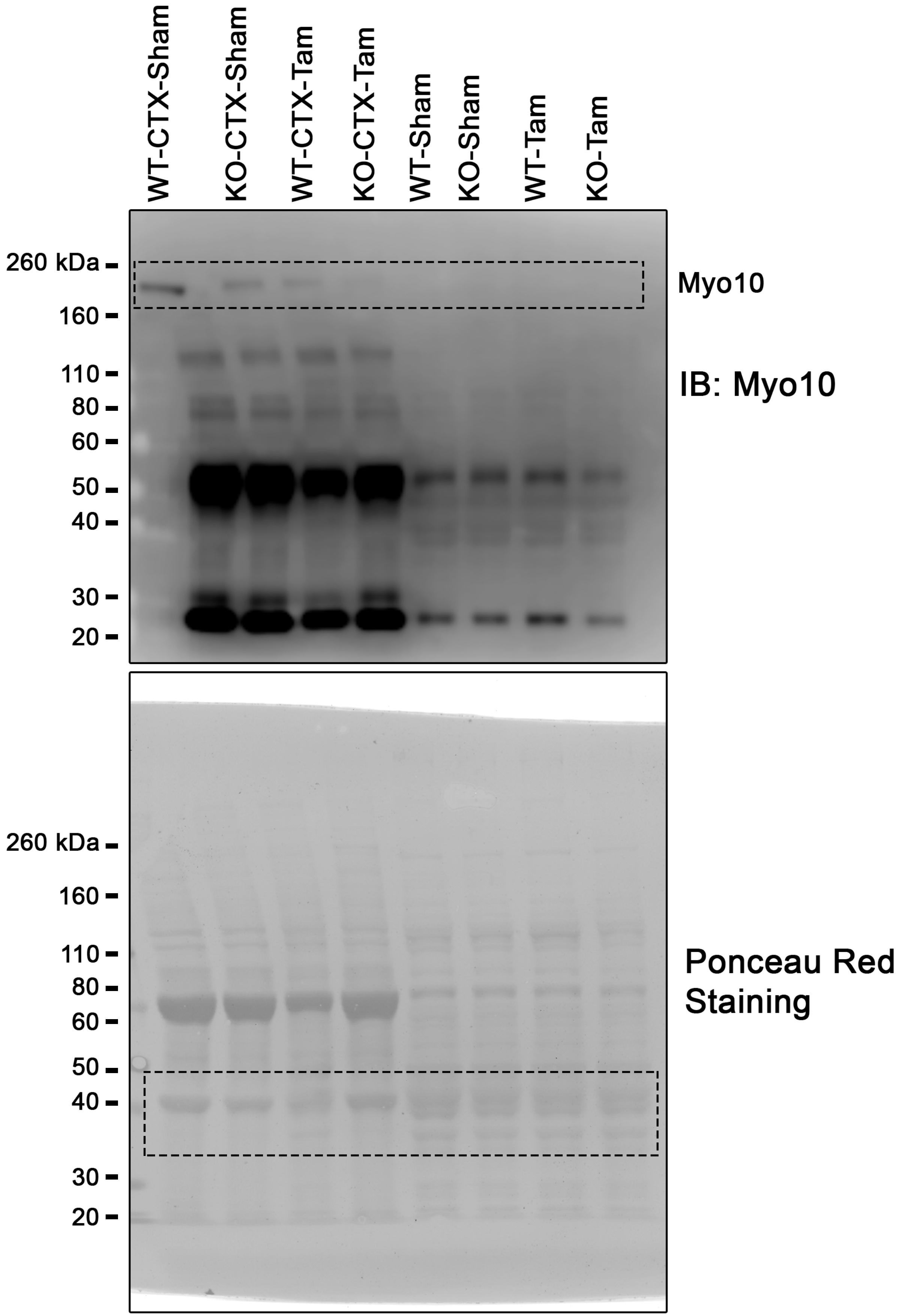
WT-CTX-Sham
KO-CTX-Sham
WT-CTX-Tam
KO-CTX-Tam
WT-Sham
KO-Sham
WT-Tam
KO-Tam
260 kDa
160
110
80
60
50
40
30
20
Myo10
IB: Myo10
260 kDa
160
110
80
60
50
40
30
20
Ponceau Red Staining
