# Supplementary material for: Filopodia powered by class x myosin promote fusion of mammalian myoblasts
Source: eLife. 2021 Sep 14;10:e72419. doi: 10.7554/eLife.72419 (PMC8500716; doi:10.7554/eLife.72419)
Supplement: Figure 4—figure supplement 2—source data 1. [file elife-72419-fig4-figsupp2-data1.pdf]

Fig S6B- Muscle mass recovery from freeze injury		
**Muscle Sample#**	**Pax7-WT**	**Pax7-M10cKO**
1	100.1745201	55.74162679
2	93.22344322	67.04545455
3	91.66666667	70.72649573
4	80.81632653	84.01559454
5	74.53798768	57.84543326
6		85.77586207
7		62.31578947
8		69.96904025
