# Supplementary material for: Filopodia powered by class x myosin promote fusion of mammalian myoblasts
Source: eLife. 2021 Sep 14;10:e72419. doi: 10.7554/eLife.72419 (PMC8500716; doi:10.7554/eLife.72419)
Supplement: Figure 4—figure supplement 2—source data 2. [file elife-72419-fig4-figsupp2-data2.pdf]

Fig S6C- Muscle fiber diameter																									
Pax7-WT																									
Muscle #1				Muscle #2				Muscle #3				Muscle #4				Muscle #5				Muscle #1				Musc	
10.661	39.837	33.495	11.224	46.213	25.056	31.464	25.132	18.882	43.466	58.335	27.778	28.745	62.364	72.433	34.811	58.04	48.513	42.38	51.039	10.661	63.539	43.339	36.738	49.854	44.86
35.714	102.549	52.263	17.196	49.122	23.848	32.29	20.949	43.534	14.973	33.069	18.707	39.131	35.056	85.665	37.037	60.272	67.697	44.896	37.651	35.714	87.319	22.432	63.714	23.992	17.948
18.997	97.123	55.413	82.771	23.281	48.132	60.798	14.212	17.196	19.425	56.078	27.764	10.045	60.684	92.509	31.46	53.868	66.037	46.232	36.159	18.997	35.873	23.058	53.215	23.859	21.84
38.36	34.936	62.589	40.601	88.136	11.905	19.841	18.519	25.132	14.55	42.438	59.411	15.329	43.025	17.151	51.258	34.574	33.069	42.754	46.13	38.36	13.434	52.91	34.714	11.789	42.981
15.402	40.441	69.731	21.164	31.801	46.607	35.714	37.67	22.189	48.173	44.896	28.236	17.459	67.986	50.785	61.646	21.564	11.81	42.064	47.311	15.402	28.159	7.067	36.692	20.957	38.36
22.487	28.278	26.177	29.272	28.807	18.214	16.613	23.81	31.746	34.392	46.296	14.031	38.191	78.01	14.55	24.705	20.427	10.06	60.09	58.072	22.487	12.982	18.022	39.586	26.124	20.845
13.095	71.649	78.298	103.216	21.448	15.873	17.328	16.482	24.212	32.361	76.072	43.651	29.845	23.878	11.905	42.005	18.47	20.088	9.693	41.887	13.095	23.175	53.25	17.617	35.238	14.205
8.418	38.244	79.262	41.913	20.055	12.973	36.68	15.616	27.778	33.909	10.663	45.418	80.987	61.701	63.055	51.755	36.114	57.594	16.107	33.084	8.418	15.722	48.044	37.03	93.508	13.779
12.842	25.791	61.546	64.046	19.222	37.44	35.542	38.36	49.283	85.981	17.028	36.183	37.609	70.149	54.431	52.801	35.62	43.935	42.327	51.587	12.842	37.878	19.665	39.309	14.69	21.733
25.254	56.094	72.751	87.439	45.397	22.035	21.433	20.957	28.06	25.132	38.28	7.393	62.916	48.597	26.455	26.292	91.226	80.688	31.801	69.155	25.254	9.865	19.841	22.386	34.241	59.77
18.588	50.076	55.525	34.979	47.418	43.738	23.408	34.045	53.192	40.026	54.233	24.143	53.618	17.76	33.069	39.683	18.603	48.942	47.988	29.101	18.588	14.55	22.487	23.742	29.973	33.693
10.582	37.888	72.287	26.189	24.432	16.743	26.892	57.673	30.959	47.619	65.11	36.568	33.638	30.278	60.796	64.092	43.188	48.942	44.896	58.389	10.582	33.468	23.81	49.556	23.99	23.81
34.527	92.55	36.309	31.801	32.285	39.683	27.699	18.707	34.588	66.437	42.626	19.707	29.93	58.595	75.182	18.068	13.015	14.643	36.478	14.681	34.527	10.601	17.196	36.678	19.841	35.761
19.532	27.124	14.681	68.968	10.884	29.101	10.874	26.461	40.845	24.859	42.532	37.063	28.933	51.587	26.809	91.207	31.125	32.599	50.265	33.069	19.532	23.837	32.123	14.505	29.101	14.965
32.909	37.037	77.4	14.785	25.132	14.031	24.318	19.154	39.399	35.126	45.841	30.423	38.569	59.159	58.988	37.888	56.274	54.206	72.205	55.556	32.909	11.241	12.835	72.022	31.336	28.803
37.764	46.982	48.152	51.896	45.006	9.259	27.778	26.843	32.012	34.788	16.755	64.886	21.164	38.83	52.585	54.233	13.393	52.428	25.017	44.974	37.764	11.224	19.581	51.529	62.767	15.901
31.956	36.725	63.98	72.156	33.337	14.55	17.168	27.593	19.185	10.215	46.751	20.389	43.724	32.197	47.499	66.138	32.402	19.16	46.296	16.368	31.956	14.55	37.54	26.627	17.196	37.037
15.382	56.399	73.494	28.859	41.005	14.409	13.099	18.508	60.577	49.08	30.423	29.145	34.33	81.497	33.069	77.982	49.046	18.519	66.196	28.443	15.382	15.873	29.759	40.228	18.707	28.06
10.067	47.451	40.076	61.222	37.008	13.949	9.585	23.455	30.197	20.731	36.103	28.06	35.736	78.004	53.571	56.242	8.372	63.249	45.629	39.683	10.067	30.428	19.572	27.02	9.259	66.489
24.586	13.018	19.01	13.402	27.766	32.083	8.795	67.018	17.447	23.276	62.886	39.442	70.847	30.423	55.392	49.93	28.995	60.83	71.792	43.55	24.586	17.202	13.228	37.314	40.412	38.88
23.298	76.638	27.81	42.242	31.408	50.598	17.204	33.717	37.607	27.232	25.777	27.768	23.363	79.862	89.393	85.48	20.95	34.878	21.914	19.946	29.101	23.416	15.673	36.705	10.497	24.683
29.101	49.958	24.888	23.333	25.194	8.532	24.219	30.423	30.701	38.037	35.367	20.632	53.551	25.254	43.693	14.03	37.024	11.224	12.457	10.111	13.704	46.296	10.289	19.841	47.367	53.947
13.704	43.222	43.304	70.665	48.094	40.529	74.408	40.687	47.349	19.022	39.461	26.455	31.135	49.237	40.754	63.583	17.017	18.519	45.468	25.132	41.005	18.431	46.144	24.886	23.81	59.369
41.005	66.203	42.328	64.297	20.351	21.316	51.335	28.995	15.202	33.069	49.958	30.622	69.876	109.878	100.619	71.408	24.958	51.587	51.364	17.892	15.733	23.075	27.678	50.475	17.771	56.592
15.733	59.341	61.961	15.427	36.618	40.219	18.519	20.509	33.672	26.676	56.598	70.709	53.566	58.743	19.581	55.861	36.966	25.132	35.939	40.622	40.058	20.871	22.487	19.841	41.639	46.237
40.058	30.107	12.159	77.663	37.294	22.233	21.028	30.018	31.746	51.486	38.334	36.049	36.548	47.619	59.825	22.487	14.03	27.778	48.96	27.778	47.491	30.782	28.36	33.047	20.257	66.69
47.491	37.886	29.288	86.633	53.202	34.186	22.233	27.506	17.338	52.551	37.931	37.27	87.306	103.507	35.714	26.056	15.873	64.475	64.487	44.076	19.025	53.786	16.862	17.022	10.654	79.461
19.025	77.426	46.122	56.516	37.189	39.173	58.33	32.299	23.446	41.005	14.091	23.81	33.508	73.011	52.351	54.003	21.412	26.068	14.55	78.042	20.922	11.905	23.025	48.525	34.769	17.174
20.922	44.94	62.938	62.369	45.33	11.905	11.905	15.402	42.328	18.603	44.918	35.482	52.9	44.831	59.764	29.196	35.116	24.843	63.779	39.683	13.646	23.676	18.681	54.681	59.186	66.105
13.646	53.552	77.875	12.385	61.53	39.614	41.839	40.033	30.381	12.63	28.404	18.707	51.321	32.502	46.799	38.718	36.478	44.891	54.517	16.58	38.915	15.386	31.191	22.467	17.958	82.775
38.915	64.537	71.085	11.905	32.736	36.684	30.423	22.759	17.017	47.619	33.576	20.968	26.189	47.985	63.492	38.469	34.143	16.728	58.313	34.449	35.542	14.55	14.55	30.423	16.672	34.913
35.542	44.442	31.771	16.747	39.447	39.071	15.873	12.49	29.881	36.271	23.531	43.516	54.387	27.023	17.196	21.164	27.778	70.028	46.164	22.435	19.841	11.343	17.196	22.877	15.6	45.409
19.841	15.983	53.178	25.545	22.487	14.774	20.577	15.386	50.617	30.866	60.25	50.607	21.639	18.62	40.219	9.259	41.099	25.132	24.98	68.054	37.973	21.164	19.829	30.591	8.303	33.057
37.973	82.007	23.474	30.339	47.476	23.635	20.881	18.636	33.068	25.132	46.005	24.687	44.974	40.016	55.184	42.236	56.878	56.339	39.621	52.633	27.77	26.04	11.7	31.187	30.662	36.043
27.77	59.61	17.156	57.919	42.055	7.932	43.063	35.147	11.016	25.132	25.773	36.772	56.811	47.047	44.974	9.259	27.755	33.354	37.264	18.519	18.94	34.645	35.926	18.94	14.459	44.37
18.94	106.666	66.869	51.958	34.392	12.311	12.428	34.313	22.828	37.465	23.083	24.318	52.006	53.138	65.526	23.224	52.939	28.397	29.763	33.331	11.572	11.905	16.654	37.949	11.905	53.87
11.572	46.296	47.594	57.972	21.26	71.353	14.798	11.248	11.47	32.877	36.897	43.413	69.199	11.209	43.192	29.001	44.566	38.817	14.681	22.487	18.264	33.069	13.228	74.554	24.895	10.592
18.264	38.36	13.696	45.888	31.257	41.313	17.196	36.795	61.429	27.967	48.221	33.609	57.053	89.089	18.309	42.328	40.476	11.838	14.55	44.58	31.746	54.448	26.999	67.451	19.21	14.017
31.746	55.556	31.746	23.676	16.836	40.554	28.995	17.572	37.037	50.508	40.691	19.895	59.053	30.971	65.125	83.153	35.312	34.352	24.93	34.392	9.259	20.249	17.196	55.622	13.025	22.487
9.259	37.537	78.843	70.567	25.622	42.027	24.682	76.957	28.887	36.176	31.924	32.051	14.965	15.873	23.032	33.98	11.905	64.578	52.91	46.335	53.442	12.475	40.626	26.956	49.603	21.553
53.442	36.015	48.187	67.638	27.455	27.807	28.427	14.333	25.188	18.378	28.184	44.886	62.816	19.841	35.542	68.512	52.285	43.746	62.326	20.711	9.353	23.008	22.927	30.866	15.331	53.179
9.353	70.593	45.196	26.478	21.639	28.753	9.259	23.346	23.09	27.506	48.942	59.331	33.048	60.796	56.878	79.387	35.638	13.242	35.004	40.829	35.873	47.619	11.375	21.743	19.841	48.084
35.873	49.906	45.967	41.576	67.744	47.691	46.296	18.34	36.188	51.587	29.484	64.803	60.244	84.579	19.841	25.072	40.38	52.364	40.069	57.99	28.042	28.764	22.603	22.459	49.171	15.345
28.042	35.916	72.854	19.048	15.972	28.654	25.459	20.714	47.291	27.544	43.266	21.352	59.237	23.515	14.438	49.589	28.266	34.754	22.487	20.622	14.398	13.228	42.348	53.184	30.071	16.476
14.398	56.341	72.25	15.873	18.45	35.381	58.649	32.894	12.424	52.079	48.541	15.873	53.791	83.186	15.873	21.394	24.515	23.397	26.175	32.558	10.151	11.871	43.254	27.056	13.228	26.63
10.151	22.312	16.086	66.659	41.391	22.435	45.882	27.625	16.104	39.154	24.295	12.693	58.897	10.077	75.761	55.103	74.461	59.861	59.092	60.386	13.478	33.4	33.432	23.32	28.131	61.937
13.478	30.837	67.283	65.134	35.714	40.188	17.056	34.753	23.076	34.231	24.822	29.489	47.334	70.574	74.074	79.434	42.328	51.773	32.67	41.429	37.8	25.009	46.026	25.067	9.259	29.654
37.8	34.902	64.105	23.354	16.34	14.009	40.434	25.809	23.383	16.412	27.807	29.16	33.69	42.438	75.761	57.055	48.005	56.599	16.836	14.754	45.485	47.244	26.455	42.312	19.524	41.291
45.485	42.587	22.492	28.765	25.062	31.231	20.032	35.391	40.652	32.402	59.828	41.005	70.744	38.044	56.591	21.095	16.746	60.829	25.132	63.228	13.099	42.328	44.974	11.287	11.4	65.668
13.099	98.856	59.524	22.448	54.899	36.685	54.893	9.628	53.284	37.928	41.794	36.478	51.116	63.492	65.395	62.169	22.402	77.665	64.815	23.909	29.251	28.995	9.787	13.619	13.195	47.534
29.251	34.392	74.933	16.4	23.913	14.55	20.601	13.618	12.277	30.423	44.415	28.06	41.005	68.073	40.37	53.359	34.387	20.711	69.954	55.024	38.168	14.482	27.778	10.006	30.423	34.607
38.168	22.434	72.189	38.831	36.293	29.907	35.613	71.429	19.841	37.215	56.946	63.649	77.452	31.576	33.047	75.916	60.918	79.48	54.233	44.738	15.141	21.518	28.348	81.331	28.06	32.891
15.141	29.549	32.433	64.133	47.818	42.041	25.304	46.456	35.714	12.515	31.969	39.697	40.875	53.723	31.9	58.143	44.352	22.487	36.658	23.81	47.768	44.055	31.336	55.078	26.936	23.538
47.768	53.437	26.879	44.562	39.965	51.159	13.609	24.408	28.717	33.069	41.394	12.968	43.392	22.013	55.654	14.965	47.913	16.262	25.132	34.873	24.461	10.582	45.028	58.6	15.873	55.154
24.461	42.041	13.919	85.42	40.608	31.534	20.551	38.82	36.311	10.582	37.108	11.251	40.806	11.656	49.337	46.345	55.556	56.878	53.58	25.132	13.228	11.905	18.917	33.458	21.774	22.487
13.228	89.765	69.38	37.819	41.818	10.401	18.672	52.302	15.873	14.786	45.61	31.545	48.304	44.446	46.54	61.16	47.402	62.365	48.774	60.118	16.412	47.6	21.164	13.095	37.867	38.277
16.412	94.338	29.338	44.758	35.153	13.095	34.973	54.249	11.224	10.295	39.224	34.001	64.872	46.25	18.363	84.227	16.481	44.719	15.873	38.36	21.739	39.529	28.96	57.715	22.12	17.576
21.739	20.963	24.421	20.197	95.35	20.044	33.729	53.063	25.933	39.144	41.133	48.266	31.191	27.692	13.761	47.706	46.147	27.817	48.558	56.792	33.919	43.309	31.156	71.938	16.406	49.728
33.919	30.41	59.524	13.087	10.098	42.328	20.626	63.138	45.27	18.519	34.606	75.486	40.459	30.236	54.68	25.804	14.55	58.568	44.296	14.427	21.592	18.201	32.621	14.696	41.159	60.313
21.592	31.621	40.139	23.81	44.974	20.459	23.742	9.259	50.822	34.646	10.46	54.154	61.368	46.522	38.2	18.519	27.266	50.265	36.258	63.776	29.69	24.78	39.064	67.989	48.942	10.849
29.69	73.367	46.344	11.294	70.106	8.701	19.331	33.003	60.125	8.613	31.746	51.303	50.615	81.702	13.023	25.132	25.249	55.199	21.562	43.222	9.259	27.217	8.88	41.803	21.298	80.444
9.259	51.243	42.43	41.005	42.838	23.81	49.711	16.321	30.163	40.865	19.334	12.372	30.423	54.7	39.781	58.16	11.719	34.801	23.903	43.605	27.124	11.905	31.487	14.883	22.487	45.518
20.309	47.392	67.598	89.235	36.332	19.683	39.949	21.513	13.629	30.866	33.487	27.223	53.609	68.069	10.929	54.26	31.372	68.957	40.526	42.733	15.891	15.386	13.844	17.151	30.423	26.64
27.124	35.206	10.546	54.233	32.776	19.603	38.641	27.778	42.777	48.075	17.454	41.612	50.007	16.951	80.555	54.792	48.711	55.457	61.732	49.137	10.388	26.455	19.232	18.739	15.308	29.042
15.891	32.436	39.416	26.21	9.259	52.91	32.571	29.371	54.233	31.746	52.602	31.572	44.091	50.265	16.736	83.333	13.413	9.259	34.928	10.591	11.834	38.146	29.101	56.603	21.164	37.037

10.388	54.497	23.81	20.908	40.832	34.261	12.431	38.204	44.896	55.038	22.029	32.287	27.356	98.622	81.949	10.02	34.91	33.679	32.603	23.684	16.573	24.209	7.906	16.092	19.816	46.92
11.834	26.651	38.36	48.445	54.367	13.228	40.439	11.851	15.582	22.487	81.34	44.083	58.482	61.59	9.259	21.965	12.848	60.936	19.06	45.769	23.151	51.615	11.241	33.337	19.841	60.517
16.573	43.651	22.321	51.921	38.121	21.023	14.21	24.28	34.303	26.493	55.268	19.403	28.707	34.527	7.937	46.317	12.968	17.757	51.587	24.256	15.354	17.18	44.767	41.183	33.069	36.694
23.151	42.328	50.265	55.81	24.849	22.452	16.836	14.03	10.295	26.313	47.34	30.575	61.748	51.776	73.078	15.062	12.484	45.558	38.157	14.965	14.756	41.616	18.519	15.784	32.015	12.263
15.354	38.74	32.736	82.999	40.286	24.211	59.712	40.026	70.106	9.429	36.226	18.524	39.745	52.378	15.062	30.53	92.748	39.683	12.622	55.277	20.599	30.423	50.508	17.777	47.241	26.623
20.177	21.164	41.682	48.933	57.581	17.196	19.874	42.41	23.353	18.555	19.841	23.376	50.771	13.806	93.736	21.164	65.737	57.806	72.78	40.73	15.611	14.138	21.763	43.087	32.235	13.095
31.952	53.505	67.956	28.26	35.692	22.459	19.854	23.383	33.901	16.966	19.841	36.637	40.514	34.733	48.986	68.535	45.081	23.77	48.463	41.005	18.519	17.017	25.69	55.688	50.571	10.524
14.756	62.896	56.263	18.707	60.546	20.2	13.02	20.461	21.225	43.172	37.814	34.973	46.679	80.311	46.953	27.124	37.179	35.714	55.133	21.142	25.517	24.705	25.132	10.14	19.841	17.196
20.599	46.909	71.938	27.826	42.311	29.265	16.216	13.702	19.362	25.254	46.902	11.905	53.776	78.354	63.424	41.005	17.196	63.602	14.118	38.197	17.191	42.692	12.61	25.449	21.997	18.207
15.611	54.666	28.878	16.225	24.695	66.02	17.626	19.908	27.81	13.228	54.074	51.951	62.232	29.851	51.254	65.231	27.593	29.93	18.519	55.907	17.47	25.8	33.069	38.381	30.561	20.121
18.519	46.12	60.876	14.541	34.53	30.768	20.042	11.235	47.597	22.113	22.59	50.723	18.167	78.689	38.18	39.362	28.744	22.487	23.041	52.472	20.555	13.856	16.745	38.948	25.106	45.468
25.517	42.566	18.815	31.464	44.064	63.512	59.259	47.209	32.865	37.438	32.116	55.192	40.847	64.811	60.214	39.89	22.361	47.702	57.749	73.275	31.549	27.916	23.008	31.408	19.465	15.976
17.191	60.145	52.505	47.784	34.415	54.323	24.391	63.802	9.259	23.793	34.207	50.891	49.055	15.163	19.152	65.118	46.763	65.54	45.997	15.784	19.601	25.805	30.423	22.639	26.64	18.677
17.47	82.211	66.931	60.458	24.266	40.929	14.55	23.692	39.645	41.717	52.618	45.241	50.719	50.831	49.332	18.897	22.487	57.241	52.91	31.746	41.005	39.683	11.421	51.869	46.176	59.372
20.555	13.808	36.004	79.442	29.362	32.828	18.72	25.132	39.724	38.516	14.55	19.35	65.963	25.554	17.005	67.588	17.869	57.03	26.189	40.998	14.55	11.905	32.633	79.666	28.646	21.926
31.549	48.96	36.867	36.264	56.878	44.974	25.245	13.27	23.578	60.847	43.923	26.508	73.227	24.906	38.857	20.639	42.201	51.587	45.195	13.258	18.006	13.228	19.966	49.496	14.799	26.627
19.601	47.587	34.607	57.742	42.328	31.746	56.264	11.336	41.005	33.909	12.391	30.449	66.639	30.423	37.037	11.592	65.29	40.376	41.834	13.095	16.747	12.134	27.958	27.787	9.793	31.374
41.005	33.168	77.333	77.404	67.146	32.736	68.328	26.455	19.783	12.159	58.926	59.342	60.394	23.241	8.808	68.181	54.941	33.596	23.81	36.478	17.196	33.069	20.926	62.169	13.615	16.84
14.55	15.442	36.448	35.453	36.384	23.275	35.552	67.643	26.711	36.39	27.333	12.159	43.903	57.99	14.765	94.495	29.101	69.813	43.012	43.651	12.117	15.062	41.137	18.864	22.686	53.009
18.006	77.303	86.657	19.648	17.648	19.7	10.695	10.884	38.14	26.944	24.293	56.961	38.597	70.328	75.051	47.619	43.098	27.523	46.477	52.674	19.841	10.582	9.741	31.314	39.683	16.747
16.747	25.777	32.943	70.149	42.606	45.603	52.91	35.551	19.747	72.336	13.776	42.606	41.876	88.655	40.924	51.935	13.388	46.384	61.082	56.46	29.377	11.241	9.304	34.112	19.506	67.965
17.196	26.591	18.519	76.624	18.347	22.487	22.716	20.08	57.181	19.841	40.38	23.354	56.829	32.088	39.363	36.834	34.043	45.129	10.128	25.946	43.025	15.482	20.684	26.189	11.905	15.873
12.117	23.672	79.241	29.626	17.729	52.593	12.835	15.901	28.601	32.077	37.134	39.665	42.891	62.169	48.404	31.235	31.693	37.746	46.605	16.144	23.618	27.805	25.527	35.409	9.477	32.526
19.841	40.839	49.609	62.79	32.79	43.027	25.446	47.602	34.294	27.778	37.914	43.96	49.609	69.632	37.107	30.281	41.141	69.684	12.246	65.726	29.484	15.661	11.995	62.294	19.29	42.649
29.377	28.976	70.102	59.22	58.468	39.07	26.093	32.758	22.759	15.601	53.028	68.505	62.704	14.745	80.688	69.833	50.295	22.417	61.436	20.785	20.353	34.133	50.552	29.746	15.875	23.276
43.025	22.015	12.676	27.202	27.437	13.753	37.277	33.888	35.551	49.46	32.706	19.642	61.732	57.314	75.397	63.919	56.509	48.779	59.474	41.081	35.542	62.169	24.886	17.333	42.921	29.127
23.618	17.163	47.107	79.187	66.096	27.418	30.192	47.013	24.261	25.132	37.5	26.455	73.909	75.656	13.8	71.383	62.855	37.881	60.548	35.083	14.118	12.159	66.138	25.254	18.717	32.928
29.484	14.844	22.989	14.55	21.164	22.225	49.543	22.487	25.277	14.685	21.477	26.448	81.992	32.874	54.532	12.614	39.184	48.115	19.298	57.888	17.196	72.464	38.36	33.48	17.196	19.572
20.353	49.558	66.72	93.262	27.124	16.744	44.295	43.407	26.455	40.905	26.292	12.848	30.16	54.233	69.761	32.013	13.701	43.651	59.059	36.236	27.778	19.632	34.656	13.388	30.708	14.978
35.542	57.68	28.302	72.065	39.214	32.736	40.697	11.905	13.13	31.746	10.229	36.826	56.616	36.253	46.272	71.718	15.873	57.83	40.708	29.768	32.435	9.259	31.346	26.82	41.071	25.942
14.118	59.345	57.121	23.489	33.768	8.313	11.819	29.645	13.228	25.937	33.857	21.848	54.249	64.744	21.164	26.641	16.002	54.067	22.915	17.196	35.25	8.9	20.683	40.944	31.852	18.519
17.196	36.97	50.808	46.296	26.776	27.349	39.586	37.76	22.174	44.01	26.189	35.249	25.121	48.46	51.044	71.188	62.474	62.419	46.452	22.448	22.893	19.841	42.583	29.133	10.15	27.741
27.778	25.434	37.02	55.971	21.739	9.47	17.017	22.047	28.407	41.005	17.246	34.607	42.09	53.15	29.98	22.487	34.226	59.485	14.092	16.849	18.519	22.485	50.541	66.172	14.459	17.015
32.435	26.774	38.148	25.113	32.85	17.558	25.94	27.778	45.517	14.092	61.66	46.067	37.207	84.312	42.699	12.323	72.751	13.775	22.842	63.492	32.57	34.253	11.424	67.061	26.455	10.551
35.25	21.969	82.165	41.3	15.267	7.956	11.905	48.513	16.914	13.099	30.348	30.799	34.105	47.859	10.289	26.065	19.776	20.195	20.18	73.227	35.135	11.905	11.994	8.223	18.519	46.79
22.893	51.858	60.258	62.143	11.926	47.607	50.186	11.508	36.5	33.384	27.844	39.561	62.243	53.069	48.817	47.271	9.1	42.718	52.479	19.298	27.817	24.749	38.699	23.025	41.084	38.109
18.519	72.312	39.063	82.834	14.745	81.29	14.281	40.621	28.022	20.684	25.705	33.084	55.592	58.705	32.185	72.723	33.866	43.072	42.495	56.465	31.746	26.455	17.975	9.353	24.853	31.746
32.57	59.132	19.841	91.247	15.237	26.455	19.082	17.763	20.559	16.829	46.663	47.881	45.473	35.272	85.115	16.519	25.132	60.017	58.572	47.866	39.617	20.752	17.744	18.333	19.657	52.91
35.135	59.171	13.228	91.334	9.259	52.216	17.208	15.834	41.521	24.028	51.359	50.479	19.672	18.164	60.055	45.255	23.997	49.944	34.515	26.711	8.28	17.196	25.414	36.735	7.606	48.787
27.817	28.265	58.798	24.212	55.03	15.985	46.157	21.063	21.025	13.228	16.672	13.155	44.974	72.88	79.095	16.317	10.582	18.707	73.464	40.395	23.27	32.532	40.027	52.661	40.635	50.55
31.746	96.046	17.196	22.962	36.951	16.365	10.724	15.661	55.734	29.406	20.613	16.135	57.053	60.365	40.539	59.518	67.738	33.366	56.359	70.045	24.859	15.219	19.215	27.138	39.224	21.049
39.617	72.729	53.176	78.578	41.412	30.258	23.584	18.472	21.105	7.393	22.727	63.916	52.303	14.738	9.259	48.178	52.501	80.65	42.284	49.886	31.197	17.196	19.841	44.352	17.938	27.46
8.28	54.2	63.492	83.478	47.953	47.982	42.05	40.437	55.556	48.245	21.794	35.323	57.304	97.695	59.613	33.132	43.025	62.569	65.257	23.894	50.494	18.519	21.513	15.623	32.397	56.985
23.27	102.375	60.769	21.668	23.632	24.64	33.114	25.132	39.504	30.35	30.065	66.565	47.316	77.632	73.031	17.568	10.955	61.687	9.259	25.006	13.129	19.841	39.003	21.696	42.328	43.819
24.859	40.75	44.564	87.313	68.131	35.098	39.084	21.164	27.273	48.389	62.862	25.446	46.017	67.316	57.702	74.478	57.266	70.742	43.823	51.587	18.841	15.062	20.762	23.872	18.068	54.419
31.197	52.779	50.755	16.99	23.587	25.243	11.332	55.025	48.26	26.455	26.85	13.478	46.532	86.14	17.017	86.22	21.164	73.169	27.591	39.055	15.632	19.841	14.506	11.561	25.787	70.106
50.494	36.774	11.923	37.037	41.479	54.448	66.714	17.771	35.714	59.299	26.455	40.219	91.901	54.321	66.788	31.374	12.343	39.683	26.189	23.913	34.392	30.383	53.479	51.803	16.044	56.554
13.129	38.597	34.326	42.159	36.411	15.784	17.991	14.163	51.888	24.386	22.539	25.132	49.593	22.841	40.994	56.878	25.695	90.485	19.148	59.118	36.463	19.841	27.778	46.222	11.494	56.758
18.841	38.753	57.624	30.303	65.31	19.165	37.611	27.616	15.927	33.069	29.958	15.873	39.159	61.747	13.393	51.443	45.789	63.602	22.487	52.845	16.464	13.87	57.07	23.286	55.556	32.736
15.632	22.487	99.206	26.998	19.841	25.828	58.002	19.75	56.235	24.219	70.703	16.132	45.708	57.78	16.636	20.913	55.429	51.402	33.142	17.571	10.045	27.778	20.661	26.949	31.579	13.095
34.392	19.841	56.815	87.338	61.518	57.658	18.338	11.419	50.748	42.328	27.468	18.519	18.363	35.587	58.707	51.721	20.652	60.736	50.051	39.284	40.822	22.487	10.464	36.001	11.787	41.514
36.463	69.3	31.796	56.379	29.29	9.927	42.854	85.437	25.439	48.16	26.781	36.222	31.953	54.533	61.093	22.962	24.847	37.159	23.052	29.93	19.527	10.289	64.815	10.582	42.328	64.406
16.464	28.934	56.57	65.663	33.586	54.26	37.064	12.973	41.728	23.383	38.818	17.771	53.325	61.539	34.149	10.33	64.378	43.768	11.31	37.134	9.259	25.809	53.305	44.447	43.025	11.387
10.045	46.18	54.16	21.466	51.337	37.037	30.423	20.099	25.178	47.285	31.538	35.908	48.131	79.35	19.148	68.308	28.988	63.977	52.548	27.762	36.467	48.166	12.65	21.298	15.907	22.48
40.822	70.609	63.498	47.538	36.647	49.721	38.36	14.459	10.661	15.482	51.081	17.69	44.691	57.427	58.364	28.641	30.188	34.049	78.942	28.994	31.202	23.628	31.439	48.71	13.532	56.832
19.527	67.777	33.69	20.121	30.651	31.566	16.042	19.448	26.316	30.423	19.841	26.914	48.118	47.977	11.905	51.369	26.455	23.81	25.566	44.317	20.013	21.344	25.132	25.132	13.883	23.931
9.259	88.624	85.115	49.563	21.064	64.216	50.322	55.556	47.606	49.727	46.075	15.873	54.771	70.149	85.047	15.082	60.254	56.878	34.61	38.284	11.905	19.047	12.239	76.843	35.18	68.975
36.467	36.167	35.457	60.554	33.459	13.822	11.868	14.55	14.55	35.362	30.403	42.584	81.938	99.058	62.803	33.048	28.407	59.436	43.78	22.686	14.55	29.338	15.873	56.889	14.767	67.813
31.202	79	12.283	73.026	49.429	14.97	51.528	18.94	41.005	12.159	30.954	42.556	60.891	80.862	64.133	42.644	22.529	26.124	54.761	14.03	33.526	48.129	26.033	21.9	13.654	15.784
20.013	21.677	34.934	78.328	26.125	27.291	28.809	31.92	26.709	35.714	25.581	14.199	45.199	37.864	61.514	22.487	22.448	57.519	27.62	38.36	15.043	28.722	12.159	53.205	19.112	22.487
11.905	45.212	51.423	30.696	41.275	13.018	25.132	17.325	26.412	35.5	20.885	36.396	44.974	38.289	10.841	73.713	44.974	72.183	66.42	51.358	25.351	38.341	32.828	26.054	23.707	22.147
14.55	89.418	51.148	74.852	64.742	26.205	58.052	21.028	35.156	19.525	26.065	25.084	44.631	67.3	39.799	54.482	52.657	24.063	19.841	35.578	21.221	27.537	22.487	66.432	29.151	19.91
33.526	83.286	52.932	56.644	20.124	13.155	43.651	35.253	11.488	45.954	22.841	19.772	54.761	72.771	61.7	68.456	31.206	62.209	10.59	29.101	47.581	53.127	36.528	12.059	17.571	39.284
15.043	36.087	52.836	35.3	68.155	40.739	27.104	16.836	36.226	24.256	20.353	42.351	29.436	23.241	69.48	52.509	65.81	10.503	24.083	22.452	28.216	55.631	25.211	50.205	23.81	14.55
25.351	55.742	31.746	77.941	23.383	16.811	36.455	29.903	40.854	37.749	19.448	49.46	83.223	63.602	66.711	64.089	27.761	58.365	48.245	14.03	32.282	14.982	23.81	46.296	14.55	58.736
21.221	107.201	58.201	58.281	39.466	10.234	16.883	27.766	17.75	28.417	43.281	30.423	55.061	68.109	21.912	19.841	26.321	66.714	19.683	21.164	37.847	26.425	21.092	15.873	37.352	12.484
47.581	16.423	63.547	37.59	62.468	27.679	40.651	11.656	41.649	18.053	25.254	49.541	61.147	75.74	29.101	11.254	20.904	21.023	62.748	33.069	60.473	59.127	33.985	49.524	14.559	18.519
28.216	22.302	69.273	21.292	61.123	26.636	35.139	57.596	78.369	16.421	10.582	76.306	74.246	65.039	69.951	20.117	35.195	30.48	59.051	21.164	34.731	15.661	11.905	26.846	14.03	57.207
32.282	38.837	38.318	37.413	44.017	29.395	32.732	35.455	25.132	46.377	65.485	28.812	80.834	26.056	66.192	60.454	39.458	39.894	41.913	15.402	52.751	41.005	12.934	33.808	51.476	20.639
44.815	29.637	49.971	32.621	38.36	23.848	42.328	20.979	37.986	23.616	23.394	29.93	53.913	66.472	26.51	18.917	37.864	41.334	42.351	11.752	25.812	29.93	17.007	16.545	45.584	79.732
37.847	66.129	37.777	54.134	35.798	29.101	18.519	43.6	55.717	39.279	35.714	34.392	42.176	31.6	70.459	55.556	43.884	33.069	37.775	63.699	30.976	11.905	9.259	32.677	18.288	33.725

60.473	29.648	26.285	20.076	37.085	31.746	34.894	14.55	30.423	20.708	38.314	13.228	51.967	44.145	15.873	41.005	63.616	27.778	66.087	12.552	17.196	22.487	11.555	44.904	13.515	48.723
34.731	35.275	60.534	88.856	38.919	27.778	39.683	33.069	24.424	54.233	21.026	21.926	45.36	62.089	59.165	13.228	36.602	17.698	15.799	36.861	15.794	15.873	29.101	23.467	51.538	50.001
52.751	87.774	21.142	57.665	17.156	14.55	50.508	18.354	30.771	21.164	59.524	34.326	62.169	80.01	63.453	49.023	21.305	31.379	57.423	29.101	47.346	32.888	23.498	40.434	16.593	59.012
25.812	75.254	23.684	83.164	30.359	14.742	31.119	28.633	17.196	64.215	35.233	50.739	46.437	38.017	37.037	68.425	32.619	62.358	32.552	53.988	47.921	56.86	33.974	26.796	9.259	76.534
30.976	41.971	83.244	26.534	57.772	97.952	51.39	25.221	31.905	34.15	32.88	31.762	63.958	66.849	79.755	62.94	32.389	61.386	13.228	52.702	39.437	25.132	26.455	20.128	26	29.516
17.196	14.93	60.118	36.624	29.744	62.148	48.019	12.869	18.707	48.106	44.877	53.333	38.469	25.509	45.621	18	46.352	31.945	31.372	34.971	8.763	24.797	30.423	72.2	60.311	38.866
15.794	80.06	42.062	78.968	45.712	31.746	47.239	17.571	21.164	13.414	29.84	28.417	62.155	53.696	28.745	22.735	54.069	54.886	14.398	41.912	14.55	22.487	18.707	19.224	40.452	74.035
47.346	61.785	43.562	13.608	46.203	30.623	11.224	49.3	50.09	29.545	32.648	37.715	65.156	27.201	33.218	45.575	40.366	19.841	61.761	44.78	19.532	12.21	35.145	22.786	12.311	74.475
47.921	36.966	63.316	68.783	29.987	43.544	13.095	23.383	45.428	35.97	37.986	34.801	36.471	28.854	22.115	15.442	23.81	23.669	22.749	14.202	39.657	46.296	17.166	44.896	68.194	40.084
39.437	23.676	63.13	43.466	28.753	22.24	47.368	7.937	41.928	40.368	18.078	32.327	67.224	83.636	56.284	62.912	10.46	47.065	26.734	61.659	19.167	13.608	18.519	24.565	42.556	43.914
8.763	17.571	20.015	17.518	43.473	19.624	40.401	20.577	55.017	43.455	35.495	40.936	49.835	32.629	17.196	31.282	44.825	60.183	14.469	42.777	30.423	15.873	23.81	29.785	25.132	25.132
14.55	29.101	31.285	54.463	29.101	23.018	34.264	11.551	65.437	35.557	28.852	35.042	26.83	34.718	97.543	58.566	41.861	46.199	37.631	32.908	20.911	33.069	19.251	66.327	32.648	35.766
19.532	36.781	23.864	94.423	37.861	39.963	15.897	18.43	40.906	20.637	23.679	66.851	50.095	24.682	70.84	69.639	31.417	55.558	45.382	48.097	18.84	59.611	15.386	71.977	22.487	19.648
39.657	48.469	52.521	24.635	45.83	42.511	19.841	34.264	51.212	42.566	27.22	17.699	36.961	76.987	22.487	65.473	47.619	69.918	18.519	25.132	43.431	30.344	40.228	51.277	25.456	10.04
19.167	15.482	62.169	64.59	54.66	39.818	13.228	11.65	14.03	47.702	32.433	59.258	79.714	41.763	79.768	26.786	23.168	71.783	21.597	16.174	21.888	16.253	33.649	14.457	28.243	18.519
30.423	71.429	100.561	35.348	48.064	57.93	26.975	11.119	42.328	14.162	20.786	28.06	32.736	69.902	56.137	21.895	15.77	72.352	52.025	11.905	21.754	11.838	41.005	47.674	25.09	17.354
20.911	58.889	95.985	15.873	46.091	26.845	42.673	11.119	26.376	14.21	61.928	31.746	63.588	50.899	52.527	39.683	40.358	43.965	64.564	50.908	18.519	18.519	11.905	76.72	19.841	47.477
18.84	57.354	48.301	46.996	25.132	52.935	49.419	23.35	19.225	33.841	34.162	26.684	44.566	46.586	74.476	13.951	56.878	17.771	28.995	48.231	10.582	9.259	27.119	8.833	58.802	20.668
43.431	67.46	20.117	29.594	25.661	46.639	39.406	17.417	24.185	41.976	30.423	39.89	40.033	56.714	48.936	39.284	21.635	42.61	76.72	19.841	18.228	28.884	29.101	17.33	66.402	18.917
21.888	60.122	25.346	9.399	51.453	30.311	41.005	26.139	43.651	26.623	15.731	15.804	62.882	69.384	50.508	31.129	51.709	44.577	25.664	28.934	31.606	38.656	20.929	17.196	53.534	32.077
23.376	74.955	67.125	53.233	33.335	7.906	13.018	30.959	64.124	27.506	22.487	25.254	49.361	68.786	72.284	70.817	25.132	52.722	19.642	23.493	13.228	75.55	14.409	59.605	43.122	15.873
21.754	58.694	22.487	39.364	69.084	33.672	24.784	47.829	45.148	54.204	33.079	40.12	42.473	60.738	48.637	24.779	20.257	72.19	61.774	52.814	12.49	14.205	40.153	26.991	14.559	29.828
18.519	38.071	43.209	55.42	37.053	30.483	18.707	28.017	49.617	22.617	18.846	39.516	74.443	15.382	11.724	37.037	42.871	20.698	71.413	28.258	12.869	30.726	12.425	17.627	48.103	45.363
10.582	19.841	30.086	32.53	25.346	11.035	41.78	19.811	38.034	16.131	63.238	25.042	47.281	101.852	25.116	63.053	60.873	17.381	59.15	62.085	28.397	24.628	9.817	20.577	46.854	48.445
18.228	38.106	84.599	43.623	37.037	30.496	53.169	11.233	8.327	27.017	54.006	29.432	67.116	81.961	22.487	47.125	22.503	18.519	32.197	59.524	16.174	28.348	40.01	9.21	40.537	44.383
31.606	64.486	47.449	55.755	20.91	26.257	17.839	31.746	51.244	18.569	24.465	51.529	19.448	59.861	52.142	64.815	26.043	89.661	34.392	50.253	17.196	25.763	19.537	19.841	28.994	35.025
13.228	74.81	85.115	80.532	65.848	11.905	32.539	69.64	19.642	30.837	36.272	41.074	58.111	52.732	62.682	47.015	42.861	43.651	27.593	18.519	23.169	48.843	29.101	38.581	53.683	46.386
12.49	80.647	52.266	61.368	37.037	50.061	27.411	15.701	24.138	28.566	32.154	66.871	57.519	22.468	16.531	71.289	39.683	16.53	46.618	79.736	29.101	20.847	8.877	24.849	47.195	44.269
12.869	57.637	22.598	53.042	15.465	16.849	72.954	31.387	33.415	19.841	31.32	14.442	42.934	29.581	21.192	58.556	53.461	34.772	44.974	30.117	14.652	36.627	29.254	37.429	35.494	51.258
28.397	55.232	13.248	64.015	31.772	52.628	20.668	23.81	13.228	53.837	11.905	27.97	57.116	16.54	74.937	60.403	44.655	52.714	54.431	46.296	9.224	13.895	28.674	84.008	50.718	26.869
16.174	79.754	50.924	73.375	17.036	30.236	13.434	7.937	31.727	44.974	34.209	35.252	64.192	17.196	52.91	47.096	54.468	14.55	25.763	56.463	38.408	17.132	29.481	13.608	26.781	28.955
17.196	67.833	16.338	52.72	21.164	22.312	51.541	45.557	63.558	46.699	26.033	34.01	62.299	20.592	57.3	12.822	44.287	14.03	18.519	33.413	15.873	23.091	32.229	68.921	23.199	22.289
23.169	16.388	52.683	77.196	68.163	7.937	14.497	33.619	55.236	40.73	57.786	46.296	77.403	36.269	39.683	32.497	26.399	23.449	23.346	23.672	28.397	21.583	34.264	33.043	58.912	20.553
29.101	14.55	50.076	44.812	24.227	28.246	32.296	15.616	47.619	45.602	15.402	23.81	62.707	81.575	37.05	65.473	18.347	31.372	32.281	14.183	39.959	67.962	18.519	17.623	17.196	13.228
14.652	45.771	47.131	77.738	34.434	35.486	15.873	52.737	35.194	50.637	39.633	18.983	55.737	42.09	29.101	49.629	20.045	21.994	28.774	25.051	16.254	19.82	22.487	26.525	41.005	43.101
9.224	50.627	14.521	45.109	38.545	18.519	46.454	77.735	36.118	19.842	41.504	73.262	41.075	76.825	58.47	64.145	24.062	82.913	68.487	44.281	17.426	23.095	18.519	52.273	32.663	13.228
38.408	65.678	65.754	70.38	35.141	28.464	13.015	11.224	41.839	23.678	60.547	44.442	60.198	51.435	39.184	46.217	36.455	44.974	26.781	32.621	32.969	35.306	7.937	21.795	58.505	56.357
15.873	66.485	17.753	46.262	23.81	41.979	37.063	17.196	50.288	30.983	31.746	75.936	41.463	33.106	57.342	47.782	32.581	83.243	18.077	39.683	17.844	33.069	12.707	14.55	14.55	19.766
28.397	61.358	75.502	26.142	26.053	21.365	38.494	10.882	21.989	51.596	39.279	52.292	52.278	69.241	86.475	46.497	36.05	27.291	24.267	38.311	30.235	16.699	15.432	13.488	56.403	46.436
39.959	65.4	20.86	49.698	42.429	58.201	27.778	20.884	49.379	22.759	37.008	63.551	47.068	66.628	55.989	23.282	69.214	51.496	47.049	33.436	19.247	84.532	21.513	13.228	35.493	16.836
16.254	61.073	52.354	70.439	54.249	26.059	25.821	21.865	23.031	19.428	26.545	21.965	35.654	22.995	18.608	11.304	51.644	22.238	56.375	29.385	20.711	20.622	42.799	14.55	23.81	62.047
17.426	46.26	25.146	73.53	24.511	8.701	13.625	22.487	29.101	41.582	27.213	22.14	38.162	57.964	95.193	55.13	67.558	44.931	30.939	38.066	20.896	26.684	15.873	35.889	43.651	26.58
32.969	15.711	36.882	53.476	35.322	56.215	20.584	35.249	47.611	51.987	50.137	20.437	74.56	62.811	35.381	21.298	50.637	44.971	48.29	7.77	33.583	35.754	59.61	23.81	35.714	43.408
17.844	39.838	59.165	22.487	24.85	45.53	42.985	9.948	35.287	33.902	29.561	41.71	39.045	30.701	35.514	50.667	49.181	48.664	31.681	13.025	17.363	13.478	16.53	16.836	64.815	58.474
30.235	75.728	92.304	65.287	59.149	89.835	44.253	16.826	70.667	28.264	31.746	54.596	60.053	21.097	40.299	21.374	48.416	38.231	67.875	14.202	10.268	29.103	47.912	23.665	45.192	34.665
19.247	22.336	33.65	91.659	51.758	13.228	45.994	20.711	30.696	16.785	35.423	11.375	76.556	65.671	61.462	22.593	58.49	18.053	53.553	22.487	45.601	20.661	61.817	59.792	21.553	35.09
20.711	66.612	65.068	60.938	78.246	11.253	33.401	13.228	47.26	51.715	11.905	36.535	48.942	20.787	76.299	22.715	15.755	62.44	60.673	59.748	12.349	16.63	37.016	17.548	37.387	33.502
20.896	36.093	59.486	54.584	28.797	17.379	37.277	19.138	41.419	45.98	54.249	48.83	47.331	60.696	33.069	68.783	18.815	53.556	59.886	57.604	30.144	11.905	25.132	62.575	26.455	44.378
33.583	29.702	86.289	19.684	40.479	15.213	30.948	23.7	31.711	29.405	14.55	24.074	10.582	59.593	78.929	58.628	49.359	23.81	54.559	18.707	11.905	26.455	9.601	19.093	19.295	49.572
17.363	35.507	14.55	49.268	29.199	20.085	13.388	22.448	50.052	26.455	23.632	43.651	70.727	59.524	52.367	10.289	29.93	73.022	53.882	51.888	36.175	9.353	38.329	18.41	29.101	47.276
10.268	55.517	17.005	38.36	62.732	36.763	24.266	8.88	21.627	21.354	42.2	39.044	50.619	55.283	21.164	71.429	65.473	53.314	48.177	33.291	13.78	26.674	20.661	11.715	73.321	20.515
45.601	52.408	95.238	63.547	13.646	15.759	39.784	14.122	31.431	22.448	32.549	20.876	48.883	43.907	30.464	47.328	29.484	37.021	29.86	17.196	52.575	22.199	59.524	39.723	64.634	27.778
12.349	60.925	82.793	24.418	30.876	18.909	35.082	14.511	8.919	33.069	36.761	34.202	56.391	31.63	11.425	71.241	48.13	68.843	19.047	48.294	34.741	51.951	19.239	39.345	43.088	75.631
30.144	12.342	45.621	46.766	36.726	19.596	40.294	7.937	14.258	44.777	68.289	32.437	105.869	18.186	67.319	56.615	27.778	49.122	34.392	14.179	20.593	23.81	31.746	19.925	49.281	46.73
11.905	13.561	62.169	97.737	13.839	22.793	30.302	31.746	33.136	18.954	35.633	64.538	50.896	85.641	20.949	48.321	68.054	40.997	48.408	29.091	17.532	14.55	17.741	21.467	43.25	51.971
36.175	37.731	34.392	11.241	36.855	25.668	39.559	9.006	29.696	17.196	13.608	48.278	60.829	80.776	50.377	54.232	58.008	59.798	24.022	49.683	20.08	28.208	14.55	14.55	33.635	33.672
13.78	17.17	32.891	34.258	44.352	62.185	37.751	10.224	56.596	21.76	16.53	35.96	53.095	49.572	30.22	22.029	54.574	31.433	48.942	20.559	39.683	39.683	38.456	17.106	39.697	28.994
52.575	27.642	52.144	59.056	58.846	55.556	46.592	14.535	26.455	32.757	37.554	41.154	69.667	33.495	26.04	29.101	27.529	36.376	13.8	46.763	17.363	15.813	18.583	43.478	43.046	20.156
34.741	66.467	26.455	17.753	52.129	37.037	16.965	69.301	45.186	30.784	17.196	35.827	62.644	23.81	45.404	29.133	35.55	36.041	42.246	51.254	13.57	64.857	25.322	39.06	26.455	64.942
20.593	27.767	30.423	74.776	33.954	23.378	15.873	27.087	39.918	37.871	26.21	25.711	71.352	104.465	26.738	61.495	59.655	16.935	31.356	15.873	13.778	39.667	24.151	17.196	26.022	49.994
17.532	33.666	72.751	59.206	56.874	45.045	27.067	21.164	9.259	14.978	33.527	9.259	59.677	44.756	55.184	14.55	37.618	52.773	75.028	34.998	50.227	22.448	11.905	18.553	9.505	65.026
20.08	72.955	25.632	48.324	36.034	31.446	16.921	26.955	57.309	53.963	56.772	39.249	62.485	76.217	86.979	28.876	29.013	24.761	74.079	50.557	30.175	28.488	9.259	34.295	11.701	17.689
39.683	63.492	102.878	64.632	31.801	18.707	30.765	13.095	25.037	13.166	44.8	35.327	39.683	17.563	43.651	27.384	32.185	10.654	38.552	29.758	20.131	18.649	13.022	23.581	36.939	41.154
17.363	62.412	69.813	26.189	45.688	26.083	45.195	40.754	22.487	17.196	27.718	41.488	64.27	16.365	47.945	24.057	75.651	26.298	57.087	37.156	29.101	20.385	60.523	42.985	44.303	44.928
13.57	15.873	13.999	45.48	13.727	34.303	22.487	21.334	29.987	54.254	33.384	45.796	54.342	77.537	13.181	65.209	49.834	75.441	56.106	37.884	14.092	14.762	16.558	29.946	45.831	63.937
45.853	42.195	35.769	77.293	36.02	22.487	27.803	33.075	35.26	77.539	17.196	51.787	59.753	30.423	58.938	51.301	48.485	58.201	32.28	22.487	11.014	24.382	23.638	19.841	61.51	10.797
13.778	11.572	48.942	51.572	17.275	33.297	52.478	15.873	31.423	53.423	16.902	35.226	94.446	31.615	9.109	20.577	35.714	12.159	31.108	38.27	21.433	41.542	34.129	31.566	18.849	18.223
50.227	16.527	28.556	55.724	28.397	26.455	37.775	12.361	29.591	48.413	25.811	14.327	72.774	57.752	41.429	48.463	44.966	67.095	45.25	40.754	25.338	10.582	33.923	60.059	40.886	21.026
30.175	50.501	23.116	46.269	77.604	30.775	51.587	38.468	19.85	64.538	41.078	14.965	59.295	16.963	51.878	50.265	55.024	63.791	55.562	21.164	20.119	34.243	15.738	32.85	36.826	48.246
20.131	38.9	70.209	56.463	37.774	49.729	21.15	11.181	46.18	52.709	26.513	22.958	43.651	10.128	47.073	29.104	40.173	61.472	38.36	31.801	16.609	26.627	25.813	39.683	61.276	45.512
29.101	29.152	78.57	50.265	42.03	18.289	29.93	11.905	22.482	56.564	26.961	50.815	86.509	60.992	57.7	20.954	80.334	64.429	50.938	35.206	73.877	25.112	19.096	19.841	25.111	30.928

14.092	10.064	81.587	79.516	44.506	33.069	46.272	25.239	13.547	40.698	47.848	9.353	54.132	15.873	23.81	23.213	54.833	36.292	47.328	19.892	28.255	19.841	17.196	48.916	47.522	28.995
11.014	33.879	82.555	53.265	22.032	18.288	13.21	18.519	16.321	18.529	18.226	33.139	64.588	20.619	65.283	19.089	22.874	73.535	13.326	20.08	27.778	35.714	57.978	47.046	17.982	45.485
21.433	44.129	53.045	56.213	21.213	30.903	24.687	19.919	66.185	42.085	38.307	22.918	39.135	70.261	29.348	20.639	31.07	40.845	44.15	48.532	13.529	27.44	11.719	24.997	52.732	31.746
25.338	47.391	18.333	18.934	35.714	49.365	35.818	11.599	39.984	29.821	27.793	55.189	60.161	50.606	55.873	35.714	57.104	43.092	37.746	11.636	16.836	69.139	38.36	43.025	50.493	32.224
20.119	64.589	62.144	91.377	18.924	60.554	39.282	63.142	13.095	19.421	72.484	28.284	40.873	62.332	46.287	24.526	61.415	24.73	52.768	14.323	31.766	21.339	21.547	50.265	42.549	14.257
16.609	24.981	60.459	39.683	25.299	28.057	18.431	12.456	33.687	27.021	45.6	42.767	45.561	77.648	61.87	37.451	46.822	23.81	30.107	60.123	14.681	31.808	39.67	51.844	51.682	10.884
73.877	20.843	30.12	71.864	23.81	21.504	52.91	25.541	23.795	23.312	25.41	31.746	43.651	98.156	84.731	35.57	63.902	56.159	15.085	20.577	17.196	12.159	11.687	32.736	37.037	55.604
15.236	40.442	67.289	36.284	51.785	17.196	18.273	10.289	24.393	37.552	18.288	55.67	56.673	11.921	18.519	9.019	50.396	66.138	33.069	29.654	26.399	21.72	11.776	22.464	39.087	26.455
28.255	40.98	79.563	43.209	12.792	19.642	20.708	16.836	37.875	16.57	19.841	21.387	41.809	48.942	30.971	79.153	26.221	59.238	32.784	50.216	16.157	39.226	14.03	41.581	57.845	68.5
27.778	19.999	68.986	46.786	31.07	27.364	22.666	14.55	54.864	27.791	35.714	36.533	59.08	42.546	61.067	42.395	22.154	17.794	73.651	27.22	12.024	37.877	20.15	24.256	27.634	78.042
13.529	73.953	58.236	33.473	23.81	20.147	23.745	16.552	19.841	13.515	47.619	19.841	79.049	18.311	47.928	18.621	44.8	79.365	39.452	51.119	37.219	33.825	34.607	45.831	61.938	33.712
16.836	31.044	32.549	16.763	27.8	13.967	12.977	31.746	28.674	14.965	31.264	9.259	62.07	21.407	47.95	44.274	47.815	40.427	43.651	61.682	17.771	26.953	23.81	43.626	53.936	43.025
31.766	50.265	39.665	21.122	30.736	16.609	41.517	46.353	29.746	48.284	17.879	50.457	46.446	50.065	69.645	33.069	58.208	38.656	48.841	37.053	19.409	13.228	52.247	30.841	38.931	16.686
14.681	46.744	47.702	23.291	60.796	16.086	12.159	30.423	22.326	40.438	47.953	30.423	55.463	30.423	54.233	49.475	56.536	70.688	16.612	51.587	25.239	19.158	16.527	60.186	51.831	33.374
17.196	34.333	31.746	54.377	63.313	17.196	12.286	58.926	52.145	35.401	22.487	13.228	82.146	14.914	38.381	72.385	60.382	56.161	13.119	28.744	17.196	23.81	17.317	19.499	22.431	60.22
26.399	59.524	67.036	60.755	22.452	26.637	34.74	20.257	28.602	34.535	30.423	34.822	57.938	11.172	46.486	16.66	50.478	52.91	48.068	51.203	26.292	19.665	25.132	47.685	33.092	44.283
16.157	57.564	41.419	35.018	42.002	26.455	42.623	10.651	49.577	41.005	46.851	33.079	53.595	18.519	34.36	18.917	59.524	76.378	72.801	56.878	24.785	37.325	17.98	14.556	20.577	26.009
12.024	28.515	47.858	78.818	34.943	23.382	66.871	18.928	65.248	44.974	35.714	32.604	55.367	31.746	19.096	39.112	41.299	14.965	58.89	37.93	35.211	11.905	43.651	76.499	36.689	14.118
37.219	20.5	66.087	14.323	53.964	45.325	46.489	24.256	40.589	22.759	27.23	17.196	91.53	18.707	15.873	54.233	62.645	24.846	30.423	43.53	31.746	54.769	15.166	9.259	55.422	21.105
17.771	73.231	82.638	60.356	51.195	18.288	20.353	34.906	76.312	27.778	26.653	10.582	54.022	47.809	51.102	21.819	24.856	31.803	22.448	58.838	31.972	71.677	27.506	19.811	46.315	44.974
19.409	47.472	12.138	59.524	52.001	64.352	36.97	17.578	33.442	41.478	27.788	26.357	89.584	13.392	49.171	43.478	25.395	22.765	27.658	28.86	34.392	44.846	8.845	24.266	24.039	13.228
25.239	18.519	19.701	46.757	57.173	29.545	14.09	48.115	80.997	55.911	43.203	37.037	39.843	44.757	76.24	73.616	51.587	44.808	47.619	29.118	21.492	23.286	41.924	36.906	21.044	62.527
17.196	10.804	41.761	43.551	36.392	15.067	31.322	45.923	31.949	31.19	18.519	23.389	36.478	23.383	17.809	16.477	19.65	23.089	19.531	17.405	26.455	21.513	39.683	29.151	33.314	51.587
26.292	56.825	104.193	63.119	50.117	14.55	33.677	17.196	39.677	15.016	18.684	37.768	30.129	78.497	10.641	57.024	22.487	11.905	37.037	65.878	38.36	40.017	30.23	27.255	17.771	56.628
24.785	34.926	55.06	44.64	60.781	32.924	13.228	50.192	28.945	30.197	17.196	60.326	96.989	42.328	63.053	46.79	51.022	23.669	43.712	50.09	26.802	12.968	13.228	9.105	32.549	52.466
35.211	19.642	46.146	49.52	68.873	46.39	35.056	11.244	46.518	27.922	24.843	45.459	93.311	63.076	42.184	14.55	22.115	67.702	39.683	29.196	26.316	31.626	23.389	30.086	38.994	21.122
31.746	65.985	73.136	54.233	20.036	14.893	35.542	20.405	27.77	28.699	11.009	18.83	53.416	30.345	46.296	12.837	38.36	48.577	28.93	24.327	14.03	48.454	17.26	21.106	19.412	22.312
31.972	14.956	83.278	76.851	53.2	41.752	24.541	26.638	37.217	27.803	51.901	49.472	55.904	84.686	56.206	45.271	51.443	27.778	19.841	47.516	54	17.786	22.614	14.08	15.722	38.021
34.392	12.523	41.005	17.339	24.553	31.262	42.76	18.519	56.545	44.974	40.71	50.791	76.302	53.683	40.894	21.164	52.092	17.658	70.403	14.918	22.256	13.228	28.995	24.492	51.295	67.24
21.492	35.6	55.619	31.098	28.887	11.271	24.318	23.23	22.087	23.198	44.785	12.698	58.235	69.781	27.778	49.921	32.288	60.796	32.247	16.747	12.428	9.259	63.882	19.642	46.009	43.431
26.455	61.174	43.831	63.686	26.768	22.435	36.828	50.508	25.233	13.418	31.746	21.164	41.278	106.146	51.381	60.555	63.514	54.439	23.327	26.926	28.05	10.582	17.196	36.955	43.96	46.052
38.36	79.983	29.285	54.249	19.841	31.704	40.68	61.533	12.159	33.672	15.873	34.336	42.026	44.701	30.65	34.533	42.344	61.825	22.375	53.692	30.866	26.764	37.744	16.584	52.91	43.78
26.802	26.373	43.936	37.037	21.891	13.913	37.529	15.901	26.971	23.354	33.482	21.764	63.922	74.779	8.514	18.944	16.281	53.205	36.647	42.312	15.901	21.653	18.519	52.089	42.006	35.542
26.316	42.72	58	25.346	21.144	44.2	31.255	27.166	27.405	43.67	17.771	48.398	64.15	71.925	57.022	28.983	55.802	22.487	38.36	21.164	14.353	28.06	27.253	80.241	37.937	48.712
14.03	14.55	48.411	33.677	32.107	36.611	19.642	19.356	42.823	23.05	43.651	27.692	24.263	10.291	47.334	55.718	53.861	27.407	51.262	51.76	30.866	19.896	37.805	27.778	54.559	26.117
54	38.458	65.356	36.322	62.978	51.322	27.205	16.836	45.418	19.648	41.828	18.519	55.81	66.4	47.331	14.55	38.483	44.596	45.831	31.687	16.566	38.341	24.156	41.208	15.172	69.792
22.256	62.605	26.953	27.455	41.07	50.776	15.873	39.906	49.635	16.368	42.556	20.419	61.801	17.42	16.116	21.106	53.84	34.292	14.01	26.535	34.727	17.833	31.984	12.164	20.813	46.466
12.428	59.409	43.025	48.521	41.005	14.339	19.572	26.239	38.35	27.217	22.565	18.519	14.438	21.677	77.825	30.696	16.824	44.529	38.36	59.407	14.427	45.4	20.374	21.235	38.905	53.632
28.05	41.199	84.595	73.388	15.983	49.572	11.475	26.125	30.239	23.008	43.422	63.867	40.832	105.775	47.619	13.228	58.738	18.519	58.685	16.075	22.606	14.535	11.905	17.771	25.711	66.654
30.866	63.492	91.564	83.082	42.139	19.143	17.775	22.448	42.188	42.328	55.644	36.448	32.322	32.67	35.494	60.847	63.567	64.174	23.81	27.273	16.336	43.402	59.894	42.328	34.316	75.352
15.901	76.177	14.819	80.555	35.102	46.296	17.332	15.255	63.095	36.789	50.262	30.169	38.912	36.558	59.772	19.998	52.108	46.676	57.395	9.732	10.001	16.836	37.642	17.418	37.413	40.929
14.353	44.974	72.657	22.487	46.73	25.132	41.843	31.103	16.042	39.122	44.273	40.166	72.955	86.833	35.166	48.67	22.229	38.793	15.902	29.763	41.412	33.739	9.353	24.86	25.527	62.053
30.866	103.011	47.619	56.11	29.101	11.073	30.855	19.096	15.873	18.246	28.462	71.732	67.46	30.423	48.699	22.253	46.817	45.584	46.673	40.028	46.523	34.906	25.879	25.706	18.917	11.834
16.566	82.011	24.587	23.353	39.467	11.905	27.796	35.79	12.577	24.219	9.787	53.821	73.062	57.858	44.215	9.421	49.727	13.228	56.385	17.196	16.336	18.717	23.624	18.519	56.239	47.113
34.727	82.014	77.046	54.807	26.102	19.751	25.879	17.905	33.577	53.463	27.778	27.124	87.016	69.337	28.031	31.809	47.475	66.919	21.063	46.135	17.161	42.645	9.353	46.525	59.111	38.494
14.427	60.154	61.415	56.897	45.831	17.81	25.682	21.819	23.346	10.067	45.785	48.418	74.237	70.529	47.251	33.974	55.481	51.319	47.708	28.641	25.934	36.843	26.368	32.236	39.683	26.196
22.606	38.665	56.761	11.905	29.612	11.905	33.617	19.087	40.097	27.22	38.884	38.36	29.817	60.099	92.633	35.416	68.988	13.54	18.15	47.348	39.048	37.861	30.423	20.666	10.91	56.959
16.336	46.71	60.065	49.705	34.392	28.958	21.097	18.488	13.388	24.692	36.817	14.55	47.702	74.304	33.334	43.047	31.746	62.4	23.744	51.845	23.551	19.291	19.939	24.746	33.253	32.573
10.001	65.213	60.153	44.762	9.064	27.596	32.165	39.898	43.651	17.046	16.788	37.864	59.063	26.83	26.21	11.905	61.099	49.226	23.81	23.974	45.883	37.037	37.257	31.746	63.492	32.39
41.412	78.508	21.495	53.947	45.549	30.423	16.048	12.707	26.189	48.03	20.638	47.434	73.056	61.808	52.129	34.392	29.322	19.596	26.627	39.559	9.353	18.881	29.742	27.701	58.694	42.732
46.523	46.296	17.132	17.196	19.841	85.979	33.693	33.88	29.792	15.873	39.784	32.083	71.587	52.846	43.241	63.426	66.817	51.871	17.196	33.136	11.162	37.799	13.714	14.609	40.326	38.36
16.336	52.91	78.042	60.889	22.621	31.746	48.637	54.233	44.316	43.812	22.487	31.353	61.406	60.393	17.164	55.332	67.404	12.479	18.776	28.631	15.722	34.235	14.55	33.38	23.286	40.579
17.161	52.579	65.473	65.923	29.611	15.901	15.351	11.224	31.832	24.117	19.386	50.909	54.302	48.511	48.415	51.617	29.888	84.179	70.787	13.025	20.124	28.803	24.055	36.301	49.638	13.228
25.934	11.905	73.627	55.196	45.658	14.55	41.924	47.006	27.803	47.341	25.773	35.906	62.493	45.305	23.81	58.201	14.143	56.269	50.443	61.938	21.509	43.65	12.552	26.83	33.281	34.392
39.048	28.884	55.184	13.177	25.974	39.604	43.856	36.677	29.734	14.55	43.522	60.469	47.791	26.22	38.823	45.194	19.623	71.927	58.389	26.886	32.458	16.744	32.9	21.164	44.659	54.233
23.551	51.439	32.759	45.765	31.379	13.31	16.285	23.81	37.291	22.087	59.003	44.501	76.054	19.748	34.65	30.336	14.281	57.21	23.678	51.69	21.513	14.643	29.219	12.475	23.383	44.802
45.883	64.903	49.383	55.171	14.55	40.693	46.315	42.849	40.885	33.727	33.339	67.64	73.011	19.462	81.009	16.22	60.183	32.961	32.496	28.664	24.318	30.948	11.905	38.468	27.778	26.25
9.353	11.787	52.91	54.574	23.596	14.412	49.36	49.603	32.643	26.455	28.908	37.321	60.053	24.318	42.266	59.524	46.275	30.24	17.224	30.423	6.614	11.905	16.52	32.189	33.144	42.606
11.162	45.873	29.93	47.351	14.012	22.487	31.746	12.869	47.938	37.037	41.709	8.139	68.98	95.753	37.037	55.964	65.945	11.475	19.642	25.889	26.221	33.442	26.42	16.836	58.913	32.549
15.722	52.464	55.666	39.646	31.801	55.302	19.765	19.253	36.721	27.778	35.282	43.651	56.585	71.219	65.196	38.36	51.587	31.746	54.718	48.354	26.595	15.661	31.746	47.477	11.838	22.041
20.124	99.757	88.65	69.81	30.493	48.428	25.597	17.16	65.095	31.368	61.301	36.297	78.898	51.587	27.585	43.519	46.436	47.619	22.487	40.564	13.616	27.374	8.925	49.946	48.404	37.037
21.509	50.796	29.101	48.942	9.259	23.264	22.244	23.355	14.808	45.891	41.005	46.309	106.395	86.258	42.09	19.281	17.571	29.93	38.194	34.885	22.665	61.744	25.937	13.228	68.031	38.912
32.458	68.405	57.297	43.806	37.533	11.664	48.566	22.448	21.562	22.296	29.585	13.155	65.058	96.285	47.98	11.241	58.474	35.235	15.736	74.281	26.444	58.623	12.258	18.519	72.376	24.968
21.513	53.431	47.217	65.89	22.487	57.267	21.888	11.834	33.625	18.608	30.358	50.691	32.965	39.022	21.01	35.095	52.443	14.55	52.104	50.945	8.877	36.984	42.487	39.149	15.601	60.023
24.318	14.329	60.796	54.233	15.873	29.16	63.006	47.392	22.063	24.207	30.986	34.4	76.697	80.901	56.75	41.83	13.228	56.878	13.095	26.455	43.474	37.492	20.842	21.654	32.294	66.138
6.614	52.108	56.791	45.095	49.652	36.184	22.487	72.476	27.942	32.754	24.779	23.442	33.531	69.298	32.495	37.884	37.208	24.58	34.098	15.482	19.58	15.382	15.099	39.058	26.087	53.144
26.221	59.703	27.952	51.208	15.144	42.894	20.266	36.652	35.714	13.228	40.884	22.321	92.284	76.322	7.937	76.141	60.02	21.083	14.55	33.192	36.598	20.326	21.164	43.474	14.973	12.595
17.196	76.939	38.744	60.001	52.906	28.255	41.895	17.811	34.915	17.667	55.771	54.437	78.643	20.553	53.591	27.326	40.711	42.354	47.613	38.688	19.723	27.56	56.113	23.691	25.846	44.988
26.595	59.801	76.9	28.813	35.714	49.152	26.455	36.478	22.178	48.126	31.532	27.77	86.953	46.296	51.27	24.066	39.48	30.995	23.626	25.281	23.724	23.977	22.56	36.478	17.75	56.103
13.616	100.48	75.157	44.974	98.549	40.974	23.376	21.164	41.085	11.905	23.634	35.846	79.278	55.556	23.132	27.778	44.589	50.265	66.259	17.196	9.89	86.375	27.762	68.938	16.255	43.206
22.665	26.065	69.101	50.622	7.937	50.55	17.196	37.802	34.607	36.906	42.317	20.701	20.229	27.831	42.716	28.169	39.065	19.717	20.661	31.746	17.771	11.911	34.663	69.802	21.101	18.612

26.444	79	68.292	78.092	35.995	19.793	50.898	14.965	35.324	39.759	39.683	14.377	55.514	39.465	19.966	43.651	53.019	15.944	50.292	49.572	21.513	32.179	10.022	14.55	20.168	42.372
8.877	28.886	102.265	66.784	40.585	31.886	42.1	23.422	22.24	46.161	31.746	37.86	48.16	86.342	45.089	75.328	60.796	16.57	58.391	54.034	28.455	26.455	11.787	46.336	50.508	68.279
43.474	20.313	23.333	48.499	45.831	17.771	9.023	9.25	11.757	65.261	21.308	44.754	26.868	59.025	46.307	40.051	43.651	44.316	32.857	52.943	20.577	26.455	39.745	27.62	68.811	39.71
19.58	58.836	62.527	29.2	69.584	50.615	24.965	35.171	36.845	25.262	21.682	43.04	43.96	28.765	59.078	42.547	24.272	57.98	25.005	15.224	16.495	23.81	14.944	23.327	29.212	47.619
36.598	57.562	10.477	40.732	28.403	19.292	11.424	17.662	39.318	25.991	41.978	31.23	30.872	64.848	52.926	58.201	44.459	27.62	21.298	17.196	10.479	32.418	11.905	23.925	43.504	37.037
19.723	67.813	43.494	60.907	57.077	18.111	13.228	38.505	41.005	19.504	51.888	32.736	31.796	13.228	11.013	16.157	26.089	20.353	21.759	40.176	41.005	24.282	31.905	25.459	36.253	8.484
23.724	27.992	95.896	34.2	35.714	29.481	19.801	34.515	11.905	41.005	83.812	45.25	13.349	63.615	43.78	51.096	55.556	54.861	21.164	33.367	15.385	26.455	18.41	10.582	47.734	26.953
9.89	62.355	62.169	44.974	41.005	21.97	20.138	22.487	48.877	26.455	27.265	9.259	71.858	36.46	23.61	46.296	28.884	22.495	34.352	11.187	13.228	23.157	21.467	20.157	69.166	22.407
17.771	44.734	51.106	38.095	27.124	51.762	27.231	57.851	44.59	52.546	23.81	19.467	24.849	38.36	57.286	24.184	25.799	28.968	21.182	17.789	30.205	8.29	15.901	26.352	41.478	25.864
21.513	84.49	63.347	43.96	32.086	21.264	21.164	32.29	44.621	44.974	24.232	36.593	37.419	68.238	45.596	44.266	17.596	29.955	60.105	28.843	27.967	22.402	13.228	8.194	55.185	53.226
28.455	19.575	10.582	49.712	39.781	25.396	11.905	15.873	41.261	18.519	37.562	15.673	43.284	61.173	16.9	8.384	69.269	10.582	52.569	22.68	29.361	11.992	14.632	34.059	31.044	14.414
20.577	12.666	81.979	54.147	34.392	23.383	45.406	24.653	37.878	36.761	24.592	19.25	19.642	51.443	25.942	41.005	52.91	15.42	13.601	48.869	18.519	26.189	40.598	58.413	41.753	24.54
16.495	62.756	78.955	53.576	22.871	30.678	16.891	7.937	26.979	25.774	53.314	21.917	44.089	64.548	26.455	26.103	68.983	19.841	24.555	25.575	17.118	13.177	27.567	17.005	17.549	39.343
10.479	34.709	41.711	66.688	10.582	66.696	13.753	30.423	41.154	27.227	17.946	44.352	77.465	61.789	18.083	18.151	34.48	47.912	20.958	45.597	18.08	17.642	24.229	24.911	42.373	24.89
41.005	49.042	69.734	72.259	20.509	57.731	10.628	50.782	43.78	41.075	39.136	49.622	44.52	76.357	41.154	36.068	66.251	35.041	57.398	33.16	28.45	44.29	24.318	24.878	38.619	39.167
15.385	12.715	26.455	50.979	12.558	18.697	20.362	20.519	45.145	31.194	26.463	40.793	9.592	53.759	58.997	64.292	48.718	24.853	27.243	29.2	17.295	11.871	15.873	16.045	42.292	36.978
13.228	60.939	59.434	19.559	31.773	39.58	12.393	18.707	23.105	35.714	15.717	26.934	86.258	49.367	25.449	15.432	68.506	56.645	19.864	25.836	25.018	35.542	22.825	60.498	29.539	45.848
30.205	44.291	73.339	19.465	40.285	32.47	16.67	38.052	21.164	16.667	31.704	43.873	37.413	23.81	53.853	15.056	53.315	55.439	73.025	36.16	21.292	16.76	32.956	45.982	38.36	66.987
27.967	74.862	76.879	52.946	27.124	13.228	39.595	34.4	45.313	14.583	32.961	44.75	59.41	53.652	47.702	36.253	42.527	42.328	40.201	51.216	18.524	22.448	40.701	59.073	54.737	52.22
29.361	68.059	68.72	56.941	44.284	18.519	26.798	7.523	60.72	7.937	30.65	47.989	65.094	21.164	55.849	44.795	35.295	34.722	41.694	23.99	33.863	13.006	11.905	43.651	50.477	30.762
18.519	47.461	71.46	66.138	36.896	46.815	18.117	23.81	24.711	48.878	10.651	66.413	92.888	84.004	27.241	24.318	35.714	25.062	37.404	46.296	13.578	13.228	14.626	30.866	49.705	66.138
17.118	62.348	45.318	72.27	34.329	11.899	43.496	9.247	36.478	22.033	26.238	50.359	57.411	65.16	9.259	21.164	26.013	46.444	51.483	17.479	18.699	40.108	12.128	15.231	34.392	46.02
18.08	66.99	85.739	50.606	62.093	69.589	20.905	45.946	30.464	41.005	32.539	16.393	58.334	30.862	38.096	49.906	28.994	57.052	65.293	40.529	33.069	28.468	49.195	13.155	63.949	30.483
28.45	65.875	51.16	43.963	26.756	40.219	26.379	30.423	57.333	27.407	19.323	21.674	99.987	100.625	13.532	62.892	57.996	29.375	48.258	31.141	21.301	31.94	23.383	23.489	35.542	56.671
17.295	48.669	28.035	46.614	20.094	44.756	49.217	9.002	56.638	21.164	15.049	51.291	53.812	36.264	63.591	36.807	61.937	30.539	42.254	63.071	21.379	27.124	13.327	39.882	30.249	42.328
25.018	12.769	86.379	68.624	29.008	20.997	35.977	22.962	55.617	18.519	38.074	42.273	49.572	63.492	50.265	19.372	30.124	60.969	63.183	37.572	29.345	34.03	27.201	31.744	32.631	57.701
21.292	44.149	63.502	36.478	65.546	24.732	15.267	29.285	25.706	27.4	21.511	49.912	30.358	18.169	26.065	14.722	73.947	49.646	26.805	17.857	15.77	15.351	16.654	32.966	55.415	22.487
18.524	47.754	9.07	61.817	61.505	40.803	37.853	36.935	31.356	25.254	62.606	50.48	26.535	74.7	7.183	51.584	50.265	61.722	32.334	25.132	22.368	27.989	14.138	11.905	24.972	28.376
33.863	78.458	90.673	60.416	27.187	39.005	31.245	18.519	43.732	25.674	26.366	48.901	31.704	35.476	20.939	32.908	15.375	30.662	17.771	48.906	27.104	21.948	26.35	21.696	34.392	25.132
13.578	78.261	55.556	55.453	25.859	48.515	38.039	49.858	47.594	20.377	25.211	17.196	67.929	58.823	27.082	47.619	41.154	46.296	51.611	21.164	11.905	10.128	14.199	33.069	37.008	48.694
18.699	45.258	81.573	33.069	28.847	28.819	27.447	13.46	20.905	23.354	33.672	45.939	36.222	68.695	43.96	38.304	24.608	33.98	20.121	55.464	15.845	22.487	15.472	20.577	56.895	12.553
33.069	66.042	54.206	49.966	37.182	12.509	41.919	12.628	17.078	11.905	19.973	53.085	55.676	10.964	10.096	23.157	22.943	23.708	60.523	18.346	38.18	8.493	19.841	52.079	67.524	17.727
21.301	18.519	72.02	68.783	32.539	25.132	19.737	38.738	39.532	42.328	27.778	51.049	28.91	57.967	15.161	59.159	47.404	54.233	21.089	47.619	18.519	15.736	18.553	52.376	31.566	55.135
21.379	69.763	29.405	58.412	54.835	22.545	34.392	17.576	29.101	19.055	18.34	44.03	54.864	29.85	23.568	16.836	27.757	60.847	55.33	29.979	11.304	13.228	17.26	36.209	14.965	65.519
29.345	54.023	13.228	47.53	35.542	28.561	23.669	33.733	36.237	22.759	47.534	43.312	34.876	38.731	69.658	39.612	41.098	30.423	24.318	8.132	13.018	31.362	29.93	30.127	37.522	18.519
15.77	45.194	72.098	66.013	36.478	23.383	23.81	36.161	47.89	34.803	30.866	21.928	8.384	68.378	14.119	28.995	21.164	38.608	56.513	42.596	17.196	47.401	26.313	28.712	28.047	39.672
22.368	35.088	84.951	73.317	46.296	21.181	20.292	28.045	37.871	42.328	41.005	39.652	9.259	63.597	18.488	22.797	29.979	28.047	28.717	26.147	11.224	11.905	32.563	41.124	60.796	21.164
27.104	38.461	71.085	50.864	31.959	39.683	31.188	32.99	37.395	50.493	25.645	35.89	48.443	63.25	27.384	55.053	26.47	77.254	66.283	13.634	18.149	14.118	19.446	28.813	37.281	29.101
11.905	57.744	24.386	45.907	44.772	11.063	27.406	19.513	44.641	25.132	12.431	7.7	24.3	28.87	15.173	40.825	61.846	19.841	11.531	18.717	32.37	31.808	13.228	39.474	53.226	35.513
15.845	77.564	25.082	14.199	54.989	26.368	13.228	36.684	24.677	53.62	52.516	30.107	45.83	81.115	69.327	11.503	29.101	51.324	19.554	19.841	24.722	29.045	11.834	30.439	29.566	87.094
38.18	62.608	76.72	40.196	41.209	14.356	65.216	53.926	25.433	49.372	59.762	56.375	51.371	55.036	15.899	19.832	48.971	32.633	56.693	17.371	15.467	17.196	14.442	11.905	39.63	40.292
18.519	24.235	74.852	61.14	17.319	26.474	23.072	17.156	41.081	25.868	74.143	32.677	44.974	76.571	76.72	53.179	44.338	39.205	36.219	7.937	48.637	21.499	30.337	28.772	47.815	32.322
11.304	37.465	53.349	50.146	26.189	44.974	38.612	14.977	46.007	14.965	18.781	26.945	66.408	74.074	17.196	46.766	64.538	35.442	27.026	23.422	20.121	27.778	17.196	42.826	13.017	41.88
13.018	67.486	13.228	66.017	22.386	22.456	66.138	27.201	23.984	29.792	35.392	52.293	42.354	68.754	23.904	26.825	41.146	63.239	44.071	42.776	11.65	22.487	21.164	23.683	47.791	62.543
17.196	23.21	57.631	7.793	68.858	9.793	33.679	15.873	40.814	11.871	32.64	44.097	17.371	52.356	14.55	10.64	32.582	29.924	24.627	15.873	28.995	42.184	10.582	71.682	33.048	48.805
11.224	57.55	48.067	16.365	69.941	57.775	23.522	28.453	34.861	13.392	28.025	43.651	25.132	27.528	49.572	44.974	44.896	44.149	19.623	16.844	36.09	28.401	23.81	41.022	9.924	26.455
20.31	34.681	77.521	26.455	30.035	14.55	31.395	14.55	57.99	15.901	32.664	55.556	62.169	26.926	22.104	50.626	67.169	59.865	23.81	12.518	20.302	21.023	19.994	66.684	70.763	68.96
18.149	39.362	70.149	42.197	11.551	58.201	22.943	14.55	27.588	40.479	33.244	27.099	86.89	99.954	60.847	48.942	58.187	56.568	14.323	37.867	15.901	24.844	34.658	47.163	33.136	35.714
32.37	65.55	37.983	64.341	58.059	17.196	32.677	27.601	39.639	23.354	21.865	15.386	62.019	52.63	57.962	35.829	28.176	20.117	17.622	25.132	28.995	13.133	47.088	57.75	58.648	47.619
24.722	65.748	62.043	19.642	26.189	41.711	35.714	38.028	45.769	37.236	38.336	37.847	64.298	13.228	77.375	64.943	68.192	54.465	52.91	65.796	37.867	25.934	30.03	31.937	38.314	46.749
15.467	44.555	94.989	66.107	48.13	37.861	42.653	67.517	68.049	41.195	50.973	50.508	72.099	99.206	33.744	57.967	55.556	41.005	21.591	44.827	54.249	50.254	24.386	48.333	14.225	42.802
48.637	14.04	73.891	44.287	23.665	34.313	17.926	13.503	24.847	34.126	26.055	66.756	12.923	95.869	48.545	38.906	34.362	57.992	19.527	45.497	11.244	17.196	13.189	18.707	63.294	31.272
20.121	49.705	44.92	49.477	21.407	42.599	17.038	22.487	39.065	44.404	17.423	61.456	86.514	68.846	64.886	35.714	51.155	63.492	28.995	59.936	35.839	43.231	12.114	26.455	19.482	22.651
11.65	59.669	27.917	73.419	21.754	35.917	14.024	38.698	48.774	28.647	50.741	52.737	19.841	17.196	43.651	29.323	49.254	27.036	17.771	59.916	23.091	50.265	13.228	16.461	34.241	36.593
28.995	61.892	105.046	24.784	17.234	35.244	31.441	10.582	35.137	23.81	22.464	35.306	64.196	74.631	105.227	29.52	51.355	32.844	24.611	34.748	22.011	18.519	12.373	62.501	52.892	24.125
36.09	88.499	17.446	34.58	44.671	34.766	26.718	14.049	41.966	47.619	37.07	53.697	76.799	47.097	57.762	33.069	64.029	64.815	14.971	37.608	30.363	45.223	32.407	46.899	47.209	63.266
20.302	54.694	11.999	15.036	53.03	21.513	16.4	19.321	16.104	57.023	29.276	38.941	29.581	69.194	69.04	12.134	56.751	69.44	16.27	84.947	14.965	36.627	30.866	20.117	48.887	39.683
15.901	15.57	20.095	58.184	35.949	15.901	31.92	53.937	27.778	11.888	78.464	50.368	57.055	66.047	18.114	23.626	52.947	41.792	47.619	69.214	30.866	29.531	13.919	63.214	34.889	28.534
28.995	11.283	68.746	42.359	30.279	63.492	44.83	64.538	26.214	29.494	12.553	41.014	33.746	49.907	43.777	49.823	47.619	36.9	32.509	30.423	21.393	20.168	13.856	37.413	22.326	52.035
37.867	72.551	73.17	63.693	42.423	75.831	39.159	15.722	31.284	31.63	28.876	21.971	21.164	18.446	60.012	13.31	75.148	47.663	33.947	41.005	28.157	12.785	22.354	34.607	54.977	62.201
54.249	59.71	58.376	51.76	40.387	65.992	44.813	39.896	32.288	21.308	21.816	35.947	58.93	40.459	62.289	40.143	55.184	25.132	25.093	39.23	11.905	22.448	16.836	30.234	52.62	45.71
11.244	59.03	73.505	60.688	24.721	39.442	24.896	18.519	64.985	52.1	33.006	21.164	14.459	57.694	26.189	23.81	35.996	29.575	49.703	41.999	21.843	37.614	25.439	56.656	62.58	45.751
35.839	63.584	80.851	59.005	15.11	25.449	10.04	15.686	57.864	53.314	22.68	40.728	87.851	60.847	46.573	33.919	66.811	53.063	51.378	24.143	20.453	9.107	41.005	31.441	13.228	43.333
23.091	33.589	85.62	15.72	72.174	25.132	38.521	24.683	41.067	32.891	33.37	59.026	59.327	42.328	17.571	44.728	74.141	27.791	23.013	11.421	7.937	10.268	33.165	21.325	52.082	49.068
22.011	106.465	70.654	57.382	45.318	15.331	25.524	11.834	41.651	35.334	30.094	36.683	60.847	60.654	66.606	26.623	47.08	42.031	55.034	25.974	67.07	27.778	10.005	50.21	18.766	30.917
30.363	47.049	23.81	46.785	36.222	20.293	28.404	26.21	38.574	52.744	67.28	10.654	10.098	61.389	54.749	42.782	49.178	42.124	45.168	48.042	16.836	57.926	52.12	14.55	42.328	44.901
14.965	29.101	30.956	52.776	13.095	32.402	10.582	27.191	26.033	9.585	66.304	79.305	84.554	40.625	56.343	34.323	59.825	34.319	11.905	60.83	23.99	29.962	48.942	34.058	51.458	32.641
30.866	47.583	38.667	48.591	74.759	14.98	11.724	24.886	50.265	44.846	57.401	40.778	38.36	55.398	18.175	41.993	36.685	47.921	48.945	71.385	31.365	18.354	28.905	30.065	38.132	36.801
21.393	68.656	47.961	34.026	28.69	23.489	18.937	42.149	32.881	44.17	22.33	58.65	40.61	39.528	36.281	40.592	31.572	41.667	33.069	51.99	30.163	22.764	26.316	45.167	44.205	15.876
28.157	37.446	61.756	55.184	37.665	32.488	32.083	16.369	30.099	18.299	16.746	48.942	43.189	15.217	6.598	27.778	32.545	52.91	45.4	49.906	24.466	21.1	21.714	25.937	44.974	31.746
11.905	59.384	58.947	41.782	21.164	20.434	30.085	15.873	29.283	39.683	46.605	45.71	57.715	58.294	57.335	37.298	32.908	74.026	33.746	51.472	37.613	50.161	7.937	29.101	23.693	20.708

21.843	63.242	74.672	67.949	46.026	48.397	20.498	17.84	34.994	30.423	16.464	28.709	21.381	76.765	54.103	37.54	52.829	56.421	29.283	42.328	17.16	21.819	29.722	44.612	55.556	69.868
20.453	21.164	23.132	30.217	32.799	38.471	14.965	44.594	23.585	20.577	53.638	55.967	74.131	40.164	42.09	32.629	10.212	49.341	27.094	54.378	23.082	21.032	27.807	56.878	18.519	43.275
7.937	62.84	79.575	82.946	31.309	43.022	26.535	24.862	38.877	62.279	20.577	33.069	77.615	19.45	19.993	21.434	22.187	68.582	76.11	76.09	28.995	25.669	13.018	43.651		
67.07	96.354	74.238	19.093	43.638	12.645	33.987	53.163	30.266	33.666	28.825	35.334	54.233	63.095	25.132	54.824	30.063	42.328	56.547	63.662	24.263	28.397	31.249	23.132		
16.836	22.003	62.725	32.534	20.149	55.556	55.208	58.093	35.762	49.789	40.529	73.097	61.564	63.013	25.132	25.394	51.587	31.348	25.464	34.017	11.79	39.14	14.159	19.816		
23.99	60.587	27.251	59.75	23.676	21.768	23.682	42.658	65.399	37.334	38.291	24.526	71.429	63.317	85.994	43.557	30.015	74.6	58.457	27.61	13.609	28.73	35.145	53.314		
31.365	45.767	63.453	40.979	16.125	54.769	16.62	33.672	42.637	38.36	31.746	43.651	31.905	66.138	55.556	33.376	21.815	12.913	23.372	45.748	14.572	9.948	21.164	44.951		
30.163	40.216	58.933	55.989	26.189	21.677	15.919	18.061	57.655	24.211	27.861	58.953	39.75	11.228	44.773	48.661	78.416	48.942	39.17	66.729	52.49	9.181	37.289	41.359		
24.466	25.429	54.275	23.596	31.687	16.788	11.987	41.005	57.426	44.048	23.316	44.974	54.909	44.896	50.508	43.336	18.151	35.91	24.709	39.051	11.952	23.116	46.688	48.34		
37.613	49.343	34.371	34.728	38.309	24.318	15.386	45.69	14.92	37.037	81.087	32.288	64.371	63.716	12.189	42.036	28.759	68.812	37.158	38.27	11.905	12.428	30.118	44.431		
17.16	69.425	27.376	43.984	46.296	30.022	11.905	46.103	22.858	41.378	32.407	43.923	65.221	59.409	18.512	47.619	60.263	33.672	70.803	80.32	11.905	28.446	11.905	16.317		
23.082	79.265	58.434	18.363	17.005	25.132	27.163	22.43	28.995	46.361	33.482	24.387	40.822	16.107	15.873	57.415	29.423	75.229	56.244	51.722	29.914	18.511	15.873	17.219		
28.995	21.403	39.284	17.196	29.101	52.558	21.164	28.803	11.905	37.844	38.88	12.531	76.8	29.528	48.912	52.156	45.328	48.445	22.015	15.077	21.672	53.749	13.615	34.392		
24.263	30.015	42.578	22.298	34.03	22.857	17.005	27.807	26.279	8.617	23.473	32.436	64.236	52.632	37.037	30.423	22.765	26.04	18.012	60.749	25.414	24.793	26.351	36.183		
11.79	77.342	55.474	17.346	63.492	39.798	14.328	36.648	17.014	27.15	35.949	56.878	51.303	40.062	43.712	25.369	49.831	30.411	24.256	51.593	21.222	14.148	32.556	28.678		
13.609	52.825	68.491	31.71	31.801	35.633	28.99	31.737	38.485	56.628	40.767	23.97	62.267	46.083	50.281	37.746	57.467	10.774	40.219	40.09	11.905	13.228	17.703	22.058		
14.572	72.833	11.905	9.754	9.911	48.637	52.791	10.51	73.635	37.061	49.208	58.841	10.268	13.608	71.27	20.774	55.73	41.94	68.234	10.388	10.582	15.979	41.709	39.887		
52.49	48.22	51.818	12.79	48.05	22	25.254	15.382	48.513	44.323	20.842	43.041	25.132	66.086	27.392	21.164	32.115	46.296	55.427	34.264	18.399	26.158	44.013	29.93		
11.952	24.318	55.978	25.942	33.332	30.358	13.228	72.955	49.983	26.749	43.96	38.84	28.258	17.945	32.584	22.405	25.254	59.334	69.098	46.288	22.459	22.015	21.164	28.708		
11.905	20.772	66.429	32.095	38.075	45.611	14.55	22.344	17.98	21.813	38.36	53.85	22.695	54.705	45.095	48.507	29.951	49.004	48.68	9.259	28.279	27.62	38.36	26.87		
11.905	81.187	54.233	13.578	9.361	41.464	11.905	17.298	53.188	46.974	22.605	29.966	69.352	58.112	46.15	33.497	69.384	23.746	21.678	62.577	25.969	12.615	24.682	38.074		
29.914	84.845	92.456	31.746	66.326	26.455	21.164	37.037	53.837	57.401	41.37	23.564	49.561	78.226	26.891	30.423	56.615	39.436	13.393	29.586	28.308	40.98	18.217	36.677		
21.672	32.368	29.72	9.865	20.377	28.208	42.917	14.03	45.97	34.392	47.645	68.32	27.585	62.165	54.13	11.115	63.201	60.479	17.406	62.353	13.228	45.439	19.841	28.125		
25.414	76.243	67.035	29.93	21.1	28.116	33.585	35.542	19.247	46.005	16.936	26.218	59.008	76.457	20.31	14.507	41.303	55.932	22.392	33.468	40.568	35.714	25.026	50.558		
21.222	75.761	23.817	16.825	50.051	25.132	30.109	53.121	23.755	25.989	30.696	41.396	71.058	60.221	59.524	35.161	45.382	29.029	18.433	14.965	14.55	27.124	11.905	24.672		
11.905	58.935	87.212	10.064	46.599	65.698	15.873	39.683	46.377	43.567	27.22	63.132	50.667	61.378	28.006	35.588	34.864	48.119	25.574	17.196	17.126	24.344	35.617	40.377		
10.582	55.189	60.32	10.289	21.895	26.019	12.05	39.284	51.719	38.115	67.344	51.448	44.54	95.204	10.642	36.855	59.524	68.144	10.59	53.853	39.383	17.571	10.464	28.228		
18.399	14.559	47.423	23.363	53.277	31.976	20.089	51.506	28.87	58.201	41.294	43.175	28.456	26.235	66.133	20.096	40.103	16.707	36.527	55.556	18.726	12.104	11.387	33.575		
22.459	45.831	14.55	21.904	28.007	23.732	13.919	38.36	36.361	11.401	65.668	22.41	13.228	19.191	24.212	39.414	48.661	23.148	38.461	64.815	25.12	27.515	38.872	11.695		
28.279	75.447	43.068	21.378	68.585	86.705	10.624	23.691	46.553	12.134	15.42	61.732	39.269	27.449	29.101	31.746	22.179	37.921	36.7	54.076	15.873	27.778	43.936	25.069		
25.969	67.789	88.405	20.683	28.298	48.56	14.333	30.696	43.979	37.037	21.032	23.375	44.602	69.964	66.407	13.796	54.249	46.766	36.075	26.455	25.254	20.577	28.06	35.535		
28.308	29.101	70.127	20.219	24.104	63.229	9.259	33.303	32.692	95.863	35.002	53.868	28.804	84.179	47.496	30.977	36.821	47.485	86.404	61.369	15.082	44.282	27.62	33.158		
13.228	44.018	48.125	21.296	54.434	45.606	53.287	35.609	24.389	30.299	27.094	35.542	32.556	35.026	57.791	35.45	13.129	39.288	56.56	26.885	15.716	11.919	39.983	47.142		
40.568	20.571	51.982	13.272	73.781	40.994	9.259	21.301	17.862	27.43	19.642	55.276	15.993	51.804	32.82	13.976	20.855	46.296	30.297	97.275	17.196	22.402	34.392	40.139		
14.55	60.079	51.443	28.988	64.431	27.573	12.785	26.68	16.223	59.223	35.17	42.528	15.873	29.425	51.982	24.553	61.735	15.126	33.929	71.488	29.1	10.14	37.037	15.973		
17.126	84.175	32.63	27.637	23.878	22.487	36.521	28.47	11.905	41.709	21.587	30.779	25.254	50.698	43.852	35.714	22.041	22.125	21.997	15.445	15.655	25.875	29.71	24.252		
39.383	53.538	43.259	50.875	46.296	22.123	24.802	31.746	9.717	21.9	28.278	41.412	84.656	26.474	22.459	65.247	40.793	30.107	55.071	63.685	19.172	23.81	17.922	46.103		
18.726	49.46	58.302	28.78	19.642	56.874	10.209	28.728	31.397	41.514	38.36	40.8	99.442	55.212	23.261	35.985	22.107	19.089	35.995	52.247	11.871	19.371	13.689	15.631		
25.12	58.78	33.888	19.075	33.604	20.612	24.697	13.228	18.519	71.759	21.304	55.236	43.025	25.711	50.508	36.985	42.09	23.776	20.293	56.916	16.554	34.378	34.607	47.619		
15.873	60.486	12.014	34.607	42.097	78.55	31.941	10.289	21.819	34.689	10.654	41.704	58.757	54.248	11.224	19.782	75.53	19.994	10.289	22.003	30.464	15.873	36.606	19.632		
25.254	26.459	13.407	18.699	23.223	58.201	33.935	44.601	42.246	55.184	78.042	60.796	16.529	43.402	80.547	44.624	36.372	63.492	33.614	27.778	27.075	19.512	34.769	25.685		
15.082	74.116	11.905	50.265	29.101	13.27	34.303	33.244	14.71	42.35	38.464	39.683	32.218	41.005	32.851	41.364	26.164	63.354	9.353	52.617	26.309	11.052	18.763	17.69		
15.716	87.017	12.777	27.778	44.355	48.979	31.746	17.454	21.709	57.133	39.986	74.582	32.865	54.559	26.357	43.651	51.608	13.095	19.994	48.661	27.629	38.36	19.841	59.969		
17.196	7.784	15.873	27.124	9.787	11.092	17.196	21.283	31.801	11.905	43.353	37.157	67.46	79.12	44.741	43.61	42.616	67.605	12.117	79.126	9.259	39.872	41.04	34.32		
29.1	95.005	19.841	12.425	67.955	33.276	7.937	51.597	22.125	21.317	27.273	34.558	70.139	79.395	13.629	11.905	67.115	69.86	52.961	28.726	31.89	17.558	13.578	28.458		
15.655	32.188	19.412	25.339	16.223	31.767	9.259	29.001	17.196	24.861	22.194	24.004	29.476	68.816	46.612	25.102	48.318	19.098	45.117	28.679	20.711	37.597	55.471	48.185		
19.172	74.074	16.317	23.529	21.301	25.043	24.689	31.271	14.215	19.841	35.581	42.26	20.08	40.765	93.216	27.778	45.938	48.942	55.556	27.35	24.968	10.392	31.746	21.311		
11.871	26.455	23.433	29.984	31.021	22.312	16.554	43.352	22.402	18.878	67.533	46.457	74.638	19.381	43.025	70.652	54.073	31.139	67.46	24.52	16.584	14.17	27.778	9.907		
16.554	52.415	20.577	11.871	14.965	34.891	21.301	36.179	24.931	46.066	57.173	50.2	60.923	73.487	17.16	43.651	40.359	45.27	37.166	44.869	11.905	20.446	37.037	21.526		
30.464	43.98	23.81	14.55	31.378	52.076	29.15	31.691	38.289	56.501	14.011	33.577	72.732	41.074	36.281	34.607	68.729	68.992	31.746	54.899	24.886	15.205	63.299	22.459		
27.075	69.664	35.113	18.134	28.581	15.606	21.298	39.169	32.219	53.59	40.552	58.087	24.831	57.157	51.359	16.474	40.75	56.671	26.068	28.761	27.342	18.338	13.615	25.282		
26.309	55.755	32.095	10.538	18.122	20.403	14.513	34.331	49.786	35.401	34.685	62.606	46.487	71.65	47.619	29.101	69.86	24.255	51.523	71.286	8.968	12.428	53.889	21.164		
27.629	61.782	23.424	35.231	72.973	23.062	23.81	39.638	38.529	34.942	47.357	38.201	45.833	49.86	64.761	64.498	38.655	43.497	23.383	38.124	36.274	36.097	22.459	37.037		
9.259	48.044	14.134	29.661	54.605	29.758	31.746	30.341	39.175	57.195	21.18	43.139	38.625	15.392	48.942	28.717	54.248	26.189	27.006	14.223	11.113	44.521	9.356	72.572		
31.89	46.157	23.417	10.045	42.328	21.375	48.891	40.269	13.095	73.408	30.765	44.918	50.785	50.999	46.175	13.228	74.171	14.82	22.487	39.96	21.997	19.642	56.878	23.81		
20.711	70.279	26.441	22.035	13.228	11.905	30.811	131.332	35.567	38.379	23.205	38.979	71.382	77.362	64.033	36.811	55.353	41.736	56.784	52.378	43.13	43.16	12.199	60.106		
24.968	79.346	18.524	19.243	24.333	46.766	22.709	33.582	21.891	45.775	36.047	46.409	20.971	55.556	72.751	13.575	55.968	41.005	21.164	28.06	20.03	10.879	21.181	36.008		
33.863	22.448	28.959	9.474	26.571	50.234	35.457	39.196	24.083	19.841	52.165	46.518	17.196	65.278	16.105	45.478	49.681	42.865	39.652	40.109	24.082	18.835	13.856	26.166		
16.584	79.84	30.423	24.467	47.126	49.982	23.81	43.38	49.646	61.269	26.052	11.752	21.747	79.592	76.229	38.109	59.468	14.965	73.91	40.228	15.976	21.089	26.455	44.974		
11.905	72.195	43.364	21.967	20.189	33.278	23.052	22.487	16.514	39.367	29.527	27.778	23.81	59.379	59.524	23.665	45.593	29.566	22.448	47.644	26.189	25.102	21.513	30.552		
24.886	21.164	14.55	46.489	38.748	13.688	19.724	17.196	35.941	51.443	20.085	12.475	39.683	18.288	42.411	22.256	19.642	39.08	36.978	44.974	11.905	21.989	8.763	36.84		
27.342	58.912	62.667	24.958	62.846	49.37	21.447	57.755	60.902	18.707	33.069	53.354	55.388	25.254	53.872	32.432	23.488	43.427	58.65	25.254	9.461	15.062	36.417	33.485		
8.968	79.878	21.164	20.453	49.162	23.42	32.016	34.387	26.796	53.314	33.291	29.93	10.919	21.606	26.012	41.005	34.179	48.353	49.573	13.996	23.81	12.116	18.519	27.323		
36.274	59.524	21.137	21.164	38.147	21.615	34.334	18.384	52.298	47.619	46.25	35.181	21.179	46.296	11.453	49.168	24.588	50.232	68.493	52.6	25.934	22.448	7.937	19.665		
11.113	45.831	12.918	48.523	33.444	36.257	37.067	41.249	14.978	27.627	26.938	45.977	13.841	69.771	60.847	31.586	63.402	21.433	48.771	21.175	12.425	37.861	23.892	27.901		
21.997	38.88	13.857	19.841	45.831	18.201	24.577	44.363	34.413	15.789	59.032	65.085	51.843	43.323	16.836	41.005	56.766	63.721	25.473	58.024	17.196	21.164	19.607	16.529		
43.13	34.999	29.954	26.044	25.132	45.831	44.815	25.967	26.419	57.558	28.556	65.9	66.138	66.021	34.556	19.944	46.063	21.164	41.613	53.314	22.715	37.729	10.128	15.784		
20.03	33.947	21.298	8.949	35.714	10.289	12.425	42.311	41.479	25.328	31.746	48.821	44.884	50.508	42.091	17.601	53.749	24.878	43.371	46.296	24.186	41.784	37.863	38.36		
24.082	51.955	51.486	20.212	31.746	29.345	21.067	25.434	34.154	30.971	49.249	43.807	60.765	37.864	16.464	23.81	46.622	64.003	42.666	20.257	40.37	35.622	13.228	46.729		
15.976	31.827	25.112	44.767	23.762	64.298	23.972	21.164	66.281	26.36	25.236	38.742	87.325	59.553	73.908	11.905	50.672	47.087	64.815	22.139	32.186	29.498	42.805	26.455		
19.651	62.169	24.838	22.362	36.301	24.107	19.841	21.932	24.728	44.405	44.974	50.739	61.347	26.455	40.418	22.074	39.378	35.23	22.015	27.491	20.028	38.041	20.553	49.828		

26.189	59.036	14.03	14.442	18.519	70.08	31.145	33.82	41.195	60.796	45.536	36.594	76.698	79.365	43.188	43.634	70.232	46.466	32.821	43.651	26.092	20.376	14.55	56.287		
11.905	48.748	15.873	34.392	16.548	31.87	37.037	40.366	42.328	29.101	26.455	48.942	44.376	25.132	17.635	33.482	83.755	64.031	52.744	50.617	20.577	35.683	39.787	26.945		
9.461	13.797	26.139	19.841	52.021	37.108	8.932	13.022	32.195	76.44	36.161	35.548	29.758	11.787	38.703	13.388	68.065	48.261	38.36	43.401	11.129	30.5	12.833	33.439		
23.81	45.869	33.069	35.915	33.529	32.799	20.384	39.5	49.778	52.386	25.175	62.169	20.094	47.308	11.231	44.599	49.2	42.328	31.123	54.233	24.318	19.222	12.159	29.133		
25.934	70.149	12.854	29.395	42.131	79.921	15.796	27.507	13.593	25.132	47.619	39.562	37.796	61.65	54.893	12.912	52.91	64.364	39.028	30.837	35.194	39.199	17.58	44.269		
12.425	46.737	15.389	7.753	12.189	46.737	35.155	29.983	25.132	31.564	34.028	49.471	33.026	63.5	37.567	29.452	40.492	33.255	57.235	12.484	24.977	40.828	67.46	61.406		
17.196	25.064	41.288	25.132	11.097	81.919	23.068	33.4	46.431	43.247	48.068	31.7	56.671	43.391	21.164	10.284	39.774	21.989	67.763	43.33	21.759	18.736	19.45	50.235		
22.715	17.955	42.027	17.655	31.628	64.842	43.186	26.281	27.118	14.438	26.496	55.429	11.076	42.006	46.398	36.27	50.65	76.475	44.513	49.46	10.214	25.094	17.332	11.843		
24.186	38.837	25.881	15.592	13.891	10.582	62.715	13.228	17.9	49.342	19.077	52.217	72.625	49.928	60.792	42.619	14.225	26.455	51.906	60.508	22.448	73.566	24.317	19.255		
40.37	13.741	26.741	25.443	31.538	28.462	39.667	22.487	27.124	45.33	12.159	51.889	22.977	94.811	92.107	19.841	61.732	52.885	22.487	62.838	31.126	14.469	23.81	10.351		
32.186	16.343	44.974	14.088	29.248	27.927	15.067	51.253	24.127	23.623	17.074	30.357	64.031	25.78	52.66	34.4	51.479	29.101	56.264	52.129	10.27	31.746	22.802	31.191		
20.028	16.52	26.75	35.167	15.74	17.698	47.702	14.55	39.399	50.265	53.616	48.006	84.018	37.037	74.425	35.201	26.206	37.037	58.548	20.331	10.464	19.841	25.969	36.761		
26.092	32.432	14.55	12.497	19.841	11.905	18.519	16.263	38.121	48.785	59.989	61.754	68.983	38.437	30.127	21.164	78.273	39.699	42.606	26.406	23.383	13.018	26.677	15.128		
20.577	52.144	17.196	13.578	39.366	53.583	41.005	12.399	18.117	15.566	41.884	45.635	73.938	94.295	16.873	17.75	57.175	78.623	41.398	68.343	12.706	17.771	59.857	13.553		
24.267	39.087	12.863	21.819	18.519	21.164	11.905	44.923	21.026	61.863	39.521	59.405	45.592	66.781	11.196	25.853	47.831	49.465	19.202	61.93	9.197	17.151	35.283	12.852		
11.129	20.286	14.301	23.079	26.313	11.387	13.575	29.933	19.642	26.357	20.767	59.861	33.755	76.51	71.187	50.136	82.551	49.671	18.519	20.535	14.965	33.663	40.353	29.196		
24.318	32.891	36.978	31.077	24.721	27.43	20.169	15.985	61.921	51.469	33.939	36.847	57.821	29.812	79.764	33.965	35.337	51.587	15.536	37.413	20.117	18.519	15.901	29.023		
35.194	30.477	18.457	19.841	21.492	11.816	31.771	30.696	27.913	34.026	16.612	70.949	72.242	50.279	53.378	33.693	43.202	46.269	81.333	11.719	23.81	25.132	24.276	27.307		
24.977	69.5	15.873	27.762	66.662	43.995	16.747	51.779	17.883	11.905	27.231	22.029	72.844	25.254	71.651	30.214	50.577	57.055	40.771	59.488	15.6	13.839	50.263	20.785		
21.759	22.487	21.305	33.672	35.142	31.801	30.055	41.664	56.71	42.156	35.24	57.875	21.164	84.711	35.534	11.271	46.147	10.582	7.713	22.174	39.357	23.669	19.841	38.36		
10.214	38.36	29.101	29.101	31.236	54.206	15.117	66.516	24.913	9.014	54.213	66.138	46.474	43.105	11.987	38.879	32.458	15.402	51.71	48.354	9.239	31.878	13.839	76.72		
22.448	10.582	56.922	21.206	53.995	30.178	23.273	24.948	25.576	47.36	32.098	45.775	27.778	37.001	54.502	16.654	54.129	27.182	22.452	16.409	19.128	21.688	25.132	23.085		
12.706	23.079	32.449	13.228	15.429	33.897	41.209	30.959	31.686	38.1	29.101	12.176	22.487	27.778	39.837	54.076	69.352	17.196	15.8	65.385	11.151	12.431	24.551	41.946		
31.126	12.428	21.594	28.594	34.021	33.069	43.756	74.032	13.228	55.429	60.028	20.6	22.448	57.045	29.101	18.162	50.408	15.897	17.698	83.735	13.585	31.296	14.55	20.538		
10.27	15.873	10.537	42.042	21.915	23.81	27.196	16.965	13.228	51.587	45.966	33.883	46.402	71.717	11.397	24.521	33.069	51.587	27.65	27.084	12.159	52.14	40.597	55.179		
10.464	21.936	22.459	18.604	34.607	51.113	29.145	14.176	23.81	65.429	39.044	56.396	23.616	55.332	66.724	45.72	43.878	36.394	32.493	54.15	32.916	18.603	31.321	18.707		
23.383	17.75	29.101	44.896	41.785	41.005	41.67	26.195	31.136	61.124	27.436	50.168	71.114	52.811	33.069	25.777	17.97	43.651	55.51	38.558	38.341	14.209	46.541	26.73		
12.706	33.069	22.314	26.633	29.101	18.134	25.132	78.925	45.102	54.647	15.049	64.14	66.669	59.954	66.195	27.956	17.617	18.134	20.577	30.499	26.065	23.696	25.443	27.528		
9.197	34.392	43.347	14.461	19.481	11.387	10.582	36.599	29.285	51.587	41.005	38.765	30.55	14.159	47.503	31.976	43.62	58.797	20.954	33.387	8.87	20.5	11.838	19.298		
14.965	33.884	18.519	31.267	75.839	23.81	23.88	16.616	24.318	27.224	29.796	56.867	70.111	18.18	11.316	33.37	31.865	36.499	27.692	49.022	24.78	26.89	10.582	16.591		
20.117	30.788	15.546	22.115	28.06	29.521	48.452	20.577	50.508	27.217	58.26	43.783	17.405	49.398	75.034	42.328	31.04	51.443	15.994	37.277	51.276	13.228	81.539	30.421		
23.81	10.891	28.545	24.974	30.071	36.292	42.328	61.88	14.03	39.494	30.777	32.657	18.113	59.22	27.201	35.789	48.017	25.773	20.577	32.233	20.668	22.487	62.052	19.874		
15.6	31.521	24.675	44.168	8.035	18.986	13.228	39.709	15.901	36.09	31.348	32.542	32.685	82.149	57.829	16.734	22.229	59.424	25.96	34.392	16.244	15.873	11.982	17.196		
39.357	37.037	10.295	23.974	45.193	43.395	14.799	15.497	9.258	37.846	30.552	8.13	80.672	44.973	82.011	26.142	38.82	31.746	15.648	31.613	10.357	29.12	11.905	18.08		
9.239	13.919	57.019	28.071	44.442	33.672	48.687	27.196	22.691	32.093	30.178	45.98	44.285	39.161	24.259	45.332	39.683	68.99	34.392	63.907	14.356	12.289	37.86	25.378		
19.128	29.361	26.455	33.069	27.811	9.522	39.552	29.251	38.231	7.951	48.237	32.322	33.71	20.461	35.448	41.848	52.525	76.72	31.746	56.863	16.188	39.638	23.81	26.626		
11.151	14.538	24.532	16.496	18.2	21.394	16.064	30.803	37.63	11.299	34.269	28.93	35.417	53.957	85.441	37.413	27.691	54.446	24.044	45.538	12.431	27.124	13.608	19.642		
13.585	53.129	11.224	18.299	16.568	27.674	37.466	20.577	17.078	80.607	42.994	21.681	18.264	27.622	44.226	25.132	33.231	46.394	43.839	59.708	26.117	37.158	43.883	17.581		
12.159	14.696	35.168	22.487	69.471	25.837	19.445	52.162	34.659	37.789	10.057	54.079	41.843	56.385	12.457	15.722	60.646	23.81	18.608	37.913	15.901	27.778	12.428	55.322		
32.916	22.229	44.688	10.077	77.487	62.282	17.531	26.455	21.635	31.656	22.487	50.508	42.488	42.328	31.721	19.647	36.253	32.877	36.063	65.512	15.076	15.166	41.313	26.471		
38.341	35.312	18.186	39.589	45.834	32.619	11.224	31.415	20.577	46.27	40.598	55.386	34.001	12.157	66.723	19.841	25.958	17.912	29.71	65.309	39.436	34.392	13.195	21.164		
26.065	53.749	70.665	53.244	41.211	42.896	30.887	32.525	48.406	22.448	34.66	35.532	35.738	42.235	36.503	30.059	49.956	38.36	51.526	31.979	12.758	22.487	27.434	17.196		
8.87	31.746	22.576	27.333	16.745	49.171	38.07	39.308	25.494	36.103	37.083	39.678	26.189	49.676	7.266	47.878	45.901	46.017	48.942	49.703	32.07	25.074	44.899	22.578		
24.78	29.248	55.786	17.16	25.608	22.754	22.877	44.415	17.227	35.493	42.061	42.613	17.427	52.91	50.737	37.037	68.582	33.132	47.991	62.574	49.572	28.581	42.589	31.746		
51.276	27.085	34.864	8.418	42.556	28.56	27.593	38.17	31.567	40.747	22.487	46.296	14.524	52.389	23.81	39.683	43.655	21.089	11.551	19.285	33.666	23.676	33.373	18.313		
20.668	25.132	23.085	27.161	59.438	25.132	22.487	31.801	21.164	40.419	29.004	20.957	47.901	42.834	66.961	42.551	41.005	59.3	17.24	71.003	23.593	23.576	20.843	37.885		
16.244	34.154	39.851	10.133	59.35	53.916	20.15	30.409	30.423	31.675	34.392	56.255	18.96	32.638	11.905	18.519	54.233	48.578	60.38	36.029	40.875	32.75	19.624	18.825		
10.357	64.302	27.124	21.96	39.893	64.313	28.04	17.707	40.476	10.897	22.487	38.454	20.345	23.373	32.542	26.606	42.284	50.716	46.678	67.636	14.792	24.782	14.55	23.141		
14.356	27.175	34.685	25.055	31.988	37.211	14.55	29.763	13.385	61.732	19.707	38.576	29.656	23.81	21.495	13.228	91.27	24.705	44.38	49.26	31.102	20.577	11.794	23.645		
16.188	25.993	34.697	21.248	19.687	50.71	35.714	29.7	20.527	49.87	27.075	42.328	30.824	50.89	27.113	30.782	32.193	58.338	33.902	69.619	12.117	27.553	13.228	41		
12.431	19.513	12.44	15.385	28.06	47.932	32.839	19.841	35.069	20.577	39.296	34.874	31.072	28.57	20.069	16.514	89.167	19.908	20.173	22.889	36.376	19.841	25.576	31.379		
26.117	30.197	10.517	9.181	42.058	23.383	35.251	39.658	26.455	29.101	38.281	44.974	29.512	42.328	12.997	29.717	51.35	46.296	23.383	46.868	18.07	21.966	14.55	32.931		
15.901	23.81	21.164	25.132	18.519	17.781	24.511	67.365	24.501	38.36	22.843	39.491	40.138	33.069	34.755	35.486	58.908	67.785	49.807	17.406	24.793	30.835	10.582	58.076		
15.076	15.663	19.841	20.473	38.22	36.623	38.33	27.264	26.455	20.077	43.787	24.207	42.405	17.196	33.069	23.258	48.413	44.842	37.513	59.375	23.383	10.582	11.686	17.958		
39.436	33.939	14.55	37.666	20.318	41.259	19.134	30.585	60.454	59.155	16.296	40.164	30.423	40.179	25.586	30.369	14.358	26.906	26.153	51.443	25.254	28.963	24.691	18.394		
12.758	38.419	23.428	18.707	22.448	32.856	14.186	30.12	47.162	37.311	46.03	45.491	14.55	30.656	15.873	31.883	60.346	44.132	68.646	52.029	25.026	39.113	27.015	30.658		
32.07	34.607	16.826	23.602	48.255	41.137	28.797	36.652	37.399	56.329	17.193	50.617	35.289	45.529	38.08	15.954	46.946	71.48	40.641	42.328	22.402	33.057	20.011	28.048		
49.572	40.783	28.995	15.897	44.837	26.189	25.399	63.073	24.022	52.044	26.898	35.019	18.645	18.519	26.726	54.101	66.606	45.597	36.478	27.692	24.856	11.905	18.72	69.5		
33.666	22.804	14.03	19.108	32.015	37.037	33.069	30.866	22.448	29.943	33.638	31.379	29.101	19.63	35.991	34.816	70.972	50.857	81.047	65.917	32.679	15.901	44.974	22.487		
23.593	24.77	34.44	19.417	27.842	22.487	13.267	30.156	13.017	30.127	56.084	47.134	38.461	25.132	36.399		43.651	55.711	27.838	29.954	27.692	26.455	43.648	38.36		
40.875	24.815	24.132	22.593	38.791	26.455	38.08	65.97	54.296	23.81	61.747	78.126	18.707	12.719	27.679		28.712	49.294	16.566	65.473	43.984	30.768	22.487	10.582		
14.792	20.498	27.611	37.102	45.056	13.57	15.297	36.131	64.522	14.438	15.321	26.023					18.583	45.432	15.873	51.865	18.363	32.736	28.275	23.079		
31.102	26.474	17.196	28.931	49.866	44.974	41.005	17.196	23.984	48.106	21.164	37.673					29.071	33.911	54.092	60.847	17.196	33.736	12.148	12.428		
12.117	47.691	26.455	11.905	35.714	26.455	34.664	17.712	79.899	43.408	17.196	38.28					53.751	24.622	15.863	54.249	22.298	16.836	37.67	15.873		
36.376	23.03	27.412	10.368	36.866	32.016	24.277	11.224	15.873	28.994	32.024	46.766					30.684	52.387	46.392	50.806	17.346	24.58	15.577	21.936		
18.07	19.729	16.844	22.234	46.392	50.774	25.833	44.334	79.531	12.979	45.541	42.652					51.255	60.87	17.196	56.12	31.71	11.905	17.17	33.069		
24.793	42.654	36.736	48.982	59.373	35.714	72.751	32.736	28.654	56.878	46.434	45.397					38.979	32.297	20.577	53.314	9.754	15.462	19.841	34.392		
23.383	9.549	18.519	14.965	38.14	61.058	37.245	31.355	10.582	49.548	10.582	47.12					62.123	57.953	26.931	53.185	12.79	17.753	19.823	33.884		
25.254	15.862	21.164	11.228	22.487	24.897	16.806	18.519	15.733	45.583	47.396	32.752					52.069	44.974	52.91	40.314	25.942	14.03	28.775	30.788		
25.026	23.008	58.076	24.537	41.79	32.611	16.836	33.482	30.775	30.959	20.577	37.123					51.561	28.347	47.05	63.602	32.095	33.721	11.636	10.891		
22.402	32.234	17.958	15.834	37.662	32.807	48.045	12.159	15.067	41.289	29.123	69.263					26.244	20.693	18.519	36.687	13.578	39.406	42.702	31.521		

24.856	28.633	18.394	40.864	39.159	45.517	35.644	42.268	13.601	48.247	43.093	34.854					69.079	33.672	17.156	47.415	31.746	47.674	23.81	37.037		
32.679	19.901	30.658	27.124	52.568	24.155	43.92	13.798	13.778	14.875	30.449	38.36					57.121	34.003	35.714	21.099	9.865	17.912	11.905	29.361		
27.692	35.2	28.048	13.228	36.243	28.314	21.653	34.494	33.219	11.161	46.207	14.409					18.264	15.336	64.719	53.314	29.93	20.299	11.63	14.538		
40.204	24.692	37.664	28.306	40.62	13.095	23.077	18.608	14.765	41.298	38.391	38.481					69.169	54.89	43.662	17.984	16.825	27.03	13.578	53.129		
24.786	45.215	33.974	21.164	30.357	31.495	21.637	18.595	16.903	40.36	19.29	36.121					44.896	49.729	19.524	46.781	10.064	26.189	31.956	14.696		
55.556	28.995	40.69	23.669	62.849	36.357	31.518	22.167	40.529	48.708	39.627	30.146					50.173	51.728	35.21	22.002	10.289	13.594	19.097	22.229		
22.488	8.081	11.244	20.913	28.83	32.651	54.249	22.589	26.455	37.828	21.164	40.539					49.591	13.228	59.276	49.051	23.363	14.03	19.871	35.312		
27.54	27.778	33.49	37.799	13.881	53.222	33.512	53.26	43.757	25.695	47.319	46.832					51.898	23.367	12.431	24.75	21.904	19.527	56.781	53.749		
47.509	18.817	10.54	49.34	16.31	36.165	8.094	50.191	35.382	74.857	36.984	63.946					52.468	40.711	62.127	26.189	21.378	22.448	17.196	31.746		
37.874	23.193	28.154	13.228	36.732	19.464	43.615	22.963	42.627	8.824	16.444	39.962					47.619	24.219	38.335	34.769	20.683	19.841	15.873	29.248		
40.522	33.192	33.672	26.053	28.042	25.999	10.582	43.519	19.394	16.829	37.037	39.683					43.444	10.522	28.647	42.211	20.219	18.519	15.681	27.085		
44.346	31.952	30.337	20.047	65.391	47.691	10.884	30.115	38.905	21.164	21.164	46.296					56.389	45.104	48.177	41.057	21.296	20.448	11.905	25.132		
53.005	23.81	12.691	30.381	39.502	15.049	20.582	28.274	17.196	55.377	29.16	23.373					37.608	48.438	51.499	28.87	13.272	17.424	26.251	34.154		
50.051	13.045	27.552	61.952	30.641	38.656	22.856	23.81	16.708	28.809	15.438	25.535					65.332	60.133	47.702	21.891	28.988	31.276	23.552	64.302		
33.274	11.31	28.803	34.646	33.004	24.573	35.876	16.527	28.27	37.57	10.887	66.226					33.756	23.81	19.841	54.717	27.637	18.926	18.902	27.175		
35.714	27.191	50.265	24.965	20.439	25.39	24.256	35.714	10.46	15.405	23.383	45.778					19.85	36.658	47.118	25.865	50.875	27.647	53.16	25.993		
7.563	22.146	14.118	24.318	19.524	57.646	24.062	30.833	56.521	38.354	10.657	48.822					17.196	22.392	71.588	39.284	19.075	22.109	24.46	30.197		
48.637	22.386	32.052	13.615	13.543	28.725	22.748	21.271	9.675	18.519	41.856	51.857					32.669	19.841	49.488	17.224	34.607	15.661	38.36	23.81		
35.593	13.228	36.097	15.873	34.884	35.347	56.615	72.248	25.132	38.249	16.862	60.531					33.492	79.254	51.587	22.448	18.699	17.156	53.764	15.663		
24.682	20.65	18.406	39.284	42.881	45.698	28.561	65.384	17.363	44.015	48.535	51.048					23.934	26.474	50.757	46.663	50.265	30.421	34.362	33.939		
23.81	25.132	25.638	51.301	16.836	43.03	14.03	55.556	49.301	71.183	20.09	66.708					55.836	26.603	69.836	39.535	27.778	26.455	34.848	38.419		
15.838	21.874	10.289	17.137	44.263	45.57	29.754	50.852	26.946	43.673	26.455	46.729					16.017	13.228	41.01	59.678	27.124	11.905	23.383	34.607		
26.455	33.755	32.271	30.952	31.129	42.859	52.404	26.455	48.748	17.196	28.143	19.728					20.577	26.455	32.367	36.141	12.425	45.212	17.339	40.783		
54.217	32.083	33.729	43.358	37.451	14.538	24.686	40.447	20.958	29.633	41.966	22.621					58.205	15.392	13.155	13.618	25.339	44.974	47.2	22.804		
57.413	31.128	24.586	26.202	36.847	30.098	11.642	13.966	13.615	15.858	62.169	46.844					17.922	33.108	34.392	43.321	23.529	22.827	15.873	24.77		
26.026	29.836	19.383	16.523	33.069	19.575	25.043	15.392	28.323	26.036	27.778	52.327					24.744	21.63	23.085	49.968	29.984	15.873	11.251	24.815		
13.552	29.101	26.43	42.57	59.861	45.561	46.436	79.42	11.424	33.008	14.413	26.942					47.938	47.954	40.845	61.098	11.871	42.899	22.462	20.498		
24.965	26.69	20.777	25.986	69.67	54.233	47.619	63.687	12.399	38.14	38.36	44.854					48.324	41.824	54.7	66.662	14.55	15.382	17.771	26.474		
41.682	59.252	37.037	20	36.631	64.758	38.529	23.082	28.745	42.626	27.699	58.882					44.974	48.25	32.37	47.935	18.134	18.519	57.448	47.691		
28.556	23.783	23.737	24.406	20.08	16.739	37.037	37.891	46.296	36.478	22.044	23.81					40.61	30.633	37.802	51.587	10.538	14.55	39.683	23.03		
37.284	25.26	13.127	41.928	47.285	39.084	56.049	54.151	33.604	62.57	24.869	37.656					20.769	30.065	49.793	39.399	35.231	29.922	17.353	19.729		
22.921	52.216	20.845	40.754	14.465	31.273	50.265	26.553	25.009	25.989	39.502	29.021					11.052	63.756	52.91	33.494	29.661	11.905	19.685	42.654		
24.295	22.639	30.464	54.266	34.925	50.101	40.848	58.565	52.046	31.746	32.655	46.747					35.236	63.127	36.005	27.525	10.045	19.45	13.22	9.549		
23.81	20.62	37.868	55.46	20.577	68.602	49.317	23.471	18.539	11.294	27.899	42.255					23.81	15.487	25.645	48.894	22.035	29.785	47.915	15.862		
44.28	17.295	33.005	33.357	29.021	37.62	47.401	42.779	42.302	60.335	29.997	52.136					53.1	52.02	23.409		19.243	15.644	18.937	32.234		
28.265	31.936	70.93	22.487	13.018	56.12	46.41	25.946	10.656	66.784	30.684	62.566					15.787	12.031	39.625		9.474	16.356	23.383	28.633		
70.56	23.382	48.626	23.332	38.515	27.223	31.117	17.196	21.339	9.736	23.489	43.585					28.417	45.327	17.408		24.467	16.499	24.511	19.901		
21.344	8.21	39.683	21.023	56.688	25.178	20.454	24.51	48.877	46.766	21.164	34.392					35.032	19.642	58.722		21.967	11.76	15.873	35.2		
24.58	74.667	59.51	38.658	28.047	42.292	25.799	30.847	19.862	44.866	55.253	32.781					30.423	63.061	22.376		46.489	25.055	25.132	24.692		
29.302	60.44	22.487	24.588	50.79	22.487	17.771	45.518	20.553	62.133	29.388	39.214					20.667	14.55	69.517		24.958	13.228	26.306	45.215		
37.067	27.106	31.746	33.989	16.609	26.455	20.689	17.729	30.866	46.691	33.98	43.588					15.951	18.519	18.519		20.453	23.777	19.091	28.995		
38.255	27.155	14.855	43.186	36.571	28.252	21.164	25.172	24.418	59.809	28.638	50.922					24.272	22.097	50.265		21.164	12.435	32.402	8.081		
48.549	32.457	44.974	19.737	93.105	53.628	17.593	33.069	21.164	39.308	16.736	72.955									48.523	27.528	28.843	27.778		
17.402	19.841	27.124	18.519	88.45	17.196	14.55	28.995	23.422	35.601	30.164	55.471									19.841	51.384	47.619	18.817		
47.136	16.688	42.579	15.873	14.55	58.578	23.81	10.891	29.101	62.086	51.915	52.94									26.044	30.333	13.612	23.193		
29.396	34.424	31.533		35.007	16.624	35.058	36.396	16.512	31.159	30.814	42.744									8.949	27.406	13.228	33.192		
36.988	35.249	39.194		17.196	39.375	42.589	30.404	18.519	36.373	38.024	70.245									20.212	28.031	13.095	31.952		
13.46	11.905	15.443		35.951	18.235	30.106	50.768	45.718	37.037	11.905	51.019									44.767	22.487	12.822	23.81		
27.778	11.8	8.375		20.284	32.572	11.092	53.006	50.938	40.612	49.131	18.833									22.362	37.995	34.392	13.045		
9.259	21.724	14.55		41.897	52.91	22.737	40.178	39.034	30.091	37.76	30.151									14.442	18.707	12.356	11.31		
21.135	23.188	24.269		24.745	27.073	25.686	54.178	21.179	40.994	31.039	48.529									34.392	30.866	41.696	27.191		
32.208	13.609	62.016		34.316	31.353	31.358	9.259	13.095	48.637	27.35	39.923									19.841	23.313	21.164	22.146		
14.792	52.477	24.167		24.763	54.511	21.836	41.288	15.161	34.423	25.65	10.284									35.915	23.669	11.924	22.386		
				59.194	51.587	25.942	21.301	15.406	26.357	31.924	67.281									7.753	15.873	27.22	20.65		
				55.21	33.672	43.651	23.798	11.727	23.438	22.319	42.328									25.132	44.928	33.908	25.132		
				27.12	10.551	15.979	40.462	29.972	39.683	30.032	42.838									17.655	17.622	34.923	21.874		
				45.066	27.596	11.905	51.896	28.464	30.278	57.121	11.203									14.088	14.906	50.051	33.755		
				14.775	28.934	43.11	27.479	11.905	51.011	22.487	46.766									35.167	17.196	14.03	31.128		
				53.175	33.069	19.807	10.057	13.608	10.077	37.037	26.455									12.497	18.519	26.644	29.836		
				65.698	37.276	36.826	18.934	15.73	57.818	23.573	48.85									13.578	17.196	37.785	29.101		
				17.833	34.112	11.268	41.55	38.198	22.216	16.548	43.666									23.079	11.63	26.817	26.69		
				65.098	30.423	31.667	13.701	56.662	24.256	18.011	24.308									31.077	18.957	25.884	59.252		
				26.348	50.044	10.057	44.641	13.095	42.509	15.873	54.233									19.841	15.607	26.888	23.783		
				33.358	62.975	21.513	12.176	42.397	38.945	44.276	39.988									27.762	27.031	18.902	25.26		
				21.66	21.164	14.03	35.773	26.289	17.812	31.803	33.69									33.672	32.743	13.228	52.216		
				29.375	38.432	24.862	19.245	9.921	24.965	39.391	22.487									29.101	23.578	26.314	22.639		
				25.107	15.873	61.328	17.721	55.914	11.905	40.539	26.474									21.206	32.777	25.768	20.62		
				16.836	51.001	44.763	35.09	9.259	26.455	52.801	52.924									13.228	23.974	70.56	17.295		
				33.069	26.455	43.54	16.061	21.164	30.707	39.641	59.15									28.594	20.393	18.347	31.936		
				32.959	23.81	51.839	42.623	39.699	48.895	18.164	38.027									42.042	21.552	36.716	23.382		
				35.94	33.058	36.927	80.109	15.901	12.428	44.974	60.71									18.604	25.757	38.915	8.21		

				27.075	16.321	29.101	20.762	28.293	56.589	42.016	62.169									44.896	15.873	29.101	74.667		
				32.083	37.913	19.414	64.009	33.458	51.349	69.693	62.196									26.633	23.354	16.609	27.106		
				60.508	38.912	48.111	62.322	30.719	50.12	21.101	49.583									14.461	11.905	52.243	27.155		
				48.942	21.164	11.905	16.688	34.091	69.354	25.132	46.652									31.267	23.655	31.488	32.457		
				42.358	29.101	68.87	15.396	21.164	68.783	26.478	51.662									22.115	12.431	50.184	19.841		
				49.616	24.498	40.839	31.191	57.46	16.107	39.435	57.637									24.974	21.513	19.642	16.688		
				27.998	74.566	32.993	47.441	42.328	40.777	23.383	53.063									44.168	8.293	25.322	34.424		
				41.575	35.787	34.244	30.16	18.623	57.055	40.039	63.213									23.974	33.757	18.225	35.249		
				38.927	21.308	24.873	52.016	31.61	32.083	10.661	49.422									28.071	43.158	32.341	11.8		
				61.059	49.102	19.305	30.724	62.169	37.037	37.216	39.49									16.496	12.114	41.005	21.724		
				14.202	80.257	39.362	10.289	35.716	37.8	20.353	65.453									18.299	30.842	28.195	23.188		
				23.626	34.392	44.157	16.822	7.773	43.651	41.851	31.663									22.487	44.974	16.744	13.609		
				75.995	28.06	26.477	45.206	22.165	19.891	12.159	61.349									39.589	23.383	18.683	52.477		
				29.713	28.059	28.995	49.939	32.432	26.958	39.486	50.934									53.244	31.746	15.357	37.664		
				53.674	19.841	8.07	43.025	42.344	47.525	15.873	69.45									27.333	67.71	23.881	33.974		
				32.167	21.164	41.414	15.873	16.963	25.788	47.52	75.004									17.16	34.323	17.196	40.69		
				35.275	43.166	63.711	37.66	21.164	53.327	29.101	17.75									8.418	37.877	32.193	11.244		
				43.793	66.347	49.572	32.227	29.93	12.44	25.877	50.265									27.161	16.566	24.435	28.154		
				34.392	28.915	33.569	26.842	21.253	26.455	31.89	38.888									21.96	17.175	57.726	33.672		
				46.035	39.532	37.823	43.28	28.813	45.349	50.159	38.164									25.055	11.572	31.362	30.337		
				26.455	18.608	30.811	22.482	26.35	39.683	12.402	50.798									21.248	11.905	49.804	27.552		
				50.303	53.521	20.988	32.214	23.102	40.919	31.746	60.546									15.385	23.843	10.128	28.803		
				48.945	17.448	30.359	20.27	14.03	46.49	85.8	13.609									9.181	16.426	43.979	50.265		
				68.09	61.548	55.153	28.158	31.746	43.852	37.107	45.095									25.132	22.487	27.71	14.118		
				54.152	47.912	27.901	18.355	40.413	63.569	15.876	47.66									20.473	25.009	6.715	32.052		
				17.16	49.71	39.058	22.487	54.704	33.888	41.32	44.393									37.666	29.445	23.925	25.638		
				43.991	16.201	23.893	24.303	28.826	21.164	18.937	39.173									18.707	11.905	40.819	10.289		
				43.756	37.146	18.519	36.93	41.247	46.919	16.996	46.898									23.602	15.901	42.652	32.271		
				39.14	44.823	10.582	37.143	26.455	45.699	13.095	56.305									15.897	44.974	16.156	33.729		
				19.4	24.4	39.965	51.859	45.141	49.702	37.037	58.485									19.108	23.213	31.825	24.586		
				26.83	27.785	40.34	38.153	51.472	23.81	32.556	57.596									19.417	14.199	23.81	19.383		
				22.983	20.4	43.615	17.196	34.714	38.36	16.069	48.577									22.593	12.968	17.394	26.43		
				33.16	50.774	18.295	24.219	35.517	34.607	24.858	44.99									28.931	29.702	32.281	37.037		
				47.735	19.232	45.877	32.539	30.696	9.948	13.025	63.492									11.905	13.228	27.395	23.737		
				48.495	26.455	21.164	15.354	19.841	34.341	19.356	46.929									10.368	36.54	10.59	20.845		
				32.875	17.196	20.718	33.296	33.048	52.444	26.505	32.19									22.234	17.196	30.671	37.868		
				67.38	35.987	24.488	20.919	27.737	61.713	28.321	57.055									48.982	22.488	15.616	70.93		
				26.898	27.52	36.949	10.517	27.778	68.976	28.06	63.972									14.965	18.704	24.33	48.626		
				31.25	31.118	52.615	45.803	34.307	55.945	20.438	50.287									11.228	41.716	17.807	39.683		
				20.554	44.151	25.132	23.81	24.582	36.797	31.746	21.164									24.537	21.164	28.933	59.51		
				44.52	54.708	63.526	77.529	25.242	51.581	57.122	65.303									15.834	28.995	25.254	22.487		
				28.183	24.544	31.533	34.008	34.392	21.164	25.548	14.912									40.864	8.883	33.577	31.746		
				64.773	13.138	68.511	25.12	36.226	28.889	25.719	10.882									27.124	24.318	30.853	14.855		
				88.362	76.494	40.769	20.212	22.485	38.212	64.082	52.284									13.228	30.423	37.176	44.974		
				22.058	13.813	30.74	33.659	36.139	47.425	38.82	32.287									15.873	43.522	14.55	27.124		
				33.28	40.702	19.841	33.368	33.132	31.746	27.054	46.766									19.841	23.367	28.446	42.579		
				34.443	61.42	11.883	30.782	26.949	80.47	42.844	45.136									19.412	32.555	59.75	31.533		
				44.066	32.214	35.96	47.728	10.582	70.106	38.36	62.996									16.317	19.841	15.368	39.194		
				63.012	14.55	41.806	16.712	22.583	60.284	37.407	14.792									23.433	24.517	49.193	24.269		
				14.924	59.755	23.669	71.089	16.873	24.083	12.715	71.429									20.577	17.196	18.704	62.016		
				21.61	16.388	14.965	43.979	50.628	34.003	46.296	22.225									23.81	31.19	18.781	24.167		
				40.535	66.216	10.236	24.805	48.542	36.116	23.676	52.49									35.113	22.407	10.289	23.669		
				54.21	44.005	11.224	27.778	12.71	55.074	37.07	51.843									32.095	13.195	41.108	20.913		
				25.211	55.222	53.314	53.506	45.762	43.678	27.251	55.608									23.424	25.132	49.676	49.34		
				24.262	24.75	21.298	19.841	24.863	49.675	10.59	55.201									14.134	19.841	15.825	13.228		
				23.132	16.131	13.228	12.835	20.913	60.884	27.095	41.12									26.441	42.989	30.423	26.053		
				74.351	72.629	24.924	21.302	19.173	46.15	26.882	18.088									28.959	21.301	18.519	20.047		
				20.78	65.696	17.363	27.138	28.226	43.785	67.94	23.379									30.423	14.03	47.619	30.381		
				55.431	17.196	30.821	32.643	67.453	25.56	13.099	19.108									43.364	19.642	43.182	61.952		
				34.952	46.366	30.286	8.68	19.356	20.085	10.582	20.121									14.55	24.08	49.249	34.646		
				44.974	31.508	37.338	35.149	18.54	33.892	28.803	29.957									62.667	29.101	47.702	24.965		
				38.288	87.656	34.7	26.455	26.449	52.515	40.578	30.366									21.164	21.825	41.005	24.318		
				23.137	24.104	21.142	48.733	31.94	43.203	25.107	24.374									21.137	19.28	20.552	15.873		
				47.856	51.388	61.164	54.265	41.797	44.53	23.451	22.593									13.857	15.873	36.709	39.284		
				42.371	25.248	33.987	43.448	32.548	57.28	19.333	31.025									29.954	8.79	42.216	30.952		
				25.482	14.087	37.505	39.738	15.668	34.392	33.59	43.713									21.298	23.008	17.228	43.358		
				34.644	70.607	15.902	10.582	62.812	39.246	24.266	14.55									51.486	18.925	15.973	42.57		
				27.748	66.07	28.372	17.648	12.024	51.337	24.566	27.22									25.112	66.447	43.878	25.986		
				46.864	44.659	21.164	20.708	21.083	68.61	36.162	43.643									14.03	32.116	21.653	20		
				22.994	69.822	53.751	20.879	38.36	24.056	26.825	10.827									15.873	10.879	19.398	41.928		
				15.074	37.864	27.778	41.438	24.148	45.234	25.214	23.708									26.139	53.706	19.642	40.754		

				22.487	53.639	26.898	31.172	11.905	38.075	17.232	23.81									15.389	7.833	12.848	54.266		
				28.022	58.773	35.714	34.515	10.804	27.778	29.841	28.102									41.288	11.851	27.119	55.46		
				22.974	56.039	12.869	29.483	25.049	28.391	18.837	36.226									42.027	23.3	40.539	23.332		
				39.741	29.298	77.077	44.835	11.816	48.576	41.185	18.519									25.881	53.941	41.681	38.658		
				77.338	65.532	38.884	18.934	31.6	65.465	52.188	16.952									26.741	11.352	29.193	33.989		
				60.261	77.758	45.946	29.362	27.392	31.746	38.443	38.897									44.974	46.634	38.454	43.186		
				22.492	12.484	34.914	41.761	34.623	66.444	41.333	10.582									26.75	23.095	14.55	15.873		
				28.581	34.049	9.586	55.628	49.602	33.402	41.855	57.686									14.55	20.117	43.508	40.204		
				53.863	46.15	32.284	65.652	31.011	15.873	27.223	15.901									17.196	14.303	23.193	55.556		
				38.699	16.836	36.108	31.767	19.642	33.072	24.947	47.73									12.863	34.829	37.713	22.488		
				47.358	21.843	35.433	13.618	19.465	18.947	41.742	9.321									14.301	48.637	20.117	37.874		
				22.457	43.002	52.91	12.399	27.104	34.392	35.089	19.024									36.978	21.513	32.223	40.522		
				43.651	37.237	32.04	16.612	39.078	22.448	35.542	35.38									18.457	27.827	35.937	44.346		
				27.922	67.111	43.807	11.9	33.412	47.665	28.143	50.904									15.873	42.821	40.219	53.005		
				30.79	35.335	56.463	19.532	21.298	16.863	57.908	27.551									29.101	56.492	57.061	50.051		
				23.573	74.826	21.164	47.784	19.292	29.718	29.175	60.869									56.922	18.72	20.855	33.274		
				24.968	27.778	25.411	42.225	37.44	27.44	23.96	19.465									32.449	42.381	19.048	35.714		
				34.303	60.379	45.001	26.092	25.934	54.181	51.686	25.54									10.537	18.595	22.749	48.637		
				30.483	18.279	42.716	40.018	42.898	41.145	36.492	22.585									22.459	13.018	34.198	35.593		
				20.292	53.313	23.81	26.026	35.526	18.413	41.044	23.158									29.101	35.571	56.743	24.682		
				27.771	38.665	27.509	74.467	32.531	15.054	38.097	14.55									43.347	42.414	30.662	23.81		
				52.91	38.68	36.316	50.873	19.355	39.577	22.448	29.351									18.519	47.671	28.407	54.217		
				54.076	50.96	23.327	32.736	27.244	60.756	26.189	25.107									28.545	49.088	10.752	57.413		
				50.822	22.448	37.264	35.542	20.215	53.253	40.31	28.48									24.675	21.044	38.439	26.026		
				60.959	18.795	26.455	24.511	48.336	10.641	40.864	19.841									10.295	10.582	23.678	24.965		
				34.973	52.322	25.132	40.36	18.219	18.295	58.095	13.951									57.019	35.175	37.998	22.921		
				64.281	31.806	20.227	22.593	20.577	63.069	29.101	27.453									26.455	15.697	23.927	23.81		
				24.526	86.671	34.241	33.559	16.784	23.238	45.676	24.912									24.532	21.927	52.91	44.28		
				77.427	28.884	29.304	24.082	39.356	37.659	15.986	11.783									11.224	35.557	21.412	28.265		
				40.219	54.826	36.685	62.485	54.228	17.193	41.261	45.076									35.168	12.968	38.686	21.344		
				30.804	39.711	40.067	32.428	38.112	39.437	52.11	53.567									18.186	42.328	28.994	24.58		
				58.589	38.315	46.296	39.683	55.965	35.131	34.51	19.298									70.665	27.104	34.602	29.302		
				48.26	22.487	33.947	13.228	43.587	18.519	17.059	19.841									22.576	27.566	31.296	37.067		
				33.313	27.924	17.199	26.084	49.38	24.229	16.528	8.836									55.786	10.582	30.506	48.549		
				15.873	41.005	21.026	9.755	17.984	66.488	54.399	52.884									34.864	19.966	18.519	29.396		
				25.026	65.946	30.874	34.482	26.455	35.542	13.589	20.577									23.085	46.144	13.228	36.988		
				43.117	72.773	53.557	21.583	25.519	37.137	26.189	23.303									39.851	13.449	13.669	13.46		
				33.487	43.651	31.384	47.856	20.965	20.474	39.147	12.234									27.124	14.55	51.439	27.778		
				23.75	22.362	44.38	34.465	49.702	29.93	35.688	19.412									34.685	39.222	24.985	9.259		
				38.967	63.602	35.982	12.645	61.011	41.29	18.698	12.198									34.697	19.849	37.438	21.135		
				59.524	33.069	39.641	21.997	18.151	50.163	43.463	48.734									12.44	23.81	22.487	32.208		
				21.381	46.924	35.627	28.06	11.686	39.837	27.094	21.991									10.517	14.965	15.21	14.792		
				36.357	54.747	48.942	19.841	39.296	55.906	36.063	44.904									21.164	49.408	26.455	21.387		
				16.925	56.12	39.683	37.352	47.25	21.164	33.722	35.08									19.841	33.77	41.755	11.868		
				20.423	24.993	36.979		45.444	23.055	42.178	76.869									23.428	18.903	18.415	22.294		
				34.961	32.883	19.82		31.746	41.232	14.55	10.549									16.826	17.196	29.377	22.487		
				47.616	31.222	29.619		20.868	45.194	40.903	30.86									28.995	33.069	28.73	24.362		
								32.179	19.931	24.555	19.334									14.03	20.893	33.009	21.552		
								34.146	20.577	44.505	26.455									34.44	16.627	40.994	10.582		
								46.668	20.446	21.164	43.093									28.383	8.19	22.448	41.273		
								12.025	33.069	36.703	52.392									27.611	10.866	13.228	31.746		
								27.668	46.751	15.153	14.965									17.196	15.295	13.095	20.173		
								35.002	43.342	21.891	44.994									26.455	33.722	16.539	9.682		
								29.101	52.622	57.055	47.921									16.844	48.352	16.807	23.553		
								29.377	18.553	45.878	12.484									36.736	23.426	12.848	7.937		
								20.344	23.801	19.841	21.448									18.519	13.228	13.763	26.455		
								40.782	48.768	29.013	39.048									23.618	26.084	14.55	21.164		
								55.264	43.651	24.89	17.196									9.342	32.571	19.73	31.059		
								32.736	27.455	35.549	25.829									31.645	35.556	50.478	36.764		
								39.683	48.196	13.646	25.132									15.873	13.763	36.415	21.164		
								38.905	9.105	32.498	9.993									11.834	15.983	11.115	9.353		
								40.089	24.194	16.57	11.905									16.408	33.834	10.588	15.35		
								49.36	58.164	30.866	19.603									19.841	39.683	26.189	17.985		
								45.571	33.722	33.384	35.613									24.67	23.072	31.746	16.406		
								15.432	63.602	13.095	28.27									13.578	21.316	15.389	14.199		
								13.228	15.432	35.867	16.321									24.793	13.228	9.259	16.609		
								35.327	38.106	49.954	37.783									13.919	31.886	33.135	14.632		
								25.254	37.037	29.75	23.7									28.809	39.683	40.922	18.825		
								38.276	42.855	33.379	32.736									20.371	46.232	17.196	26.726		
								40.849	25.175	27.532	41.372									26.405	62.329	17.771	22.504		
								28.258	39.474	55.408	13.237									17.16	11.659	12.031	45.253		

								27.555	13.095	14.55	25.638									15.873	38.365	20.762	21.827		
								38.029	46.769	24.234	35.268									17.996	19.224	25.132	26.513		
								61.216	40.219	30.054	17.196									26.926	24.925	21.539	39.658		
								27.169	39.061	49.817	30.337									24.131	20.026	32.677	15.402		
								21.216	50.871	10.045	29.52									22.205	13.228	37.037	28.654		
								15.545	62.341	41.753	29.553									12.782	21.856	30.099	8.418		
								46.066	13.979	40.687	28.39									31.619	38.134	16.317	15.873		
								19.233	13.996	39.804	23.442									24.515	11.905	17.196	23.81		
								20.711	16.307	11.28	17.76									36.669	14.352	17.581	25.254		
								44.281	75.397	20.569	69.07									11.224	14.03	29.581	11.424		
								18.338	28.24	42.609	52.49									11.625	18.058	11.814	34.727		
								19.433	40.913	20.173	32.068									21.164	19.841	33.111	19.414		
								28.255	26.419	16.048	56.391									34.326	9.259	28.131	19.599		
								36.716	34.594	20.702	55.155									30.423	28.208	16.446	32.399		
								36.585	86.862	20.577	47.02									19.499	20.414	19.343	16.862		
								13.393	56.898	23.833	27.778									37.413	19.892	15.386	9.696		
								24.318	59.353	36.247	15.873									44.493	15.873	16.256	23.872		
								17.18	47.754	12.425	29.852									28.995	14.97	23.81	29.93		
								44.073	49.43	44.535	44.703									18.972	35.859	22.167	13.337		
								20.954	16.336	18.211	25.774									27.667	33.224	26.419	21.733		
								39.25	16.741	47.832	9.61									46.384	51.253	10.582	11.787		
								39.475	66.036	43.063	27.778									24.682	10.664	49.574	16.812		
								25.132	23.596	29.101	12.159									38.028	37.429	20.717	17.196		
								26.998	30.423	19.841	37.037									14.55	29.416	18.519	14.82		
								26.22	34.818	15.382	26.483									13.228	26.455	28.06	23.81		
								14.61	16.873	17.757	25.362									15.901	20.741	44.795			
								31.379	29.6	47.939	31.567									13.434	13.228	13.228			
								25.521	52.195	13.155	27.124									30.432	39.628	22.487			
								33.022	61.844	56.735	29.499														
								27.217	41.357	16.279	19.919														
								47.7	70.644	27.442	11.224														
								18.776	25.435	35.384	16.629														
								44.107	43.78	30.423	24.849														
								25.254	33.807	43.698	27.778														
								37.351	36.25	25.132	9.759														
								41.038	13.967	36.044	10.651														
								34.324	39.373	37.966	11.905														
								40.716	41.84	22.354	9.259														
								21.653	50.994	54.233	20.913														
								11.838	37.037	14.377	23.524														
								51.258	34.392	27.523	20.377														
								10.04	40.963	15.834	34.234														
								13.262	26.455	21.819	30.043														
								14.55	45.774	39.683	37.378														
								20.546	27.807	63.98	14.55														
								29.836	25.863	43.601	47.66														
								30.561	47.012	37.44	27.235														
								32.727	61.175	19.642	26.455														
								44.352	43.171	36.434	28.158														
								19.356	24.159	35.287	30.423														
								17.548	55.556	39.164	40.423														
								62.169	39.539	49.149	22														
								25.466	28.062	40.89	12.389														
								45.589	29.863	22.084	14.55														
								27.213	37.324	38.252	29.93														
								11.605	29.549	36.545	45.859														
								26.189	29.702	19.295	24.318														
								22.487	42.725	27.778	20.881														
								11.241	55.167	39.16	43.645														
								49.595	34.445	29.416	14.659														
								15.222	41.885	26.298	12.273														
								19.517	45.511	11.905	39.559														
								48.414	42.328	25.449	20.891														
								34.981	47.609	38.484	20.626														
								17.771	34.133	9.033	35.457														
								34.546	31.09	34.138	16.223														
								25.132	53.847	49.888	21.301														
								31.509	23.107	54.079	31.746														
								47.619	29.101	25.833	15.873														
								39.927	39.481	33.129	45.595														
								35.308	33.058	33.281	28.431														

								22.925	18.38	23.64	39.772														
								27.085	44.38	67.945	34.333														
								19.934	17.842	45.961	47.631														
								27.835	45	26.124	14.681														
								30.423	8.824	22.178	74.217														
								11.724	48.08	21.982	63.492														
								43.504	52.685	58.926	30.486														
								39.544	52.91	45.645															
								19.841	62.667	27.213															

21 days following freeze injury																									
Pax7-M10cKO																									
cle #2		Muscle #3				Muscle #4				Muscle #5				Muscle #6				Muscle #7				Muscle #8			
38.396	62.746	60.796	67.816	58.201	37.501	49.256	40.705	50.051	27.778	10.582	59.34	26.455	33.951	48.824	37.037	21.857	19.841	40.238	55.627	17.793	19.413	27.053	52.91	24.864	27.217
55.556	35.937	54.751	81.937	66.328	61.276	48.595	26.603	34.388	76.324	18.519	35.714	15.976	67.669	26.347	34.258	13.051	25.535	29.606	26.24	29.189	26.455	39.975	70.564	38.143	18.672
45.881	16.564	43.651	9.259	80.851	63.574	28.995	20.802	63.849	21.164	14.442	61.795	96.194	17.287	20.639	43.96	22.8	12.719	25.449	37.916	29.242	71.178	30.978	44.974	48.184	29.737
45.891	90.188	54.974	25.132	29.707	18.519	44.673	33.196	28.902	22.035	37.797	68.219	55.617	20.559	42.6	23.489	35.83	29.93	43.881	25.671	37.802	43.651	45.222	16.836	37.584	23.383
71.883	16.788	33.672	33.868	41.201	56.878	26.82	17.005	21.502	33.447	19.513	47.171	70.789	64.116	35.761	43.557	53.144	39.738	61.313	35.491	44.286	42.322	39.683	56.949	9.344	32.214
44.464	24.997	28.933	84.743	71.005	10.582	24.318	9.259	26.461	18.835	55.329	61.553	48.301	29.93	52.811	66.879	22.448	30.946	22.593	40.071	30.469	25.543	57.285	56.478	26.309	45.63
70.095	34.607	48.439	88.362	15.666	25.798	19.465	16.836	46.241	17.648	28.008	88.052	75.571	21.298	37.278	18.849	32.274	43.651	51.608	38.164	35.979	10.651	50.215	62.348	49.586	31.356
56.19	18.519	46.978	39.683	15.389	32.361	24.044	9.259	30.745	12.848	45.115	84.652	34.258	21.164	13.641	46.147	54.744	19.321	38.927	10.582	10.51	36.383	9.259	48.231	25.875	21.186
20.887	51.566	33.672	17.857	34.894	65.077	17.485	31.746	18.756	14.984	24.065	62.327	34.334	38.03	39.683	34.392	11.905	21.164	51.951	31.846	38.36	29.101	44.865	28.707	47.268	42.009
50.571	42.328	30.385	51.479	72.2	93.072	25.864	30.884	49.353	17.219	35.236	63.602	51.469	51.464	46.184	57.637	29.146	48.834	51.175	32.089	21.283	53.097	56.935	30.579	44.896	9.911
25.132	77.854	58.39	13.741	73.547	64.664	16.836	24.945	40.504	19.534	11.905	58.758	30.666	40.793	38.108	24.684	30.296	51.443	62.412	50.063	13.856	46.893	37.738	21.072	26.441	23.81
67.738	32.41	52.633	62.654	103.945	31.984	18.519	47.846	57.745	24.701	18.27	65.727	15.332	44.525	52.763	21.072	23.81	62.083	35.759	40.926	19.25	27.778	42.485	83.313	36.956	19.892
49.967	10.652	48.912	26.643	30.964	65.556	30.308	12.479	50.254	11.905	26.74	45.518	57.83	70.895	30.147	41.61	14.55	22.598	25.213	26.189	42.599	20.345	49.312	55.184	23.428	22.487
11.728	48.56	30.708	66.363	79.18	67.711	30.799	44.109	38.59	12.835	24.386	54.128	57.759	68.808	51.759	23.679	12.434	19.746	41.457	53.408	23.383	54.193	46.021	37.129	21.164	48.611
47.183	23.81	59.369	18.681	64.815	43.057	13.763	27.453	32.911	43.651	14.965	73.956	65.912	58.825	51.14	34.327	57.988	38.348	15.873	40.965	17.771	26.455	15.873	17.036	27.616	44.974
52.205	63.9	61.764	60.884	46.663	47.259	25.777	18.052	41.245	60.447	13.839	89.762	47.78	50.63	33.494	52.284	16.574	43.623	67.947	40.551	38.36	35.397	46.608	27.668	24.389	11.905
50.304	27.452	23.494	51.587	46.567	20.292	34.895	59.544	16.917	12.368	32.26	22.916	37.697	22.234	61.757	38.904	14.205	35.126	54.233	44.888	24.794	18.052	49.679	29.101	51.532	25.626
56.747	35.714	37.302	41.623	77.524	62.97	49.098	20.738	47.281	16.566	14.017	42.08	64.881	31.746	58.011	30.604	17.196	26.455	30.423	43.194	8.418	48.898	32.749	50.979	19.196	39.447
42.168	30.423	22.603	18.343	56.744	103.292	29.585	50.752	14.869	17.005	34.979	42.136	57.992	43.615	41.258	42.409	33.19	29.251	27.854	31.007	17.074	29.481	56.061	19.255	45.519	60.92
49.894	36.658	29.785	78.473	8.664	49.86	25.157	25.741	19.642	22.554	21.601	73.499	34.607	18.506	16.906	25.254	24.713	15.172	55.092	34.235	20.553	29.611	44.798	62.408	23.621	42.715
55.917	37.86	32.437	58.953	17.645	11.905	17.545	27.585	58.052	14.965	27.68	63.797	36.668	8.418	11.905	53.859	17.771	35.714	68.069	48.942	30.81	21.1	44.093	20.55	45.054	13.217
31.19	75.074	38.928	92.738	25.092	54.233	27.124	23.075	35.345	23.872	27.124	46.684	91.369	11.503	10.851	41.275	31.367	61.732	51.565	20.932	32.299	29.151	40.252	44.243	53.256	26.379
56.94	15.873	37.059	22.434	46.296	56.071	17.97	24.614	18.199	14.55	39.708	50.898	45.712	43.597	42.328	46.831	23.488	23.776	10.59	29.143	10.438	42.26	41.792	45.376	58.864	23.489
24.71	38.898	13.779	62.829	51.587	62.446	35.313	19.396	17.771	57.473	22.452	85.062	51.858	13.654	42.583	58.664	17.747	60.449	31.83	22.564	14.55	36.373	29.442	18.064	38.36	27.481
47.702	21.739	69.105	89.998	11.775	22.487	60.892	26.455	44.538	26.868	41.422	63.001	46.927	28.635	38.1	48.653	21.164	34.029	14.302	19.533	40.749	48.177	18.288	46.42	23.374	64.478
26.065	19.966	57.607	41.005	78.695	68.66	15.873	32.559	21.298	28.199	58.302	93.102	58.385	31.425	36.486	31.746	41.005	26.455	23.988	23.762	30.775	33.306	54.082	43.006	34.646	33.184
12.428	42.767	36.384	42.904	21.895	57.055	22.432	27.97	53.649	11.878	39.781	71.492	63.504	21.164	36.895	41.005	19.998	50.316	53.969	38.871	41.29	24.097	41.011	52.922	12.489	26.455
62.535	16.576	14.03	71.914	43.22	49.115	19.841	15.873	37.857	40.213	37.525	57.452	39.645	40.662	22.487	48.521	19.874	10.482	79.791	39.398	58.971	47.405	53.226	36.279	46.332	56.673
48.221	30.901	15.901	47.619	24.425	51.655	19.381	38.806	33.231	41.825	26.937	61.927	11.224	13.761	16.672	17.474	32.281	34.882	54.151	26.998	31.34	38.824	35.199	17.196	18.357	39.225
66.257	60.467	42.748	52.31	21.653	40.5	24.878	42.013	35.879	28.247	17.196	41.434	44.974	13.382	17.221	31.223	31.775	43.755	12.49	30.865	23.082	35.578	40.92	58.339	70.574	37.856
23.81	31.45	70.779	29.84	73.268	48.324	32.119	42.955	35.823	31.577	13.809	40.929	28.598	28.394	40.32	35.803	26.534	9.259	17.163	56.184	10.224	25.824	24.342	49.345	66.948	34.392
70.165	57.735	59.419	62.662	58.572	47.961	17.132	36.46	62.353	15.566	26	80.913	63.824	34.217	47.235	24.93	38.348	14.55	9.259	41.834	29.93	21.839	46.766	46.193	34.392	39.609
32.796	28.749	61.906	52.291	24.878	71.8	44.871	11.528	56.31	23.533	23.7	39.819	55.558	21.311	22.487	44.684	29.101	56.214	66.99	41.03	31.803	17.085	84.299	51.548	37.037	27.778
42.683	26.535	42.166	91.313	68.125	40.755	35.904	9.259	38.891	28.863	55.447	47.928	59.362	23.862	26.005	30.414	12.325	13.612	69.238	50.882	49.705	48.81	14.742	62.667	30.775	49.785
22.234	45.132	41.009	66.323	42.648	33.136	36.287	28.242	21.095	30.689	26.483	51.683	43.025	72.797	29.355	36.66	16.744	21.513	28.561	22.522	25.132	49.917	53.553	51.514	45.246	56.274
57.537	30.736	11.224	27.76	56.706	65.653	46.296	55.928	40.452	35.042	10.582	77.143	26.395	48.767	11.251	14.55	18.519	45.176	39.284	47.848	41.753	34.607	46.252	25.132	36.666	47.959
46.255	58.926	46.866	64.591	73.613	24.817	62.77	37.102	59.207	13.228	40.69	93.554	57.187	25.198	38.725	15.35	16.826	37.557	29.874	23.383	26.455	38.536	54.312	44.912	28.778	34.65
39.069	46.561	40.256	72.131	69.352	16.66	13.095	48.362	40.78	30.999	50.882	66.332	58.95	33.528	39.349	32.736	15.011	62.976	33.938	24.913	17.196	41.217	86.602	26.455	25.17	14.209
21.164	23.052	50.162	38.697	46.365	29.101	21.164	33.25	19.427	27.376	26.166	45.86	47.918	14.559	68.438	41.154	39.173	21.754	15.722	11.224	59.721	54.864	51.438	68.805	53.692	29.12
42.346	19.917	53.107	61.57	91.901	66.998	41.361	51.799	17.684	35.993	19.082	49.604	73.473	34.178	52.73	34.755	23.8	25.584	32.874	19.642	12.899	21.53	50.265	62.408	58.201	67.586
48.028	40.592	54.311	63.215	27.096	56.458	21.379	10.33	13.329	10.02	17.771	67.644	31.055	40.098	34.392	22.828	17.196	25.107	70.542	36.478	50.265	48.255	59.555	80.417	34.369	17.196
29.091	43.651	39.782	45.56	13.362	12.665	29.285	26.455	15.804	39.294	40.476	47.543	13.27	14.03	52.747	32.742	30.423	29.079	25.816	41.993	27.167	27.184	77.517	45.621	45.618	36.3
28.925	77.733	51.443	59.048	75.847	66.878	17.698	35.5	45.574	30.369	14.442	65.085	36.811	43.489	59.147	21.667	18.835	40.342	34.137	38.36	27.778	39.493	68.713	42.932	30.348	32.239
19.841	49.37	45.491	94.769	22.561	34.841	19.169	20.382	38.206	33.773	40.46	54.962	38.496	58.083	66.372	42.485	22.547	18.519	42.328	46.46	22.92	31.684	31.136	39.486	62.54	17.771
22.487	79.201	46.907	48.342	57.321	54.233	39.837	25.531	14.662	21.164	39.683	59.756	49.062	42.716	45.907	14.438	17.054	17.074	12.363	11.244	22.874	37.037	29.047	63.161	37.735	51.966
29.495	21.032	22.487	22.977	26.061	11.911	19.574	10.625	21.213	19.336	14.864	77.768	73.52	33.267	10.582	15.873	45.148	58.065	32.044	42.683	19.581	31.75	63.492	56.878	29.961	30.785
20.653	20.661	40.093	59.93	29.738	50.615	18.069	11.412	12.671	30.46	12.922	47.683	47.411	46.546	17.834	42.09	51.809	56.573	24.266	39.683	14.55	41.836	56.791	75.451	30.171	36.362
33.567	28.631	36.758	34.392	55.343	68.137	70.733	25.556	25.842	22.448	20.67	63.592	61.002	32.768	59.712	20.742	24.207	24.082	14.09	51.587	12.421	35.117	39.222	33.59	23.432	25.645
48.632	53.716	15.766	59.242	15.049	36.362	25.132	16.616	33.672	23.183	36.469	47.401	42.328	47.619	47.311	51.342	20.465	34.931	21.604	43.651	37.425	18.366	49.4	33.136	23.04	22.487
23.958	24.586	28.184	89.877	55.546	49.18	27.611	11.905	22.487	41.619	22.401	78.898	27.97	66.953	47.156	11.714	46.149	22.487	52.216	36.704	25.952	57.845	49.914	47.458	46.452	11.868
42.263	40.054	52.557	92.14	17.196	76.164	29.046	25.57	52.301	26.455	10.582	60.348	17.99	14.647	19.642	24.706	48.434	13.095	57.371	36.087	28.315	41.852	47.64	20.721	31.746	46.459
31.767	43.625	44.974	63.172	52.566	55.544	26.627	70.942	55.831	15.983	14.643	57.728	15.873	67.094	28.176	43.786	13.228	41.832	47.808	45.872	55.837	43.458	67.068	42.43	54.855	11.905
29.23	54.222	39.296	37.27	57.151	59.073	17.147	41.561	12.934	16.836	19.369	43.901	70.761	61.265	29.705	28.031	17.295	41.88	23.81	29.066	22.729	33.114	62.265	25.132	56.476	60.457
20.878	47.645	52.34	96.756	68.087	55.613	36.087	15.242	48.513	34.91	34.737	69.337	26.998	46.766	44.463	47.619	41.165	26.189	46.754	45.802	21.751	29.482	82.011	35.557	46.009	33.069
38.893	38.71	37.636	59.524	55.556	20.743	40.552	15.411	42.328	11.868	41.713	63.833	50.686	33.652	24.98	15.873	19.114	17.151	38.348	54.139	34.764	22.405	106.091	43.651	19.841	32.736
52.063	14.196	77.125	31.689	42.819	52.593	14.55	26.623	39.683	21.513	8.13	80.201	46.478	31.107	11.014	34.334	25.804	35.977	37.861	28.876	51.693	35.714	31.925	13.846	46.399	25.608
13.228	38.36	29.113	65.792	85.147	60.936	26.945	29.93	54.233	18.825	8.855	90.522	55.966	27.778	49.912	30.751	13.228	42.678	70.85	49.021	39.58	22.874	44.689	8.613	16.659	49.868
32.736	83.238	35.297	23.81	49.593	43.082	42.134	9.836	29.76	31.434	21.315	58.189	49.9	19.993	8.418	20.434	42.596	10.582	28.556	43.107	38.778	34.685	77.522	72.233	40.003	31.746
61.3	25.018	25.066	55.095	20.599	48.576	41.218	10.289	30.866	22.363	26.838	19.685	44.48	52.91	30.127	31.058	38.023	55.464	12.159	48.746	20.807	26.455	50.04	25.738	64.386	27.77
54.233	29.101	44.974	83.153	22.417	60.978	52.91	19.841	12.553	21.936	21.412	51.292	52.516	39.691	43.002	59.729	54.055	19.841	15.873	22.448	28.91	22.962	44.818	18.549	42.254	40.672
16.072	14.792	53.94	58.205	43.733	66.841	24.856	14.55	18.185	48.371	17.964	17.707	39.645	16.336	28.898	62.376	11.905	46.296	17.151	13.06	13.557	47.233	39.147	12.824	15.873	15.205
21.862	26.672	39.286	47.619	11.819	47.313	29.406	40.465	17.92	13.976	26.898	77.552	49.052	29.64	26.547	41.942	13.228	30.423	56.211	31.746	10.879	18.937	39.976	37.037	19.456	31.395
36.107	64.592	39.977	12.032	61.838	67.439	31.106	23.613	31.959	18.65	40.124	60.646	44.341	14.841	69.459	53.192	24.043	33.888	17.081	29.726	23.665	14.356	70.149	40.811	20.424	24.853
18.004	51.327	50.265	25.529	48.743	62.489	65.228	21.448	15.355	11.129	14.03	61.54	52.656	79.652	23.81	32.186	48.364	15.077	62.022	37.651	9.985	61.594	14.225	35.766	43.536	40.891
30.696	51.203	27.503	47.619	24.318	56.816	8.84	10.464	22.651	28.057	26.455	49.226	55.669	28.578	14.965	29.371	21.164	53.858	27.778	43.513	38.85	29.588	55.612	11.018	20.75	22.321

45.657	32.777	68.147	49.249	57.285	73.42	21.164	18.934	29.93	31.763	17.858	63.572	59.825	31.255	19.885	26.455	16.644	29.71	31.357	42.344	56.12	18.741	67.349	27.528	22.487	47.415
38.738	61.071	32.548	77.815	21.458	61.63	33.048	34.392	31.022	17.196	26.963	83.449	62.277	15.564	26.191	48.14	34.068	44.308	26.321	36.478	18.799	49.509	27.654	70.021	43.954	11.905
13.228	37.637	32.251	46.296	87.946	7.899	22.786	19.532	27.925	58.127	41.711	52.063	70.413	27.075	26.441	35.407	45.653	47.619	30.32	39.399	21.458	26.455	84.039	51.015	49.526	41.394
18.94	61.999	52.378	51.508	68.994	67.075	31.583	34.857	63.793	41.674	28.995	68.041	30.158	43.088	18.307	36.675	39.284	18.381	39.527	41.476	37.037	34.22	45.891	36.257	43.651	29.163
20.577	57.643	18.2	56.693	21.032	76.4	22.448	10.582	48.73	17.137	12.092	54.391	84.656	50.508	25.132	24.793	28.925	20.196	74.922	12.307	14.642	54.31	43.796	56.257	46.619	31.686
26.972	48.929	31.067	74.851	30.618	56.015	15.873	18.519	22.638	19.269	7.946	48.535	31.931	24.724	32.122	63.976	9.259	73.613	36.861	9.948	17.196	32.086	49.729	48.448	10.884	44.963
19.642	28.556	17.479	61.732	48.942	20.68	32.446	31.277	50.63	33.718	26.455	42.225	12.549	19.513	18.32	61.831	53.581	50.552	21.336	11.905	29.805	43.559	18.519	50.295	61.918	20.117
12.195	32.822	43.453	29.454	72.2	42.114	35.306	32.281	15.47	30.923	44.842	59.428	46.141	17.617	18.519	13.018	17.574	23.132	20.461	29.29	39.24	49.85	57.403	28.865	39.683	32.285
21.557	24.977	46.931	22.487	57.596	58.088	23.083	61.156	23.085	23.562	15.873	56.044	60.102	21.898	25.346	43.574	15.453	55.321	18.681	23.341	15.873	21.997	41.154	16.772	62.89	34.823
31.409	33.672	53.796	66.875	23.81	48.544	14.681	53.314	45.63	20.714	11.905	70.106	62.169	22.487	22.402	19.847	22.446	14.03	26.904	62.956	44.785	8.372	62.904	83.184	13.228	15.117
13.228	12.785	33.372	76.378	29.101	64.815	47.144	15.493	64.748	30.423	47.878	71.973	93.581	73.153	31.415	10.005	19.841	40.884	32.67	46.419	30.775	44.893	58.151	31.388	46.516	25.804
45.948	19.989	11.905	14.55	65.728	51.474	21.025	15.921	19.642	45.564	26.184	44.274	44.273	31.064	44.485	36.922	34.973	15.873	14.55	43.106	11.838	36.821	58.595	58.152	22.497	13.891
21.164	34.071	54.099	50.219	40.752	79.75	17.753	15.901	61.297	15.538	29.479	67.96	43.396	81.003	9.625	45.466	19.642	19.049	65.994	21.305	53.273	34.251	64.59	27.778	38.03	22.412
51.541	54.003	41.005	51.934	21.104	71.899	33.726	23.672	18.781	47.619	47.434	61.428	29.669	14.477	45.608	32.296	16.947	19.841	44.974	43.651	19.527	25.757	39.091	20.292	23.531	55.454
35.629	55.812	56.152	58.146	56.076	32.427	17.163	46.566	60.749	14.55	19.881	65.094	41.877	38.9	11.251	35.198	39.683	20.504	46.978	21.164	12.586	32.961	86.612	66.36	31.355	31.322
17.576	14.323	77.985	80.709	60.312	62.446	34.926	14.965	60.96	21.164	23.676	40.647	61.564	49.388	67.063	29.946	19.642	37.655	26.36	26.399	24.215	19.527	74.27	33.436	49.384	34.766
28.397	51.587	48.824	54.871	52.113	62.068	23.596	19.957	32.893	19.841	22.809	80.917	63.797	65.647	44.333	41.25	25.162	52.211	89.387	34.329	26.775	50.048	49.854	72.955	29.013	24.256
28.384	52.209	83.13	24.835	23.346	35.513	17.964	28.708	32.6	24.843	21.283	69.79	103.792	29.966	14.647	25.372	16.34	68.847	38.36	58.021	41.005	32.983	10.601	9.415	18.619	25.083
47.619	26.357	52.245	63.692	93.81	36.582	58.706	47.848	26.455	39.718	13.095	60.865	63.055	15.873	27.778	30.488	33.028	34.392	7.937	27.156	9.064	25.613	25.75	41.537	71.538	39.9
17.005	50.265	48.365	33.65	26.455	36.352	31.594	32.731	40.796	13.248	33.873	65.567	38.461	47.165	38.247	27.946	48.258	11.224	10.582	37.217	35.986	71.623	99.825	68.783	22.749	16.687
11.224	41.32	47.619	47.098	35.434	15.382	52.539	29.26	16.959	41.005	11.744	64.383	20.553	14.03	56.788	21.55	32.501	13.228	58.339	36.051	29.963	42.328	78.988	28.306	60.847	21.549
48.637	54.121	52.63	52.618	15.873	34.317	39.303	22.811	47.623	20.072	18.006	50.392	21.123	38.36	41.005	16.41	43.127	39.525	23.81	23.707	15.901	39.294	56.567	17.771	38.227	18.832
13.467	23.008	63.247	57.766	73.868	38.36	63.51	10.224	36.474	21.98	40.312	75.41	85.979	20.599	17.163	44.772	18.932	11.849	22.448	39.068	32.839	45.625	15.152	17.332	45.27	38.023
13.228	41.322	20.553	16.836	58.575	39.514	27.705	35.817	21.289	21.453	72.013	59.428	11.528	54.604	13.895	17.571	36.642	19.398	26.054	61.937	42.249	43.526	30.938	23.299	23.825	9.259
13.228	29.93	57.109	63.539	21.164	72.56	39.673	12.815	26.778	30.707	40.219	25.103	42.716	28.19	49.713	65.31	31.237	67.351	11.161	52.173	36.478	42.576	13.228	46.332	24.266	38.592
16.321	29.555	14.526	55.115	54.669	55.713	38.427	21.895	20.772	24.682	20.051	37.451	58.884	39.683	26.033	27.57	12.027	19.77	10.289	42.443	10.513	63.387	14.55	53.033	35.142	30.145
54.04	81.203	19.226	49.266	60.547	30.173	15.873	43.106	10.388	19.672	35.474	59.406	57.472	67.735	44.045	16.613	37.408	22.044	46.644	47.924	12.706	28.594	51.584	55.254	25.132	40.049
16.788	27.62	94.891	60.774	41.103	27.691	30.423	29.013	66.915	28.233	15.278	54.234	38.573	17.778	10.582	12.838	17.715	45.276	10.336	38.818	33.141	35.708	57.823	50.889	66.121	15.976
32.927	23.949	69.852	59.192	34.589	66.397	25.132	29.101	44.201	26.698	17.196	52.104	37.067	31.309	26.606	30.423	19.642	52.378	14.281	25.132	26.21	52.363	42.829	43.309	49.122	42.591
21.513	35.752	41.619	30.358	16.838	61.933	44.269	18.196	33.124	49.981	18.838	64.777	71.949	30.778	31.949	32.311	42.341	27.541	27.184	29.336	33.14	42.636	14.896	37.577	17.75	23.742
24.965	11.905	39.965	71.117	18.767	46.091	24.849	41.398	34.65	20.855	24.256	89.842	41.453	22.13	32.658	42.22	11.39	47.464	21.914	18.72	44.088	23.665	78.567	52.638	20.983	15.237
8.333	18.519	44.805	41.161	65.547	32.736	43.628	21.513	28.496	20.553	20.22	49.909	36.839	46.296	32.539	16.085	16.836	25.132	48.909	27.691	31.311	39.683	69.689	10.582	10.904	10.582
27.528	35.666	33.442	37.665	21.506	37.511	29.739	41.005	42.09	67.826	9.304	21.89	27.124	48.442	26.419	36.982	28.842	31.443	17.614	24.683	45.831	35.405	51.416	14.03	26.799	39.821
33.217	44.896	41.005	52.438	55.663	25.877	40.232	13.228	17.196	15.873	18.264	42.027	35.136	28.995	40.775	27.057	11.905	21.936	7.937	27.139	39.888	11.224	56.465	46.71	31.298	34.914
53.093	14.55	19.527	58.073	29.642	41.851	21.095	19.056	74.015	26.189	19.994	63.666	76.366	34.581	23.422	39.686	17.156	21.164	14.55	46.566	19.531	54.074	46.737	68.43	29.111	23.336
30.765	30.542	17.715	49.429	19.642	66.408	15.903	21.164	48.942	35.714	37.314	66.139	60.237	52.91	48.234	41.682	15.606	26.455	55.981	34.392	40.079	41.607	27.787	41.022	17.771	11.57
14.55	73.593	49.545	58.945	17.292	24.999	33.069	33.541	55.64	12.114	31.067	74.475	70.149	36.894	48.942	36.545	38.991	35.881	41.398	39.456	9.74	16.387	38.162	37.037	26.455	31.638
19.841	34.908	64.82	60.955	56.309	58.165	29.101	25.934	49.988	32.364	21.14	73.559	35.714	30.271	66.523	48.873	9.259	11.747	39.683	43.065	14.55	40.017	35.542	31.431	46.911	15.047
13.228	22.916	56.159	62.699	54.839	60.486	13.099	47.619	35.017	16.585	23.972	86.615	20.219	40.494	44.816	34.65	13.007	64.815	18.005	34.131	33.883	19.841	49.44	18.664	10.04	21.825
17.196	31.746	37.965	49.786	42.197	52.91	17.575	15.392	49.932	36.093	29.101	67.694	51.946	41.839	25.829	34.994	23.321	22.228	56.12	30.171	19.858	13.677	65.261	59.255	37.066	35.314
24.135	50.718	53.104	56.84	39.683	56.927	50.097	27.217	27.692	19.474	26.24	67.783	20.997	12.134	26.357	56.036	43.96	17.196	19.45	33.86	24.192	33.863	83.707	24.129	15.08	24.588
12.148	50.875	66.721	18.553	18.925	69.787	16.859	52.1	35.714	15.336	39.334	12.216	45.482	42.773	44.151	58.434	18.535	45.237	18.627	14.965	50.265	49.088	42.556	13.925	24.263	13.228
47.918	34.914	37.867	84.271	62.505	28.338	52.88	31.349	37.723	9.259	69.755	54.968	36.738	36.493	22.487	34.452	45.616	58.073	44.002	49.715	44.357	25.132	42.805	14.228	49.772	13.469
57.045	13.716	93.271	62.357	40.261	24.959	53.183	18.519	48.869	15.973	33.735	55.528	44.267	15.427	17.75	7.937	18.707	13.22	35.714	37.413	17.476	46.7	92.998	14.681	51.581	10.011
20.117	15.873	38.816	65.954	13.839	29.804	21.836	25.132	74.038	17.574	25.901	72.072	36.767	36.478	44.611	49.807	32.495	31.752	26.788	25.439	48.583	18.983	103.67	19.841	32.938	7.937
23.81	27.898	18.825	65.671	47.208	58.416	21.339	54.272	17.567	19.115	20.5	51.503	34.262	18.519	16.334	35.199	27.778	20.762	23.618	53.058	30.993	20.841	52.269	45.161	17.196	19.524
14.183	34.273	81.072	43.786	36.545	59.698	26.547	14.55	22.959	14.979	21.134	93.8	9.259	19.827	22.286	23.079	18.519	21.836	19.902	16.788	39.645	15.976	62.071	51.072	48.892	7.937
23.031	39.683	62.603	66.624	34.392	48.413	29.746	30.782	32.997	33.994	32.47	73.378	34.905	53.837	36.882	46.978	44.974	14.354	41.824	36.469	21.175	41.005	44.896	32.331	17.575	18.519
55.066	50.021	36.24	16.954	43.205	45.616	52.596	32.393	13.8	15.873	25.449	49.893	35.142	14.202	14.024	58.181	29.484	47.957	26.989	42.002	11.995	9.1	27.503	9.259	28.981	13.736
23.215	56.914	40.442	36.026	56.509	17.771	37.841	39.683	36.49	37.546	18.519	69.4	22.162	41.633	39.309	26.863	34.63	38.36	45.343	34.813	27.185	57.33	57.036	93.301	36.545	29.93
11.905	51.381	52.546	32.891	43.651	51.759	26.321	19.841	23.097	40.351	23.602	55.556	10.582	45.881	46.808	57.866	23.742	28.966	51.177	28.852	13.813	38.281	31.071	101.064	11.905	39.749
35.287	23.383	48.472	94.718	15.873	38.36	39.683	13.228	32.541	13.999	14.55	13.467	38.031	60.605	19.993	29.101	32.539	21.296	21.164	29.549	15.047	42.989	41.709	58.094	30.087	31.818
13.794	58.725	90.727	31.746	62.243	55.627	47.485	26.916	42.2	19.642	8.283	92.107	61.337	25.132	53.314	46.655	34.149	32.096	25.132	29.101	19.477	17.196	32.362	32.313	50.095	11.838
25.823	44.536	47.924	38.108	50.204	42.807	20.121	29.101	35.761	9.353	31.316	52.708	42.184	85.562	13.503	28.995	23.738	28.962	39.683	40.104	16.736	11.551	56.873	47.328	25.132	23.506
19.464	34.392	75.864	71.002	58.588	60.844	14.643	36.037	17.834	26.667	11.905	61.416	49.002	41.419	28.995	14.965	41.687	27.294	63.476	34.821	14.965	41.99	25.132	31.368	42.023	9.259
63.106	34.769	5.583	22.765	55.994	74.965	50.939	43.001	14.332	28.017	49.074	59.041	64.688	16.358	31.825	42.028	23.346	26.455	59.524	51.587	14.304	46.773	19.462	10.582	23.81	27.901
14.681	33.359	22.487	70.71	9.911	30.492	20.708	18.519	38.345	30.959	42.369	65.787	57.46	30.096	9.717	25.132	19.298	26.545	34.711	46.63	31.801	49.572	52.91	54.876	21.688	18.519
10.897	23.72	38.074	60.791	80.999	49.686	13.918	20.718	17.196	25.441	13.027	56.928	31.628	61.162	11.905	26.781	30.56	7.957	47.478	21.498	39.683	21.164	99.537	42.09	13.705	51.416
23.383	48.942	55.972	36.796	48.942	18.141	15.799	15.431	43.651	23.411	22.339	50.407	75.028	28.403	43.96	32.196	12.835	25.325	19.126	19.33	33.672	33.069	55.038	41.975	41.661	58.666
8.786	29.028	69.554	55.598	69.843	64.066	14.438	17.196	44.175	40.999	18.292	57.121	98.414	28.647	38.281	44.719	45.983	55.631	44.638	47.081	50.265	51.644	60.307	16.757	38.771	30.423
26.795	31.746	19.054	64.345	67.813	57.672	50.265	14.068	74.732	31.215	29.199	64.151	20.08	14.55	20.361	64.815	31.117	14.03	46.392	39.261	26.455	43.789	46.338	13.575	18.519	31.801
37.037	28.401	19.54	16.836	37.066	14.793	62.888	30.923	59.296	19.841	23.672	76.192	51.877	18.15	63.513	9.259	37.44	46.296	63.939	24.893	49.912	18.258	58.201	73.147	42.328	17.196
30.423	32.443	39.196	7.402	59.609	56.959	31.355	23.894	29.286	26.636	9.617	64.384	56.808	15.411	23.261	27.111	22.482	54.665	18.692	28.132	35.069	34.334	73.607	36.478	41.005	46.9
50.931	49.684	22.539	62.013	33.068	30.423	51.203	29.17	34.468	40.219	32.557	77.013	21.628	21.182	23.848	42.96	14.696	29.988	22.084	48.768	19.841	45.428	53.266	23.881	56.057	25.779
23.498	31.379	34.971	64.922	42.031	30.018	29.265	43.274	59.497	20.976	28.959	55.37	34.71	27.124	19.95	52.142	48.639	30.519	69.484	44.493	34.228	51.034	52.121	19.793	24.682	10.544
41.832	52.378	56.507	63.907	58.782	36.577	30.978	54.249	53.243	24.267	16.372	56.938	54.956	22.069	49.131	47.619	64.602	20.775	32.324	21.164	49.281	47.021	23.351	20.553	11.65	31.746
34.678	95.52	62.667	30.971	64.022	32.736	53.874	8.344	29.456	19.577	14.03	86.147	43.778	15.386	8.988	33.968	22.053	19.676	51.673	38.838	24.229	45.31	25.254	18.519	19.033	45.192
13.779	58.201	66.371	26.455	57.451	41.919	45.831	19.832	47.629	21.453	30.945	69.072	27.089	83.346	25.741	32.684	27.213	52.953	32.379	14.681	39.362	39.63	85.56	20.535	16.746	13.228
45.796	10.284	35.036	46.903	33.069	43.96	33.616	26.093	28.174	23.256	14.55	53.106	47.277	27.778	47.319	40.028	21.513	26.461	17.972	33.602	33.984	33.069	50.91	20.287	28.231	11.905
22.487	62.154	25.971	50.644	13.228	44.278	24.318	80.84	54.92	21.53	27.547	78.601	68.326	19.841	16.88	31.746	15.873	42.019	18.519	26.419	11.42	37.035	46.054	53.212	29.122	55.057
16.921	32.783	48.371	50.334	10.654	26.177	52.404	28.062	53.28	23.81	58.489	41.041	76.24	21.164	19.841	24.651	43.549	34.183	59.762	35.757	31.598	31.794	66.138	21.298	14.55	45.15

18.14	59.923	61.978	23.81	53.578	11.905	68.541	23.81	43.465	62.053	39.717	72.846	20.405	32.406	20.442	32.441	25.741	41.362	38.65	20.521	37.037	26.455	55.505	40.829	58.033	46.112
11.589	36.35	60.41	54.677	30.97	46.561	43.651	16.9	17.196	33.931	13.095	54.233	73.794	29.576	19.524	27.442	44.781	22.913	43.651	46.783	48.38	25.919	63.843	72.237	54.073	44.946
10.006	17.729	63.53	24.704	19.841	39.639	37.509	30.423	24.707	28.165	9.259	23.079	55.556	29.7	22.487	14.162	41.422	26.169	35.542	31.99	14.225	29.744	45.118	19.122	35.335	44.199
26.455	61.012	61.877	14.03	58.978	38.761	22.487	24.318	15.482	29.084	22.448	24.435	52.405	20.994	41.031	59.404	51.379	25.796	10.582	50.881	13.228	21.62	57.823	23.134	77.532	16.612
17.968	17.196	60.348	44.555	30.147	25.254	15.467	16.836	58.746	35.131	18.707	69.486	29.613	36.535	26.455	19.491	11.775	22.058	21.11	27.772	34.392	24.898	62.862	37.39	12.984	30.73
17.196	50.368	21.298	59.4	50.937	44.123	51.831	30.275	59.668	23.81	33.605	62.928	56.749	38.355	39.284	15.952	27.323	23.286	38.097	33.001	12.515	62.341	23.744	35.578	40.227	30.866
39.361	29.689	12.14	45.817	45.293	70.336	23.81	27.554	33.585	52.629	11.688	40.756	19.841	24.092	39.218	50	20.539	46.766	27.404	48.724	22.099	23.601	47.724	18.707	7.542	23.81
20.811	27.778	46.175	60.049	60.646	51.125	32.002	33.333	41.005	38.931	27.807	55.244	30.288	39.623	30.339	56.179	22.381	62.169	33.798	26.455	44.454	31.079	28.661	25.695	26.425	34.804
13.228	15.873	55.217	14.994	45.252	33.672	47.392	11.905	43.803	41.627	26.706	76.4	84.928	29.581	57.926	21.792	18.519	37.241	53.164	36.83	15.873	33.807	76.224	43.651	19.412	21.9
70.43	65.531	48.463	48.784	64.032	51.103	15.655	32.736	59.173	38.793	13.051	69.11	52.506	59.175	14.024	10.582	10.564	18.519	57.627	28.593	11.587	22.487	82.647	60.424	29.588	46.771
23.81	48.941	37.413	10.582	43.651	34.345	63.268	22.386	35.792	15.262	11.905	74.185	27.13	25.132	43.912	27.778	30.88	27.807	22.312	13.839	14.965	27.778	54.005	14.796	12.271	19.527
17.402	34.852	50.665	63.492	22.498	41.125	19.177	31.327	17.151	22.43	48.765	36.74	30.383	18.417	55.471	18.519	31.374	11.905	41.877	21.688	48.587	33.942	56.12	38.689	58.201	23.383
25.466	21.305	56.996	61.142	30.129	38.298	25.648	22.443	80.688	20.718	14.232	48.269	51.049	23.241	53.265	18.707	19.052	22.058	22.487	36.478	11.558	41.005	16.298	33.337	33.237	14.03
17.196	10.46	54.324	63.576	58.201	56.651	15.08	11.905	21.298	30.797	27.423	52.709	68.986	27.571	43.089	50.151	17.507	26.621	36.861	15.331	21.477	27.916	69.701	19.828	46.851	35.982
43.516	58.85	68.261	74.333	65.31	13.608	61.1	30.866	20.892	19.841	62.169	98.21	44.871	25.132	23.888	13.982	17.33	19.623	49.664	58.894	48.942	41.555	20.051	29.503	55.556	14.55
51.599	33.069	55.184	14.039	60.07	45.795	21.164	47.273	53.03	29.151	22.764	17.571	62.88	31.54	17.991	23.383	76.471	37.037	31.746	10.045	45.196	25.926	67.344	66.606	43.025	34.531
39.895	25.588	16.903	49.695	24.33	29.372	42.292	41.55	61.234	24.841	12.336	88.322	69.007	21.825	19.924	24.137	30.089	39.683	21.067	28.06	14.559	39.307	20.711	28.725	42.091	19.524
11.905	9.259	68.424	11.064	79.272	19.572	33.672	27.126	47.619	18.155	40.26	78.042	59.441	19.816	22.487	21.164	15.985	44.357	43.803	42.09	48.942	19.729	58.404	34.676	43.063	19.199
24.077	61.536	65.313	47.256	65.433	17.363	42.354	14.506	29.71	43.025	21.677	17.771	28.292	42.765	13.228	35.502	17.008	40.095	26.036	26.642	30.015	51.203	41.302	31.033	34.154	26.21
15.873	69.572	85.924	54.233	71.233	33.471	55.269	80.408	30.16	45.828	34.973	66.007	51.283	39.841	60.356	22.167	33.333	20.483	69.704	32.044	51.796	30.851	72.933	77.3	15.047	56.878
18.519	23.744	20.092	51.587	59.546	10.342	9.259	22.402	38.6	27.593	14.543	62.278	12.399	36.486	13.718	58.146	41.995	27.778	52.218	53.314	58.187	36.947	42.328	37.926	33.338	39.468
16.048	42.271	34.039	54.894	66.138	20.336	34.48	17.196	52.795	22.839	17.196	58.91	73.573	26.56	25.937	31.193	23.573	45.248	31.801	45.665	29.533	31.678	72.02	21.513	52.666	57.888
35.966	55.018	54.378	63.904	58.946	16.151	21.164	29.331	37.037	25.804	12.765	65.473	9.618	27.834	31.153	47.368	13.951	35.494	44.9	48.518	11.688	24.286	52.753	67.46	60.847	32.367
46.459	66.676	58.201	54.159	69.628	41.707	21.164	25.804	28.878	34.913	36.771	43.11	23.433	51.769	23.81	26.956	15.064	24.295	22.759	38.895	31.68	20.934	27.371	30.908	53.326	27.411
9.799	24.575	51.338	64.279	59.148	23.286	51.587	51.496	50.508	13.839	23.394	54.338	66.743	26.019	29.879	28.45	28.781	39.683	42.09	16.959	18.309	25.797	52.306	55.229	43.254	47.428
14.55	26.914	74.642	15.054	37.489	49.367	50.044	17.014	18.519	18.519	25.017	56.673	51.443	29.887	11.558	63.911	13.158	17.567	50.051	39.13	16.317	26.098	65.486	11.299	36.023	56.973
15.504	25.124	40.972	49.024	65.046	30.439	25.341	18.636	16.921	30.48	18.83	52.718	35.714	57.4	16.836	15.873	17.402	27.778	43.006	19.326	18.612	16.393	35.688	15.873	52.228	42.056
20.147	30.89	36.968	53.569	48.52	35.193	19.255	22.487	31.467	21.466	36.614	78.727	35.714	30.344	25.132	22.487	42.881	14.55	37.385	56.677	17.771	24.856	46.506	19.642	26.455	36.634
62.065	43.658	28.231	23.81	59.67	48.126	30.841	50.608	59.586	19.255	47.619	22.763	39.022	11.905	39.899	47.313	14.125	22.487	13.095	20.403	35.563	32.195	37.841	27.778	57.171	65.62
14.292	13.095	49.166	81.044	16.584	11.069	44.327	51.36	20.969	27.721	37.366	61.069	65.063	39.043	10.582	29.645	28.722	21.865	54.249	49.935	42.86	12.558	55.429	23.062	47.619	43.499
10.06	20.711	52.643	55.102	21.76	32.179	19.091	14.55	54.102	29.93	10.641	57.775	41.228	52.073	38.768	27.007	34.392	41.049	13.228	28.355	38.148	21.334	66.605	47.55	73.218	17.543
57.81	72.357	45.878	58.404	66.863	66.297	46.754	20.302	24.289	9.3	50.214	84.533	48.287	26.221	18.133	14.816	36.753	8.745	56.79	57.217	38.846	17.13	57.614	60.092	45.882	43.651
16.421	23.669	42.1	55.759	82.841	18.237	29.066	26.189	40.482	18.94	17.196	70.149	9.259	87.641	38.1	31.801	17.99	22.459	17.48	37.037	27.778	42.328	103.821	41.082	74.664	35.714
11.905	27.058	36.935	28.397	42.528	18.355	55.184	23.81	16.321	36.478	51.587	77.49	29.836	43.622	45.333	20.879	28.364	13.187	53.272	29.101	12.968	43.191	41.888	45.903	25.132	34.973
16.919	43.776	42.966	75.44	80.227	42.088	25.668	29.143	40.923	19.096	26.455	67.614	43.626	60.798	51.25	50.214	20.386	75.818	29.101	28.854	44.69	34.392	49.379	22.487	20.08	57.876
29.93	57.429	9.799	52.753	46.931	28.48	12.721	21.101	62.733	13.228	26.83	61.34	68.521	24.219	43.517	63.9	15.873	27.895	18.169	34.106	24.318	40.861	15.902	66.013	38.098	47.063
27.901	54.53	23.574	14.307	50.265	15.645	32.517	20.446	20.721	20.391	20.661	29.103	37.037	9.623	23.573	45.939	17.137	36.609	22.487	34.426	27.204	52.516	60.796	71.031	20.124	29.614
14.566	20.782	26.653	86.083	68.077	34.414	23.576	36.832	8.252	43.681	12.838	50.997	71.546	20.59	40.996	36.534	21.219	30.449	53.085	35.805	37.27	59.564	61.198	43.624	39.618	66.918
55.806	21.714	27.346	39.683	64.212	50.496	25.759	33.583	19.839	13.951	24.965	51.127	13.503	20.813	20.939	39.683	66.323	18.448	44.797	18.519	14.82	41.494	21.513	15.834	60.07	55.459
12.239	37.037	57.876	70.223	73.195	50.195	28.724	18.519	24.793	25.833	16.174	82.022	69.231	34.323	13.996	19.99	21.336	12.159	53.026	48.302	15.873	43.96	54.504	67.281	14.118	25.072
28.337	43.032	15.47	64.593	55.641	24.212	27.267	26.089	26.107	24.256	26.474	45.293	47.691	22.229	28.06	21.164	24.277	26.455	60.847	40.522	41.313	42.902	51.847	28.938	10.045	56.312
16.292	15.505	51.206	32.717	48.899	56.877	47.293	18.27	23.168	31.746	13.996	52.277	22.278	27.958	46.617	27.036	46.344	26.189	18.961	19.966	31.546	27.778	16.269	15.716	49.693	14.915
9.525	65.46	46.443	57.425	69.214	38.36	45.757	27.8	32.097	37.037	33.379	70.436	26.64	47.892	28.713	33.473	52.724	13.276	19.243	57.954	51.794	30.673	39.478	42.328	33.903	15.888
26.772	24.049	73.79	61.054	53.875	28.231	37.661	28.991	27.511	12.164	45.801	29.888	35.24	52.509	45.831	22.487	14.03	50.508	11.905	33.86	54.019	30.423	13.28	37.799	52.113	43.064
19.534	10.582	30.557	11.528	55.393	54.098	22.386	32.403	43.321	49.408	20.117	92.901	28.426	39.496	12.685	22.326	31.746	37.264	29.381	30.423	27.992	29.101	57.413	30.8	37.577	53.425
14.55	27.77	51.587	51.268	29.909	14.55	47.382	11.052	24.959	19.841	11.164	58.435	17.648	61.756	23.453	34.54	34.392	69.575	67.888	43.53	25.394	30.536	52.91	17.321	55.049	37.718
18.519	30.423	83.728	62.02	25.132	43.915	30.442	19.841	26.336	15.873	68.14	52.772	43.979	67.919	43.961	14.223	33.902	30.205	27.814	26.361	56.133	46.296	83.333	36.789	56.311	33.431
21.891	25.132	32.631	26.357	52.454	25.037	17.771	30.423	42.606	15.054	13.608	23.952	43.949	49.428	21.048	39.622	31.576	21.553	62.956	19.651	37.308	48.942	22.487	35.606	27.778	47.249
59.199	84.656	12.835	44.772	60.292	45.343	31.534	24.386	53.941	43.025	16.364	68.393	33.752	60.042	19.572	39.683	15.386	51.06	28.995	51.459	23.659	29.863	44.278	62.169	11.299	15.873
14.681	35.849	51.556	30.987	72.672	31.973	15.908	23.383	55.637	12.755	26.079	23.81	39.683	30.803	20.858	26.593	11.551	37.037	32.195	45.875	47.232	22.626	64.33	40.232	49.572	29.675
27.753	61.361	57.544	34.392	14.544	43.452	37.277	12.984	33.572	22.485	18.595	91.62	40.89	66.998	26.468	18.519	17.156	47.662	28.086	39.134	29.836	30.824	44.977	63.789	50.585	18.707
12.833	62.861	31.124	55.424	69.89	35.542	21.119	14.55	28.21	48.464	28.708	67.314	31.225	29.411	33.791	61.694	38.174	45.831	50.864	26.244	32.505	50.265	45.206	53.461	33.337	34.537
22.344	60.033	57.395	22.448	68.883	23.213	31.294	12.664	10.879	19.642	17.571	66.97	30.423	28.556	46.856	19.892	36.668	24.985	41.4	45.773	21.164	16.57	47.619	36.848	43.25	18.937
12.165	34.031	34.861	41.32	71.321	37.173	31.671	30.343	46.766	13.228	30.234	44.596	46.777	25.522	44.014	30.197	49.094	60.268	28.218	23.154	21.301	44.169	71.494	25.989	60.606	40.615
15.873	48.081	40.835	28.613	70.962	58.193	70.894	25.098	58.897	54.446	47.141	56.241	53.15	23.383	15.546	25.989	18.608	52.68	48.538	45.881	19.524	43.071	56.878	93.945	28.517	41.005
26.653	37.549	45.729	17.777	58.61	11.601	39.683	21.271	21.297	19.642	22.406	54.605	45.467	46.662	28.874	39.144	11.834	39.288	30.175	57.385	54.147	34.723	62.27	55.367	47.619	46.158
10.582	16.802	40.571	14.55	11.905	8.418	25.769	23.341	26.405	21.466	33.936	89.614	47.619	26.843	40.734	15.18	31.253	8.661	47.597	31.509	14.03	22.448	44.54	66.867	30.086	43.383
13.24	56.784	57.864	55.737	54.233	50.031	44.591	23.632	61.352	36.478	23.81	21.898	31.766	70.754	15.873	29.101	13.04	27.778	29.431	57.341	34.465	42.328	55.582	34.509	40.592	31.801
20.394	18.519	71.646	53.071	32.349	48.942	56.652	23.812	29.836	21.989	17.793	60.316	77.321	18.08	27.81	25.628	13.018	48.454	11.623	10.464	26.695	41.641	63.288	25.052	21.164	15.873
56.316	22.08	95.858	30.362	45.939	47.302	25.449	43.596	70.357	26.808	13.618	53.58	69.648	68.675	16.319	39.073	39.778	32.936	44.263	25.36	7.694	30.549	21.513	30.077	30.853	36.486
52.845	62.732	44.532	57.307	55.825	41.686	21.164	27.511	23.81	12.978	21.634	64.066	28.257	21.299	73.943	36.393	24.049	20.507	10.289	27.693	18.519	43.516	29.09	56.815	31.746	28.954
40.729	58.201	10.582	56.758	58.959	24.267	15.172	26.455	31.746	34.32	15.399	49.595	13.228	16.788	33.034	42.596	47.658	42.019	41.032	26.455	47.619	15.873	90.651	44.974	37.649	55.848
70.689	70.408	52.456	76.976	47.7	12.543	28.169	23.25	63.89	14.398	18.519	56.63	64.042	52.409	25.342	52.073	17.196	35.285	48.91	21.389	50.43	54.387	44.339	27.77	36.662	38.244
16.27	63.88	25.2	57.27	23.81	52.703	21.013	15.08	30.284	31.746	25.584	86.492	22.487	16.788	15.467	34.91	29.922	24.256	54.392	10.289	29.101	16.965	27.777	14.792	41.867	63.575
19.285	28.035	62.138	54.27	70.439	64.643	27.817	21.181	39.528	74.241	14.643	68.783	20.143	19.841	16.758	8.688	41.692	23.81	38.976	14.03	30.16	39.076	36.511	24.968	19.539	52.687
42.019	55.94	52.737	46.259	30.134	34.155	47.335	22.487	11.905	36.299	44.294	34.476	19.781	9.47	57.426	45.651	10.689	30.066	43.072	38.348	14.768	21.103	42.939	28.268	25.386	46.629
18.723	77.522	47.387	84.822	14.521	46.822	17.576	33.069	21.164	35.455	17.184	42.311	38.85	24.66	62.219	62.169	23.81	28.45	37.621	27.611	18.519	17.196	47.235	26.455	54.849	33.234
25.79	31.721	17.771	27.258	7.937	25.638	31.255	27.065	14.82	14.232	11.757	58.856	71.186	33.059	43.555	33.069	34.392	16.752	17.771	14.55	19.932	16.836	59.749	65.864	23.843	11.803
28.231	59.405	51.597	54.225	64.995	50.265	25.132	34.787	43.765	26.455	24.575	55.503	25.282	23.455	23.99	59.372	16.32	37.447	23.686	55.556	30.464	32.629	67.516	52.606	34.676	21.257
14.483	33.722	49.573	19.841	69.827	55.824	55.091	44.896	32.816	10.058	20.884	22.487	36.102	38.329	71.429	31.78	46.925	52.677	38.463	42.1	43.96	19.841	45.77	38.471	30.233	41.016
66.058	8.481	14.559	84.753	56.12	41.913	31.924	15.759	62.97	32.483	46.205	61.714	35.714	54.234	26.791	92.508	27.296	43.693	71.725	27.094	46.605	13.558	51.042	23.81	63.132	13.025

44.961	61.825	63.68	28.594	65.632	13.239	28.767	14.756	61.763	19.145	31.941	25.082	19.356	42.872	31.426	39.448	28.581	20.577	26.455	23.81	23.259	49.145	11.132	35.714	39.068	24.713
23.823	34.778	26.309	44.699	15.873	15.392	13.763	29.101	41.23	28.487	21.044	69.857	38.262	25.198	61.973	36.622	34.443	66.879	9.308	41.971	38.559	40.485	23.383	57.16	35.17	30.004
17.196	34.414	70.344	10.582	33.871	48.373	18.09	15.382	29.071	32.402	59.761	52.169	75.407	60.406	16.736	26.04	27.301	23.903	35.092	44.863	41.334	25.439	33.672	26.455	25.132	11.905
13.811	60.108	35.102	37.102	22.532	29.703	70.696	30.721	49.197	10.582	16.745	59.764	21.032	28.994	38.085	37.486	17.035	8.598	37.027	17.266	18.707	20	95.852	22.487	38.884	57.635
45.785	48.875	66.137	69.891	56.314	43.793	65.147	19.456	18.519	23.132	32.012	48.637	19.841	17.151	25.441	31.276	42.964	14.13	33.721	59.849	33.672	26.955	63.238	54.233	68.212	11.905
48.942	14.841	19.202	15.719	53.801	27.593	36.975	23.164	25.132	16.747	9.807	58.299	59.692	46.296	43.13	37.037	38.08	10.46	28.488	53.405	41.851	19.841	51.474	75.397	20.675	31.206
39.751	31.746	25.356	68.687	56.18	33.316	57.953	18.052	33.069	14.008	18.762	19.485	39.344	72.671	23.148	24.942	28.934	17.757	31.341	37.844	39.699	54.188	61.483	56.354	49.574	27.715
57.055	19.729	28.383	33.37	39.529	27.507	36.607	37.037	17.987	17.982	22.487	73.894	14.03	15.386	51.305	49.261	43.78	12.159	62.335	32.005	29.101	14.03	8.312	15.873	57.627	56.929
18.488	36.478	44.896	21.164	30.83	39.641	24.683	17.244	55.22	43.255	30.736	52.453	34.013	15.546	19.642	15.901	22.487	28.035	28.819	23.562	37.451	34.607	55.832	10.879	33.726	18.773
11.014	12.854	39.284	74.016	39.048	39.284	20.521	20.013	14.762	6.614	39.336	61.269	14.506	40.128	28.401	19.44	23.31	42.815	51.992	21.954	39.683	44.689	55.335	22.939	17.381	15.482
33.069	48.034	36.073	57.414	41.4	29.101	69.099	19.793	56.151	17.929	29.575	52.639	38.944	33.757	44.176	33.069	25.879	11.244	47.619	42.828	19.096	22.443	56.878	39.77	35.168	29.101
57.211	59.702	72.024	58.201	82.303	11.905	14.55	9.927	15.577	23.81	17.196	77.91	21.989	15.769	32.497	16.2	26.455	22.959	33.672	27.778	35.154	32.068	16.836	45.409	33.672	13.503
31.945	26.003	33.855	47.426	44.214	47.619	37.659	11.834	57.518	19.632	62.713	78.974	57.285	34.174	26.455	36.373	34.392	27.723	16.634	30.423	31.799	21.296	107.549	25.317	14.692	34.392
20.013	76.375	90.77	14.04	50.265	23.81	13.267	13.228	47.081	25.622	17.204	43.746	14.55	28.988	30.769	20.577	35.878	56.003	35.914	55.456	61.732	49.638	45.407	19.245	24.397	15.665
23.092	48.48	35.295	78.026	67.53	35.935	17.909	22.519	29.066	35.046	14.643	28.995	59.405	40.342	64.433	46.131	38.074	29.836	27.778	29.101	36.486	24.049	64.837	30.096	31.75	51.204
24.672	19.575	74.258	57.118	64.09	22.759	15.534	11.018	11.747	24.878	22.319	68.02	14.992	29.248	11.905	22.569	37.805	24.318	31.633	18.945	27.916	10.582	76.743	34.212	36.528	32.524
40.219	33.069	25.132	71.377	62.862	11.058	31.132	23.353	37.037	25.721	34.303	73.457	47.619	21.223	15.677	32.964	11.744	36.093	49.735	55.236	23.81	59.441	54.011	39.517	22.903	33.902
23.967	48.942	14.55	23.81	22.487	35.329	14.521	28.45	65.226	13.195	20.442	50.265	42.53	11.014	19.574	35.335	29.93	29.758	12.253	12.826	36.477	35.264	62.983	32.5	25.132	24.411
33.733	52.133	70.704	28.362	65.624	38.711	40.717	11.871	26.553	39.778	40.219	70.587	36.721	46.741	20.319	24.917	29.038	46.766	25.988	20.045	27.526	43.938	26.905	26.321	44.283	23.32
66.138	43.78	37.853	56.064	62.529	18.068	49.672	21.146	10.098	14.725	23.786	37.413	61.42	15.713	13.095	22.487	25.132	43.284	51.443	22.725	33.314	56.085	78.917	41.387	10.641	33.136
50.418	56.184	37.845	48.93	54.641	14.55	44.225	12.342	18.284	24.853	20.235	87.898	23.825	20.877	20.181	23.913	35.084	46.54	24.546	31.801	24.207	52.313	65	39.177	15.901	22.487
33.069	47.81	29.593	51.542	34.392	12.745	55.383	19.841	19.641	13.473	57.441	71.429	20.121	26.189	54.094	33.623	20.377	27.233	52.708	26.908	42.955	63.2	30.866	53.314	27.692	54.633
8.74	40.254	52.082	55.556	9.215	56.735	36.139	23.018	63.531	25.035	14.03	54.112	42.327	24.276	11.838	13.228	18.805	38.36	76.753	15.498	35.542	10.289	99.206	49.378	34.527	39.77
14.768	56.653	41.97	55.89	47.409	27.901	25.132	23.186	30.129	21.164	43.799	49.48	19.841	18.265	54.724	13.461	23.877	10.749	51.318	43.508	24.135	10.582	22.844	34.994	35.432	16.566
22.89	30.821	76.996	68.783	30.784	25.132	34.286	12.732	57.008	16.906	29.886	53.213	25.062	27.917	19.841	28.39	49.3	16.104	26.776	51.913	45.403	32.727	22.906	51.076	56.117	32.18
32.257	20.958	47.352	25.132	15.873	47.786	11.719	23.422	52.551	45.681	56.204	45.828	40.055	19.724	31.746	16.583	31.984	56.347	26.455	45.177	30.444	34.344	41.94	36.827	38.36	42.649
22.487	52.228	53.847	59.981	58.753	44.398	40.752	46.505	15.778	11.905	22.779	36.677	60.847	14.223	27.995	59.593	19.841	29.93	24.687	18.72	26.956	46.021	54.9	16.705	39.155	27.047
15.891	71.429	50.131	57.209	26.846	49.572	21.318	18.185	34.392	24.287	36.084	58.426	17.196	31.395	29.728	29.272	23.779	37.02	41.908	49.392	31.801	18.519	50.66	34.215	38.588	31.06
74.283	13.228	47.619	13.228	11.012	54.089	18.832	32.621	28.693	37.588	34.449	50.09	27.217	22.487	32.966	28.337	35.833	37.196	17.16	26.629	34.392	29.467	38.433	44.699	20.313	12.44
15.166	36.478	43.655	43.651	62.94	20.419	43.269	18.472	39.313	13.228	25.464	37.009	33.873	18.519	42.521	21.816	23.81	54.233	33.332	61.992	16.712	35.714	15.59	51.587	50.265	59.351
31.162	37.432	52.964	22.448	68.48	46.271	51.587	35.542	35.173	33.357	25.124	85.949	14.977	22.105	25.864	19.642	55.944	14.118	25.969	62.738	61.732	65.473	27.124	55.168	38.188	24.139
49.118	12.36	17.891	21.164	15.577	36.762	43.593	13.808	56.045	30.851	18.519	24.93	26.953	71.073	15.547	26.455	44.967	13.555	36.35	38.156	50.265	12.808	96.283	44.433	17.196	60.293
19.841	42.2	41.747	21.513	68.666	31.746	24.626	12.899	55.982	33.672	32.539	47.292	28.515	25.254	16.965	42.005	13.095	12.004	35.714	36.694	31.984	24.886	44.544	26.581	25.132	37.591
28.081	51.879	71.445	79.323	64.731	14.55	48.075	21.164	64.744	26.455	37.037	27.953	66.423	20.774	30.661	15.069	14.281	32.977	29.381	49.091	45.534	35.563	76.463	34.392	30.288	27.381
33.069	55.736	56.878	47.736	62.499	28.344	37.022	10.111	12.962	34.547	29.954	52.378	35.76	65.595	18.934	39.294	28.365	46.161	26.58	33.655	13.228	27.901	47.954	47.391	14.55	35.374
15.237	47.537	52.61	55.556	74.074	38.535	44.974	19.642	32.818	14.873	12.3	37.39	69.751	23.411	50.318	35.643	44.974	28.105	20.958	20.577	30.32	44.762	75.091	35.714	63.781	44.672
20.969	22.487	41.307	74.213	17.196	45.791	53.039	30.296	21.164	29.295	17.196	12.65	35.714	40.192	28.865	17.196	39.696	17.196	25.212	45.694	27.52	27.778	54.058	39.958	58.341	16.529
21.758	69.654	40.933	66.972	35.979	45.788	24.007	23.383	10.688	24.398	19.841	50.425	36.606	59.154	26.161	26.455	18.786	17.571	25.621	44.922	27.054	38.712	65.136	27.778	54.7	38.806
31.064	55.163	94.345	13.388	75.052	52.294	17.196	22.423	16.318	41.361	33.348	75.291	34.157	20.788	19.524	24.697	15.873	7.872	41.93	53.821	39.712	10.651	57.594	48.56	19.631	10.609
60.549	15.873	94.841	74.356	79.503	32.998	26.401	15.733	44.69	47.362	16.836	70.188	50.048	25.874	17.938	29.93	22.229	23.742	47.921	50.699	14.55	20.775	54.866	19.739	20.148	38.622
13.808	69.411	63.937	74.074	60.135	48.942	15.873	14.55	17.95	18.35	45.938	80.226	39.654	10.891	24.359	15.557	28.401	20.117	34.174	32.174	17.468	16.966	62.08	26.623	36.073	52.738
55.556	9.304	39.304	35.714	52.866	30.519	39.406	18.378	42.539	24.277	15.558	45.999	25.254	26.633	29.113	41.875	12.835	33.528	17.87	63.714	19.609	42.328	46.777	57.018	26.441	16.736
40.565	47.619	68.74	36.106	36.693	18.262	26.189	24.318	17.905	21.369	26.255	78.834	13.228	55.129	12.848	8.988	19.209	18.917	40.198	39.301	43.651	15.485	49.897	48.535	55.253	30.359
20.932	37.037	52.91	62.169	26.749	15.873	28.021	16.037	29.612	38.015	19.527	59.882	13.634	28.464	27.273	55.743	15.968	19.169	9.243	51.813	19.841	56.839	14.681	53.778	35.245	59.755
14.55	34.733	69.779	27.785	38.396	10.582	20.944	16.836	8.65	20.465	27.732	63.971	45.478	31.686	16.336	23.81	36.835	12.491	24.318	25.434	37.549	22.487	59.205	55.821	26.953	35.006
42.255	11.113	19.841	64.842	16.476	47.257	11.905	10.278	34.392	13.527	21.173	77.664	52.443	32.141	30.523	26.535	19.917	17.015	13.167	23.632	31.646	22.828	64.373	31.621	32.47	12.116
20.465	12.428	32.624	70.503	47.818	32.004	42.674	15.814	41.005	21.134	13.696	51.395	52.642	41.235	46.845	11.905	45.398	17.163	11.905	18.519	32.736	19.642	44.725	59.848	27.778	56.066
33.063	55.865	41.154	53.936	41.825	41.504	45.372	27.778	12.431	27.526	20.912	81.989	77.553	52.934	10.582	21.146	33.644	50.761	21.295	24.64	33.302	38.483	16.939	12.683	36.081	52.035
33.672	54.249	61.724	75.101	57.933	17.576	18.707	29.689	34.392	39.516	25.132	21.716	50.575	44.033	36.892	38.349	24.266	56.83	49.207	15.593	24.616	25.892	33.64	39.422	75.796	36.888
39.683	35.789	64.815	79.818	15.253	51.253	37.522	21.495	26.547	38.529	25.548	38.797	48.531	21.344	21.283	38.171	14.696	23.483	22.157	62.815	36.336	48.728	55.421	49.695	20.901	40.811
11.266	32.804	37.688	44.905	75.319	63.406	10.582	29.793	47.17	21.348	28.621	59.779	23.022	20.19	41.983	51.587	28.875	39.84	36.493	17.196	36.7	19.766	82.539	17.16	16.215	37.452
44.201	42.328	70.693	39.932	32.736	53.867	27.729	17.423	34.057	35.714	19.841	76.415	63.075	61.668	43.746	33.296	39.683	26.169	33.672	12.159	48.929	25.836	18.519	17.269	32.916	47.619
18.384	36.295	84.946	9.353	34.686	56.645	31.746	37.716	27.601	20.701	15.789	58.449	63.349	27.515	27.082	24.856	11.841	52.895	26.376	47.46	27.291	30.125	43.557	47.878	22.487	35.433
31.971	47.316	54.676	62.961	53.314	54.431	28.06	23.151	57.562	45.466	22.98	52.199	72.726	37.986	13.228	32.736	37.045	20.169	14.55	43.766	9.955	28.319	62.412	30.12	46.296	24.817
25.856	37.381	37.592	11.463	34.743	23.724	23.81	13.388	48.942	33.069	15.453	70.223	37.037	20.442	39.683	35.714	44.591	31.794	23.81	46.154	27.778	19.582	39.76	27.408	22.856	25.625
67.351	32.153	16.836	85.252	10.006	48.967	16.612	30.507	62.821	14.868	8.305	36.906	17.427	11.905	12.719	31.437	14.03	44.307	24.849	46.73	33.068	29.369	58.972	46.766	70.851	15.616
23.8	25.439	29.101	57.967	67.157	13.398	49.132	41.607	22.184	21.164	29.101	20.114	37.413	37.217	15.873	37.764	34.678	72.764	42.034	22.035	26.376	32.828	39.773	35.792	54.987	54.402
26.455	43.402	12.428	76.83	22.487	66.561	12.379	31.763	26.455	57.187	11.787	70.503	33.63	27.01	31.746	47.197	26.455	26.169	11.299	21.73	12.114	22.34	62.454	49.132	41.154	39.594
24.187	36.709	46.408	14.965	21.1	92.968	83.363	27.155	29.668	21.305	16.747	35.168	23.81	23.579	64.187	17.572	21.713	71.962	54.867	30.483	37.866	23.81	48.108	20.577	54.793	31.801
70.117	37.249	65.028	40.755	61.96	75.397	32.962	16.311	50.408	24.257	52.594	31.264	69.529	14.55	14.55	37.823	17.468	28.707	16.453	8.701	31.639	25.746	60.616	43.923	29.954	24.608
14.758	42.668	23.81	78.042	42.328	79.4	28.36	40.974	44.308	19.75	20.632	86.839	39.244	30.986	36.136	9.793	17.843	23.769	47.978	39.641	9.259	13.228	68.625	27.084	40.448	17.984
15.482	42.981	29.93	44.45	72.977	61.033	53.222	27.068	24.818	28.394	19.729	21.895	25.132	14.982	21.164	24.695	24.909	9.858	20.047	19.803	15.873	20.127	51.376	33.069	62.404	30.423
50.265	32.921	63.661	26.455	16.4	59.949	23.9	22.826	32.651	22.532	65.771	24.675	51.327	23.079	22.448	41.495	21.666	38.36	13.569	31.374	57.691	13.035	58.416	59.927	28.597	20.721
14.55	38.348	73.147	56.625	42.328	42.306	21.072	20.423	33.57	24.08	53.809	71.085	34.053	32.525	15.77	10.964	30.071	21.164	24.853	43.858	51.035	24.572	70.105	26.68	30.731	12.031
48.887	9.259	66.565	13.155	51.833	26.309	29.889	18.519	14.965	13.211	9.353	23.276	30.969	18.343	34.392	17.673	13.37	10.3	37.598	16.66	20.154	34.868	64.828	40.219	23.446	10.582
47.393	37.02	20.426	44.974	52.516	9.279	54.851	36.107	34.507	40.789	24.981	45.522	43.596	16.613	34.607	14.021	17.976	23.383	44.974	37.296	20.09	29.563	36.832	17.196	21.759	13.228
18.398	33.329	36.044	52.91	61.706	68.627	26.097	15.873	28.908	26.955	26.86	16.686	32.126	65.611	35.325	26.371	29.101	19.841	33.285	32.126	36.404	37.01	81.795	32.86	45.608	37.037
57.52	50.562	38.36	95.459	23.958	47.641	17.132	20.853	9.537	20.376	20.637	88.624	60.139	50.508	43.651	19.243	19.245	48.942	14.55	19.217	13.228	44.081	56.419	11.905	23.383	46.549
18.519	45.738	100.638	41.391	38.971	18.508	76.697	21.164	58.684	20.683	22.488	67.46	32.297	25.167	35.381	16.514	23.383	14.809	43.932	8.418	39.484	65.71	12.553	28.258	31.681	38.924
38.258	31.746	62.179	72.211	58.201	64.829	10.151	20.791	53.736	42.276	36.485	37.949	26.888	19.642	18.519	37.037	14.55	30.155	36.707	13.778	31.233	11.269	59.672	42.362	38.36	25.864
56.878	37.413	48.485	14.55	63.734	54.866	14.13	23.81	28.284	20.069	55.215	54.233	23.746	67.352	30.423	57.415	14.55	13.625	8.418	17.196	28.502	44.974	28.488	59.845	55.169	27.221

31.023	42.519	17.436	71.723	46.812	70.597	55.917	23.81	39.638	23.81	31.21	48.667	54.695	32.493	19.259	25.24	48.942	21.318	53.554	48.35	45.782	34.392	54.987	12.159	52.898	21.859
47.619	36.843	26.787	63.144	19.524	41.415	41.042	28.614	57.814	35.622	50.847	76.318	31.765	52.942	49.804	43.431	19.177	24.387	42.645	26.769	29.101	34.154	71.366	25.138	14.767	19.259
39.266	85.752	54.508	54.109	52.177	50.063	40.996	19.841	42.328	18.519	11.851	51.088	27.778	28.751	23.669	18.934	63.898	27.778	42.792	41.696	36.499	23.81	79.693	23.031	54.181	13.228
62.447	33.853	72.47	30.423	52.91	65.554	48.312	14.799	32.632	46.614	22.672	77.303	45.608	21.006	52.901	15.787	22.319	43.775	62.169	45.815	10.956	23.241	87.213	25.542	63.276	43.124
19.642	37.703	37.839	31.362	77.722	35.179	40.359	19.968	20.4	47.304	17.771	60.524	65.695	17.987	19.841	23.81	52.292	58.572	45.705	26.189	37.378	19.277	87.498	25.949	45.727	42.718
58.859	44.446	79.65	30.689	64.292	21.989	18.316	14.643	34.319	28.419	18.693	68.894	54.962	42.566	36.868	34.392	86.363	41.678	31.337	18.083	35.57	21.791	27.977	24.853	47.73	50.832
35.977	44.845	43.025	13.575	74.37	60.847	11.977	31.566	19.513	25.378	33.069	35.714	52.786	32.908	12.727	15.313	49.399	22.995	42.931	10.168	38.391	28.446	55.806	28.53	17.648	19.53
21.105	23.81	41.005	72.071	64.894	53.425	56.486	64.017	34.885	35.183	52.293	54.211	8.332	42.312	21.051	48.16	29.246	71.321	56.047	32.202	30.423	35.275	48.623	17.984	10.464	16.836
12.449	51.443	46.254	10.582	13.515	16.768	30.423	16.263	22.542	26.455	17.196	65.155	15.645	21.164	54.616	33.716	56.421	34.919	25.132	23.372	20.577	13.641	46.857	45.916	27.085	23.22
34.392	36.7	30.136	80.772	53.461	59.475	80.688	28.426	27.196	13.808	9.224	45.681	11.254	15.787	17.196	15.661	37.616	24.539	55.568	13.839	25.172	33.808	60.939	10.582	27.692	20.652
31.746	28.45	32.657	78.521	21.513	20.807	42.139	29.835	60.069	30.695	26.455	43.402	30.847	13.21	15.906	68.279	56.878	11.224	50.028	13.154	28.563	41.191	62.079	16.529	35.335	11.744
26.455	39.403	37.378	16.644	69.214	49.102	38.513	15.577	48.811	43.651	51.737	56.586	60.625	69.373	26.21	31.746	54.96	30.125	28.141	25.346	42.328	27.503	20.724	34.392	32.947	43.519
45.738	15.237	30.89	67.338	70.986	58.926	71.07	21.164	24.711	30.712	27.64	34.704	51.803	28.278	26.856	35.503	37.631	23.81	31.22	53.334	61.376	25.202	26.221	33.904	42.631	16.59
25.613	37.958	25.248	15.047	72.62	23.765	55.556	22.371	38.641	15.386	39.183	39.683	9.826	21.752	26.455	26.189	28.85	17.16	16.634	8.824	19.841	41.757	41.617	18.519	34.295	13.612
71.839	31.069	21.164	17.707	80.507	30.565	46.082	25.522	60.117	11.905	29.76	45.754	25.132	35.131	19.841	27.484	23.169	32.511	26.054	25.204	54.433	50.626	46.719	21.379	18.177	14.567
60.828	33.069	64.459	56.158	48.879	9.569	56.071	26.255	18.343	51.071	38.92	21.534	75.036	52.378	50.148	20.556	22.462	36.075	16.747	29.875	29.992	19.356	53.532	28.843	34.676	14.55
13.1	58.181	18.092	69.523	80.954	26.009	14.03	21.164	38.36	50.101	31.483	69.061	39.043	45.412	21.164	39.484	36.658	21.164	47.987	27.281	25.018	39.474	17.184	17.246	63.406	49.799
44.994	13.237	14.03	26.455	61.916	9.472	24.091	46.21	49.401	34.186	22.487	44.974	79.365	22.487	11.927	18.519	13.018	34.045	71.182	55.184	28.411	37.982	7.693	20.218	24.357	38.36
67.599	41.707	75.862	61.461	58.31	16.841	42.914	21.865	37.616	34.896	42.584	65.498	39.134	23.541	32.643	30.922	17.196	54.934	21.653	48.974	26.629	49.408	49.389	47.619	37.851	27.273
12.117	46.718	27.43	53.84	71.408	64.528	33.041	12.159	31.427	15.873	22.418	12.948	13.095	13.095	20.577	13.095	18.448	28.06	32.388	25.132	11.358	46.032	64.336	60.664	49.698	31.125
32.404	54.551	64.962	52.074	19.841	49.761	40.539	21.089	26.304	42.372	52.065	78.139	59.786	49.37	54.233	15.011	26.425	47.174	42.613	27.223	18.519	43.936	72.692	28.584	39.466	63.605
31.92	49.425	40.434	37.864	31.564	42.499	57.09	24.831	42.002	34.967	21.681	84.509	36.1	11.905	61.422	40.055	78.092	22.405	31.38	48.853	48.075	29.426	32.955	18.42	31.447	91.825
25.946	9.347	57.475	39.678	49.793	29.101	55.31	16.836	32.188	26.316	15.054	31.801	46.337	32.095	19.169	36.076	13.228	12.515	74.345	29.361	32.297	38.082	48.406	56.028	54.934	71.679
20.711	25.548	42.328	92.898	18.723	17.028	73.794	26.5	34.607	27.66	23.051	16.686	82.669	54.927	52.91	26.455	33.794	55.295	18.942	30.027	14.55	39.725	53.314	20.647	12.341	10.651
33.888	44.974	37.505	18.519	36.349	19.841	26.632	17.068	15.873	27.221	19.876	56.811	101.769	34.392	40.215	10.582	25.033	11.33	33.291	11.905	34.32	50.234	69.54	44.464	48.983	51.236
46.296	62.675	36.93	52.409	24.735	70.89	19.418	15.973	28.792	13.095	9.259	83.021	29.747	50.629	45.695	45.977	23.848	64.815	47.691	29.101	32.545	11.558	55.184	21.164	22.901	15.834
25.538	50.124	57.429	55.611	9.259	59.524	18.124	42.009	33.871	22.612	39.683	25.6	59.01	37.037	42.585	13.022	39.661	18.41	22.407	20.718	49.011	18.93	39.618	18.519	39.022	65.244
36.953	45.382	43.203	83.669	51.88	34.055	22.856	14.442	65.792	36.105	9.017	28.156	21.21	30.863	43.312	49.492	21.513	21.431	42.984	40.852	22.526	41.154	9.353	13.612	55.083	59.524
48.991	22.487	22.686	47.168	25.695	63.13	58.539	36.093	11.421	11.905	39.655	51.223	21.898	28.753	30.624	49.791	29.251	25.132	51.831	42.599	31.367	28.397	22.487	33.142	16.844	86.607
64.579	39.633	51.733	13.228	52.642	63.567	36.538	23.506	47.351	12.854	15.386	66.216	41.298	26.084	11.063	13.883	27.778	18.707	13.27	38.964	27.596	12.843	56.878	31.003	28.633	61.732
41.106	45.773	52.91	21.164	43.651	64.325	9.259	18.472	17.4	21.308	39.301	80.818	32.99	48.593	64	35.443	31.746	38.422	31.583	28.863	55.401	39.901	88.681	18.873	45.459	27.599
46.296	12.024	25.132	63.061	48.029	51.867	14.55	27.526	55.134	23.116	12.719	67.302	33.069	63.013	53.356	9.259	24.849	33.622	27.778	26.012	35.187	31.984	50.508	46.161	52.378	31.746
35.45	44.31	52.946	22.029	20.378	42.985	69.422	14.771	23.694	28.269	15.81	48.485	11.235	48.734	57.434	27.322	32.966	15.565	41.004	15.115	30.599	34.226	43.25	17.402	18.343	41.422
44.208	50.375	64.487	31.931	18.088	61.55	48.106	17.292	19.876	19.841	20.5	77.295	27.066	13.228	65.829	12.852	12.425	20.929	24.362	31.159	11.397	14.618	52.684	42.507	69.572	42.991
29.024	44.588	68.542	19.895	55.501	21.819	14.895	26.22	33.649	16.648	41.397	60.847	46.481	28.217	23.81	10.122	27.778	37.953	25.678	35.518	38.319	44.698	52.636	30.423	67.916	34.128
37.166	77.946	42.443	58.064	57.975	55.006	21.295	50.552	57.553	26.776	42.371	31.365	41.419	36.078	33.471	34.861	8.631	15.873	28.227	35.348	29.356	31.686	54.233	57.285	19.085	70.106
39.262	20.714	80.148	80.902	60.851	76.368	12.341	13.228	40.193	23.81	12.797	64.433	79.772	10.289	48.331	17.698	39.284	12.342	28.404	17.371	23.132	47.453	67.12	38.971	43.527	13.228
50.048	20.448	71.777	68.25	45.839	80.241	33.136	14.55	67.093	16.508	24.318	49.901	23.853	29.101	20.577	18.384	32.407	46.237	21.552	24.806	30.885	45.115	18.54	11.299	55.556	46.436
16.317	66.808	66.697	80.165	40.081	81.014	15.655	50.691	16.46	17.689	73.707	55.939	21.164	38.978	35.542	34.646	10.651	28.06	54.869	26.455	37.544	56.635	7.937	54.347	10.582	
10.582	27.778	55.208	37.037	61.54	86.805	26.73	10.582	46.296	34.316	34.392	72.063	16.346	46.181	23.964	9.528	30.633	32.889	21.173	26.284	20.92	40.156	54.207	26.455	24.521	32.221
10.513	26.147	39.595	61.186	17.16	77.098	23.81	14.538	35.953	12.721	59.165	56.639	17.847	26.455	51.022	20.318	18.697	17.75	37.593	15.834	44.442	20.577	52.625	51.443	26.026	12.159
32.831	25.132	34.068	42.172	48.923	56.113	51.88	11.224	54.987	10.582	42.227	49.859	16.661	59.694	12.721	57.391	27.406	9.08	21.552	65.978	34.295	28.099	42.881	42.012	30.821	43.621
47.248	78.341	50.265	32.013	55.661	16.736	33.174	28.654	27.964	33.306	15.843	68.91	18.519	45.791	52.653	50.784	79.903	48.768	11.905	14.232	22.328	18.935	38.444	23.258	43.466	19.152
25.132	43.18	35.147	14.583	62.722	88.602	18.264	20.616	26.478	11.063	31.746	72.366	30.481	33.577	23.383	14.024	16.674	45.279	38.73	13.808	41.071	25.011	14.03	13.737	62.97	22.487
53.163	22.155	16.661	44.643	53.314	53.59	88.624	19.466	32.07	35.542	42.648	67.024	42.299	47.997	23.707	19.265	21.067	48.942	12.214	14.559	16.679	10.064	38.143	39.273	14.397	23.81
62.085	38.247	29.535	75.372	21.164	54.742	26.999	21.716	29.174	19.841	17.151	36.226	37.435	41.611	48.637	21.164	15.354	39.262	21.552	30.821	33.4	35.708	34.428	54.233	22.452	11.996
11.463	93.373	51.439	62.571	38.412	10.582	34.392	17.196	44.278	18.519	28.4	73.792	17.826	63.101	57.834	24.256	52.49	18.347	25.69	59.573	64.602	55.33	66.892	32.002	43.651	31.746
16.685	30.041	69.578	32.25	37.404	84.359	23.085	40.707	14.55	45.657	12.448	57.969	46.627	21.836	33.686	55.556	25.482	13.239	31.71	42.328	18.34	14.61	66.804	43.651	50.278	40.144
42.012	50.462	52.247	73.679	14.817	19.356	39.35	33.069	28.995	29.101	62.499	43.96	17.507	24.979	37.656	16.836	24.066	22.487	15.047	33.069	18.519	28.017	56.912	19.841	34.392	27.778
53.094	43.807	53.192	65.293	63.897	75.95	21.167	12.159	29.387	24.318	27.844	58.246	31.401	18.41	12.848	20.085	19.642	35.571	30.107	37.037	48.111	54.233	34.74	31.222	82.133	31.681
25.694	28.995	56.649	19.683	41.026	51.587	61.119	26.998	48.047	13.228	44.536	81.923	32.955	40.522	17.16	34.833	19.982	36.832	35.563	48.65	47.71	13.228	63.526	15.616	54.434	31.746
48.422	27.623	57.82	18.403	36.922	51.541	25.79	10.673	14.55	12.911	24.028	82.024	48.239	18.519	19.096	35.944	24.049	34.312	25.937	35.714	26.389	27.778	101.544	35.293	20.173	27.778
52.472	14.55	69.605	24.965	72.751	38.583	43.651	13.31	15.08	29.84	26.508	72.751	28.081	13.485	37.037	9.965	43.651	13.228	14.03	41.518	21.164	22.977	53.46	60.847	33.852	19.642
60.433	64.444	73.804	55.556	72.751	10.582	58.079	19.527	14.758	33.577	47.358	35.747	24.803	39.954	23.81	21.066	49.731	30.423	38.309	43.945	47.146	39.144	59.444	33.069	32.304	54.165
30.745	46.235	31.209	56.779	50.726	91.46	37.664	11.905	34.983	38.916	20.112	75.012	41.911	53.837	55.136	32.9	38.49	41.545	44.633	19.531	10.853	23.158	54.633	51.111	60.783	80.301
48.809	63.069	44.373	13.228	48.607	45.412	38.936	11.168	33.677	11.905	13.681	51.111	39.025	29.93	37.413	27.691	26.258	25.035	50.265	23.733	47.959	33.069	48.37	11.905	19.572	15.313
43.975	39.663	17.551	47.286	48.131	51.331	51.508	21.298	51.439	10.045	37.037	43.37	33.069	35.714	14.55	16.919	15.873	18.461	33.069	48.927	13.022	42.102	50.875	21.926	36.732	11.905
29.062	24.082	27.778	80.912	51.011	58.201	54.097	15.873	25.292	29.812	31.018	29.071	88.468	39.683	12.968	24.923	22.286	51.379	35.447	42.668	26.455	21.173	55.671	11.905	39.464	37.189
38.092	12.558	61.549	26.455	12.615	23.81	60.285	20.292	47.894	53.522	10.388	66.138	15.873	15.232	49.985	19.832	17.196	30.423	57.183	29.066	20.862	17.196	37.955	59.368	25.936	25.926
51.443	27.778	38.348	49.017	46.747	62	15.715	16.514	17.196	37.037	30.865	38.419	43.098	26.455	21.164	24.318	25.339	34.229	42.351	30.856	77.55	31.023	51.893	35.84	22.155	34.392
27.778	16.672	19.464	20.196	65.721	60.342	56.407	33.069	29.995	43.494	17.48	67.188	17.196	58.024	20.121	36.984	42.225	46.109	48.908	16.695	17.95	23.81	33.069	13.022	28.202	37.336
14.738	63.523	89.752	13.951	25.612	14.55	21.891	29.841	12.902	32.563	23.986	60.107	27.778	30.423	25.449	25.132	39.07	26.391	53.977	26.548	33.828	33.903	35.714	24.501	22.873	69.708
20.464	51.545	52.66	25.774	47.194	28.304	54.233	19.469	38.52	55.621	20.845	67.46	96.525	43.525	26.068	17.698	26.877	50.17	52.91	21.98	67.675	16.218	41.005	37.759	62.144	9.662
14.55	11.551	57.448	54.606	51.083	39.603	45.657	20.855	44.531	11.033	26.614	34.778	72.351	70.912	54.028	9.757	17.6	22.541	16.316	30.311	19.471	18.134	48.734	16.836	61.827	11.077
39.202	54.714	11.387	71.019	56.764	16.913	42.328	16.576	46.874	14.55	26.455	67.16	85.088	27.601	18.519	21.044	16.636	26.926	33.625	26.189	13.604	38.545	51.796	22.325	52.252	11.694
34.392	16.175	84.995	61.841	7.937	40.017	55.522	21.653	29.494	11.905	25.428	67.351	13.318	45.831	9.259	21.926	29.93	48.939	21.866	58.042	48.637	20.314	60.359	14.118	39.044	21.164
36.922	78.271	53.115	50.676	27.442	55.647	68.783	24.291	13.228	52.91	16.965	79.244	47.292	25.481	19.915	32.376	23.707	40.019	41.154	52.91	24.435	18.97	53.864	24.334	31.801	41.337
37.831	45.255	32.79	82.011	46.322	54.081	43.873	52.702	53.788	21.164	14.55	52.773	23.831	14.72	51.563	18.944	17.196	32.839	35.964	44.027	51.737	43.104	93.985	19.841	37.986	29.498
36.72	18.461	41.769	54.439	58.53	39.162	15.726	18.707	17.196	15.661	21.429	65.529	15.794	19.642	48.942	34.034	44.056	43.651	41.394	41.005	9.639	35.976	88.242	23.81	25.946	22.828
21.967	12.138	79.494	70.735	18.181	45.983	45.009	34.409	42.995	26.189	21.298	90.279	33.268	35.547	17.219	10.692	26.794	43.695	12.861	15.964	49.664	39.022	94.411	18.997	7.696	34.906
36.901		14.55	51.88	13.155	41.005	43.118	16.609	19.78	23.013	19.202	41.31	64.408	22.939	26.012	10.651	22.487	15.616	38.231	25.096	56.446	25.708	87.295	57.929	61.069	16.573

21.164		51.783	44.581	76.939	29.682	12.159	42.372	59.281	45.343	13.775	65.427	55.965	32.994	11.479	41.005	14.55	29.727	28.989	69.484	29.734	31.872	22.035	11.801	17.196	35.603
61.682		38.15	62.788	72.923	54.228	21.003	9.259	30.97	13.228	66.01	47.913	22.225	32.67	16.836	20.682	12.622	21.305	44.19	12.798	15.873	56.12	47.011	7.937	45.831	17.196
		49.272	81.475	43.142	43.96	57.951	43.928	48.54	23.762	30.252	57.578	24.687	17.571	69.183	22.55	28.887	19.841	39.683	10.582	42.328	54.061	17.912	25.132	39.435	30.964
		77.653	71.715	25.132	14.281	51.158	23.504	40.915	36.674	33.205	26.972	34.312	17.015	35.133	37.037	44.601	16.573	14.55	15.873	32.67	24.262	14.792	12.967	47.721	34.895
		54.876	62.169	20.212	41.112	52.51	19.841	10.592	30.492	48.942	37.948	31.203	32.378	30.495	25.132	61.201	19.841	35.578	48.303	17.196	46.296	39.683	26.659	32.834	14.182
		65.745	21.045	46.434	26.881	46.03	66.408	18.608	44.72	15.873	39.245	26.455	49.917	24.066	57.978	43.083	23.81	44.803	33.652	55.516	13.155	27.778	16.306	57.385	14.55
		7.937	27.778	21.023	56.015	64.187	19.524	28.858	27.144	45.502	72.922	49.499	31.564	26.263	26.91	12.768	20.653	59.035	43.561	34.296	15.901	48.463	70.106	78.545	55.04
		32.367	18.604	39.063	51.587	37.444	30.768	26.298	21.028	34.149	58.93	20.038	18.937	48.942	36.58	31.379	15.834	44.431	47.207	10.366	35.666	43.025	33.543	23.863	25.394
		47.931	56.878	60.786	71.79	43.349	17.771	21.719	27.363	34.428	53.063	30.423	18.707	34.797	46.127	10.582	24.25	21.352	71.525	20.094	27.692	66.743	15.873	63.141	48.665
		75.269	17.113	39.82	29.923	50.265	13.228	52.063	14.643	34.018	97.884	26.455	57.386	67.231	40.606	20.387	34.392	76.131	10.289	34.408	35.599	82.776	32.216	22.585	21.048
		15.725	49.695	60.393	55.184	24.806	22.487	21.301	42.576	22.487	70.549	27.778	55.268	18.707	28.726	28.613	16.514	42.885	45.279	13.095	39.683	60.939	19.757	61.21	41.494
		63.492	77.828	47.503	18.519	35.3	24.259	19.654	39.058	25.132	22.173	33.602	32.334	37.812	49.572	24.823	22.716	12.321	15.784	18.672	27.167	48.117	32.214	46.416	24.622
		19.524	71.287	55.382	76.57	18.519	19.026	38.776	56.745	21.594	97.561	38.537	45.328	12.835	25.132	42.932	19.841	15.577	42.859	20.142	53.483	30.423	34.179	20.958	40.098
		59.523	53.451	28.131	60.847	26.814	30.049	42.328	36.368	53.453	95.486	26.582	102.84	26.455	42.121	25.837	53.765	23.825	47.531	44.697	52.055	26.067	30.778	70.448	47.846
		40.043	46.031	48.438	54.437	66.635	12.353	34.926	25.132	20.949	47.83	44.326	34.84	44.213	32.449	17.196	65.185	22.448	38.247	37.885	27.598	57.278	83.871	36.639	26.474
		64.536	42.328	12.666	41.689	20.278	20.577	18.825	34.392	24.744	49.71	16.573	47.537	25.464	30.8	28.401	22.915	51.644	51.195	64.183	23.428	39.878	21.6	10.582	65.421
		22.855	71.108	58.059	66.674	26.189	19.915	20.577	32.909	33.873	28.479	23.81	8.418	15.453	51.372	21.451	28.496	48.074	10.624	80.259	35.364	52.739	30.069	58.603	20.287
		66.885	36.386	41.419	49.44	57.295	28.06	23.626	31.688	10.804	41.695	56.235	68.165	16.368	29.914	22.229	26.455	56.523	36.294	18.83	36.701	12.911	20.919	59.055	25.132
		78.115	83.021	50.292	52.304	26.455	19.281	10.582	11.905	13.045	79.833	49.572	10.641	19.477	37.611	26.373	45.028	66.001	24.501	15.873	50.634	61.732	13.612	18.934	58.144
		43.112	61.067	14.118	23.085	61.539	19.751	28.249	30.134	20.992	16.564	35.06	16.836	37.99	31.746	44.51	16.788	22.498	21.1	34.841	33.026	59.524	14.405	17.196	45.596
		24.849	41.926	16.92	26.455	30.14	15.267	10.386	39.683	25.132	53.201	29.493	27.267	35.714	44.589	61.563	35.714	48.117	27.217	34.515	32.728	60.012	23.874	16.503	54.363
		64.573	47.676	57.99	23.561	43.199	25.949	20.692	31.583	22.248	59.491	100.581	48.942	25.132	23.487	34.816	29.793	11.905	49.854	33.069	15.873	21.532	56.578	47.585	23.507
		62.743	52.338	44.458	49.466	31.6	15.873	11.905	23.113	31.172	71.428	36.38	15.834	36.571	46.607	17.447	29.076	23.13	32.736	22.487	27.155	24.761	66.821	47.913	55.922
		55.621	44.896	75.101	25.132	18.279	10.582	47.453	9.343	36.075	46.136	35.666	23.81	27.778	17.645	25.799	41.005	35.528	14.55	24.856	17.95	38.693	34.14	23.376	32.223
		58.171	41.137	46.997	43.919	66.483	19.665	18.519	18.519	34.392	57.273	11.747	34.392	17.16	18.519	33.291	25.833	26.448	12.216	48.661	11.238	53.382	13.629	26.64	50.73
		27.503	67.346	31.086	77.453	45.134	12.588	42.09	32.819	39.324	21.164	31.494	54.136	33.069	39.048	24.579	16.836	30.423	11.905	19.793	19.915	21.164	12.913	27.598	28.91
		52.697	82.23	61.459	64.62	48.097	19.966	26.081	17.028	30.707	72.764	80.867	17.771	19.531	47.429	29.079	27.482	43.651	37.995	34.371	10.582	19.607	50.006	26.091	36.843
		47.853	74.561	53.046	60.578	13.061	20.141	22.301	30.423	35.448	33.132	33.837	15.873	15.873	19.747	25.169	17.208	22.487	13.28	43.651	68.039	56.758	37.037	24.507	14.55
		59.908	61.235	10.073	60.561	26.863	13.924	33.84	11.905	51.853	46.891	89.685	58.201	25.449	31.7	24.453	26.633	35.978	39.875	19.895	10.868	74.347	60.022	56.962	44.96
		68.771	58.344	11.905	66.459	48.463	26.8	44.977	14.55	18.519	41.005	18.519	68.242	12.531	30.423	23.218	35.507	19.374	57.99	15.574	49.093	61.098	30.217	52.476	39.484
		28.317	75.074	36.886	70.258	64.806	15.445	38.752	30.313	29.546	42.275	56.67	22.036	37.306	18.937	27.728	50.265	25.542	18.519	19.994	17.991	53.9	34.288	49.579	17.637
		46.08	75.937	46.456	10.289	76.733	21.305	22.487	35.163	38.834	51.597	21.506	20.504	19.841	26.255	15.242	11.014	43.046	26.424	15.326	24.618	46.088	19.976	38.606	49.9
		64.481	69.815	15.873	23.853	51.443	25.934	13.951	36.093	33.124	59.825	48.12	28.995	24.034	15.212	35.571	11.838	28.725	21.238	36.516	12.277	34.099	27.109	54.95	64.942
		69.086	66.658	51.216	64.917	68.783	25.102	49.778	38.985	13.753	88.419	20.948	72.651	61.047	44.896	29.004	50.265	22.466	36.545	68.721	22.811	44.974	24.318	51.148	58.191
		32.746	43.148	58.155	37.86	47.485	11.714	36.478	11.241	24.829	17.664	37.524	52.834	42.318	57.121	15.901	14.521	19.024	10.46	24.821	17.973	26.316	18.93	15.396	35.273
		69.386	51.335	40.45	15.798	45.554	41.413	49.621	31.191	20.423	55.053	17.991	29.608	10.289	42.203	37.202	43.025	27.201	15.873	20.187	20	61.549	37.037	44.073	86.687
		81.644	44.373	50.74	23.831	13.228	18.257	15.901	41.185	32.291	49.39	38.706	18.519	31.625	16.752	33.672	25.214	43.352	15.873	22.061	26.643	57.985	18.347	38.834	49.86
		76.393	59.051	18.431	80.808	14.965	21.164	55.672	13.608	14.55	23.587	18.661	13.095	33.053	39.472	56.369	19.45	39.683	31.724	36.018	23.596	59.861	23.206	27.784	26.319
		52.579	12.615	50.32	50.12	79.736	19.537	42.055	30.192	31.232	17.196	80.57	29.89	26.06	33.729	71.492	34.664	9.669	21.435	30.423	34.319	27.778	27.017	31.998	46.997
		68.86	55.471	55.429		54.334	20.5	19.762	42.26	12.325	27.913	24.267	32.736	18.531	29.101	60.655	22.655	20.527	49.309	42.036	10.582	34.894	23.81	62.797	38.655
		70.28	49.131	74.826		60.685	39.601	15.675	39.214	30.765	65.744	27.665	34.392	28.87	14.908	34.7	29.221	18.285	54.522	37.413	15.069	34.392	21.076	16.54	21.513
		89.393	56.716	17.736		23.426	19.767	40.835	18.983	69.048	28.219	28.169	25.098	37.088	31.191	30.821	40.597	32.563	15.616	19.269	27.492	15.873	23.81	36.455	40.707
		61.732	18.519	46.296		28.446	16.622	59.906	9.138	14.921	48.328	21.164	67.68	31.746	30.405	31.924	39.444	10.04	21.221	32.736	21.562	53.25	37.5	42.651	22.773
		53.1	36.122	56.413		52.293	11.905	19.665	36.821	32.109	78.801	63.612	14.965	19.459	38.231	30.063	42.423	40.772	29.076	42.118	37.383	56.878	11.063	55.02	49.711
		77.319	90.252	58.543		62.654	22.085	22.782	17.156	12.394	80.343	15.501	72.605	41.59	26.206	44.827	38.882	24.386	18.519	14.134	38.359	39.922	14.333	43.912	11.714
		58.145	78.042	49.756		56.219	14.228	16.836	41.711	27.951	28.498	34.159	25.948	23.81	35.563	25.132	24.012	19.243	48.554	21.656	68.62	12.265	17.074	31.146	32.952
		50.265	49.196	57.99		59.426	22.162	51.924	22.487	19.841	57.657	79.356	23.758	23.478	58.045	9.259	22.448	14.55	44.189	38.817	14.978	57.608	31.325	66.272	
		63.051	47.43	25.912		61.445	13.018	42.949	18.38	36.873	56.12	22.487	15.565	37.986	47.302	12.693	48.565	43.96	52.866	19.596	13.478	71.867	42.372	51.976	
		21.754	44.596	59.524		24.499	23.475	15.082	18.707	22.402	64.105	60.876	11.952	12.154	41.392	21.458	29.523	45.778	17.99	27.83	27.52	32.677	62.341	37.331	
						63.484	10.582	58.972	16.836	61.687	37.522	60.319	13.158	15.876	54.249	25.132	51.026	15.671	22.911	45.875	26.205				
						45.483	10.611	17.196	38.742	25.6	14.55	45.198	16.598	21.206	19.841	32.823	31.621	12.968	64.815	13.005	13.593				
						44.29	40.546	50.421	38.931	39.295	28.45	56.905	44.008	12.552	42.551	18.851	52.635	34.607	21.479	17.8	49.78				
						50.222	21.06	38.075	27.291	34.392	71.823	63.183	26.842	36.367	59.773	23.81	13.228	41.436	10.849	11.905	30.866				
						89.832	14.55	30.995	46.296	37.881	50.196	28.677	29.875	26.309	50.146	41.84	43.651	24.334	28.712	25.132	26.519				
						15.786	11.388	33.069	31.358	24.582	71.958	37.167	34.999	24.706	11.424	23.81	16.794	45.638	14.55	29.531	36.478				
						35.235	19.465	55.284	20.577	39.768	38.793	12.488	15.496	28.06	21.164	30.995	17.132	17.938	24.849	21.023	32.407				
						26.707	15.386	28.802	20.124	43.936	38.36	29.705	57.69	14.306	21.747	49.731	40.241	37.019	43.651	41.773	18.707				
						33.29	22.015	14.55	63.168	33.231	80.461	48.81	38.945	68.354	40.708	25.252	12.372	17.196	22.389	10.008	12.159				
						94.813	38.266	19.841	41.277	38.948	31.533	24.473	27.506	13.228	41.005	21.495	31.746	23.199	60.801	47.14	16.573				
						45.663	46.983	52.941	10.582	44.249	73.217	27.507	13.228	12.216	13.228	57.99	24.143	26.455	38.366	55.941	26.455				
						46.164	24.18	54.249	35.926	61.461	23.095	28.208	39.648	44.281	51.245	19.513	14.55	44.302	26.629	37.452	43.961				
						48.234	17.196	34.598	28.934	39.683	83.433	57.869	8.059	42.428	25.638	32.394	18.495	46.709	62.743	11.714	29.588				
						39.36	29.581	28.704	13.095	35.145	32.643	31.952	54.596	22.985	24.888	47.13	24.586	23.213	47.992	28.693	18.306				
						77.053	29.101	20.714	21.504	35.152	51.682	32.432	26.455	27.872	31.066	18.96	35.173	44.974	19.824	35.381	29.101				
						38.15	25.459	38.18	31.924	14.758	54.613	50.749	39.445	35.522	21.164	14.55	17.602	58.416	34.392	37.499	18.519				
						52.91	39.69	16.908	12.457	47.587	57.395	45.766	27.949	36.456	13.864	15.402	22.063	11.224	24.156	38.121	15.879				
						48.66	33.789	26.068	30.775	55.556	83.076	39.048	41.986	79.79	33.243	45.831	51.443	14.55	26.635	49.813	42.09				
						55.34	18.299	26.455	38.68	31.696	12.529	13.228	38.037	25.844	27.215	45.193	43.559	28.49	26.763	22.487	27.82				
						77.832	13.095	30.964	53.314	28.813	66.248	35.562	17.42	36.971	18.519	29.581	44.808	34.112	44.551	11.714	25.674				
						59.861	41.133	54.906	32.511	22.402	17.196	45.635	10.663	17.196	42.582	41.861	25.132	52.206	32.179	19.814	28.644				
						61.996	11.818	32.542	47.619	21.96	14.681	30.866	66.958	30.067	15.482	41.81	29.101	37.93	13.018	41.003	9.237				

						31.372	13.22	22.715	20.577	22.462	36.826	25.138	42.585	33.505	21.891	15.873	18.097	12.092	38.236	53.215	25.132				
						54.354	14.281	14.55	23.81	23.249	54.904	27.402	29.646	29.619	17.375	15.873	48.329	55.553	34.392	13.228	23.81				
						56.12	26.189	31.38	26.065	11.834	40.031	40.617	45.412	57.963	36.83	23.85	23.647	25.254	24.491	25.132	31.746				
						42.315	27.778	43.329	19.527	10.582	17.75	38.313	35.604	17.888	64.873	37.612	39.779	40.031	24.266	12.777	19.841				
						58.06	13.967	23.303	33.359	11.905	28.884	20.711	10.891	20.236	24.682	44.762	15.902	31.87	43.712	55.74	14.506				
						73.647	15.784	32.834	37.018	47.619	50.103	20.086	50.655	24.631	23.665	18.658	43.361	26.455	26.337	22.849	39.655				
						46.691	30.423	27.778	23.025	23.81	39.754	25.132	23.248	13.22	27.103	34.392	22.487	11.905	34.906	19.513	48.757				
						10.084	23.17	41.392	13.799	17.196	10.077	61.732	8.712	37.651	30.192	47.047	25.254	18.242	36.703	43.142	14.965				
						57.431	30.423	23.079	20.19	23.853	38.189	40.778	29.187	24.706	24.259	49.689	69.388	36.593	11.294	58.797	23.81				
						75.649	29.387	44.923	38.494	32.367	52.322	10.805	46.692	60.428	29.112	27.618	16.836	27.778	23.626	29.101	25.132				
						51.443	21.08	65.71	38.066	39.325	15.25	53.475	43.38	19.845	25.213	45.428	46.641	43.957	39.162	35.866	17.882				
						39.641	16.209	9.259	30.632	27.778	37.593	38.348	15.755	22.488	37.037	27.787	8.214	56.693	22.478	10.867	21.72				
						30.155	16.412	31.753	10.661	28.613	45.61	40.661	16.836	31.762	17.196	37.037	42.09	42.134	39.627	28.56	11.905				
						50.92	45.831	21.888	22.487	26.906	86.058	42.591	57.29	17.417	37.037	25.951	12.814	47.487	44.741	28.057	18.519				
						26.285	42.86	38.963	31.766	13.393	10.651	29.2	14.973	21.164	40.881	46.296	46.296	61.422	54.785	14.812	25.743				
						93.858	13.188	20.387	19.245	19.433	25.15	42.947	44.296	32.592	25.992	18.488	11.834	23.082	21.583	21.381	14.281				
						55.134	47.619	20.504	44.963	26.189	26.758	20.921	35.075	25.444	36.784	31.687	21.759	40.407	50.508	43.786	12.425				
						55.184	23.383	24.431	43.508	29.654	21.061	28.833	56.878	16.741	22.4	10.651	15.873	32.621	44.907	34.307	13.228				
						65.968	82.095	10.582	30.423	30.976	9.985	27.835	33.026	45.446	65.968	26.752	41.026	67.344	31.746	31.384	38.94				
						63.897	30.866	17.196	42.838	22.24	41.005	44.967	14.55	32.908	27.837	23.735	28.06	22.747	51.553	52.91	32.058				
						33.883	35.015	17.617	45.838	63.602	27.761	18.44	23.354	30.369	37.775	41.349	34.352	23.797	32.288	14.55	16.554				
						20.785	26.455	37.874	32.397	19.245	60.197	24.007	20.226	18.285	36.828	19.535	26.21	43.51	26.075	41.014	22.107				
						55.572	18.343	22.299	58.155	45.893	25.254	71.579	13.475	35.337	31.069	24.056	30.821	24.497	38.665	23.383	51.19				
						53.938	23.286	28.561	20.121	26.189	13.051	25.799	11.688	37.982	23.742	21.514	23.01	35.185	21.553	14.559	20.556				
						56.519	22.604	25.132	36.268	23.81	26.14	60.247	23.632	41.415	26.399	23.374	63.132	49.409	45.452	22.415	25.942				
						43.025	18.381	24.232	17.354	21.839	96.685	24.995	21.997	12.968	52.403	19.376	56.29	44.485	29.391	12.431	17.622				
						36.176	17.47	24.631	47.14	25.322	13.849	50.629	19.881	44.963	15.574	47.163	36.478	39.591	31.026	43.651	37.857				
						46.832	36.613	54.129	25.254	29.68	19.841	36.9	19.29	17.381	17.196	34.84	30.423	28.911	30.657	26.455	29.598				
						52.835	57.435	30.316	22.882	13.228	16.157	33.675	29.833	12.853	49.638	36.461	11.905	40.46	30.73	15.505	10.54				
						56.94	25.132	32.865	17.151	35.542	33.697	22.92	80.357	34.294	27.778	36.093	19.841	45.831	32.011	22.541	21.134				
						53.566	20.045	37.746	19.874	16.35	65.552	91.837	41.005	28.995	31.355	19.841	30.642	36.498	30.434	50.99	44.896				
						52.304	34.308	26.455	39.638	34.217	30.115	35.068	16.566	66.826	39.11	23.056	39.683	18.707	25.322	19.534	23.848				
						13.344	42.398	19.841	25.78	21.164	14.55	41.062	42.136	48.215	52.245	19.096	28.624	32.657	24.856	14.118	33.296				
						27.572	28.213	22.487	27.862	24.132	29.503	20.939	13.919	13.018	23.27	22.487	44.264	41.005	53.314	20.36	34.853				
						35.174	19.285	32.246	14.742	15.801	20.924	55.015	11.843	10.582	19.841	13.211	64.815	41.005	36.923	46.605	45.196				
						44.346	15.716	32.457	26.189	36.96	29.101	19.841	40.874	23.072	55.459	25.673	37.25	67.303	35.075	56.878	33.048				
						62.004	15.784	58.234	31.878	31.438	20.966	31.476	73.781	29.101	11.905	20.31	34.392	26.737	47.34	44.974	37.799				
						39.037	29.299	9.625	13.659	67.476	27.483	68.55	32.572	31.503	28.647	35.137	23.81	37.214	63.675	14.792	25.132				
						32.155	35.24	22.488	20.91	16.244	35.594	34.294	19.098	12.968	32.548	30.29	26.455	48.173	8.985	31.984	31.728				
						53.58	27.473	54.209	29.191	16.864	15.7	33.727	32.256	26.189	47.126	20.367	32.82	26.534	47.274	32.79	41.636				
						70.224	13.578	17.196	25.781	30.423	22.842	9.259	31.443	40.219	29.27	20.08	30.777	32.888	42.55	18.045	20.855				
						50.265	15.799	19.648	13.155	11.905	42.328	35.964	13.919	19.434	38.755	53.068	23.142	20.119	56.924	14.792	41.837				
						56.647	15.645	21.364	40.994	56.263	46.901	40.819	39.683	32.925	33.069	85.115	22.593	41.871	52.91	43.594	46.728				
						30.178	37.984	58.374	39.638	14.99	50.701	10.958	22.184	34.876	31.746	16.836	40.21	36.486	58.084	47.619	26.366				
						48.995	14.55	27.778	23.587	43.895	33.19	68.508	61.927	21.539	24.798	47.619	19.839	18.279	60.77	13.301	52.655				
						33.069	17.26	23.383	27.778	36.602	41.931	62.633	32.281	28.729	20.112	35.075	30.561	46.521	33.069	27.778	27.077				
						28.631	12.984	23.533	30.099	37.318	50.708	29.588	46.296	26.891	23.29	35.714	20.5	41.277	47.538	20.577	24.267				
						37.037	37.562	41.863	34.965	19.195	30.125	27.725	63.506	38.458	24.192	36.019	43.651	37.665	31.056	27.191	45.974				
						23.93	22.829	17.49	40.352	44.896	21.283	20.419	48.311	38.983	11.672	42.09	30.423	15.733	26.606	40.219	32.961				
						33.069	28.386	10.262	22.748	31.801	55.184	54.997	22.487	53.856	50.683	51.072	36.096	38.458	31.368	25.132	28.364				
						32.189	9.259	37.44	15.81	28.293	39.855	56.12	37.235	32.868	17.16	11.787	33.069	46.879	30.68	31.098	30.423				
						37.798	25.659	33.957	30.641	14.681	14.965	44.957	64.815	20.286	39.194	27.217	19.841	29.186	36.868	31.415	43.651				
						74.838	25.132	20.624	27.291	52.115	48.428	32.088	32.736	25.132	16.573	38.12	33.069	49.666	42.847	15.873	25.157				
						28.995	42.934	18.93	13.028	26.637	31.997	30.391	44.601	27.817	10.879	25.011	15.293	45.115	23.81	9.259	30.423				
										9.743	28.35	76.264	45.89	25.883	20.714	20.94	31.746	44.347	20.885	35.542	45.831				
										14.197	21.164	61.879	15.766	19.527	11.233	27.183	28.45	40.812	29.101	17.151	35.488				
										44.965	29.494	35.182	34.353	26.455	14.965	10.582	41.834	47.785	21.936	16.412	40.235				
										11.905	19.321	31.542	54.861	21.006	62.667	25.446	23.72	23.81	25.132	47.619	29.101				
										39.977	28.87	13.032	36.995	21.513	19.642	43.723	29.101	52.104	33.083	19.527	12.693				
										31.358	11.453	38.378	34.607	22.487	28.124	10.837	22.779	10.582	13.022	34.392	17.151				
										25.942	43.567	14.506	28.06	43.138	33.346	15.656	22.448	36.811	54.644	13.891	60.685				
										68.091	25.676	30.778	11.953	15.973	29.42	69.501	18.111	46.296	28.925	13.834	30.286				
										23.074	70.936	35.062	51.443	32.407	48.216	14.323	45.735	46.769	33.961	26.623	44.251				
										28.263	11.605	31.962	61.875	22.975	14.119	27.916	33.691	37.413	33.672	13.017	47.711				
										37.137	34.392	20.622	42.49	53.936	41.071	22.813	20.859	34.868	42.421	19.654	32.229				
										26.789	13.158	35.084	33.267	74.047	38.426	14.788	14.118	43.148	40.023	38.641	28.414				
										34.756	16.427	19.839	25.64	48.637	13.022	25.353	46.137	33.72	32.526	26.455	26.455				
										32.638	21.164	70.737	24.259	15.873	32.693	48.234	31.493	31.269	26.641	31.269	56.868				
										40.598	38.36	14.965	70.275	13.228	40.156	56.479	19.436	30.431	40.232	13.228	35.514				
										38.355	28.613	23.872	29.512	15.432	34.133	26.67	16.988	40.054	15.661	12.838	23.853				
										26.27	10.884	26.669	23.383	35.109	26.422	29.101	17.033	49.504	38.795	20.889	29.726				

										20.168	21.164	30.423	37.547	14.559	29.847	15.269	9.911	43.73	17.464	40.424	26.991				
										31.039	30.369	59.524	39.382	29.03	33.903	9.983	19.642	17.909	46.572	34.312	57.158				
										32.221	63.956	30.423	53.745	23.428	48.315	21.192	13.017	25.446	48.535	34.607	35.296				
										15.873	53.282	21.717	47.835	36.478	24.289	23.81	54.233	30.423	26.125	26.926	10.651				
										32.686	29.698	23.471	83.465	67.409	18.585	45.787	42.626	27.778	32.84	27.211	10.335				
										27.124	27.411	28.847	27.997	64.494	24.977	15.015	17.476	31.753	31.903	20.577	70.812				
										14.55	79.801	39.4	27.818	13.228	38.795	22.98	29.416	35.371	41.736	42.24	34.392				
										37.037	23.7	27.951	36.69	28.822	57.055	66.408	13.578	33.332	39.012	36.834	29.801				
										30.359	27.778	36.89	30.667	58.377	33.767	27.711	23.82	43.923	42.328	22.487	14.082				
										21.151	14.69	44.231	44.109	16.514	14.55	20.95	17.196	9.259	33.415	31.949	58.255				
										47.593	22.448	46.483	39.646	30.144	17.408	14.03	40.848	60.466	9.911	32.895	25.132				
										23.847	23.836	26.419	14.55	12.159	50.867	27.848	24.914	34.43	31.76	42.181	30.777				
										67.922	58.568	29.535	43.517	53.105	17.872	41.874	37.803	34.097	41.29	37.676	29.101				
										59.862	17.622	51.329	54.249	39.683	42.328	29.451	14.225	13.228	25.239	42.867	19.202				
										13.179	30.196	54.687	75.427	33.069	20.647	26.455	42.328	21.164	36.811	49.358	13.228				
										69.881	9.259	33.069	26.781	31.687	41.775	22.984	29.2	52.602	14.792	30.151	18.157				
										16.042	26.624	81.175	40.422	30.342	53.584	42.939	23.299	26.455	42.058	41.307	9.259				
										41.436	55.549	19.027	67.063	37.783	31.7	14.768	28.501	53.163	25.597	51.441	17.938				
										42.23	41.108	44.623	31.79	40.663	31.437	34.162	47.354	41.001	29.886	38.355	14.03				
										47.368	60.876	51.711	18.519	68.978	52.91	31.118	32.873	55.556	30.123	26.425	37.173				
										21.316	44.275	24.168	30.486	16.573	50.912	31.033	37.705	38.968	51.608	11.224	42.999				
										42.351	46.253	44.458	17.507	19.793	21.721	31.741	48.546	40.97	18.608	28.045	32.051				
										18.825	87.266	17.642	13.553	36.587	44.918	19.067	11.981	51.43	20.482	21.164	19.693				
										65.773	89.489	46.296	32.8	47.832	30.439	18.431	42.328	43.61	38.647	49.449	47.987				
										23.672	44.561	26.603	45.317	18.292	19.524	36.099	18.519	37.279	15.873	11.801	35.764				
										38.931	45.502	7.937	32.727	31.746	42.815	17.771	32.442	33.37	16.741	29.35	47.894				
										23.81	31.958	43.025	20.08	22.896	23.548	29.295	18.524	35.614	13.768	43.994	17.771				
										77.632	65.932	9.353	32.15	30.067	32.928	24.77	33.345	55.69	13.095	41.401	48.097				
										76.697	67.365	76.239	50.916	34.023	13.798	58.568	16.836	11.834	35.714	25.782	19.052				
										29.749	49.848	30.067	66.138	25.517	26.908	32.423	26.646	37.027	34.584	24.52	12.809				
										34.295	64.137	44.974	44.323	11.248	29.793	13.155	41.235	34.906	35.002	16.836	22.52				
										18.347	55.556	35.788	30.866	20.157	34.023	32.821	43.923	20.95	14.03	37.986	28.148				
										22.776	88.6	35.714	9.353	26.455	33.329	32.408	9.601	41.769	26.965	35.792	54.539				
										10.12	26.009	44.896	12.835	54.865	17.175	18.144	15.883	38.809	16.042	46.857	34.973				
										47.424	14.03	69.396	28.404	50.08	15.873	35.954	12.49	24.947	22.195	17.224	21.754				
										47.466	50.265	38.761	15.697	14.55	60.86	35.714	30.866	57.972	28.06	20.883	60.36				
										43.683	41.005	54.556	21.717	16.831	23.27	14.03	30.423	39.953	16.699	43.481	38.749				
										17.712	40.087	35.714	70.274	11.905	27.732	27.559	61.038	38.91	30.16	31.746	27.89				
										38.221	25.194	33.929	10.964	51.043	24.262	16.05	23.413	23.77	39.284	26.402	54.871				
										48.598	36.336	12.24	55.082	46.15	44.51	40.422	44.779	66.723	19.535	35.578	59.524				
										53.899	79.938	62.576	47.509	28.931	29.792	27.353	42.592	23.81	41.154	50.265	33.883				
										23.572	21.101	41.383	38.416	28.06	24.821	43.786	24.066	19.177	22.468	27.778	38.085				
										35.977	59.923	20.708	24.318	26.922	22.487	18.415	46.615	12.006	39.051	38.793	30.201				
										35.496	13.018	47.762	38.69	20.577	17.771	15.873	17.184	47.279	13.228	16.514	71.047				
										30.392	14.243	15.465	15.873	53.351	39.02	18.897	27.039	27.778	23.204	39.963	41.005				
										61.935	53.84	34.392	75.317	21.394	33.75	28.169	28.421	27.038	29.101	23.81	18.164				
										17.581	11.905	50.146	17.716	24.52	35.272	16.25	15.406	36.034	37.704	46.741	28.798				
										32.581	44.226	82.344	21.513	34.392	14.017	23.452	17.219	73.303	45.647	71.597	46.265				
										29.843	44.772	56.253	24.311	35.603	25.896	37.47	11.975	32.389	34.392	42.799	35.698				
										22.297	65.9	49.483	37.061	51.587	16.378	23.853	44.402	29.172	25.254	46.157	32.083				
										56.733	26.291	38.72	16.836	49.572	62.047	27.742	25.976	28.207	38.642	41.841	22.432				
										29	36.255	52.155	28.06	20.5	22.268	19.574	41.005	44.064	20.652	44.974	13.569				
										21.164	74.381	46.455	11.555	51.114	24.129	13.228	20.826	56.38	34.392	41.414	35.977				
										28.671	27.463	40.096	22.775	19.841	48.78	14.03	20.08	9.474	30.726	26.455	37.037				
										35.071	41.126	15.873	34.537	19.331	31.471	35.153	17.154	44.679	15.104	57.465	48.525				
										9.517	39.577	10.582	18.519	40.7	22.361	33.902	51.479	38.458	26.065	45.612	24.713				
										47.718	23.521	41.432	68.279	12.921	13.615	33.804	29.798	21.164	29.52	58.746	24.787				
										35.606	13.025	17.571	56.12	37.037	40.219	13.701	15.873	19.448	22.448	37.545	53.251				
										30.21	27.245	23.983	44.136	31.466	18.661	31.901	11.181	52.693	33.629	48.87	32.966				
										15.064	38.135	39.343	25.132	28.703	40.033	40.963	36.122	56.12	39.645	35.598	49.149				
										64.539	26.542	30.51	28.7	44.765	20.335	57.963	19.841	22.75	44.278	30.359	23.122				
										35.028	63.117	14.965	18.735	18.706	13.393	40.158	13.951	27.835	29.5	38.266	44.059				
										28.06	47.977	22.087	61.74	13.228	41.398	27.941	27.161	35.931	19.642	48.518	35.596				
										37.797	11.817	62.127	48.289	21.513	22.319	54.954	14.576	9.646	47.065	56.675	12.838				
										23.224	13.018	12.814	29.101	25.946	28.025	70.149	13.262	21.928	17.005	45.928	33.069				
										26.189	63.746	18.519	16.663	42.328	27.794	11.161	31.94	11.224	44.322	41.154	14.55				
										27.713	58.253	23.707	42.225	11.224	15.402	36.937	22.374	33.765	16.172	39.48	13.277				
										31.746	45.075	38.454	47.702	36.658	56.675	29.101	39.963	23.534	31.949	24.878	39.001				
										37.037	19.793	32.73	55.367	28.549	21.142	16.496	27.593	51.037	37.366	29.101	32.432				
										14.011	25.937	22.962	17.771	69.701	43.025	48.916	44.785	21.164	45.95	37.946	36.976				
										33.063	15.873	43.562	65.854	13.228	26.623	27.778	25.531								

										39.187	35.973	22.487	35.368	35.565	37.156	13.228	30.423								
										44.956	42.899	38.275	23.072	39.749	42.056	13.228	37.291								
										15.293	24.583	29.101	27.265	27.859	17.905	25.416	39.131								
										12.134	18.707	14.908	33.069	26.455	44.373	27.189	29.094								
										42.09	22.448	15.432	37.487	22.018	53.447	13.073	29.547								
										25.132	26.21	32.599	27.778	28.327	21.758	50.016	21.164								
										39.8	26.419	33.468	24.031	37.173	26.737	36.948	27.62								
										13.715	34.241	44.973	15.863	16.936	22.983	12.978	32.542								
										27.183	11.077	52.845	17.642	34.415	48.281	15.482	41.639								
										31.578	9.488	30.959	31.362	31.877	11.009	54.655	33.672								
										12.289	29.588	46.74	55.016	11.162	27.034	11.905	36.7								
										28.809	17.99	43.11	54.43	32.313	12.456	35.964	13.966								
										31.173	28.25	14.521	17.771	26.633	17.576	31.064	31.126								
										23.853	14.55	23.294	37.306	35.242	20.519	19.841	17.196								
										22.563	25.649	10.289	39.375	21.902	61.285	38.36	18.41								
										76.72	15.706	38.461	77.767	44.109	41.819	44.01	14.547								
										33.09	19.758	20.977	10.582	32.264	26.455	48.895	42.444								
										57.963	37.044	23.792	27.345	32.693	24.269	54.128	20.377								
										41.839	13.387	46.348	26.627	42.885	70.346	30.127	16.37								
										13.811	29.76	44.675	17.196	29.304	27.778	11.905	46.64								
										31.746	12.782	24.771	36.287	39.034	34.752	14.409	25.763								
										26.926	27.474	25.621	21.188	44.103	24.143	23.81	17.196								
										27.273	25.132	43.652		46.766	27.455	18.519	20.336								
										35.714	56.132	29.423		41.005	25.439	54.482	26.189								
														17.926	28.753	50.855	35.265								
														21.967	28.661	35.281	13.018								
														29.858	19.841	39.284	24.318								
														70.571	30.098	24.012	22.487								
														23.81	14.205	53.98	14.934								
														34.067	39.559	24.878	38.348								
														46.006	36.297	22.997	46.338								
														30.022	48.73	23.81	14.199								
														50.265	19.323	20.744	24.858								
														39.273	34.242	12.984	24.295								
														20.535	21.164	47.207	12.159								
														15.645	39.578	13.023	19.841								
														23.541	52.232	21.163	22.464								
														63.252	26.189	39	36.207								
														33.773	42.026	27.11	14.55								
														61.489	44.691	17.48	26.618								
														34.317	40.747	41.005	12.088								
														58.246	36.239	28.658	10.346								
														23.698	7.937	18.922	51.517								
														38.91	36.489	23.876	26.189								
														12.125	36.552	26.315	41.005								
														21.08	38.978	48.309	13.228								
														10.582	38.194	27.138	19.841								
														44.963	29.377	19.158	15.616								
														30.866	13.753	43.629	33.227								
														34.926	19.93	46.747	30.768								
														36.336	29.561	36.921	20.577								
														37.864	34.684	20.535	18.603								
														21.752	22.24	23.047	47.371								
														71.085	13.402	39.683	49.422								
														27.062	53.671	49.249	34.319								
														12.362	41.73	36.607	19.222								
														25.325	19.504	35.606	17.196								
														25.029	21.754	23.81	15.066								
														50.882	14.939	19.446	40.884								
														31.492	38.14	23.587	19.321								
														37.154	43.648	35.854	42.209								
														68.279	26.169	51.158	32.736								
														43.651	26.189	24.353	45.306								
														46.021	18.083	43.382	21.048								
														27.637	18.582	19.572	13.569								
														46.944	59.237	22.961	21.164								
														20.419	35.463	28.06	71.587								
														30.769	14.55	42.617	12.092								
														36.526	33.482	23.018	24.798								
														18.343	24.623	42.435	22.593								
														13.275	44.698	36.376	58.291								

														20.391	22.497	27.778	24.706								
														33.54	34.069	39.535	27.778								
														70.653	20.2	15.382	33.984								
														48.743	29.714	17.148	22.487								
														22.492	21.677	34.503	23.075								
														18.932	29.584	54.445	47.874								
														18.219	67.344	25.132	28.061								
														61.764	17.509	19.082	15.072								
														21.026	21.542	28.753	67.344								
														23.762	27.485	33.069	11.299								
														20.152	53.681	12.159	30.993								
														14.239	38.22	53.742	27.895								
														20.577	24.614	54.749	21.145								
														38.04	27.796	59.169	38.36								
														10.641	44.866	37.212	24.318								
														17.196	43.928	21.512	40.098								
														69.406	37.037	50.772	35.429								
														42.016	33.124	16.223	28.45								
														22.487	48.266	23.008	9.717								
														12.438	45.873	24.151	26.697								
														35.21	28.393	54.022	30.865								
														26.271	49.633	41.469	22.256								
														68.746	26.221	9.292	17.102								
														44.904	16.627	24.448	19.412								
														23.515	46.894	25.238	35.367								
														15.632	43.411	23.595	21.298								
														47.616	35.23	42.885	14.55								
														27.778	35.714	47.673	29.376								
														28.51	37.037	24.049	29.564								
														29.285	44.026	32.214	40.895								
														37.345	28.907	22.943	18.998								
														38.185	30.423	24.862	24.259								
														12.717	22.362	18.926	30.701								
														18.431	35.937	31.484	39.296								
														36.478	38.348	17.563	46.296								
														17.527	33.069	52.237	53.435								
														25.102	23.81	26.474	47.856								
														30.765	34.607	29.93	15.834								
														28.488	45.33	22.487	28.717								
														29.101	13.167	20.919	34.653								
														50.253	19.841	27.528	17.767								
														40.514	38.993	17.771	54.249								
														25.132	24.073	20.708	26.455								
														31.509	61.579	31.801	38.31								
														29.257	48.111	36.239	11.144								
														16.346	35.542	20.52	38.9								
														49.738	27.9	13.242	32.747								
														46.017	34.126	17.196	25.132								
														20.256	33.672	31.801	14.944								

